# Endocrine-Sensitive Disease Rate in Postmenopausal Patients With Estrogen Receptor–Rich/ERBB2-Negative Breast Cancer Receiving Neoadjuvant Anastrozole, Fulvestrant, or Their Combination: A Phase 3 Randomized Clinical Trial

**DOI:** 10.1001/jamaoncol.2023.6038

**Published:** 2024-01-18

**Authors:** Cynthia X. Ma, Vera J. Suman, Souzan Sanati, Kiran Vij, Meenakshi Anurag, A. Marilyn Leitch, Gary W. Unzeitig, Jeremy Hoog, Aranzazu Fernandez-Martinez, Cheng Fan, Richard A. Gibbs, Mark A. Watson, Travis J. Dockter, Olwen Hahn, Joseph M. Guenther, Abigail Caudle, Erika Crouch, Amy Tiersten, Monica Mita, Wajeeha Razaq, Tina J. Hieken, Yang Wang, Mothaffar F. Rimawi, Anna Weiss, Eric P. Winer, Kelly K. Hunt, Charles M. Perou, Matthew J. Ellis, Ann H. Partridge, Lisa A. Carey

**Affiliations:** 1 Washington University School of Medicine, St Louis, Missouri; 2 Alliance Statistics and Data Management Center, Mayo Clinic, Rochester, Minnesota; 3 Cedars-Sinai Medical Center, Los Angeles, California; 4 Baylor College of Medicine, Houston, Texas; 5 University of Texas Southwestern Medical Center, Dallas; 6 Doctor’s Hospital of Laredo, Laredo, Texas; 7 University of North Carolina at Chapel Hill; 8 University of Chicago, Chicago, Illinois; 9 Saint Elizabeth Medical Center South, Edgewood, Kentucky; 10 MD Anderson Cancer Center, Houston, Texas; 11 Mount Sinai Hospital, New York, New York; 12 University of Oklahoma Health Sciences Center, Oklahoma City; 13 Mayo Clinic, Rochester, Minnesota; 14 Presbyterian Kaseman Hospital, Albuquerque, New Mexico; 15 University of Rochester, Rochester, New York; 16 Dana-Farber/Partners CancerCare, Boston, Massachusetts; 17 Yale School of Medicine, New Hartford, Connecticut

## Abstract

**Importance:**

Adding fulvestrant to anastrozole (A+F) improved survival in postmenopausal women with advanced estrogen receptor (ER)–positive/ERBB2 (formerly HER2)–negative breast cancer. However, the combination has not been tested in early-stage disease.

**Objective:**

To determine whether neoadjuvant fulvestrant or A+F increases the rate of pathologic complete response or ypT1-2N0/N1mic/Ki67 2.7% or less residual disease (referred to as endocrine-sensitive disease) over anastrozole alone.

**Design, Setting, and Participants:**

A phase 3 randomized clinical trial assessing differences in clinical and correlative outcomes between each of the fulvestrant-containing arms and the anastrozole arm. Postmenopausal women with clinical stage II to III, ER-rich (Allred score 6-8 or >66%)/ERBB2-negative breast cancer were included. All analyses were based on data frozen on March 2, 2023.

**Interventions:**

Patients received anastrozole, fulvestrant, or a combination for 6 months preoperatively. Tumor Ki67 was assessed at week 4 and optionally at week 12, and if greater than 10% at either time point, the patient switched to neoadjuvant chemotherapy or immediate surgery.

**Main Outcomes and Measures:**

The primary outcome was the endocrine-sensitive disease rate (ESDR). A secondary outcome was the percentage change in Ki67 after 4 weeks of neoadjuvant endocrine therapy (NET) (week 4 Ki67 suppression).

**Results:**

Between February 2014 and November 2018, 1362 female patients (mean [SD] age, 65.0 [8.2] years) were enrolled. Among the 1298 evaluable patients, ESDRs were 18.7% (95% CI, 15.1%-22.7%), 22.8% (95% CI, 18.9%-27.1%), and 20.5% (95% CI, 16.8%-24.6%) with anastrozole, fulvestrant, and A+F, respectively. Compared to anastrozole, neither fulvestrant-containing regimen significantly improved ESDR or week 4 Ki67 suppression. The rate of week 4 or week 12 Ki67 greater than 10% was 25.1%, 24.2%, and 15.7% with anastrozole, fulvestrant, and A+F, respectively. Pathologic complete response/residual cancer burden class I occurred in 8 of 167 patients and 17 of 167 patients, respectively (15.0%; 95% CI, 9.9%-21.3%), after switching to neoadjuvant chemotherapy due to week 4 or week 12 Ki67 greater than 10%. PAM50 subtyping derived from RNA sequencing of baseline biopsies available for 753 patients (58%) identified 394 luminal A, 304 luminal B, and 55 nonluminal tumors. A+F led to a greater week 4 Ki67 suppression than anastrozole alone in luminal B tumors (median [IQR], −90.4% [−95.2 to −81.9%] vs −76.7% [−89.0 to −55.6%]; *P* < .001), but not luminal A tumors. Thirty-six nonluminal tumors (65.5%) had a week 4 or week 12 Ki67 greater than 10%.

**Conclusions and Relevance:**

In this randomized clinical trial, neither fulvestrant nor A+F significantly improved the 6-month ESDR over anastrozole in ER-rich/ERBB2-negative breast cancer. Aromatase inhibition remains the standard-of-care NET. Differential NET response by PAM50 subtype in exploratory analyses warrants further investigation.

**Trial Registration:**

ClinicalTrials.gov Identifier: NCT01953588

## Key Points

**Question:**

Is fulvestrant, alone or in combination with the aromatase inhibitor anastrozole, superior to anastrozole alone as neoadjuvant endocrine therapy (NET) in postmenopausal women with estrogen receptor (ER)–rich/ERBB2-negative breast cancer, in terms of endocrine-sensitive disease rate (ESDR)?

**Findings:**

In this phase 3 randomized clinical trial of 1362 patients, the ESDR following 6 months of neoadjuvant fulvestrant or anastrozole plus fulvestrant was 22.8% and 20.5%, respectively; neither was significantly higher than the 18.7% ESDR with anastrozole alone.

**Meaning:**

Aromatase inhibition remains the standard-of-care NET for postmenopausal ER-rich/ERBB2-negative breast cancer.

## Teaser

This randomized clinical trial assesses whether neoadjuvant fulvestrant or anastrozole plus fulvestrant vs anastrozole alone increases endocrine-sensitive disease rate (ESDR) in postmenopausal women with estrogen receptor (ER)–rich/ERBB2-negative breast cancer.

## Introduction

Aromatase inhibition (AI) is standard of care for postmenopausal patients with early-stage estrogen receptor (ER)–positive/ERBB2 (formerly HER2)–negative breast cancer (BC).^1^ However, approximately 20% of patients experience disease recurrence.^1,2,3,4,5^ Selective ER degraders (SERDs) have the potential to further improve outcomes by inhibiting both estrogen-dependent and estrogen-independent ER signaling.^6^ The SERD fulvestrant improved progression-free survival compared to anastrozole as first-line therapy for postmenopausal women with advanced hormone receptor (HR)–positive BC in the FALCON trial.^7^ The combination of anastrozole plus fulvestrant (A+F) was also investigated in the metastatic setting based on preclinical evidence that fulvestrant is more effective in a low-estrogen environment,^8^ and that AI plus fulvestrant was more effective than monotherapy.^9,10^ The SWOG S0226 trial demonstrated that A+F improved survival compared to anastrozole as first-line therapy for advanced HR-positive BC. Benefits were particularly notable in the endocrine therapy (ET)–naive population.^11,12^ In contrast, the FACT trial found no significant improvement in time to progression with the addition of fulvestrant to anastrozole in the first-line metastatic setting, but few patients were ET naive.^13^ Both trials evaluated fulvestrant at 250 mg, raising the question of whether anastrozole would add to the efficacy of fulvestrant at the currently approved 500-mg monthly dose.^14,15^

Neoadjuvant ET (NET) trials provide opportunities to assess individual endocrine sensitivities in treatment-naive early-stage ER-positive/ERBB2-negative BC.^16^ ET resistance can be defined by tumor Ki67 greater than 10% following 2 to 4 weeks of NET, which is a prospectively validated biomarker for increased recurrence risk in postmenopausal individuals.^17^ In addition, a higher degree of Ki67 suppression in randomized NET trials predicted improved adjuvant efficacy of AI compared to tamoxifen.^16^ The finding that pathological stage, residual tumor Ki67, and ER levels following 4 to 6 months of neoadjuvant AI or tamoxifen treatment were all independent prognostic factors led to the development of the preoperative endocrine prognostic index (PEPI) to determine the risk of relapse.^18^ A PEPI 0, defined as ypT1-2 N0, residual tumor ER positive, and Ki67 of 2.7% or less, was associated with a 5-year relapse risk less than 5%.^18,19^ PEPI therefore offers a novel end point for NET trials, as pathologic complete response (pCR) is rare. In designing the ALTERNATE (Alliance A011106) trial reported here, we used modified PEPI (mPEPI) to exclude the ER component because fulvestrant reduces ER levels. The mPEPI remained prognostic in previous NET trials because loss of ER to below an Allred score of 3 was rarely the exclusive cause of PEPI greater than 0.^18,20^

Herein is the primary analysis of the neoadjuvant phase of the ALTERNATE trial designed to determine whether fulvestrant alone or A+F increases the rate of endocrine-sensitive disease (ESD), defined as pCR or mPEPI 0 (ypT1-2N0/N1mic/Ki67 ≤2.7%). Secondary end points included Ki67 suppression after 4 weeks of NET and the pCR rate after switching to neoadjuvant chemotherapy (NCT) due to a week 4 or week 12 Ki67 greater than 10%. As ER-positive BC is molecularly heterogeneous, ALTERNATE correlative studies included preplanned genomic and transcriptomic analyses to gain insights into ET resistance mechanisms. Since ACOSOG Z1031 demonstrated that the rate of PEPI 0 and Ki67 suppression from neoadjuvant AI differed by PAM50 subtype,^21^ the association between RNA sequencing–based PAM50 subtype and week 4 Ki67 suppression was explored.

## Methods

### Study Design

This multi-institutional open-label phase 3 randomized clinical trial enrolled postmenopausal women with treatment-naive palpable clinical T2-T4c, any N, M0, ER-rich (Allred score 6-8 or >66%), ERBB2-negative BC. Full eligibility criteria can be found in protocol section 3.0 (Supplement 1). Each participant was asked to select her race and ethnicity from among the National Cancer Institute–defined categories. Participants were free to not report this information or to specify something other than the predefined categories. Patients were randomly assigned (1:1:1) to the anastrozole, fulvestrant, or A+F arm, using the Pocock-Simon dynamic allocation procedure, balancing the marginal distributions of the stratification factors between arms. The stratification factors were clinical tumor stage (T2 vs T3 vs T4a-c), lymph node status (positive vs negative), and Eastern Cooperative Oncology Group performance status (0-1 vs 2). Treatments were anastrozole, 1 mg by mouth daily; fulvestrant, 500 mg intramuscularly on days 1 and 15 of cycle 1, then day 1 of subsequent cycles; or A+F (same doses) for 6 cycles (cycle length: 28 days) followed by surgery (eFigure 1 in Supplement 2). Research BC biopsies were required pretreatment, at week 4, and at the time of surgery, and optionally at week 12 for central Ki67 assessment. Patients with week 4 or week 12 Ki67 greater than 10% were to switch to NCT or surgery. NCT regimens were paclitaxel for 12 weeks or per standard of care. Patients with week 4 or week 12 Ki67 10% or less or insufficient tumor cells to determine week 4 or week 12 Ki67 continued their assigned NET. Bidimensional breast lesion measurements were performed on day 1 of each cycle prior to surgery. Progression on examination was confirmed by mammography or ultrasonography. Breast surgery was per standard of care. Sentinel lymph node with or without axillary lymph node dissection was required to ensure ability to determine PEPI score and residual cancer burden (RCB).^22^ Central Ki67 and ER assessment and RNA extraction and sequencing for PAM50 subtype are described in Supplement 1. The trial was conducted in accordance with the Declaration of Helsinki and Good Clinical Practice guidelines. The protocol was approved by the National Cancer Institute Adult–Late Phase central institutional review board (IRB) and local IRBs as appropriate. Each participant signed an IRB-approved, protocol-specific informed consent document in accordance with federal and institutional guidelines. This study followed the Consolidated Standards of Reporting Trials (CONSORT) reporting guideline.

### Statistical Analysis

All eligible patients who began protocol treatment were evaluable and included in the analysis cohort (Figure). The primary outcome was ESDR defined as the proportion of patients with pCR (no invasive disease in breast or lymph nodes) or mPEPI 0. Patients with week 4 or week 12 Ki67 greater than 10%, confirmed progressive disease (PD), mPEPI greater than 0, or insufficient data to determine mPEPI or who discontinued protocol treatment for any reason, without completing surgery, were considered as not having ESD.

**Figure.**
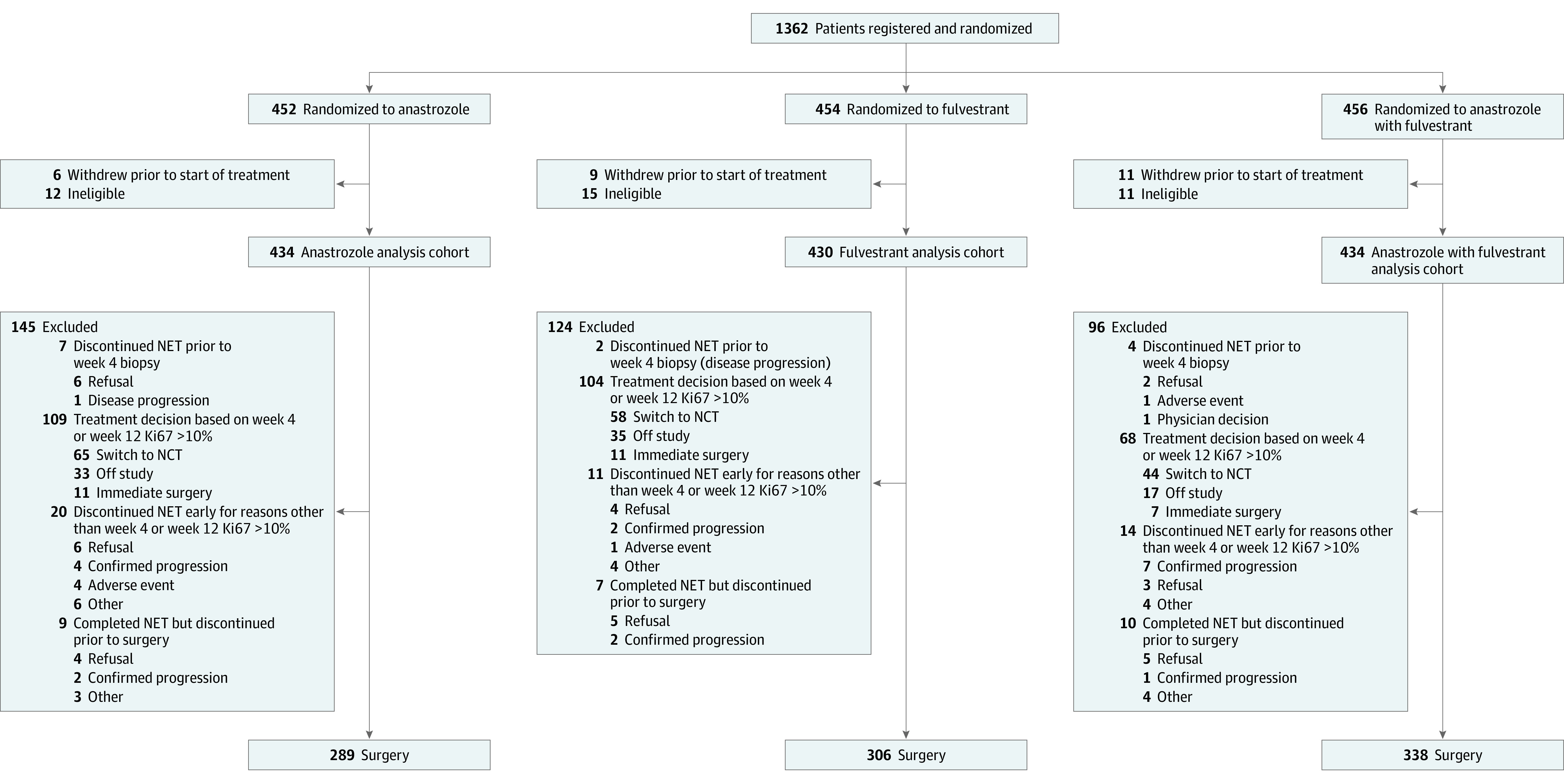
CONSORT Diagram NCT indicates neoadjuvant chemotherapy; NET, neoadjuvant endocrine therapy.

The trial was designed to ascertain whether fulvestrant alone or A+F increases the ESDR by 10% or greater over anastrozole alone. The sample size was determined under the assumption that ESDR with anastrozole alone would be similar to that in the ACOSOG Z1031B trial, namely 30%.^21^ With a sample size of 425 per arm, a 1-sided α of 0.025 χ^2^ test of 2 independent proportions would have 84% power to detect an increase of 10% or greater in ESDR for a given fulvestrant-containing arm relative to that in the anastrozole arm. A Wald test, which is asymptotically equivalent to this χ^2^ test, was used for assessing the primary aim. No adjustments for randomization stratification factors were made.

Wilcoxon rank sum tests were used to assess whether the percentage change in Ki67 after 4 weeks of NET differed between treatments and by PAM50 subtype. Conditional logistic regression modeling with the Score method of model selection was used with pretreatment Ki67 as the stratification factor to assess whether the likelihood of week 4 Ki67 greater than 10% differed by arm and then with treatment arm as the stratification factor to assess whether age, body mass index (calculated as weight in kilograms divided by height in meters squared), Eastern Cooperative Oncology Group performance status, cT stage, cN stage, grade, or pretreatment Ki67 were associated with likelihood of not achieving ESD. Testing was performed such that a 2-sided *P* value less than .05 was considered significant. The preplanned end point for the cohort of women who switched to NCT due to week 4 or week 12 Ki67 greater than 10% was pCR/RCB class I (RCB-I) rate, defined as the proportion with pCR or RCB-I. Analyses were performed using SAS, version 9.4M6 (SAS Institute). All analyses were based on data frozen on March 2, 2023. Information regarding data and safety monitoring and data quality is provided in Supplement 1.

## Results

### Patient Characteristics

From February 2014 to November 2018, 1362 female patients (mean [SD] age, 65.0 [8.2] years) were registered to the neoadjuvant phase. Subsequently, 38 were found to be ineligible, and 26 withdrew consent prior to starting protocol treatment. The remaining 1298 (anastrozole: n = 434; fulvestrant: n = 430; A+F: n = 434) women composed the analysis cohort (Figure). Baseline characteristics were similar across the arms (Table 1). Most patients were White, more than 40% had node-positive disease, and approximately 30% had locally advanced T3-4c disease. At baseline, 10% to 15% of patients had pretreatment Ki67 10% or less, and few (approximately 2% across all arms) had pretreatment Ki67 2.7% or less (Table 1).

**Table 1.** Baseline Patient and Tumor Characteristics

Characteristic	No. (%)		
	Anastrozole (n = 434)	Fulvestrant (n = 430)	Anastrozole + fulvestrant (n = 434)
Age at registration, y			
45-59	129 (29.7)	116 (27.0)	147 (33.9)
60-69	190 (43.8)	198 (46.0)	169 (38.9)
70-95	115 (26.5)	116 (27.0)	118 (27.2)
Race and ethnicity			
American Indian/Alaska Native	6 (1.4)	0	2 (0.5)
Asian	8 (1.8)	8 (1.9)	12 (2.8)
Black/African American	37 (8.5)	40 (9.3)	26 (6.0)
Hispanic or Latino	55 (12.7)	54 (12.6)	63 (14.5)
White	358 (82.5)	359 (83.5)	368 (84.8)
Not reported	25 (5.8)	23 (5.3)	26 (6.0)
ECOG PS			
0	342 (78.8)	346 (80.5)	349 (80.4)
1-2	92 (21.2)	84 (19.5)	85 (19.6)
BMI category			
Underweight/normal weight	83 (19.1)	89 (20.7)	113 (26.0)
Overweight	130 (30.0)	116 (27.0)	118 (27.2)
Obese	221 (50.9)	224 (52.1)	203 (46.8)
Not reported	0	1 (0.2)	0
History			
Arthritis	125 (28.8)	110 (25.6)	90 (20.7)
Cardiac disease	222 (51.2)	200 (46.5)	203 (46.8)
Diabetes	89 (20.5)	91 (21.2)	83 (19.1)
Osteopenia/osteoporosis	68 (15.7)	67 (15.6)	66 (15.2)
Clinical T stage			
T2	314 (72.4)	312 (72.6)	317 (73.0)
T3-4c	120 (27.6)	118 (27.4)	117 (27.0)
Clinical N stage			
N0	246 (56.7)	253 (58.8)	254 (58.5)
N1-3	188 (43.3)	177 (41.2)	180 (41.5)
ER/PgR status			
Pos/Pos	381 (87.8)	386 (89.7)	387 (89.2)
Pos/Neg	53 (12.2)	44 (10.2)	47 (10.8)
Histologic grade			
G1	94 (21.7)	82 (19.1)	109 (25.1)
G2	255 (58.8)	265 (61.6)	255 (58.8)
G3	80 (18.4)	80 (18.6)	65 (15.0)
Not reported	5 (1.2)	3 (0.7)	5 (1.2)
Central laboratory baseline Ki67 results, %			
0-2.7	7 (1.6)	9 (2.1)	10 (2.3)
2.8-10.0	46 (10.6)	55 (12.8)	53 (12.2)
10.1-20.0	102 (23.5)	86 (20.0)	103 (23.7)
20.1-30.0	87 (20.0)	90 (20.9)	80 (18.4)
30.1-40.0	63 (14.5)	61 (14.2)	58 (13.4)
40.1-50.0	35 (8.1)	40 (9.3)	38 (8.8)
50.1-100.0	74 (17.1)	63 (14.7)	69 (15.9)
No/insufficient tumor cells	20 (4.6)	26 (6.0)	23 (5.3)

Abbreviations: BMI, body mass index; ECOG PS, Eastern Cooperative Oncology Group performance status; ER, estrogen receptor; neg, negative; PgR, progesterone receptor; pos, positive.

### Treatment Course

Of the 1298 eligible patients who began NET, 933 (71.9%) (anastrozole: n = 289; fulvestrant: n = 306; A+F: n = 338) had week 4 or week 12 Ki67 10% or less or insufficient tumor cells to ascertain week 4 or week 12 Ki67 and completed 6 cycles of NET and surgery (Figure). A total of 302 patients (23.2%) discontinued NET by study design due to week 4 or week 12 Ki67 greater than 10% (anastrozole: n = 109; fulvestrant: n = 104; A+F: n = 68) or PD by imaging (anastrozole: n = 7; fulvestrant: n = 6; A+F: n = 8). Another 63 (4.9%) discontinued during NET or prior to surgery due to patient choice (anastrozole: n = 16; fulvestrant: n = 9; A+F: n = 10), adverse event (anastrozole: n = 4; fulvestrant: n = 1; A+F: n = 1), or other reasons (anastrozole: n = 9; fulvestrant: n = 4; A+F: n = 9) (Figure). Each NET regimen was well tolerated, with similar adverse event profiles (eTable 1 in Supplement 2). The grade 3 to 4 adverse event rate ranged from 4% to 6% across the 3 NET arms.

Of the 281 patients found to have a week 4 or week 12 Ki67 greater than 10%, 114 (40.6%) chose to proceed to surgery or went off study (Figure; eTable 2 in Supplement 2). The remaining 167 (59.4%) switched to NCT, most commonly, doxorubicin/cyclophosphamide followed by paclitaxel (60 [35.9%]); paclitaxel alone (56 [33.5%]); and docetaxel/cyclophosphamide (33 [19.8%]). Fourteen patients discontinued NCT early or went off study prior to surgery due to adverse events (n = 4), PD (n = 3), patient choice (n = 3), second primary cancer diagnosis (n = 1), death (n = 1), and other reasons (n = 2).

### Surgery Outcomes

A total of 933 patients who had week 4 or week 12 Ki67 10% or less or indeterminant week 4 or week 12 Ki67 completed 6 cycles of NET (Table 2). Approximately 70% had breast-conserving surgery, and more than 60% had sentinel lymphadenectomy without dissection, similar across arms. Macrometastasis in lymph nodes was found in 474 patients (50.8%), rendering mPEPI greater than 0. Only 36 of 365 patients (9.9%) with clinically node-positive disease were downstaged to pathologically node-negative or microinvasive disease (pN0/N1mic) (anastrozole: 10 of 115; fulvestrant: 11 of 115; A+F: 15 of 135). Among the 918 patients with residual invasive BC, high ER levels (Allred score 6-8) were maintained at surgery after 6 months of NET in 97.2% (95% CI, 94.5%-98.2%) receiving anastrozole, 75.7% (95% CI, 70.4%-80.4%) receiving fulvestrant, and 71.3% (95% CI, 66.2%-76.1%) receiving A+F, an expected difference given the ER downregulation effect of fulvestrant. Post-NET Ki67 2.7% or less was found in 63.3% overall, 57.8% receiving anastrozole, 61.4% receiving fulvestrant, and 62.4% receiving A+F. Table 2 also displays surgical procedures and pathologic findings of the 153 women who had a week 4 or week 12 Ki67 greater than 10% and switched to NCT. Approximately 60% had breast-conserving surgery, and 56% had sentinel lymphadenectomy alone. Invasive disease in the breast was found in 143 patients (93.5%) and lymph node macrometastasis in 87 patients (56.9%); only 5.2% had pCR.

**Table 2.** Surgical Outcomes

Outcome	No. (%)			
	Patients with a week 4 Ki67 ≤10% or indeterminate who continued receiving NET			Patients with a week 4 Ki67 >10% and switched to NCT
	Anastrozole (n = 289)	Fulvestrant (n = 306)	Anastrozole + fulvestrant (n = 338)	NCT (n = 153)
Extent of breast surgery				
Lumpectomy	201 (69.6)	212 (69.3)	239 (70.7)	91 (59.5)
Mastectomy	88 (30.5)	94 (30.7)	99 (29.3)	62 (40.5)
Extent of nodal surgery				
None	2 (0.7)	2 (0.7)	3 (0.9)	1 (0.7)
SLN surgery	175 (60.6)	199 (65.0)	220 (65.1)	86 (56.2)
ALND	52 (18.0)	39 (12.7)	45 (13.3)	32 (20.9)
SLN surgery + ALND	60 (20.8)	66 (21.6)	70 (20.7)	34 (22.2)
pT stage				
Tis/T0	6 (2.1)	6 (2.0)	3 (0.9)	10 (6.5)
T1-T2	232 (80.3)	253 (82.7)	290 (85.8)	122 (79.7)
T3-T4	51 (17.6)	47 (15.4)	45 (13.3)	21 (13.7)
pN stage				
pNX	3 (1.0)	2 (0.7)	5 (1.5)	1 (0.7)
pN0/N1mi	133 (46.0)	153 (50.0)	163 (48.2)	65 (42.5)
pN1-3	153 (52.9)	151 (49.3)	170 (50.3)	87 (56.9)
Residual invasive disease Ki67 from central laboratory, %				
0-2.7	167 (57.8)	188 (61.4)	211 (62.4)	NA
2.8-10.0	65 (22.5)	70 (22.9)	70 (20.7)	
10.1-20.0	21 (7.3)	24 (7.8)	21 (6.2)	
20.1-100	22 (7.6)	13 (4.2)	11 (3.3)	
Not obtained; pCR/pTis/pT0	6 (2.1)	6 (2.0)	3 (0.9)	
Not obtained; insufficient cells	8 (2.8)	5 (1.6)	22 (6.5)	
Residual invasive disease ER Allred score from central laboratory				
0-2	2 (0.7)	8 (2.6)	16 (4.7)	NA
3-5	4 (1.4)	61 (19.9)	68 (20.1)	
6-8	275 (95.6)	227 (74.2)	239 (70.7)	
Not obtained; pCR/pTis/pT0	6 (2.1)	6 (2.0)	3 (0.9)	
Not obtained; insufficient cells	2 (0.7)	4 (1.3)	12 (3.6)	
mPEPI score				
pCR/0	81 (28.0)	98 (32.0)	89 (26.3)	NA
1-2	35 (12.1)	43 (14.1)	48 (14.2)	
3-5	125 (43.3)	121 (39.5)	150 (44.4)	
6-8	36 (12.5)	37 (12.1)	24 (7.1)	
Nonzero	8 (2.8)	2 (0.7)	15 (4.4)	
Indeterminate	4 (1.4)	5 (1.6)	12 (3.6)	
pCR/RCB class from local laboratory				
pCR	NA	NA	NA	8 (5.2)
I				17 (11.1)
II				79 (51.6)
III				42 (27.5)
Not obtained				7 (4.6)

Abbreviations: ALND, axillary lymph node dissection; ER, estrogen receptor; mPEPI, modified preoperative endocrine prognostic index; NA, not applicable; NCT, neoadjuvant chemotherapy; NET, neoadjuvant endocrine therapy; pCR, pathologic complete response; RCB, residual cancer burden; SLN, sentinel lymph node.

### Primary End Point: ESDR

Table 3 details the determination of ESD status by treatment arm. The pCR rate was 0.5% to 1.2%, and the mPEPI 0 rate was 17.5% to 21.9% across the treatment arms. The ESDR was 18.7% with anastrozole, 22.8% with fulvestrant, and 20.5% with A+F (Table 3). The difference in ESDR was 4.1% (1-sided 97.5% upper confidence bound [UCB], 9.1%) between the fulvestrant and anastrozole arms, and 1.8% (1-sided 97.5% UCB, 7.1%) between the A+F and anastrozole arms. The control-arm ESDR was substantially lower than seen in the Z1031B trial (30%).^21^ Neither fulvestrant-containing regimen improved ESDR by 10% or greater over anastrozole (Wald test: *P* = .98 and *P* > .99, respectively; supporting data in Table 3). Multivariate conditional logistic regression modeling found that the likelihood of not achieving ESD was increased in the presence of cT3-4 (adjusted odds ratio [aOR], 2.48; 95% CI, 1.66-3.72; *P* < .001), cN1-3 (aOR, 17.85; 95% CI, 10.05-31.71; *P* < .001), grade 3 (aOR, 1.71; 95% CI, 1.04-2.80; *P* = .03), or pretreatment Ki67 greater than 20% (aOR, 1.52; 95% CI, 1.10-2.09; *P* = .01).

**Table 3.** Neoadjuvant Endocrine Therapy (NET) Outcomes and Endocrine-Sensitive Disease (ESD) Rate (ESDR) by Treatment Arm

ESD	NET outcome	No. (%)			
		Anastrozole (n = 434)	Fulvestrant (n = 430)	Anastrozole + fulvestrant (n = 434)	Total (N = 1298)
Yes	mPEPI = 0	76 (17.5)	94 (21.9)	87 (20.0)	257 (19.8)
	pCR	5 (1.2)	4 (0.9)	2 (0.5)	11 (0.8)
No	Ki67 > 10% at week 4 or 12	109 (25.1)	104 (24.2)	68 (15.7)	281 (21.6)
	Confirmed progression during or at completion of NET	7 (1.6)	6 (1.4)	8 (1.6)	21 (1.6)
	NET or surgery not completed on study	29 (6.7)	14 (3.3)	20 (4.6)	63 (4.9)
	mPEPI unable to be determined^a^	4 (0.9)	2 (0.5)	12 (2.8)	18 (1.4)
	mPEPI > 0	204 (47.0)	206 (47.9)	237 (54.5)	647 (49.8)
ESDR, % (95% CI)		18.7 (15.1-22.7)	22.8 (18.9-27.1)	20.5 (16.8-24.6)	20.7 (18.5-23.0)
Difference in ESDR compared to anastrozole (1-sided 97.5% upper confidence bound), %		NA	4.1 (9.5)	1.8 (7.1)	NA
Wald test *P* value^b^		NA	.98	>.99	NA

Abbreviations: mPEPI, modified preoperative endocrine prognostic index; NA, not applicable; pCR, pathologic complete response.

^a^ Unable to determine whether mPEPI was 0 or nonzero due to missing information on pT stage, pN stage, or Ki67.

^b^ Testing H0: difference in ESDR of 10% or less against Ha: difference in ESDR greater than 10%.

### Secondary End Point for NCT Cohort

Among the 167 women who switched to NCT due to week 4 or week 12 Ki67 greater than 10%, there were 8 patients with pCR (4.8%; 95% CI, 2.1%-9.2%) and 17 patients with RCB-I (10.2%; 95% CI, 6.0%-15.8%). This yielded a pCR/RCB-I rate of 15.0% (25 of 167; 95% CI, 9.9%-21.3%).

### Ki67 Suppression at Week 4

There were 1122 patients (86.4%) who had central determination of both pretreatment Ki67 and week 4 Ki67. eFigure 2 in Supplement 2 depicts the scatterplot of paired pretreatment Ki67 and week 4 Ki67 levels by treatment arm. The median (IQR) percentage change in Ki67 at week 4 was −80.6% (−92.2% to −55.6%) with anastrozole, not significantly different than that with fulvestrant (−79.1% [−90.8% to −55.5%]; *P* = .36) or A+F (−83.5% [−92.3% to −62.4%]; *P* = .15) (Table 4). Ki67 suppression to 10% or less at week 4 was also examined. Of the 155 patients who had a pretreatment Ki67 10% or less, only 6 (3.9%) had a week 4 Ki67 greater than 10% (ranging 12%-18%). Of the 967 patients with pretreatment Ki67 greater than 10%, 103 of 336 patients receiving anastrozole (30.7%), 94 of 315 patients receiving fulvestrant (29.8%), and 60 of 316 patients (19.0%) receiving A+F had week 4 Ki67 greater than 10%; the remainder of evaluable patients had suppression to 10% or less. The likelihood of week 4 Ki67 greater than 10%, adjusting for pretreatment Ki67 levels, was significantly lower with A+F than anastrozole (*P* < .001) but not significantly different between fulvestrant and anastrozole (*P* = .91) (supporting data in Table 4).

**Table 4.** Ki67 Changes After 4 Weeks of Neoadjuvant Endocrine Therapy

Patient group	Anastrozole	Fulvestrant	Anastrozole + fulvestrant	Difference in median percent change in Ki67 at week 4 from pretreatment Ki67 between 2 given arms, % (95% CI)^a^	Wilcoxon rank sum *P* value
**% Change in Ki67 at week 4 from pretreatment Ki67 levels**					
All patients with pretreatment and week 4 Ki67 results					
No.	379	372	371	F vs A: 1.19 (−1.33 to 3.71) A+F vs A: −1.70 (−3.97 to 0.58)	F vs A: .36 A+F vs A: .15
Median (IQR), %	−80.6 (−92.2 to −55.6)	−79.1 (−90.8 to −55.5)	−83.5 (−92.3 to −62.4)		
Luminal A					
No.	127	113	115	F vs A: 2.7 (−1.4 to 7.2) A+F vs A: 0.1 (−3.5 to 4.2)	F vs A: .21 A+F vs A: .94
Median (IQR), %	−84.8 (−92.2 to −58.8)	−79.1 (−90.9 to −55.1)	−81.5 (−91.7 to −64.8)		
Luminal B					
No.	104	86	97	F vs A: −3.2 (−9.0 to 2.2) A+F vs A: −10.6 (−16.0 to −5.7)	F vs A: .25 A+F vs A: <.001
Median (IQR), %	−76.7 (−89.0 to −55.6)	−80.2 (−92.1 to −60.5)	−90.4 (−95.2 to −81.9)		
**Patient group**	**Anastrozole**	**Fulvestrant**	**Anastrozole + fulvestrant**	**Likelihood of week 4 Ki67 >10% adjusted for pretreatment Ki67 levels^b^**	**Wald test *P* value**
**Week 4 Ki67 >10% rate among those with pretreatment Ki67 >10%^c^**					
Patients with a pretreatment Ki67 >10% and a week 4 Ki67 result, % (No./total No.)	30.7 (103/336)	29.8 (94/315)	19.0 (60/316)	aOR (F/A) = 0.98 (0.68-1.41) aOR (A+F/A) = 0.49 (0.33-0.73)	F/A: .91 A+F/A: <.001
Luminal A, % (No./total No.)	13.5 (15/111)	23.7 (22/93)	9.0 (8/89)	aOR (F/A) = 1.85 (0.88-3.88) aOR (A+F/A) = 0.64 (0.25-1.63)	F/A: .11 A+F/A: .35
Luminal B, % (No./total No.)	43.7 (45/103)	30.6 (26/85)	19.6 (19/97)	aOR (F/A) = 0.52 (0.27-1.00) aOR (A+F/A) = 0.27 (0.13-0.53)	F/A: .05 A+F/A: <.001

Abbreviations: A, anastrozole; aOR, adjusted odds ratio; F, fulvestrant.

^a^ Hodges-Lehmann estimate of the difference in 2 medians with corresponding distribution-free confidence interval (Moses) based on the Wilcoxon rank sum test.

^b^ Stratification factor pretreatment Ki67 categorized as 10.1% to 20%, 20.1% to 30%, 30.1% to 40%, 40.1% to 50%, and 50.1% to 100%.

^c^ Exclusions due to pretreatment Ki67 not obtained or 0% to 10% or week 4 Ki67 not obtained: A, 98; F, 115; and A+F, 118.

### Ki67 Suppression at Week 4 in the PAM50 Cohort

Preplanned correlative studies included an examination of the association between gene expression profiles, such as PAM50 subtype, and NET sensitivity. A determination of PAM50 subtype was possible for 753 patient samples (58%) (anastrozole: n = 265; fulvestrant: n = 234; and A+F: n = 254) (eFigure 3 in Supplement 2). The rest were not possible due to withdrawal, ineligibility, lack of an adequate sample, less than 50% tumor cellularity, or insufficient RNA (eFigure 3 in Supplement 2). The PAM50 cohort had more histologic grade 3 tumors, cN+ tumors, and pretreatment Ki67 greater than 20% than the rest of the trial population (eTable 3 in Supplement 2). Baseline patient and tumor characteristics are shown in eTable 4 in Supplement 2. There were 394 (52.3%) luminal A (LumA), 304 (40.3%) LumB, 45 (6.0%) ERBB2-enriched, and 10 basal-like tumors (1.3%). Patients with ERBB2-enriched or basal-like tumors fared poorly on NET: 36 (65.5%) discontinued NET due to week 4 or week 12 Ki67 greater than 10%, 2 (3.6%) experienced progression while receiving NET, 13 (23.6%) had a mPEPI greater than 0, and 1 (1.8%) had an mPEPI 0; in 3 patients (8.6%), mPEPI could not be determined.

eFigure 2 in Supplement 2 depicts the scatterplot of paired pretreatment Ki67 and week 4 Ki67 levels by treatment arm among the LumA and LumB tumors. The percentage change in Ki67 and the likelihood of maintaining Ki67 greater than 10% after 4 weeks of NET were similar between fulvestrant-containing regimens and anastrozole in LumA (Table 4). In contrast, in LumB tumors, A+F led to significantly greater percentage change in week 4 Ki67 from pretreatment Ki67 (A+F vs anastrozole: −10.6%; 95% CI, −16.0% to −5.7%; *P* < .001) and lower likelihood of week 4 Ki67 greater than 10% (aOR, A+F/A = 0.27; 95% CI, 0.13-0.53; *P* < .001) compared to anastrozole alone (Table 4). The rates of mPEPI 0, 1 to 3, or greater than 3 by treatment arm in the overall, LumA, and LumB populations are shown in eTable 5 in Supplement 2.

## Discussion

In this phase 3 randomized clinical trial, the 6-month ESDR following anastrozole, fulvestrant, or A+F was 19% to 23% in postmenopausal women with ER-rich/ERBB2-negative BC. Neither fulvestrant nor A+F significantly increased the ESDR over anastrozole. There was a low rate of pCR (0.8%) and axillary lymph node clearance (10%) across the arms, which was consistent with previous reports.^19,21,23^ This study demonstrated that nodal positivity, locally advanced tumor size, high grade, or pretreatment Ki67 greater than 20% significantly increased the likelihood of not achieving ESD. A higher proportion of patients had node-positive and locally advanced disease in ALTERNATE than in the Z1031B trial, which may have contributed to the lower than expected ESDR.^19^ However, PD on NET was rare in this trial, approximately 1% overall. This may be a consequence of the early triaging of patients with on-treatment Ki67 greater than 10% off NET.^24^

In contrast to limited pathologic downstaging, complete cell cycle arrest (Ki67 ≤ 2.7%) at surgery was observed in 43.1% of the 1272 patients with baseline Ki67 greater than 2.7%, indicating the potent antiproliferative effects of NET. It is noted that the likelihood of week 4 or week 12 Ki67 greater than 10% was lower on A+F than anastrozole, leading to fewer patients receiving A+F going off NET at week 4 than those receiving anastrozole. However, this did not translate to an improved ESDR or significantly greater Ki67 suppression at week 4 between A+F and anastrozole because of the limited effect on the other, histopathological, PEPI components.

As a SERD, the antitumor activity of fulvestrant has been shown to be dependent on its ability to degrade ER.^25^ The observation that ER remained high (Allred score 6-8) in more than 70% of patients after 6 months of fulvestrant or A+F in this trial indicates that fulvestrant is a relatively ineffective ER degrader. While dose-dependent ER degradation was observed for fulvestrant in the NEWEST trial,^26^ the ability to dose escalate fulvestrant is limited by pharmacokinetic properties. Our data therefore support the development of more effective SERDs or other novel ER-targeting mechanisms.^27^ The recent approval of elacestrant for *ESR1*-mutated metastatic BC represents an example; however, *ESR1* mutations are rarely present at diagnosis.^28^

The 4.8% pCR rate after switching to NCT due to week 4 or week 12 Ki67 greater than 10% is consistent with observations in the Z1031B trial.^19^ The much larger sample size of ALTERNATE provides further support for the relative ineffectiveness of chemotherapy for ET-resistant tumors. The RCB-I rate was also low. Since post-NCT RCB index is prognostic in patients with BC, including those with HR-positive/ERBB2-negative disease,^22,29^ our data underscore the need for more effective systemic treatments for these patients.

PAM50 subtype, which is the basis for the commercial assay Prosigna, is prognostic in ER-positive BC,^30,31^ and emerging evidence indicates an association with ET sensitivity.^21^ In ALTERNATE, A+F led to a significantly greater Ki67 suppression and decreased likelihood of maintaining Ki67 greater than 10% at week 4 in LumB vs LumA tumors. This finding is hypothesis generating, but suggests that despite relative AI resistance, LumB tumors, which comprise 40% of ER-rich BCs, are frequently ER dependent. Thus, improvements in ET may particularly benefit this tumor subset. Notably, nonluminal BC was found in 7.3% of patients in the PAM50 cohort despite high ER levels required for eligibility. Their markedly reduced sensitivity to NET is consistent with earlier findings in the Z1031 trial,^19^ thus calling into question the appropriateness of NET for nonluminal ER-positive BCs.

### Strengths and Limitations

To our knowledge, ALTERNATE is the first reported clinical trial comparing fulvestrant or A+F with anastrozole in postmenopausal women with early-stage ER-rich/ERBB2-negative BC. Strengths include the randomized phase 3 trial design, central Ki67 analysis, early triaging to chemotherapy/surgery if week 4 or week 12 Ki67 greater than 10%, and more than 750 tumors analyzed by PAM50. There are several limitations. First, the importance of achieving ESD (mPEPI 0) depends on whether it is associated with a low risk of disease recurrence. This question is being examined in the adjuvant portion of the ALTERNATE trial. However, further follow-up is required to address the relapse-free survival end point. Second, radiographic response rates were not analyzed since pretreatment and posttreatment imaging was completed in less than 60% of patients. Lastly, baseline clinical characteristics of the PAM50 cohort, representing 58% of the trial population, were similar between the treatment arms, but had disease more locally advanced, higher in grade, and higher in pretreatment Ki67 than those without PAM50 determination.

## Conclusions

In this randomized clinical trial, the neoadjuvant phase of the ALTERNATE trial did not demonstrate superiority for fulvestrant or A+F over anastrozole alone in improving ESDR. Pathologic eradication of disease was uncommon across the arms, largely limiting mPEPI 0 status following NET to those with initial node-negative disease at presentation. The limited ER degradation following fulvestrant or A+F supports the ongoing development of more effective SERDs. ALTERNATE also confirmed the poor pathologic response to NCT among ER-rich/ERBB2-negative BC resistant to NET. The higher antiproliferative effect of combining AI with a SERD in the LumB population is provocative and warrants further study, particularly in light of the development of oral SERDs.^26^

## Notes

Supplement 1.
Trial Protocol

Supplement 2.
eAppendix.
**eTable 1.** Common Adverse Events possibly, probably, or definitely related to NET **eTable 2.** Characteristics of Patients with Ki67_wk4||wk12_>10% **eTable 3.** Characteristics of Patients with PAM50 Determination vs. Patients without PAM50 determination **eTable 4.** Patient and Tumor Characteristics for the PAM50 Subtype Analysis Cohort by Treatment Arm **eTable 5.** NET Outcomes by Treatment Arm in All Patients and by LumA and LumB Subtype **eFigure 1.** The ALTERNATE Trial Study Schema **eFigure 2.** Scatterplot of Paired Ki67_pre_ and Ki67_wk4_ Levels **eFigure 3.** REMARK Diagram

Supplement 3.
Data Sharing STATEMENT
