# Supplementary material for: Endocrine-Sensitive Disease Rate in Postmenopausal Patients With Estrogen Receptor–Rich/ERBB2-Negative Breast Cancer Receiving Neoadjuvant Anastrozole, Fulvestrant, or Their Combination: A Phase 3 Randomized Clinical Trial
Source: JAMA Oncol. 2024 Jan 18;10(3):362–71. doi: 10.1001/jamaoncol.2023.6038 (PMC10797521; doi:10.1001/jamaoncol.2023.6038)
Supplement: Supplement 1. — Trial Protocol [file jamaoncol-e236038-s001.pdf]

Alliance for Clinical Trials in Oncology
Alliance A011106

ALTernate approaches for clinical stage II or III Estrogen Receptor positive breast cancer NeoAdjuvant TrEatment (ALTERNATE) in postmenopausal women: A Phase III Study

Fulvestrant (NSC #719276, IND Exempt) supplied by AstraZeneca, distributed by McKesson
Commercial Agents: Anastrozole (NSC #719344) Paclitaxel (NSC #673089)

Clinical Trials.gov Identifier: NCT01953588

Study Chair
Cynthia X. Ma, MD, PhD
Washington University School of Medicine
660 S. Euclid Ave, Campus Box 8056
St. Louis, MO 63110
Tel: 314-362-9383 Fax: 314-367-7086
cynthiaxma@wustl.edu

Community Oncology Co-Chair
Gary W. Unzeitig, MD, FACS
Laredo Breast Care
Tel: 956-726-3691 Fax: 956-726-3692
drgwu@laredobreastcare.com

Surgery Co-Chair
A. Marilyn Leitch, MD, FACS
UT Southwestern Medical Center
Tel: 214-648-5890
marilyn.leitch@utsouthwestern.edu

Pathology Co-Chair
Souzan Sanati, MD
Cedars-Sinai Medical Center
Tel: 310-423-3277
Souzan.Sanati@cshs.org

Correlative Science Co-Chairs
Matthew J. Ellis, MB, BChir, PhD
Baylor College of Medicine
Tel: 713-798-1845
Matthew.Ellis@bcm.edu

Lisa Carey, MD
University of North Carolina
Tel: 919-966-4431
Lisa_carey@med.unc.edu

Pathology Data Coordinator
Shana Thomas, MS, CCRP
Washington University School of Medicine
Tel: 636-209-2203
shana.thomas@wustl.edu

Breast Committee Co-Chairs
Lisa A Carey, MD
UNC Lineberger Comprehensive Cancer Center
Tel: 919-966-4431
Lisa_carey@med.unc.edu

Ann H. Partridge, MD
Dana-Farber/Partners
Tel:617-632-3800
ahpartridge@partners.org

Legacy ACOSOG Breast Committee Chair
Kelly Hunt, MD
MD Anderson
Tel: 713-792-7216
khunt@mdanderson.org

Primary Statistician
Vera Suman, PhD
Mayo Clinic
Tel: 507-284-8803
suman@mayo.edu

Secondary Statistician
Travis Dockter, MS
Mayo Clinic
Tel: 507-266-9803
dockter.travis@mayo.edu

Protocol Coordinator
Laura Hoffman
Tel: 773-834-2546
lhoffman22@bsd.uchicago.edu

Data Manager
Adam Eggert
Tel: 507-538-1760
eggert.adam@mayo.edu

Participating NCTN groups: **Alliance**/Alliance for Clinical Trials in Oncology, **ECOG-ACRIN**/ECOG-ACRIN Cancer Research Group, **NRG**/NRG Oncology, **SWOG**/SWOG

**Alliance Central Protocol Operations Office**	**Alliance Statistics and Data Center**
125 S. Wacker Drive, Suite1600<br>Chicago, IL 60606<br>Tel: 773-702-9171 Fax: 312-345-0117<br>*www.allianceforclinicaltrialsinoncology.org*	Mayo Clinic<br>200 First St. SW<br>Rochester, MN 55905
**Expedited Adverse Event Reporting**<br>*http://eapps-ctep.nci.nih.gov/ctepaers/*	**Medidata Rave® iMedidata portal**<br>*https://login.imedidata.com*
**OPEN (Oncology Patient Enrollment Network)***https://open.ctsu.org*	**Biospecimen Management System**<br>*http://bioms.allianceforclinicaltrialsinoncology.org*

Protocol Resources

**A011106 Nursing Contact**	**A011106 Pharmacy Contact**
Nicole Moxon, RN, BSN, ONC<br>Providence Breast Cancer Clinical Trials<br>Tel: 503-215-2619<br>*Nicole.moxon@providence.org*	Myounghee Lee, Pharm D, PhD, CCRP<br>University of Maryland Medical Center<br>Tel: 410-328-5344<br>*mlee1@umm.edu*
**Alliance Biorepository at Washington University (WUSTL)**<br>Mark Watson, MD, PhD<br><br>Washington University<br>Tel: 314-454-7615<br>Fax: 314-454-5525<br>*tbank@wudosis.wustl.edu*	**A011106 Specimen Coordinator**<br><br>Cynthia Zhang<br>Washington University<br>Tel: 314-747-4793<br>Fax: 314-363-0206<br>*cynthiazhang@wustl.edu*

Protocol-related questions may be directed as follows:

**Questions**	**Contact**
Questions regarding patient eligibility, treatment, and dose modification:	Study Chair, Committee Chair, Nursing Liaison, Protocol Coordinator, or Data Manager
Questions related to data submission, RAVE or patient follow-up:	Data Manager
Questions regarding the protocol document:	Protocol Coordinator
Questions related to IRB review and model consent revisions	Alliance Regulatory Inbox<br>Email: *regulatory@alliancenctn.org*
Questions related to CTEP-AERS reporting:	Alliance Pharmacovigilance Inbox<br>pharmacovigilance@alliancenctn.org
Questions related to biospecimen registration in BioMS	1-855-552-4667
Questions related to biospecimen requirements and processing	Specimen Coordinator, Study Chair, Correlative Science Co-chair
Questions related to biopsy kit ordering	1-855-552-4667
Questions related to tracking of specimens for Ki67 biomarker analysis:	A011106 Specimen Coordinator
Questions related to interpretation of Ki67 results, calculation of modified PEPI score and Residual Cancer Burden:	Study Chair, Pathology Co-chair, Correlative Science Co-chair

CONTACT INFORMATION		
**For regulatory requirements:**	**For patient enrollments:**	**For data submission:**
Regulatory documentation must be submitted to the CTSU via the Regulatory Submission Portal.<br>(Sign in at www.ctsu.org, and select the Regulatory > Regulatory Submission.)<br><br>Institutions with patients waiting that are unable to use the Portal should alert the CTSU Regulatory Office immediately at 1-866-651-2878 to receive further instruction and support.<br><br>Contact the CTSU Regulatory Help Desk at 1-866-651-2878 for regulatory assistance.	Refer to the patient enrollment section of the protocol for instructions on using the Oncology Patient Enrollment Network (OPEN). OPEN is accessed at https://www.ctsu.org/OPEN_SYSTEM or https://OPEN.ctsu.org.<br><br>Contact the CTSU Help Desk with any OPEN related questions by phone or email : 1-888-823-5923, or ctsucontact@westat.com.	Data collection for this study will be done exclusively through Medidata Rave. Refer to the data submission section of the protocol for further instructions.
The most current version of **the study protocol and all supporting documents** must be downloaded from the protocol-specific page located on the CTSU members' website (https://www.ctsu.org). Access to the CTSU members' website is managed through the Cancer Therapy and Evaluation Program - Identity and Access Management (CTEP-IAM) registration system and requires log in with a CTEP-IAM username and password		
**For clinical questions (i.e. patient eligibility or treatment-related)** see the Protocol Contacts, Page 2.		
**For non-clinical questions (i.e. unrelated to patient eligibility, treatment, or clinical data submission)**<br>Contact the CTSU Help Desk by phone or e-mail:<br>CTSU General Information Line – 1-888-823-5923, or ctsucontact@westat.com. All calls and correspondence will be triaged to the appropriate CTSU representative.		

Table of Contents

1.0 Introduction and Background 7
1.1 Overview 7
1.2 Background 8
1.3 Rationale for trial design 15
1.4 Significance 16
1.5 Rationale for the change in study design (protocol Update #05) 16

2.0 Objectives 17
2.1 Primary objectives 17
2.2 Secondary objectives 17
2.3 Correlative science objectives 18

3.0 Schema 20

4.0 Patient Selection 21
4.1 On-study guidelines 21
4.2 Eligibility criteria 21
4.3 Staging criteria 23

5.0 Study Calendar 23
5.1 All patients: Prior to registration through cycle 2 (week 8) 23
5.2 Study calendar cycle 3 through surgery 24
5.3 Post-operative study calendar 27

6.0 Patient Registration and Randomization 29
6.1 Investigator and Research Associate Registration with CTEP 29
6.2 Cancer Trials Support Unit Registration Procedures 30
6.3 Stratification factors 32
6.4 Registration to correlative and companion studies 32
6.5 Treatment arms 33

7.0 Specimen Submission for Essential Integral and integrated Biomarker and Correlative Studies 35
7.1 Required pre-treatment core biopsy for biomarker and correlative studies 37
7.2 Required 4-week core biopsy for integral Ki67 assay on Arms I, II and III for treatment decision making and correlative studies 38
7.3 Required post-treatment core biopsy at time of surgery for all patients after the completion of neoadjuvant endocrine or chemotherapy for correlative studies 38
7.4 Required fixed tumor tissue slide submission for Ki67 to calculate PEPI and modified PEPI score on Arms I, II and III at time of surgery 38
7.5 Optional 12-week core biopsies for Ki67 biomarker assay on Arms I, II and III for treatment decision making and correlative studies 39
7.6 Optional cycle 1, day 2 core biopsy for patients in the Neoadjuvant Chemotherapy Group on neoadjuvant paclitaxel only for correlative studies 39
7.7 Optional core biopsy at disease progression 39
7.8 Neoadjuvant serum, plasma and anti-coagulated whole blood for DNA collection 39
7.9 Forms submission 41
7.10 Specimen registration and tracking 41
7.11 Shipment of samples 42
7.12 Funding for research biopsy procedures 43

8.0 Treatment 44
8.1 Neoadjuvant therapy: 44

8.2 Neoadjuvant treatment evaluation and decision tree 45
8.3 Dose modifications, and management of toxicity........ 48
8.4 Surgery........ 48
8.5 Postoperative chemotherapy........ 49
8.6 Postoperative endocrine therapy........ 50
8.7 Adjuvant radiation therapy 50
8.8 Study drug pharmacology........ 51
8.9 Prohibited medications 58
8.10 Concomitant medications 58
8.11 Criteria for early discontinuation of study drug........ 58

9.0 Follow-up 58
9.1 Patients who have been registered to the study and signed a consent form, but have *withdrawn consent prior to receiving any study therapy* will be considered a cancellation. On-study data, but no biospecimens or images are to be submitted. No further follow-up is required. 59
9.2 Patients who have been *found not to have fulfilled all of the eligibility requirements* after signing a consent form and starting study therapy may continue on study following pre-protocol (that is, follow the treatment and evaluation schedules laid out in the protocol), if they are deriving benefit. 59
9.3 If any one of the following scenarios is true, then the patient will enter the survival and disease status follow-up phase of the study where information regarding disease and survival status will be collected and reported by on-line data submission yearly from the time of registration until death or a maximum of 10 years post registration. 59
9.4 Research blood collection during the follow up period 59

10.0 Evaluation of Outcomes........ 60
10.1 Neoadjuvant treatment phase........ 60
10.2 Surgery........ 60
10.3 Following surgery, diagnosis of breast cancer recurrence and other cancer events 60

11.0 Adverse Event Reporting 61
11.1 Routine adverse event reporting 62
11.2 Expedited adverse event reporting........ 62
11.3 Expected adverse events 65

12.0 Data Considerations........ 66
12.1 Patient data quality control 66
12.2 Data Quality Portal 67

13.0 Statistical Considerations 67
13.1 Study design: 67
13.2 Endpoints........ 68
13.3 Stratification 68
13.4 Accrual rate........ 68
13.5 Sample size 69
13.6. Study duration........ 71
13.7 Analyses plans 71

14.0 Regulatory Considerations........ 73
14.1 Registering physician........ 73
14.2 Registering institution........ 73
14.3 Submission of IRB approval........ 73
14.4 Inclusion of women and minorities 74
14.5 Clinical site audits........ 74

14.6 Clinical monitoring 74
14.7 Data safety and monitoring 74

15.0 Correlative Research Studies (A011106-ST) 75
15.1 Real time essential biomarker studies 75
15.2 Additional biomarker studies 78
15.3 Analysis of plasma circulating tumor DNA (ctDNA) 90
15.4 Analysis of NF1 Status 97
15.5 Clinical Proteomic Tumor Analysis Consortium (CPTAC) analysis of ALTERNATE samples 102

16.0 References 108

17.0 Appendices 118
17.1 Staging reference 118
17.2 ALLRED score 119
17.3 ECOG/Zubrod performance status scale 119
17.4 SOP for Whole Exome Sequencing 120

1.0 INTRODUCTION AND BACKGROUND

1.1 Overview

Estrogen receptor positive (ER+) breast cancer in postmenopausal women is a major public health problem. In the United States (US), 1 out of 8 women will be diagnosed with breast cancer in their life time [1]. A total of 230,480 new cancer cases and 39,520 deaths are expected to be attributed to breast cancer in the year 2011 [2]. Among all breast cancer cases, over 75% occur in postmenopausal women, in whom 80% of the cases are ER+ [3]. Since the majority of breast cancer cases are diagnosed at an early stage (I – III), relapse of early stage disease accounts for the majority of breast cancer deaths [1]. Although ER+ breast cancer tends to recur later in the course of disease than ER- breast cancer, the cumulative rate of recurrence over time is similar for both disease groups [4, 5]. Therefore, recurrence of ER+ breast cancer in postmenopausal women is a major contributor of breast cancer mortality.

Adjuvant therapy following curative surgery has significantly improved breast cancer outcome. In the case of ER+ breast cancer, systemic chemotherapy followed by endocrine treatment with tamoxifen has been shown to half the breast cancer mortality rate [6]. The recent introduction of aromatase inhibitors (AIs) in early stage breast cancer has further reduced the recurrence rate, however a significant number of patients recur despite the current standard treatment. At a median follow-up of 120 months in patients enrolled in the Arimidex, Tamoxifen, Alone or in Combination (ATAC) trial, recurrence was observed in 19.7% and 24.0% of patients treated with 5 years of adjuvant anastrozole and tamoxifen, respectively, with a persistent risk of relapse over time observed in both treatment arms [7], indicating a need to improve the current standard therapy. However, the evaluation of new agents in the adjuvant setting has traditionally required large number of patients and years of follow up to demonstrate the effectiveness in reducing cancer relapse and/or mortality. The development of surrogate endpoints for disease free survival (DFS) and overall survival (OS) is needed for efficient drug screening and to expedite the drug development process.

The goal of this trial is to develop a Ki67-based biomarker strategy in the neoadjuvant setting to predict long-term outcome of patients with ER+ breast cancer. We intend to validate the achievement of the Modified Preoperative Endocrine Prognostic Index (PEPI) score of 0, post neoadjuvant endocrine therapy ([8] and Section 1.2.2 for modified PEPI definition) as a surrogate marker of success for DFS. Based on promising data in the metastatic setting, we will also compare fulvestrant alone, fulvestrant in combination with anastrozole and anastrozole alone in regards to the rate of modified PEPI 0 to provide rationale for future adjuvant studies of fulvestrant in ER+ early stage breast cancer. In this trial endocrine resistant tumors are identified early by Ki67 assessment on the 4-week (required) and then the 12-week (optional) tumor biopsies. Patients with tumor levels of Ki67 > 10% at these time points will be switched to neoadjuvant weekly paclitaxel, or other standard taxane and/or anthracycline or CMF regimens to assess the rate of complete pathologic response (pCR) to chemotherapy as a secondary endpoint. By providing validated surrogate endpoints for endocrine therapy agents and the response data (pCR rate) to standard chemotherapy for the resistant population, results from the ALTERNATE study are expected to provide the foundation for future novel therapeutics development for early stage ER+ breast cancer.

1.2 Background

1.2.1 Evidence of neoadjuvant Ki67 suppression in predicting adjuvant outcomes of endocrine agents

Neoadjuvant endocrine therapy has become a standard of care in postmenopausal women with bulky ER+ breast cancer because of improvement in the rate of breast conserving surgeries observed in previous neoadjuvant tamoxifen and aromatase inhibitor trials [9]. Importantly, the neoadjuvant setting has become an important research tool in drug development and in studying mechanisms of endocrine resistance [10].

Can short-term tumor response observed in the neoadjuvant setting predict adjuvant efficacy of endocrine therapy agents? The answer to this question has not been straightforward. Unlike neoadjuvant chemotherapy trials in ER negative breast cancer where pCR is an established endpoint, neoadjuvant endocrine therapy in ER+ breast cancer has been associated with a low pCR rate (approximately 1%), therefore alternative endpoints are needed. Clinical response, which is measured by calipers or measuring tapes was the primary endpoint in the four major randomized neoadjuvant aromatase inhibitor (AI) studies conducted in postmenopausal women with stage II-III ER+ breast cancer, including the P024 trial (Letrozole vs Tamoxifen) [11], the Immediate Preoperative Anastrozole, Tamoxifen, or Combined with Tamoxifen (IMPACT) trial [12], and the Pre-Operative "Arimidex" Compared to Tamoxifen (PROACT) trial (Table 1) [13], which compared an AI to tamoxifen, and the recently reported ACOSOG Z1031 (Letrozole vs Anastrozole vs Exemestane) that compared the three AIs [14]. Secondary endpoints of these studies have included radiologic response and breast conservation rate. These endpoints are far from perfect since 1) the measurement of clinical response is subject to variations from individual examiners; 2) radiological response may not truly reflect tumor response; and 3) surgical outcome are subject to bias and practice differences among surgeons.

Table 1 Major Randomized Neoadjuvant Aromatase Inhibitor vs Tamoxifen Trials

Overall Response												
Study	Clinical Response			Mammogram Response			Ultrasound Response			Rate of Breast Conserving Surgery		
	AI	Tam	P Value	AI	Tam	P value	AI	Tam	P value	AI	Tam	P value
P024	55%	36%	<0.001	34%	16%	<0.001	35%	25%	0.042	45%	35%	0.022
IMPACT	37%	36%	NS	ND			24%	20%	NS	44%	31%	NS
PROACT	49.7%	39.7%	NS	ND			36.2%	26.5%	NS	43%	30.8%	0.04

Because of the intrinsic flaws associated with the above endpoints, the correlations between neoadjuvant response and adjuvant outcomes have been inconsistent. In the P024 trial, letrozole treatment was associated with a significant improvement in clinical response rate, radiographic response and breast conservation rate (Table 1) [11]. However, the IMPACT and PROACT trials only demonstrated a trend favoring the anastrozole arm, which was not statistically significant (Table 1) [12, 13]. A subsequent meta-analysis was required to demonstrate that AI is more effective than tamoxifen in both clinical response and breast conservation rate [15]. In the ACOSOG Z1031 trial, there were no differences among the three AIs in surgical outcome or biological outcome (by Ki67), however exemestane was dropped out for further evaluation based on clinical response (exemestane 62.9%, letrozole 74.8%, anastrozole 69.1%) [14].

In contrast, Ki67 response, measured as a secondary endpoint in these studies, has consistently recapitulated adjuvant trial outcomes (Table 2). A greater suppression of Ki67 has been consistently observed following treatment with an AI versus tamoxifen, which parallels findings based on relapse events in adjuvant endocrine trials with matched treatment randomizations. In the P024 trial, treatment-induced reduction in geometric mean Ki67 was significantly greater with 4 months of letrozole (87%) than tamoxifen (75%; analysis of covariance P = 0.0009) [16]. The superiority of letrozole compared to tamoxifen in Ki67 suppression correlates to its superior relapse free survival (RFS) observed in the BIG-1 98 adjuvant trial [17]. In the IMPACT trial, suppression of Ki67 after 2 and 12 weeks was significantly greater with anastrozole than with tamoxifen (P = 0.004), which mirrored the result based on RFS in the ATAC trial [18]. In the ACOSOG Z1031 trial, the effects on Ki67 was similar among all three AIs (anastrozole 78%, exemestane 81.2% and letrozole 87.1%), which correlates to the similar DFS for patients treated with either anastrozole or exemestane in the adjuvant MA27 trial [19]. These data indicate that Ki67 response in the neoadjuvant setting could potentially be employed as a robust surrogate endpoint to predict adjuvant outcome [20]. Indeed, several pre-operative studies utilized Ki67 suppression as a primary endpoint [21-24].

Table 2: Neoadjuvant Biomarker Study to Predict Efficacy of Endocrine Agents in the Adjuvant Setting

**Adjuvant Trials**		**Neoadjuvant Trials**	
Study (Sample Size)	Results (Based on Events)	Study (Sample Size with available Ki67 data)	Results (Based on Ki67)
BIG 1-98 (N=8010)	L > /t	P024 (N=185)	L > T
ATAC (N=9366)	A > T or A + T	IMPACT (N=259)	A > T and also T + A
MA27 (N=7576)	A similar to E	Z1031 (N=266)	

A: Anastrozole, T: Tamoxifen, E: Exemestane, L: Letrozole, >: better

1.2.2 Evidence of Preoperative Endocrine Prognostic Index (PEPI) Score 0 or Modified PEPI 0 in predicting success in RFS

In addition to Ki67 response, in a multivariable analysis conducted on the P024 trial, 3 other post-neoadjuvant endocrine therapy tumor factors were determined to have independent prognostic value for relapse and death after relapse [8]. These included pathological tumor size (T1/2 versus T3/4), pathological node status (positive or negative), the natural logarithm of the Ki67 value and the ER status of the tumor. A prognostic score, the preoperative endocrine prognostic index (PEPI), was developed, which weighs each of these factors according to their associated hazard ratios. PEPI was then validated in an independent data set from the IMPACT trial [8]. No relapses were recorded in either trial in patients with T1, N0 tumors with a PEPI score of 0 (residual tumor with Ki67 index of 2.7% - natural logarithm of 1- or less with maintained ER expression) or in the rare patient with a pCR. PEPI has also recently been validated in the POL Trial (PreOperative Letrozole trial: A multicenter phase II trial of letrozole in postmenopausal women with clinical stage II or III hormone receptor positive breast cancer) [25]. In the combined analysis of P024 trial/POL trial, no relapse was observed with a median follow up of 61.3 months in the 24 patients (16 pT1N0, 8 pT2N0) in the PEPI 0 category (Figure 1A). The extremely low event rates for patients in the PEPI-0 category (green line) are illustrated in Figure 1.

Figure 1 RFS in patients with PEPI (A) or modified PEPI (B) by score 0 (green) vs non-0 (red)

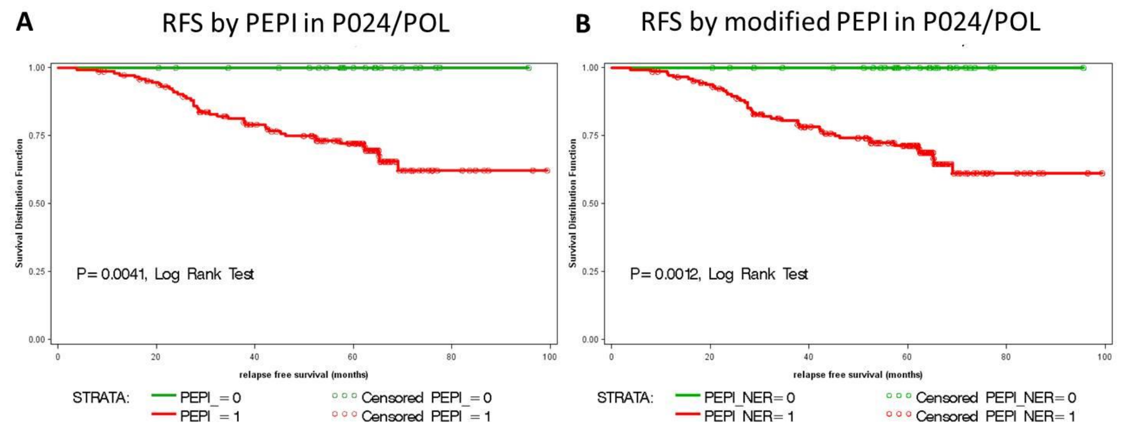

Since fulvestrant down-regulates ER expression and a negative ER in fulvestrant treated tumors may not necessarily reflect a poor outcome, a modified PEPI score that includes all factors, including post-treatment tumor size, node status, and Ki67 level, with the exception of ER, will be used when comparing the PEPI 0 rate between the fulvestrant arms and the anastrozole arm in the ALTERNATE study. To determine whether the rate of PEPI 0 is different from the modified PEPI 0 in AI-treated patients, we reviewed cases from the IMPACT, P024 and ACOSOG Z1031 trials. There have been no instances that excluding ER has led to a change in PEPI 0 rate since post treatment ER negativity almost invariably associates with either high Ki67 or tumor staging which has already rendered the PEPI score being at least one. In the combined P024/POL analysis, no relapse was observed in the 29 patients (19 pT1N0, 10 pT2N0) with modified PEPI 0 (excluding ER) during the median follow up of 62.5 months (Fig 1B).

The ALTERNATE trial is therefore designed to prospectively validate that Modified PEPI score of 0 in the neoadjuvant setting predicts success in RFS, so that in future trials of endocrine agents or combinations, modified PEPI 0 rate could be used as a surrogate marker to screen drugs efficiently. A secondary endpoint is to prospectively examine whether a PEPI score of 0 in the neoadjuvant setting of anastrozole therapy predicts success in RFS.

1.2.3 Evidence of tumor Ki67 2-4 weeks post neoadjuvant therapy in predicting endocrine responsiveness

A limitation of the PEPI is that the prognostic information is available only after months of treatment - an earlier marker of response is needed so that tumors that are not adequately responding to neoadjuvant endocrine therapy can be identified and their treatment plan altered appropriately. Data from previous neoadjuvant endocrine trials indicate that 2-4 week tumor Ki67 expression on endocrine therapy is predictive of individual patient outcome long term [8, 26, 27]. In the IMPACT trial, 2-week Ki67 was a significant independent predictor of RFS (HR = 1.95; 95% CI = 1.23–3.07; P = .004) [27]. The 5-year RFS rates were 85%, 75%, and 60% for the lowest, middle, and highest values of 2-week Ki67 expression, respectively [27]. In the P024 trial, while baseline Ki67 was not associated with relapse, post 16-week treatment Ki67 levels had a robust association with RFS (HR = 1.4, CI = 1.2–1.6 per natural log unit increase; P < .001), and breast cancer–specific survival (HR = 1.4, CI = 1.1–1.7; P = .009) [8].

To further investigate these findings, Ellis et al examined the interaction between Ki67 levels and a gene expression-based definition of luminal A breast cancer versus luminal B breast cancer. By using ROC methodology, a cut point of Ki67 10% served as the best surrogate for the LumA versus LumB distinction [28]. The 10% Ki67 cut point was then applied to the baseline and early on-treatment data in two data sets (Table 3), Preoperative Letrozole study (POL) [25] and IMPACT trial [27]. At baseline the dichotomized Ki67 definition was not significantly predictive for surgical Ki67 level, PEPI score or RFS in these modest sized sample sets. In contrast, high levels of Ki67 on the one month POL samples predicted a higher level of Ki67 in the surgical samples at four months after treatment initiation (P=.01), a poorer PEPI score (P=0.01), a smaller number of patients in the PEPI-0 group (P=0.08) and worse RFS (P=0.003). The IMPACT data confirmed that a 2-week Ki67 >10% predicted higher Ki67 in the surgical specimen (P=0.001), a poorer PEPI score (P=0.001), smaller numbers of patients in the PEPI-0 group (P= 0.004) and worse RFS (P=0.008) (Table 3).

Table 3 Early Ki67 Assessments and Outcome in IMPACT and POL Trials

POL 4W Ki67	% PEPI 0	RFS (events)
>10%	1/19 (5%)	5/21 (23%)
≤10%	10/36 (28%)	1/41 (2.4%)
P Value	P=0.08 (Fisher)	P=0.003 (log rank)
IMPACT 2W Ki67	% PEPI 0	RFS (events)
>10%	0/32 (0%)	9/35 (26%)
≤10%	21/101 (21%)	13/118 (11%)
P Value	P=0.004 (Fisher)	P=0.008 (log rank)

1.2.4 Rationale to evaluate fulvestrant versus anastrozole in the neoadjuvant setting

Fulvestrant is an analog of 17 β-estradiol that binds ER which leads to receptor degradation [29, 30], blocks ER signaling, without any agonist activity [30]. Fulvestrant 250 mg has been shown to be at least as effective as anastrozole [31-33] or exemestane [34] as second-line endocrine therapy for advanced hormone receptor positive (HR+) breast cancer. However, there are biological and clinical data suggesting that fulvestrant 500 mg is more effective than 250 mg and is more effective than anastrozole [35]. In a biomarker preoperative study of fulvestrant administered as a single dose of 50 mg, 125 mg, or 250 mg followed by surgery 14 to 21 days later, a dose-dependent reduction in tumor expression of ER, PR and Ki67 by fulvestrant was observed, but no plateau was reached [22]. The ER H score (a combination of percent ER+ cells and the intensity of staining) was decreased by 39%, 50% and 59% respectively for fulvestrant at 50, 125 and 250mg. In a subsequent randomized neoadjuvant (Neoadjuvant Endocrine Therapy for Women with Estrogen-Sensitive Tumors NEWEST) study of fulvestrant 500 mg versus 250 mg, fulvestrant 500 mg significantly reduced Ki67 levels by more than 78.8% while fulvestrant 250 mg reduced it by only 47.3% (P <.0001) at 4 weeks post single injection [36]. There were also significant differences in the expression of downgraded estrogen receptors between the two doses in favor of the 500 mg dose (P <.0003). Both doses were well tolerated and consistent with the known toxicity profile of fulvestrant. In the fulvestrant 500 mg arm, Ki67 suppression at 4 weeks was similar to that observed at 16 weeks, indicating that the biologic effect of fulvestrant 500 mg has reached steady state by 4 weeks.

CONFIRM (COmparisoN of Faslodex In Recurrent or Metastatic breast cancer) is a randomized, double-blind, parallel-group, multicenter, Phase III study comparing fulvestrant (250 mg) with fulvestrant (500 mg) in postmenopausal women with ER+ advanced disease recurring or progressing after prior endocrine therapy (anti-estrogen or AI) [37]. Progression Free Survival (PFS) was significantly longer for fulvestrant 500 mg (n=362) than 250 mg (n=374) (hazard ratio [HR] = 0.80; 95% CI, 0.68 to 0.94; P = .006), corresponding to a 20% reduction in risk of progression, without increase in toxicity. There was a numerical advantage in clinical benefit rate (CBR) for patients receiving fulvestrant 500 mg vs fulvestrant 250 mg (45.6% vs 39.6%; odds ratio 1.28 [95% CI 0.95, 1.71]; p=0.1), and in duration of benefit (16.6 vs 13.9 months for fulvestrant 500 mg [n=165] and fulvestrant (250 mg) [n=148] respectively). There was a trend for improved Overall Survival (OS) for patients treated with fulvestrant 500 mg compared with fulvestrant 250 mg (HR 0.84 [95% CI: 0.69, 1.03] p=0.091). Based on this data, FDA approved the use fulvestrant 500 mg as the standard dose in place of the 250 mg in postmenopausal women with metastatic HR+ breast cancer progressing following anti-estrogen therapy.

A direct comparison of fulvestrant 500 mg and anastrozole was made in the metastatic setting as first-line endocrine treatments. FIRST was a phase II, open-label, randomized, multi-center, parallel-group study of fulvestrant 500 mg (500 mg IM on Day 0, then 500 mg IM on Days 14 and 28 and every 28 days thereafter) versus anastrozole (1 mg P.O. daily), as first-line treatment for HR+ advanced breast cancer in postmenopausal women [38]. At data cut-off for the primary analysis, performed 6 months after the last patient was randomized, the median time to progression (TTP) was not reached for fulvestrant 500 mg vs 12.5 months for anastrozole (HR 0.63; 95% CI 0.39, 1.00; p=0.0496). A subsequent follow-up data analysis was performed when 79.5% of patients had discontinued study treatment. A total of 205 patients received fulvestrant 500 mg (n=102) or anastrozole (n=103). Median TTP was 23.4 months for the fulvestrant 500 mg group vs 13.1 months for the anastrozole group, corresponding to a 35% reduction in risk of progression (HR 0.66; 95% CI 0.47, 0.92; p=0.01). Median time to treatment failure (TTF) was 17.6 vs 12.7 months for the fulvestrant 500 mg and anastrozole groups, respectively (HR 0.73; 95% CI 0.54, 1.00; p=0.05). Fulvestrant 500 mg was well tolerated and no safety concerns were documented.

These data suggest fulvestrant could be an appropriate candidate for studies in the adjuvant setting, however the sample size for adjuvant endocrine therapy studies is large and the cost is high. The demonstration that fulvestrant clearly had superior biological effectiveness in the neoadjuvant setting would justify the levels of investment required for such investigation. In addition, the longer TTP of fulvestrant arm in the FIRST study suggests that the major benefit of fulvestrant over anastrozole is likely due to its more durable anti-proliferative effect on tumor cells. Therefore, a longer term of therapy is likely necessary to reveal the difference between the two agents. We therefore propose the treatment duration of 24 weeks in the ALTERNATE study, rather than the 16 weeks employed in previously neoadjuvant AI studies, with an optional assessment of tumor Ki67 at 12 weeks if the clinical response is less than a partial response to identify late resistant tumors for triage to chemotherapy or surgery.

Week 12 sample collection → discontinued in Update #07

1.2.5 Rationale to evaluate fulvestrant in combination with anastrozole in the neoadjuvant setting

Fulvestrant and aromatase inhibitors inactivate ER through different mechanisms. Fulvestrant binds to ER and destabilizes the protein, causing a decrease in ER expression. In contrast, aromatase inhibitors (AIs) dramatically reduce estradiol levels. Theoretically these two mechanisms could be synergistic because the decrease in estradiol levels with AI therapy should increase the ability of fulvestrant to bind to ER, and thereby potentiate ER inactivation and down-regulation increasing anti-tumor effects. This concept is supported by preclinical

data, whereby fulvestrant in combination with estrogen deprivation was more effective than either treatment alone in an MCF7-based model of aromatase-dependent ER+ breast cancer xenograft growth [39-41]. In these experiments, the combination of AI plus fulvestrant was associated with lower ER levels than either treatment alone, and both anastrozole and letrozole added significant benefit to fulvestrant in tumor growth delay [39-41]. In addition to ER, greater degrees of down-regulation of IGF-IR, and downstream MAPK and PI3K pathways were also observed compared to single agent therapy [41]. Osborne et al. also found that fulvestrant was more effective in the absence of estrogen (through ovariectomy) in a model of MCF7 xenograft tumor growth inhibition [42].

As a result of these promising preclinical data, three trials were launched to test the AI fulvestrant combination approach in postmenopausal women with metastatic breast cancer (FACT, SOFEA and SWOG 0226). The FACT trial is an open-label, randomized phase III multinational study of fulvestrant in combination with anastrozole versus anastrozole at first relapse in hormone receptor positive breast cancer. Patients were randomized to receive either anastrozole 1 mg PO daily, or anastrozole 1 mg PO daily in combination with fulvestrant 250 mg IM every 28 days, following a loading dose of 500 mg on Day 1, 250 mg on Day 14 and 250 mg on Day 28, during the first cycle [43]. Five hundred and fourteen patients were randomized between January 2004 and March 2008. There was no difference in TTP (10.8 months on combination therapy versus 10.2 months on anastrozole alone), HR 0.99 (95% CI 0.81 – 1.20), p=0.91), clinical benefit rate (CR, PR, SD at 24 weeks) (55.1% in the combination therapy arm and 55.0% in the anastrozole alone arm) or OS (37.8 months in the combination therapy arm versus 38.2 months in the anastrozole alone arm). A slight increase in the incidence of hot flushes was found in the combination arm (24.6%), compared to the anastrozole arm (13.8%), $p < 0.01$. The SOFEA trial (Study of Faslodex with or without concomitant Arimidex vs Exemestane following progression on non-steroidal Aromatase inhibitors) has completed an accrual of approximately 700 patients, with results yet to be published in peer-reviewed journals.

However, the enthusiasm for the combination of fulvestrant and AI approach was renewed after the recent report of SWOG S0226 trial at the 2011 SABCS and the subsequent publication in NEJM [44]. The SWOG S0226 trial is a phase III randomized study of anastrozole versus anastrozole and fulvestrant as first-line therapy for postmenopausal women with metastatic breast cancer. The inclusion criteria of the SWOG S0226 trial were similar to that of the FACT trial. Eligible patients were those with ER and/or PR+ disease by local institutional standards, measurable or non-measurable disease, and no prior chemotherapy, hormonal therapy, or immunotherapy for metastatic disease. Prior adjuvant or neoadjuvant chemotherapy was allowed if completed more than 12 months prior. The use of prior adjuvant tamoxifen was used as a stratification factor. Fulvestrant was administered at the same dose and schedule as in the FACT trial. The primary endpoint was Progression-free survival (PFS). The trial was designed to have a 90% power to detect an increase in median PFS from 10 months (anastrozole monotherapy) to 13 months (combination therapy) with a 2-sided alpha = 0.05. In the intent-to-treat analysis of eligible patients, 707 patients were randomized in the period between June 2004 and July 2009, 694 patients were analyzed, excluding 12 who were ineligible and 1 who withdrew consent. The median PFS and the same definition for the TTP as in the FACT trial, was 15.0 months (95% CI 13.2 -18.4 months) in the combination arm, compared to the 13.5 months (95% CI 12.1 – 15.1 months) in the anastrozole arm, p = 0.007, HR 0.8 (95% CI 0.68 - 0.94), favoring the combination therapy. This was accompanied by a statistically significant improvement in OS (combination therapy: 47.7 months (95% CI 43.4 -55.7) versus the anastrozole arm: 41.3 months (95% CI 37.2 -45.0), p = 0.049, HR 0.81 (95% CI 0.65 -1.00). Interestingly in the unplanned analysis according to prior adjuvant tamoxifen exposure, the improvement in PFS (17 months versus 12.6 months, HR 0.74 [0.59 - 0.92], p = 0.006) and OS

(47.7 months versus 39.7 months, HR 0.74 [0.56 – 0.98], p =0.04) was found only in patients who had no prior adjuvant tamoxifen therapy (n = 414). Adverse events did not differ significantly by treatment group. Grade 3 or higher toxicities were 14.7% in the combination arm and 12.7% on the anastrozole arm (including musculoskeletal pain (2.8%), influenza-like symptoms (2.4%), gastrointestinal disturbances (1.5%) and hematologic effects (1.5%).

The positive S0226 trial stands in contrast to the negative results of the FACT trial. However, there were more endocrine naïve cases in the S0226 trial than in the FACT trial. Compared to the FACT trial, in which 30.2% (combination arm) and 35.2% (anastrozole arm) had no prior endocrine therapy, 60% of patients had no prior endocrine therapy in the S0226 and 38.9% had de novo metastatic disease. These data are consistent with the postulate that the benefit of the combination was restricted to patients who had not received prior tamoxifen treatment. In both trials, the number of patients who had prior adjuvant AI therapy was minimal (2% in the S0226 trial and 1% in the FACT trial). These data, therefore suggest that the population of patients in which to further test the fulvestrant anastrozole combination are those receiving these agents as first line therapy, such as in the adjuvant/neoadjuvant treatment settings. However, a weakness of both trials is that they were conducted with the lower dose of fulvestrant at 250 mg monthly, which is now known to be inferior to the newly approved 500 mg high dose (HD) in both the first and second line settings [37, 38]. Thus, it is not clear that the proposed synergy between anastrozole and fulvestrant seen in S0226 is due to anastrozole "compensating" for inadequate fulvestrant dosing in some patients, or a true positive interaction. Furthermore, neither of these trials had the third arm of fulvestrant alone, which is necessary for a full evaluation of the synergy hypothesis. The ALTERNATE trial provides an opportunity to assess the synergy hypothesis between fulvestrant and anastrozole at the tissue level, through the measurement of on-treatment proliferation and ER levels.

1.2.6 Rationale to assess the rate of pCR and residual disease burden on neoadjuvant weekly paclitaxel or standard chemotherapy regimens in the endocrine resistant (Ki67 level > 10% at 4-week or 12-week biopsy) population and the need for long-term follow-up

Response to neoadjuvant chemotherapy measured by pCR, and post-chemotherapy residual disease burden has been shown to correlate with long-term outcomes [45-47]. Patients who achieved a pCR and minimal residual disease burden have an excellent prognosis, regardless of subtypes of breast cancer [47, 48]. In the case of ER positive breast cancer, a low pCR rate has been consistently observed. In a study of 82 breast cancers, the pCR rate with neoadjuvant paclitaxel followed by 5-fluorouricil, doxorubicin and cyclophosphamide in luminal breast cancer was 6%, compared to 45% observed in both basal-like and HER2-E subtypes defined by gene expression profiling [49]. Similarly, in a study of 107 patients, pCR with neoadjuvant AC (adriamycin and cyclophosphamide) was 7% in the luminal subtype, compared to 36% in HER2+/ER- and 27% in basal-like breast cancer, defined by the immunohistochemistry method [48]. The low pCR rate to neoadjuvant chemotherapy is a reflection of an overall smaller chemotherapy benefit for ER+ disease observed in the adjuvant setting [50]. There is an unmet clinical need to identify predictors of chemotherapy sensitivity in ER+ cancers [51]. Data is also lacking regarding the likelihood of benefit from chemotherapy in the endocrine resistant population. This is a particularly important issue to address since chemotherapy is a standard of care in the adjuvant setting for these patients. In addition, long-term outcome data is needed to provide confidence that future drug development could be aimed at improving the rate of pCR or minimal residual burden in this patient population.

Z1031 cohort B was the first trial that attempted to address the pCR rate to neoadjuvant chemotherapy in the endocrine resistant ER+ population identified by the 2-week tumor Ki67 level > 10% on neoadjuvant endocrine therapy. Thirty-five patients with endocrine resistant ER+ breast cancers were treated with neoadjuvant chemotherapy regimens of physician's

choice. There were two (5.7%, 95% CI: 0.7-19.1%) pathologic complete responses (pCR) among these 35 patients (in press).

In addition to standard neoadjuvant chemotherapy, we have included weekly paclitaxel as an option in a separate cohort, since paclitaxel is a favored chemotherapy partner for molecularly targeted agents being developed. The response data to neoadjuvant paclitaxel will help to provide important historical control data for future trials of combination therapies.

1.3 Rationale for trial design

Z1031 Cohort B, a Risk-adapted Clinical Trials Design for Stage 2 and 3 ER+ Breast Cancer

Figure 2 Schema for Z1031 Cohort B.

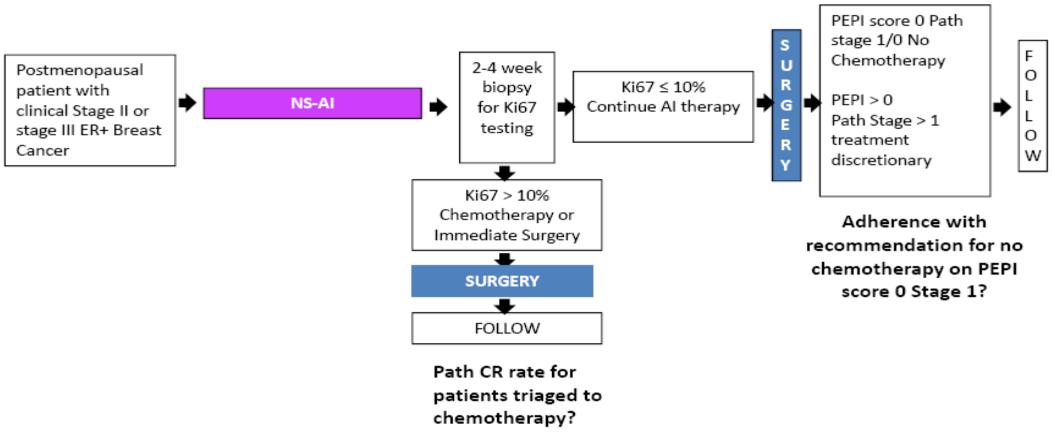

As the Z1031 trial approached its accrual goal ACOSOG received approval to run a pilot extension study (Cohort B) to determine if a triage trial, based on early tumor Ki67 assessment at 2 to 4-week on-treatment biopsy was feasible. The schema outlined in Figure 2 illustrates this approach. Biopsy was mandatory at baseline, 2 to 4 weeks and at surgery. Patients with Ki67 > 10% were triaged to chemotherapy and the objective in this group was to determine the pCR rate to standard chemotherapy. If the early Ki67 < 10%, the patient remains on treatment and in this group the objective is to determine the acceptability of a recommendation that no chemotherapy is necessary for patients with PEPI-0 score.

Of the 236 eligible patients, 49 (21%) patients had a 2 week Ki67 > 10%. There were another 22 patients who lacked sufficient tumor tissue to ascertain a 2 week Ki67: 6 of these patients choose to continue on AI and not be re-biopsied and the remaining 16 patients were re-biopsied after 4 weeks of treatment with a finding of Ki67 ≤ 10%. One patient who had a 2-4 week Ki67 ≤ 10% refused to continued AI treatment. Of the 186 patients who continued on AI, 9 patients did not undergo surgery due to refusal, comorbid conditions, or disease progression; 4 patients who had surgery either did not have nodal surgery or did not have a ki67 value determined from their surgical specimen; and 109 had a non-zero mPEPI score. Thus, we would expect approximately 30% [(236-49-1-9-4-109)/236] percent of the patient enrolled onto this trial to have endocrine sensitive disease.

Data from the Z1031 Cohort B also indicates that 6% of those with Ki67 ≤ 10% at 2-4 week biopsy have an increase in Ki67 to a level above 10% at the time of surgery (following another 14 weeks of therapy), indicating that the antiproliferative effect of AI is not durable in some cases which could potentially be improved upon.

Based on the success of Z1031 Cohort B design and the observation of the rebound Ki67 elevation on prolonged AI treatment, we plan to incorporate early treatment biopsies at 4 weeks (fulvestrant reaches steady state concentration and pharmacodynamic effect by 4 weeks) for Ki67 assay to determine endocrine responsiveness. Patients will continue endocrine therapy only if Ki67 ≤ 10%. An optional biopsy at 12 weeks is recommended if the clinical response is not optimal (i.e. no tumor shrinkage as judged by treating physician) (at 12 weeks. Patients will continue endocrine therapy if Ki67 < 10% and there is no clinical disease progression. If Ki67 > 10% at 4 or 12-week biopsy, it is recommended that patients be treated with weekly paclitaxel or other standard chemotherapy regimens or proceed to surgery.

Week 12 sample collection → discontinued in Update #07

1.4 Significance

The ALTERNATE study will address several important issues and unmet needs in drug development for early stage ER positive breast cancer:

1) It will establish whether modified PEPI 0 can be used as a surrogate marker for long-term outcomes so that future endocrine agents or combinations (for example a biologic agent in combination with either fulvestrant or an aromatase inhibitor) can be screened more efficiently using modified PEPI 0 rate as a primary endpoint before embarking on a definitive adjuvant trial, which requires years to follow and significant number of patients and financial resources.
2) It will provide further validation of early Ki67 assessment and other markers such as intrinsic subtype using public domain bioinformatics, NKI-70 gene, 21 gene recurrence score, as well as other models that may arise, run on non-commercial platforms, as predictors of endocrine sensitivity so that individualized care of patients with early stage breast cancer can be improved.
3) It will evaluate the rate of pCR and pCR/RCB-1 of weekly paclitaxel for endocrine resistant populations, so that promising paclitaxel combinations can be tested against in separate single arm phase II trials in the future.
4) It will evaluate the rate of pCR and pCR/RCB1 of other standard chemotherapy regimens for the endocrine resistant population.
5) It will provide efficacy information on fulvestrant, either alone or in combination with anastrozole, compared to anastrozole alone in postmenopausal women with ER+ early stage breast cancer.

For reasons stated above the results of the A011106 (ALTERNATE) trial, with a moderate sample size, is expected to have a significant importance on drug development and potentially the clinical practice in the setting of early stage ER+ breast cancer to improve the survival of these patients.

1.5 Rationale for the change in study design (protocol Update #05)

In Update # 05, the major change is to modify the design of the second phase of the trial. To reduce sample size, we have modified the primary objectives of the second phase of the trial. The original aims included assessing the 5-year RFS among women with a modified PEPI score of 0 in the anastrozole arm. In addition, depending upon the findings of the first phase of the trial, fulvestrant containing regimens were to be carried forward if their modified PEPI (mPEPI) 0 rate was at least 10% more than that seen in the anastrozole arm. For each of the fulvestrant regimens carried forward into the second phase of the study, as well as the anastrozole arm, we were to assess whether the 5-year RFS among women with a modified PEPI (mPEPI) score of 0 randomized to that arm was at least 95%. As the enrollment rate is less than expected, we have had discussions with NCI Biostatistics Branch to modify the second phase of this trial in order to reduce both its size and duration. Our discussions with NCI Biostatistics Branch lead to the conclusion that the second phase of the trial should center on assessing the 5 year RFS rate among the patients treated with anastrozole whose mPEPI=0 and that a one-sided alpha of 0.05 was more appropriate in this setting than a one-sided alpha of 0.025. With these modifications, as well as setting the accrual rate

to 20 patients per month, and closing enrollment to the fulvestrant containing arms once the accrual goal of 425 patients per arm is met for the first phase of the trial, we recalculated the sample size for the anastrozole arm so that a one-sided alpha=0.05 nonparametric Brookmeyer-Crowley type one sample survival test would have at least an 85% chance of rejecting that the 5 year RFS rate is at most 90% in patients with mPEPI score of 0 after 16 weeks of neoadjuvant anastrozole treatment, when the true 5 year RFS rate in this patient population is at least 95%.

2.0 OBJECTIVES

2.1 Primary objectives

1. To determine whether fulvestrant administered for 24 weeks as neoadjuvant endocrine treatment increases the proportion of endocrine sensitive tumors* relative to patients treated with anastrozole.
2. To determine whether fulvestrant in combination with anastrozole, administered for 24 weeks as neoadjuvant endocrine treatment, increases the proportion of endocrine sensitive tumors* relative to patients treated with anastrozole.
3. If both of the fulvestrant containing arms are found to have an endocrine sensitive disease rate at least 10% higher than that of the anastrozole arm, we will assess whether the endocrine sensitive disease rate is greater with the combination of anastrozole and fulvestrant than with fulvestrant alone.
4. To assess whether the 5-year RFS rate among women treated with anastrozole with a modified preoperative endocrine prognostic index (PEPI) score of 0 following 24 weeks of neoadjuvant treatment is at most 90%.
5. For the fulvestrant containing regimens, a point and interval estimate of the 5 year RFS will be obtained.

* **Endocrine resistance tumor is defined by any one of the following criteria:**

- Ki67 > 10% after 4 weeks on neoadjuvant endocrine therapy;
- Ki67 > 10% after 12 weeks on neoadjuvant endocrine therapy;

Week 12 sample collection → discontinued in Update #07

- progressive disease is documented anytime during neoadjuvant endocrine therapy;
- surgical findings at 21-24 weeks post neoadjuvant endocrine therapy are such that: pT stage is 3/4, positive lymph nodes are present or Ki67 > 2.7% (i.e. modified PEPI score of not being 0);
- discontinued neoadjuvant endocrine treatment for any reason.

A patient who does not meet any of the criteria of endocrine resistant disease will be referred to as having endocrine sensitive disease.

2.2 Secondary objectives

1. To examine the differences in surgical outcome, clinical and radiological response rates, and safety profile between the fulvestrant arm and the anastrozole arm.
2. To examine the differences in surgical outcome, clinical and radiological response rates, and safety profile between patients randomized to fulvestrant in combination with anastrozole and those randomized to anastrozole.
3. To examine the rate of pathologic complete response (pCR) of 12 weeks of neoadjuvant paclitaxel in patients with endocrine resistant disease following 4-weeks or 12-weeks of neoadjuvant endocrine therapy (with either fulvestrant or anastrozole or the combination of fulvestrant and anastrozole).

4. To examine the rate of pathologic complete response (pCR) among those patients with endocrine resistant disease, following 4 weeks or 12-weeks of neoadjuvant endocrine therapy (with either fulvestrant or anastrozole or the combination of fulvestrant and anastrozole), who choose not to receive neoadjuvant paclitaxel, but another standard neoadjuvant taxane and /or anthracycline containing regimen or CMF.
5. To summarize the frequency of severe (NCI CTCAE grade > 3) adverse events encountered with administration of paclitaxel in the neoadjuvant setting.
6. To assess time to breast cancer recurrence for patients with endocrine resistant tumors defined by tumor 1) Ki67 >10% at week 4; 2) Ki67 >10% at week 12; and 3) modified PEPI score of non-zero on neoadjuvant endocrine therapy, with all three groups combined or separated.
7. To determine the impact of NF1 gene copy loss and stop/gain mutations on short and long-term neoadjuvant/adjuvant endocrine therapy outcomes.
8. To assess whether women with ctDNA present after 4 weeks of NET is less likely to achieve mPEPI 0 or pCR among those with week 4 Ki67 ≤ 10% and continued on NET
9. To examine whether the proportion of women with ctDNA present at week 4 differs between those with week 4 Ki67 >10% on NET and those with week 4 Ki67 ≤ 10%
10. To assess whether RCB class differs with respect to the presence of ctDNA after week 4 NET among those with a week 4 Ki67 levels > 10% who switched to neo-adjuvant chemotherapy.

2.3 Correlative science objectives

1. To assess whether the degree of tumor Ki67 suppression at week 4 and surgery differs between patients randomized to fulvestrant and those randomized to anastrozole.
2. To assess whether the degree of tumor Ki67 suppression at week 4 and surgery differs between patients randomized to fulvestrant in combination with anastrozole and those randomized to anastrozole.
3. To examine the impact of tumor ER expression level post-neoadjuvant endocrine therapy on RFS in each treatment arm separately.
4. To examine whether RFS differs with respect to pathologic tumor stage (T1 vs. T2) post-neoadjuvant endocrine therapy in the subgroup of women with a modified PEPI score of 0.
5. To examine whether rate of endocrine resistant tumors or RFS differs with respect to the degree of week 4 Ki67 suppression.
6. To examine whether the rate of week 4 Ki67 level > 10%, the rate of endocrine resistant tumors or RFS differs with respect to pre-treatment gene expression profile.
7. To examine whether gene expression profiles at week 4 can further refine the patient population who have modified PEPI score non-0 or shorter RFS.
8. To assess the pCR/RCB-1 rate in each of the following cohorts:

a. Those who chose to switch to paclitaxel after finding their week 4 Ki67 was > 10%.

Week 12 sample collection discontinued in Update #07 →

b. Those who chose to switch to paclitaxel after finding their week 12 Ki67 was > 10%.

c. Those patients who chose to switch to a standard neoadjuvant taxane and/or anthracycline containing regimen or CMF (rather than paclitaxel) after finding their week 4 Ki67 was > 10%.

Week 12 sample collection discontinued in Update #07 →

d. Those patients who chose to switch to a standard neoadjuvant taxane and/or anthracycline containing regimen or CMF (rather than paclitaxel) after finding their week 12 Ki67 was > 10%.

9. To evaluate Cycle 1, day 2 tumor biopsy following the initiation of paclitaxel to develop early molecular markers of tumor response to paclitaxel.
10. To evaluate tumor tissue, serum, and plasma specimens collected at baseline, on-therapy, and at surgery, and blood collected during follow-up and at recurrence for biomarker discovery (through methods such as gene expression profiling, patterns of gains or losses of DNA, tumor whole genome and targeted DNA and RNA sequencing and proteomics) in studies that aim to understand signaling pathways associated with endocrine therapy and taxane therapy sensitivity and resistance.
11. To compare the RCB profile between NF1-low and NF1-normal tumors triaged to neoadjuvant chemotherapy.
12. Exploratory objectives for ctDNA analysis:
    (a) To examine the association between the presence of pre-NET ctDNA and each of the following patient and disease characteristics: age, race, body mass index, cTstage, cN stage, pre-NET Ki67, tumor grade, histology, breast cancer intrinsic subtype, gene expression or mutation profiles, week 4 Ki67 levels ≤ 10% , week 4 Ki67 ≤ 2.7% (complete cell cycle arrest)
    (b) To assess whether the presence of pre-NET ctDNA decreases the likelihood to achieve mPEPI 0 + pCR among patients with week 4 Ki67 levels ≤ 10% who completed NET or subsequently discontinued NET due to disease progression
    (c) To estimate the proportion of women who maintain ctDNA positivity or attain ctDNA positivity after completion of 4 weeks, or after 24 weeks of NET
    (d) To assess whether the presence of ctDNA at completion of NET decreases the duration of breast cancer-free interval among patients with week 4 Ki67 levels ≤ 10% who completed NET
    (e) To assess whether the duration of breast cancer-free interval is decreased in those with ctDNA present at week 4 NET among patients with a week 4 Ki67 levels > 10% who switched to neo-adjuvant chemotherapy
    (f) To assess whether RCB class or duration of breast cancer-free interval differs with respect to the presence of ctDNA at the completion of NCT among those with a week 4 Ki67 levels > 10% who switched to NCT
    (g) To determine the sensitivity, specificity, and lead-time interval for ctDNA detection during follow up after surgery for distant disease recurrence among high risk patients
    (h) To assess the ctDNA positivity rate at 5 years after surgery and its association with late recurrence among women with a week 4 Ki67 levels ≤ 10% who completed NET
    (i) To assess the ctDNA positivity rate at 5 years after surgery and its association with late recurrence among women with a week 4 Ki67 levels > 10% who switched to neoadjuvant chemotherapy
    (j) To examine changes in ctDNA quantity over time up to surgery during neoadjuvant therapy among patients with week 4 Ki67 >10%, those with week 4 Ki67 ≤ 10%, and mPEPI and RCB categories
    (k) To compare mutation profiles of ctDNA at metastatic recurrence with persistent/emerging mutations in tumor tissues at surgery post neoadjuvant therapy to identify driver mechanisms of recurrence

3.0 SCHEMA

Sample collection of the optional Ki67 week 12 biopsy discontinued in Update #07

Effective 11/01/2018, Arms II and III will be closed to enrollment and all new patients will be assigned to Arm I.

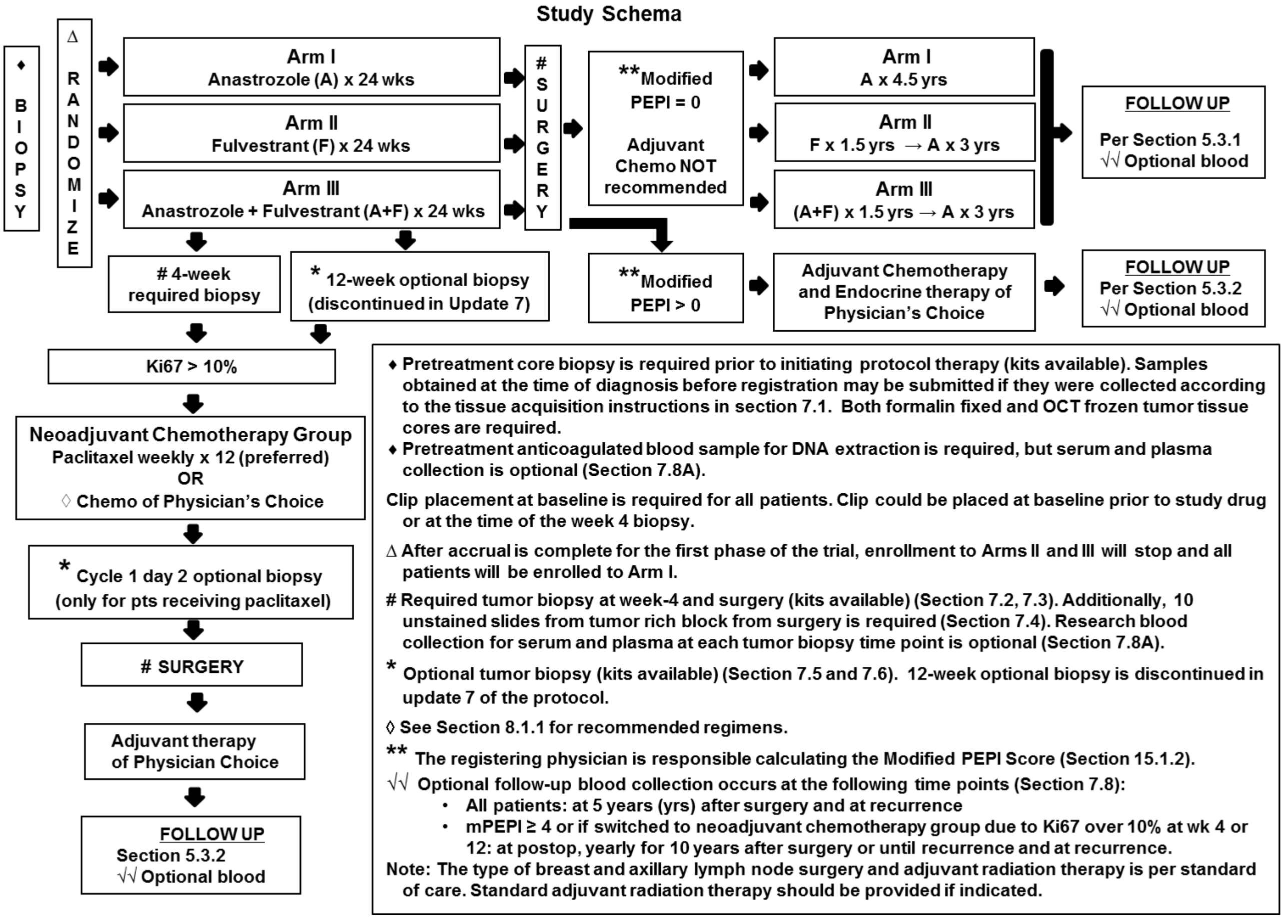

- ♦ Pretreatment core biopsy is required prior to initiating protocol therapy (kits available). Samples obtained at the time of diagnosis before registration may be submitted if they were collected according to the tissue acquisition instructions in section 7.1. Both formalin fixed and OCT frozen tumor tissue cores are required.
- ♦ Pretreatment anticoagulated blood sample for DNA extraction is required, but serum and plasma collection is optional (Section 7.8A).

Clip placement at baseline is required for all patients. Clip could be placed at baseline prior to study drug or at the time of the week 4 biopsy.

∆ After accrual is complete for the first phase of the trial, enrollment to Arms II and III will stop and all patients will be enrolled to Arm I.

# Required tumor biopsy at week-4 and surgery (kits available) (Section 7.2, 7.3). Additionally, 10 unstained slides from tumor rich block from surgery is required (Section 7.4). Research blood collection for serum and plasma at each tumor biopsy time point is optional (Section 7.8A).

* Optional tumor biopsy (kits available) (Section 7.5 and 7.6). 12-week optional biopsy is discontinued in update 7 of the protocol.

◊ See Section 8.1.1 for recommended regimens.

** The registering physician is responsible calculating the Modified PEPI Score (Section 15.1.2).

√√ Optional follow-up blood collection occurs at the following time points (Section 7.8):
- All patients: at 5 years (yrs) after surgery and at recurrence
- mPEPI ≥ 4 or if switched to neoadjuvant chemotherapy group due to Ki67 over 10% at wk 4 or 12: at postop, yearly for 10 years after surgery or until recurrence and at recurrence.

Note: The type of breast and axillary lymph node surgery and adjuvant radiation therapy is per standard of care. Standard adjuvant radiation therapy should be provided if indicated.

See Section 8.0 for the protocol step when disease progression is documented during neoadjuvant treatment, or during post-surgery period.

4.0 PATIENT SELECTION

4.1 On-study guidelines

The following guidelines should be taken into account when considering patients for this trial, although they will not be considered formal eligibility (exclusion) criteria for this protocol.

- If a patient is a cancer survivor, they have undergone potentially curative therapy for all prior malignancies, with no evidence of recurrence > 5 years.
- No prior history of malignancy within 5 years except for successfully treated cervical carcinoma in situ, lobular carcinoma in situ of the breast, or non-melanoma skin cancer.
- Patient is deemed by their treatment physician to be at low risk for recurrence (i.e. < 30%) from prior malignancies.
- Uncontrolled intercurrent illness including, but not limited to, ongoing or active infection, symptomatic congestive heart failure, unstable angina pectoris, cardiac arrhythmia, or psychiatric illness/social situations that would limit compliance with study requirements or make patient not candidate for surgery.
- Psychiatric illness or condition impairing decision making capacity, which would prevent the patient from giving informed consent.

4.2 Eligibility criteria

All questions regarding eligibility criteria should be directed to the Alliance Study Chair. Please note that the Study Chair cannot grant waivers to eligibility.

Each eligibility criterion must be evaluated and documented in the patient's medical record. No eligibility exceptions are permitted. NOTE: All staging examinations must be done at time of diagnosis and prior to preoperative treatment.

A patient will be eligible for inclusion in this study only if **ALL** of the following criteria apply:

1. Female ≥ 18 years of age.
2. ECOG performance status 0-2.
3. Postmenopausal, verified by:

- Post bilateral surgical oophorectomy, or
- No spontaneous menses ≥ 1 year or
- No menses for < 1 year with FSH and estradiol levels in postmenopausal range, according to institutional standards

4. Pathologic confirmation of invasive breast cancer diagnosed by core needle biopsy.
5. Clinical T2-T4c, any N, M0 invasive breast cancer, by AJCC 7th edition clinical staging, with the goal being surgery to complete excision of the tumor in the breast and the lymph node.

   Primary tumor must be:

   - palpable
   - its largest diameter is > 2.0 cm by physical examination or by radiological assessment
   - bi-dimensional measurement by tape, ruler or caliper technique must be provided

   Note:

   - Patients with contralateral ductal carcinoma in situ and/or invasive breast cancer are not eligible.
   - Patients with multi-focal breast cancer (defined as more than one lesion of invasive breast cancer in the same breast separated from the dominant breast lesion by less than 5 cm of

radiologically normal breast tissue) are eligible. If the other lesions have been biopsied (biopsy not required) they must meet the ER/HER2 eligibility requirements. Research biopsies and Ki67 assessment and radiological measures are to be performed on the dominant breast lesion.

6. Invasive breast cancer is estrogen receptor (ER) positive with an Allred score of 6, 7 or 8 by local institution standard protocol. If an Allred Score is not reported on the diagnostic pathology report, ER positivity in > 66% cells is eligible. If ER positivity is ≤ 66%, the staining intensity (weak, intermediate, strong) is needed to calculate the Allred Score to determine eligibility.

7. Invasive breast cancer is HER2 negative

   A patient is considered to have HER2 negative breast cancer if one of the following if one of the following applies:

   (1) 0 or 1+ by IHC and ISH not done.

   (2) 0 or 1+ by IHC or ISH ratio (HER2 gene copy/chromosome 17) < 2

   (3) 2+ by IHC and ISH ratio (HER2 gene copy/chromosome 17) < 2

8. Documentation of mammogram and ultrasound (including DCIS and invasive cancer) of the diseased breast performed within 56 days prior to registration. Mammogram for the unaffected contralateral breast is required within 12 months prior to registration.

9. Laboratory values (≤14 days prior to registration)

   Absolute Neutrophil Count (ANC) > 1,000/mm^3

   Platelet Count > 100,000/mm^3

   Total Bilirubin < 1.5 x upper limits of normal (ULN)

   Creatinine <1.5 x upper limits of normal (ULN)

   Serum ALT < 2.5 x upper limits of normal (ULN)

10. Tissue acquisition: Patient must agree to provide the required research biopsies at baseline, week 4 and at surgery for integral and integrated biomarker and correlative studies.

4.2.1 Contradictions to study registration:

1. Premenopausal status
2. Inflammatory breast cancer defined as clinically significant erythema of the breast and/or documented dermal lymphatic invasion (not direct skin invasion by tumor or peau d'orange without erythema).
3. An excisional biopsy of this breast cancer
4. Hormone replacement therapy of any type, megestrol acetate, or raloxifene within one week prior to registration.
5. Tumor ER Allred score between 0-5 or HER2 positive by IHC (3+) or amplified by FISH > 2.0
6. Surgical axillary staging procedure prior to study entry.

   Note: FNA or core needle biopsy of axillary node is permitted.

7 Clinical or radiographic evidence of metastatic disease. Metastatic workup is not required, but is recommended for patients with clinical stage III disease

   Note: isolated ipsilateral supraclavicular node involvement is permitted

8. Breast implants are contraindicated only if the implant precludes the required research biopsies or interferes with palpating the breast lesion.

9. Treatment for this cancer including surgery, radiation therapy, chemotherapy, biotherapy, hormonal therapy or investigational agent prior to study entry.
10. History of invasive breast cancer or contralateral DCIS.

4.3 Staging criteria

Patients will be staged prior to registration according to the clinical staging criteria adapted from the American Joint Committee on Cancer (AJCC) Cancer Staging Data Forms of the AJCC Cancer Staging Manual, 7th Edition, 2009 (See Appendices).

5.0 STUDY CALENDAR

5.1 All patients: Prior to registration through cycle 2 (week 8)

	No more than 14 days prior to registration	Registration And Randomization	Prior to start of neo-adjuvant endocrine therapy	Neoadjuvant endocrine therapy Day 1 (+/-3 days) of Cycle 2◆
**CLINICAL ASSESSMENT**				
History & physical exam, height, weight, performance status	X			X
Clinical measurements of breast lesions[A]	A			A
Adverse event assessment			X	X
Labs: CBC, Diff, Plts, PT/INR, serum creat, ALT, T. bilirubin	X			
Drug compliance assessment*				X
**RADIOLOGY**				
Mammogram & Ultrasound of breast and axillary masses	B			
**SAMPLE COLLECTION**				
Tumor Biopsies			C	D
Research blood samples			E, F	E

A. Using a standard cm calibrated caliper, measuring tape or ruler, the longest axis and the perpendicular axis of the tumor are to be measured and recorded in metric notation. Baseline evaluation should be done no more than 14 days prior to initiation of study therapy.

B. Baseline mammogram and ultrasound of the diseased breast must be completed within 56 days of registration. Baseline imaging studies must include bi-dimensional breast tumor measurements. Mammogram of the contralateral breast is required to be within 12 months unless a mastectomy was performed.

C. Pre-treatment core biopsies for correlative studies are required prior to the initiation of protocol therapy. Samples obtained at the time of diagnosis before registration to A011106 may be submitted if they were collected according to the tissue acquisition instructions in Section 7.0. It is advised that the baseline samples are harvested during ultrasound guided clip placement to optimize tissue accrual.

D. The week 4 biopsy for both integral Ki67 biomarker assay and correlative studies is required within 3 days before or after Day 1 of Cycle 2 for treatment decisions. If Ki67 is over 10%, patient will discontinue neoadjuvant endocrine protocol therapy, and it is recommended that the patient be switched to 12 weeks of neo-adjuvant paclitaxel (preferred) outlined in Section 8.1.1, or another regimen containing a taxane

and/or anthracycline or CMF regimen, administered per NCCN Guidelines (Neoadjuvant Chemotherapy Group) and then undergo surgery. If, at the discretion of the treating physician, the patient would not be a candidate for neo-adjuvant chemotherapy, the patient may proceed directly to surgery.

E. Patient consent to research blood sample collection (serum and plasma) is optional (see Section 7.0).

F. The baseline anticoagulated whole blood sample for DNA extraction is required for all patients (see Section 7.8)

◆ Ideally the clinic visit and all the study requirements should be performed on Day 1 of Cycle 2, but may be performed within 3 days before or after that day. Each cycle is 28 days.

* Anastrozole medication diary is to be used in Arms I, and III.

5.2 Study calendar cycle 3 through surgery

5.2.1 Patients continuing on Arm I, Arm II or Arm III after week 4 biopsy

	Neoadjuvant endocrine therapy Day 1(+/-3 days) of Cycles 3 to 6	At completion of neoadjuvant endocrine therapy	Discontinuation of neoadjuvant endocrine therapy due to tumor progression **	SURGERY*	Post-op follow-up
**CLINICAL ASSESSMENT**					See Section 5.3.1 for post-op follow-up schedule for patients with ***modified PEPI 0 score***.
History & physical exam, weight, performance status	X		X		
Clinical measurements of breast lesions[A]	A		A		
Adverse event assessment	X	F	X		
Drug compliance assessment♦	X	X	X		
**RADIOLOGY**					See Section 5.3.2 for post-op follow-up schedule for patients with ***a modified PEPI Non-0 score.***
Mammogram & Ultrasound of breast and axillary masses		C	X		
**SAMPLE COLLECTION**					
Tumor Biopsies	B			D	
Research blood samples				E	

* Surgery should be performed during days 7-28 of cycle 6 (the last dose of neoadjuvant anastrozole is the day before surgery. The last dose of neoadjuvant fulvestrant is day 1 of cycle 6. In the fulvestrant and anastrozole + fulvestrant arms, if surgery is delayed beyond the last day of cycle 6, fulvestrant should be given 28 (+/- 3) days after the last fulvestrant dose. Note that all protocol treatment given from day 1 of cycle 6 until surgery should be entered on the Cycle 6 treatment CRFs. Lymph node sampling should be performed at the time of definitive surgery so that modified PEPI score can be calculated. The choice of breast surgery (partial mastectomy vs mastectomy) and axillary lymph node evaluation (sentinel lymph node with or without axillary lymph node dissection) will be determined by the treating surgeon according to institutional standard.

** If breast tumor progression is suspected by measuring tape, ruler or caliper measurement, confirm with bidimensional measurements by mammogram/ultrasound. If axilla tumor progression is suspected by clinical assessment, then confirm by ultrasound.

If progression (breast and/or axilla) is confirmed, at the investigator's discretion, operate immediately or begin other anti-neoplastic approaches such as chemotherapy or radiation therapy. If progressive disease is confirmed and the patient will receive immediate surgery, obtain the optional research tumor tissue samples and for patients who have consented the research blood samples at the time of surgery. Continue study medication until the day before surgery.

A. Using a standard cm calibrated caliper, measuring tape or ruler, the longest axis and the perpendicular axis of the tumor are to be measured and recorded in metric notation.

Week 12 sample collection discontinued in Update #07→

B. Patient consent to core biopsies for integral Ki67 biomarker assay and correlative studies at week 12 (cycle 4 day 1 [+/- 3 days]) if clinical response (by breast exam) is not optimal (i.e. not tumor shrinkage or as judged by treating physician) or if the 4-week Ki67 assay was unsuccessful, is optional (see Section 7.0). If Ki67 > 10%, the patient will discontinue neo-endocrine protocol therapy and it is recommend that the patient be to switched to 12 weeks of neoadjuvant paclitaxel (preferred), as outlined in Section 8.1.1, or another regimen containing a taxane and/or anthracycline or CMF regimen per NCCN Guidelines in (Neoadjuvant Chemotherapy Group) and then undergo surgery. If, at the discretion of the treating physician, the patient would not be a candidate for neoadjuvant chemotherapy, the patient may proceed to surgery.

C. Mammogram and ultrasound of the diseased breast within 42 days prior to surgery.

D. Required research tumor tissue biopsies can be obtained at the time of surgery or pre-operatively with a core biopsy (up to 1 week prior to surgery) during standard of care localization procedure (such as radioactive seed placement). In addition to the tumor tissue biopsies, required sample submission also includes 10 Superfrost Plus unstained slides from tumor rich block after surgery for Ki67 and correlative studies, see (Sections 7.3 and 7.4). The slides will be shipped separately from the surgical samples.

E. Patient consent to research blood sample is optional (see Section 7.0). The sample can be obtained at the time of surgery for consented patients, see Section 7.8 for details.

F. Adverse event assessment should be conducted at the pre-operative evaluation.

◆ Anastrozole medication diary is to be used in Arms I, and III. Note: The medication diary for the last cycle of anastrozole prior to surgery, may be given to the treating physician at the post-operative visit as anastrozole should be taken until the day before surgery.

Week 12 sample collection Ki67→ discontinued in Update #07

5.2.2 Patients switching to the Neoadjuvant Chemotherapy Group due to determination of Ki67 > 10% at week 4 or 12 biopsy

	Prior to the start of neo-adjuvant chemo-therapy	Neoadjuvant chemotherapy Day 1 (+/-3 days) of each cycle*	At completion of the last cycle of neoadjuvant chemotherapy	SURGERY**	Post-op Follow-up
**CLINICAL ASSESSMENT**					See Section 5.3.2 for post-op follow-up schedule
Height	X				
History & physical exam, weight, performance status, BSA	X	X	X		
Clinical measurements of breast lesions[A]	A	A	A		
Adverse event assessment	X	X	X		
**LABORATORY**					
CBC+D, Platelets	X	B	X		
Serum Creat, T. Bili, ALT	X	X	X		
**RADIOLOGY**					
Mammogram & Ultrasound of breast and axillary masses			F		
**SAMPLE COLLECTION**					
Tumor Biopsies		C		E	
Research blood samples		D		E	

* Cycle length is dependent on the type of chemotherapy regimen administered. See Section 8.1 for recommended paclitaxel (preferred) regimen and NCCN chemotherapy regimens.

** Surgery is scheduled between 3-6 weeks following the last dose of paclitaxel or NCCN chemotherapy regimen. Lymph node sampling should be performed at the time of definitive surgery so that cancer residual burden can be calculated. The choice of breast surgery (partial mastectomy vs mastectomy) and axillary lymph node evaluation (sentinel lymph node with or without axillary lymph node dissection) will be determined by the treating surgeon according to institutional standard.

A. Using a standard cm calibrated caliper, tape or ruler, the longest axis and the perpendicular axis of the tumor are to be measured and recorded in metric notation.

B. Weekly prior to each paclitaxel infusion. For other chemotherapy regimens, evaluate per institutional standard of care.

C. For patients receiving paclitaxel, optional tumor biopsy is performed on day 2 of cycle 1.

D. For patients receiving paclitaxel, optional research blood sample is collected on day 2 of cycle 1.

E. The end of therapy tumor collection can be taken at the time of surgery, or pre-operatively with a core biopsy (up to 1 week prior to prior to surgery) during standard of care localization procedure (such as radioactive seed placement). Research optional blood sample may be taken at the time of surgery.

F. Mammogram and ultra sound of the diseased breast to be completed within 42 days prior to surgery.

5.3 Post-operative study calendar

5.3.1 Patients who continued on Arm I, Arm II or Arm III after week 4 biopsy and with modified PEPI score 0

	**Adjuvant Therapy** $^{A, B}$ **(per randomization assignment) +/- RT**^{B} Post-op visit within 2-4 weeks after surgery Every 6 months (+/- 3 months) years 1-5 post surgery$^{\Delta}$	**Clinical Monitoring** Yearly (+/- 3 months) for years 6-10 post surgery$^{\Delta}$ until disease progression	Following documentation of disease recurrence, patients will enter the survival and disease status follow-up period of the study See Section 9.0
**CLINICAL ASSESSMENT**			
History & physical exam, weight, performance status	X		X
Clinical breast exam	C	X	C
Adverse event assessment	X		
Drug compliance assessment*	X		
**RADIOLOGY**			
Mammogram of remaining breast(s)	D	D	D
**RESEARCH BLOOD**			
Research blood	E	E	E

A See Sections 8.5 - 8.7: Adjuvant therapy should begin 2-8 weeks following the last surgery date. Adjuvant chemotherapy is not recommended. Participation in other adjuvant pharmaceutical intervention trials is not permitted.

B Administration of whole breast radiation, rather than partial breast or brachytherapy, is recommended in those for whom adjuvant radiation is indicated.

C Clinical breast exam is performed at each visit.

D Patients with breast tissue remaining will undergo annual mammograms of both breasts. All routine mammograms that are unlinked to clinic appointments may be performed within a +/- 6-month window.

E Optional follow-up research blood (see Section 7.8.1) is collected at 5 years (+/- 6 months) after surgery and at recurrence if applicable. Kits are available.

Δ The timing of the office visit and imaging is also indicated if recurrence is suspected.

* Anastrozole pill diary is to be used in Arms I, and III and in Arm II after switching to anastrozole.

5.3.2 Patients who continued on Arm I, Arm II or Arm III after week 4 biopsy and who had Non-0 modified PEPI score OR Patients who switched to the Neoadjuvant Chemotherapy Group

<table>
<tr><th></th><th>Adjuvant Therapy[A]
Every 6 months (+/- 3 months) years 1-5 post surgery△</th><th>Clinical Monitoring
Yearly (+/- 3 months) years 6-10 post surgery△</th><th rowspan="7">Following documentation of disease recurrence patients will enter the survival and disease status follow-up period of the study

See Section 9.0</th></tr>
<tr><td>CLINICAL ASSESSMENT</td><td></td><td></td></tr>
<tr><td>Clinical breast exam</td><td>X B</td><td>X</td></tr>
<tr><td>RADIOLOGY</td><td></td><td></td></tr>
<tr><td>Mammogram of remaining breast(s)</td><td>C</td><td>C</td></tr>
<tr><td>RESEARCH BLOOD</td><td></td><td></td></tr>
<tr><td>Research blood</td><td>D</td><td>D</td></tr>
</table>

A Adjuvant therapy should begin 2-8 weeks following the last surgery date.

- Standard adjuvant endocrine therapy of treating physician’s choice is administered for 4.5 years or longer (see Section 8.6). Participation in other adjuvant trials is permitted.
- Administration of whole breast radiation, rather than partial breast or brachytherapy, is recommended for patients in whom adjuvant radiation is indicated.
- The choice and regimen of adjuvant chemotherapy is at the discretion of the treating physician. Participation in other adjuvant trials is permitted.

B Post-op visit within 2-4 weeks after surgery to include history, physical examination, weight and performance status.

C Patients with breast tissue remaining will undergo annual mammograms of both breasts. All routine mammograms that are unlinked to clinic appointments may be performed within a +/- 6-month window.

D Optional follow-up research blood (see Section 7.8.1; kits are available) is collected at the following time points (+/- 6 months acceptable for yrs 1-10 collection):

- All patients: at 5 yrs (+/- 6 months) after surgery and at recurrence (any time prior to the start of new treatment)
- mPEPI ≥ 4 or if switched to neoadjuvant chemotherapy due to Ki67 > 10% at wk 4 or 12: at postop visit (2-8 wks post surgery), yearly for years 1-10 (+/- 6 months) post surgery until recurrence and at recurrence (any time prior to the start of new treatment)

  Week 12 applies only to those patients who underwent or consented to the 12-week biopsy prior to Update #07.

△ The timing of the office visit and imaging is also indicated if recurrence is suspected.

6.0 PATIENT REGISTRATION AND RANDOMIZATION

6.1 Investigator and Research Associate Registration with CTEP

Food and Drug Administration (FDA) regulations and National Cancer Institute (NCI) policy require all individuals contributing to NCI-sponsored trials to register and renew their registration annually. To register, all individuals must obtain a Cancer Therapy Evaluation Program (CTEP) Identity and Access Management (IAM) account at https://ctepcore.nci.nih.gov/iam. In addition, persons with a registration type of Investigator (IVR), Non-Physician Investigator (NPIVR), or Associate Plus (AP) must complete their annual registration using CTEP's web-based Registration and Credential Repository (RCR) at https://ctepcore.nci.nih.gov/rcr.

RCR utilizes five person registration types.

- • IVR — MD, DO, or international equivalent;
- NPIVR — advanced practice providers (e.g., NP or PA) or graduate level researchers (e.g., PhD);
- AP — clinical site staff (e.g., RN or CRA) with data entry access to CTSU applications such as the Roster Update Management System [RUMS], OPEN, Rave, acting as a primary site contact, or with consenting privileges;
- Associate (A) — other clinical site staff involved in the conduct of NCI-sponsored trials; and
- Associate Basic (AB) — individuals (e.g., pharmaceutical company employees) with limited access to NCI-supported systems.

RCR requires the following registration documents:

Documentation Required	IVR	NPIVR	AP	A	AB
FDA Form 1572	✓	✓			
Financial Disclosure Form	✓	✓	✓		
NCI Biosketch (education, training, employment, license, and certification)	✓	✓	✓		
GCP training	✓	✓	✓		
Agent Shipment Form (if applicable)	✓				
CV (optional)	✓	✓	✓		

An active CTEP-IAM user account and appropriate RCR registration is required to access all CTEP and Cancer Trials Support Unit (CTSU) websites and applications. In addition, IVRs and NPIVRs must list all clinical practice sites and Institutional Review Boards (IRBs) covering their practice sites on the FDA Form 1572 in RCR to allow the following:

- Addition to a site roster;
- Assign the treating, credit, consenting, or drug shipment (IVR only) tasks in OPEN;
- Act as the site-protocol Principal Investigator (PI) on the IRB approval; and
- Assign the Clinical Investigator (CI) role on the Delegation of Tasks Log (DTL)

Additional information is located on the CTEP website at https://ctep.cancer.gov/investigatorResources/default.htm. For questions, please contact the RCR Help Desk by email at RCRHelpDesk@nih.gov.

6.2 Cancer Trials Support Unit Registration Procedures

This study is supported by the NCI Cancer Trials Support Unit (CTSU).

IRB Approval

For CTEP and Division of Cancer Prevention (DCP) studies open to the National Clinical Trials Network (NCTN) and NCI Community Oncology Research Program (NCORP) Research Bases after March 1, 2019, all U.S.-based sites must be members of the NCI Central Institutional Review Board (NCI CIRB). In addition, U.S.-based sites must accept the NCI CIRB review to activate new studies at the site after March 1, 2019. Local IRB review will continue to be accepted for studies that are not reviewed by the CIRB, or if the study was previously open at the site under the local IRB. International sites should continue to submit Research Ethics Board (REB) approval to the CTSU Regulatory Office following country-specific regulations.

Sites participating with the NCI CIRB must submit the Study Specific Worksheet for Local Context (SSW) to the CIRB using IRBManager to indicate their intent to open the study locally. The NCI CIRB's approval of the SSW is automatically communicated to the CTSU Regulatory Office, but sites are required to contact the CTSU Regulatory Office at CTSURegPref@ctsu.coccg.org to establish site preferences for applying NCI CIRB approvals across their Signatory Network. Site preferences can be set at the network or protocol level. Questions about establishing site preferences can be addressed to the CTSU Regulatory Office by email or calling 1-888-651-CTSU (2878).

Sites using their local IRB or REB, must submit their approval to the CTSU Regulatory Office using the Regulatory Submission Portal located in the Regulatory section of the CTSU website. Acceptable documentation of local IRB/REB approval includes:

- Local IRB documentation;
- IRB-signed CTSU IRB Certification Form; and/or
- Protocol of Human Subjects Assurance Identification/IRB Certification/Declaration of Exemption Form.

In addition, the Site-Protocol Principal Investigator (PI) (i.e. the investigator on the IRB/REB approval) must meet the following criteria in order for the processing of the IRB/REB approval record to be completed:

- Holds an active CTEP status;
- Rostered at the site on the IRB/REB approval (applies to US and Canadian sites only) and on at least one participating roster;
- If using NCI CIRB, rostered on the NCI CIRB Signatory record;
- Includes the IRB number of the IRB providing approval in the Form FDA 1572 in the RCR profile; and
- Holds the appropriate CTEP registration type for the protocol.

Additional Requirements

Additional requirements to obtain an approved site registration status include:

- An active Federal Wide Assurance (FWA) number;
- An active roster affiliation with the Lead Protocol Organization (LPO) or a Participating Organization (PO); and
- Compliance with all protocol-specific requirements (PSRs).

6.2.1 Downloading Site Registration Documents

Download the site registration forms from the protocol-specific page located on the CTSU members' website. Permission to view and download this protocol and its supporting documents is restricted based on person and site roster assignment. To participate, the institution and its associated investigators and staff must be associated with the LPO or a Protocol Organization (PO) on the protocol. One way to search for a protocol is listed below.

- Log in to the CTSU members' website (https://www.ctsu.org) using your CTEP-IAM username and password;
- Click on Protocols in the upper left of the screen
    - Enter the protocol number in the search field at the top of the protocol tree; or
    - Click on the By Lead Organization folder to expand, then select Alliance, and protocol number A011106.
- Click on Documents, select Site Registration, and download and complete the forms provided. (Note: For sites under the CIRB, IRB data will load automatically to the CTSU.)

6.2.2 Submitting Regulatory Documents

Submit required forms and documents to the CTSU Regulatory Office using the Regulatory Submission Portal on the CTSU website.

To access the Regulatory Submission Portal log in to the CTSU members' website, go to the Regulatory section and select Regulatory Submission.

Institutions with patients waiting that are unable to use the Regulatory Submission Portal should alert the CTSU Regulatory Office immediately at 1-866-651-2878 in order to receive further instruction and support.

6.2.3 Checking Site's Registration Status

Site registration status may be verified on the CTSU members' website.

- Click on Regulatory at the top of the screen;
- Click on Site Registration; and
- Enter the sites 5-character CTEP Institution Code and click on Go.
    - Additional filters are available to sort by Protocol, Registration Status, Protocol Status, and/or IRB Type.

Note: The status shown only reflects institutional compliance with site registration requirements as outlined within the protocol. It does not reflect compliance with protocol requirements for individuals participating on the protocol or the enrolling investigator's status with NCI or their affiliated networks.

6.2.4 Patient Registration Requirements

The Oncology Patient Enrollment Network (OPEN) is a web-based registration system available on a 24/7 basis. OPEN is integrated with CTSU regulatory and roster data and with the LPOs registration/randomization systems or the Theradex Interactive Web Response System (IWRS) for retrieval of patient registration/randomization assignment. OPEN will populate the patient enrollment data in NCI's clinical data management system, Medidata Rave.

Requirements for OPEN access:

- A valid CTEP-IAM account;

- To perform enrollments or request slot reservations: Must be on an LPO roster, ETCTN corresponding roster, or participating organization roster with the role of Registrar. Registrars must hold a minimum of an Associate Plus (AP) registration type;
- If a Delegation of Tasks Log (DTL) is required for the study, the registrars must hold the OPEN Registrar task on the DTL for the site; and
- Have an approved site registration for the protocol prior to patient enrollment.

To assign an Investigator (IVR) or Non-Physician Investigator (NPIVR) as the treating, crediting, consenting, drug shipment (IVR only), or receiving investigator for a patient transfer in OPEN, the IVR or NPIVR must list the IRB number used on the site's IRB approval on their Form FDA 1572 in RCR. If a DTL is required for the study, the IVR or NPIVR must be assigned the appropriate OPEN-related tasks on the DTL.

Prior to accessing OPEN, site staff should verify the following:

- Patient has met all eligibility criteria within the protocol stated timeframes; and
- All patients have signed an appropriate consent form and HIPAA authorization form (if applicable).

Note: The OPEN system will provide the site with a printable confirmation of registration and treatment information. You may print this confirmation for your records.

Access OPEN at https://open.ctsu.org or from the OPEN link on the CTSU members' website. Further instructional information is in the OPEN section of the CTSU website at https://www.ctsu.org or https://open.ctsu.org. For any additional questions, contact the CTSU Help Desk at 1-888-823-5923 or ctsucontact@westat.com.

6.3 Stratification factors

Clinical tumor stage:	1) T2
	2) T3
	3) T4 a-c
Clinical lymph node status:	1) Positive
	2) Negative
Performance Status:	1) 0 or 1
	2) 2

6.4 Registration to correlative and companion studies

There is one substudy with Alliance A011106 that is described in Section 15.0 of the protocol. This companion study must be offered to all patients enrolled on Alliance A011106 (although patients may opt not to participate). This substudy does not require separate IRB approval. The substudy included within Alliance A011106 is:

- A detailed description of biomarker analyses A011106-ST is found in Sections 15.2.

If a patient answers "yes" to question #1 A011106-ST they have consented to participate in the correlative study and should be registered within the OPEN system at the same time that s/he is registered to the treatment trial A011106.

If the patient answers "yes" to question #2 A011106-ST have consented to participate in the correlative study and should be registered within the OPEN system at the same time that s/he is registered to the treatment trial A011106.

If a patient answers "yes" to question #3 they have consented to participate in the correlative study A011106-ST and should be registered within the OPEN system at the same time that s/he is registered to the treatment trial A011106.

If a patient answers "yes" to question #4 they have consented to participate in the correlative study A011106-ST and should be registered within the OPEN system at the same time that s/he is registered to the treatment trial A011106.

If patient answers "yes" to question #5 and/or question #6, responses should be captured on the "Consent Status for Follow-up Blood Collection" CRF in Medidata Rave, and the samples should be logged in BioMS and recorded on the "Specimen Submission: Blood (Follow-Up)" form in Rave once collected. Patients should not be registered in OPEN to the A011106-ST substudy for questions #5 and #6.

6.5 Treatment arms

A treatment cycle is defined to be 4 weeks in length.

First Phase of the trial: Neoadjuvant comparison phase

Patients will be randomized to one of the 3 following treatment arms:

Arm I: Anastrozole arm: Anastrozole po daily for 6 cycles* followed by surgery.

- For the modified PEPI 0 group, adjuvant anastrozole is to be administered for 54 cycles (approximately 4.5 years after surgery is completed). Standard of care endocrine therapy beyond 5 years or protocol therapy is per physician discretion. Adjuvant chemotherapy is not recommended. Patients may ***not*** participate in other adjuvant pharmaceutical trials.
- For the modified PEPI Non-0 group it is recommended that adjuvant chemotherapy and endocrine therapy of the treating physician's choice be administered. Patients may participate in other adjuvant pharmaceutical trials.

Arm II: Fulvestrant arm: Fulvestrant IM on days 1 & 15 of cycle 1 and day 1 of cycles 2-6* followed by surgery.

- For the modified PEPI 0 group, adjuvant fulvestrant is to be administered for 18 cycles (approximately 1.5 years) after surgery is completed and when fulvestrant treatment is complete begin anastrozole for an additional 36 cycles (approximately 3 years). Standard of care endocrine therapy beyond 5 years of protocol therapy is per physician discretion. Adjuvant chemotherapy is not recommended. Patients may ***not*** participate in other adjuvant pharmaceutical trials.
- For the modified PEPI Non-0 group it is recommended that adjuvant chemotherapy and endocrine therapy of the treating physician's choice be administered. Patients may participate in other adjuvant pharmaceutical trials.

Arm III: Anastrozole + Fulvestrant arm: Anastrozole po daily in combination with fulvestrant IM on days 1 & 15 of cycle 1, and on day 1 of cycles 2-6*, followed by surgery.

- For the modified PEPI 0 group, adjuvant fulvestrant plus anastrozole are to be administered for 18 cycles (approximately 1.5 years) after surgery is completed and when fulvestrant treatment is complete continue anastrozole for an additional 36 cycles (approximately 3 years post surgery). Standard of care endocrine therapy beyond 5 years of protocol therapy is per physician discretion. Adjuvant chemotherapy is not recommended. Patients may ***not*** participate in other adjuvant pharmaceutical trials.
- For the modified PEPI Non-0 group it is recommended that adjuvant chemotherapy and endocrine therapy of the treating physician's choice be administered. Patients may participate in other adjuvant pharmaceutical trials.

Second phase of the trial

Patients will be enrolled only to the anastrozole arm during the period while the primary endpoint for the neoadjuvant phase of the trial is being assessed.

- In both phases of the trial, if a patient is found to have endocrine therapy resistant disease, defined by Ki67 > 10% at the 4-week biopsy, the patient will discontinue neoadjuvant endocrine protocol therapy, and it is recommended that the patient be switched to 12 weeks of neoadjuvant paclitaxel (the Neoadjuvant Chemotherapy Group) as outlined below and in Section 8.1, or alternatively another regimen containing a taxane and/or anthracycline or CMF regimen administered per NCCN Guidelines, and then proceed to surgery. If at the discretion of the treating physician, the patient would not be a candidate of neoadjuvant chemotherapy, the patient may proceed to surgery.
- Neoadjuvant Chemotherapy Group: Paclitaxel 80mg/m^2 IV on days 1, 8, 15 and 22 every 4 weeks for 3 cycles followed by surgery.

  OR

  A chemotherapy regimen according to NCCN Guidelines.

7.0 SPECIMEN SUBMISSION FOR ESSENTIAL INTEGRAL AND INTEGRATED BIOMARKER AND CORRELATIVE STUDIES

Tissue, serum and plasma specimens are to be sent under controlled conditions to the Alliance WUSTL, which is based at the Washington University School of Medicine (see Section 7.11). The WUSTL is fully operational and is funded through a mechanism that is independent from both this proposal and the funding of the correlative studies. Since the tissues and demographic data will be collected prospectively, it will be possible to use the stored tissues for subsequent analysis and correlative studies.

All patients will have the required core biopsies taken pretreatment, at week 4 and end of neoadjuvant treatment (at the time of surgery) for integral biomarker and correlative studies. Additionally, anti-coagulated whole blood collection for germline DNA at baseline is required for all patients consented after Update #06. Neoadjuvant serum and plasma collection at pretreatment, week 4, and end of neoadjuvant phase (at the time of surgery) as well as follow-up plasma and whole blood collection in BCT-Streck tubes for ctDNA and potentially other circulating biomarker analysis are optional as described in Section 7.8.

Note that all patients on the study will be reconsented for the follow-up plasma and Streck tube whole blood collection for circulating tumor DNA (ctDNA) and potentially other circulating biomarkers after the activation of Update #08. See Section 7.8.1. No germline DNA sample collection is required for any patient at the time of this re-consent.

For patients who have already passed the earlier time points in their treatment course at the time of Update #08 activation, follow-up plasma and Streck tube whole blood collection starts at the next immediate time point or at the next visit if the missing time point was the final time point in the schedule (e.g. if a patient has a mPEPI score of 0-3 and misses the 5 year post-surgery timepoint).

Tissue sample collection: All patients must be offered participation in the optional tissue specimen collections for correlative science (although patients may opt not to participate) at the following time points:

Week 12 sample collection → discontinued in Update #07

1) At week 12 (for patients on Arms I, II or III if the clinical response is not optimal (i.e. no tumor shrinkage as judged by treating physician), or if the week 4 biopsy was unsuccessful in the Ki67 assay).

   Note: Patients are allowed to proceed with the optional week 12 biopsy if they consented to it prior to Update #07.

2) On day 2 of Cycle 1 for patients in the Neoadjuvant Chemotherapy Group, who chose to receive neoadjuvant paclitaxel.

3) Patients on Arms I, II, III and the Neoadjuvant Chemotherapy Group following disease progression.

When possible, to avoid extra procedures we encourage obtaining the research tumor tissues concurrently with another procedure (such as clip placement, axillary node FNA).

Blood sample collection: All patients must also be offered participation in the optional blood specimen collections for correlative studies (although patients may opt not to participate) according to the schedule in Section 7.8.

While participation in the optional tissue specimen collections for research was offered to patients at the time of initial consent to the study, it is possible that the patient may change her mind during the course of the study treatment. For example, a patient who initially opted “No” to the week 12 biopsy at the time of initial consent may decide to proceed with the biopsy at week 12.

Week 12 sample collection → discontinued in Update #07

In this situation, the patient will need to amend their original consent form by striking out the previous denial of the week 12 biopsy and then checking "yes" to the 12 week biopsy and signing her initials and adding the date in the margin. The site must then send the first page, the page with the amended research biopsy question with patient initials and the date in the margin and the final signature page with the de-identified original consent date (the patient’s Alliance ID number must be on all consent pages that are submitted) and a copy of the original OPEN Enrollment form the mailbox shared by the Registration/Randomization office at: random01@mayo.edu or fax to 507-284-0885. The Registration/Randomization office will update the patient’s OPEN Registration Application and will push it into BioMs so that database is updated as well.

All questions regarding sample processing and submission should be directed to the Alliance Biorepository at Washington University (WUSTL).

Specialized neoadjuvant tissue specimen collection/shipment kits and follow-up plasma and Streck tube blood collection/shipment kits will be used for this study. Kits should be ordered through BioMS, at https://bioms.wustl.edu/bioms/login, at the time that the trial protocol is submitted for local IRB approval. Each tissue specimen collection/shipment kit contains the necessary materials that are needed for the biospecimen (tissue and blood) collection at one time point on this trial. For additional information the guidelines for collection, handling, and storage of specimens from the National Cancer Institute (NCI) Cooperative Group and Breast International Group (BIG) Breast Cancer Clinical Trials may be accessed at: http://ctep.info.nih.gov/investigatorResources/tbci/trials.htm. Unused kits should be returned to the Alliance WUSTL biorepository.

NOTE: If the patient does not give consent for banking, tissue and blood leftover from the required correlative studies (questions 5 and 6 in the A011106 Model Consent) will be discarded and will not be returned to the submitting institution.

Specimen Collection Schedule

	Baseline	Week 4	Day 2 (Neoadj. paclitaxel group)	End of neo-adj. treatment (at surg)	Disease progression on neoadj. therapy	Post-op (2-8 weeks after surg)	Follow -up*	5 years (+/- 6 mos) after surgery	Disease recur-rence
**Mandatory for all patients**									
**Tumor biopsy**	X	X		X[3]					
**Whole Blood Germline DNA[4]**	X								
**For patients who consent to optional sample collection**									
**Whole Blood Germline DNA**		X	X	X	X				
**Serum/ plasma**	X	X	X	X	X				
**Tumor biopsy**			X	X[3]	X				
**Plasma and Streck tube whole blood (ctDNA)**						X[2]	X[2]	X[1]	X[1]

*Yearly for years 1-10 (+/-6 mos) post-surgery until recurrence

[1]For all patients

[2]For patients with mPEPI score ≥ 4 or if switched to neoadjuvant chemotherapy due to Ki67 > 10% at wk 4 or 12 (week 12 applies only to those patients who underwent or consented to the 12-week biopsy prior to Update #07).

[3]Additionally, 10 unstained Superfrost Plus slides cut from a tumor rich block from the surgically resected tumor are to be submitted for patients who completed neoadjuvant endocrine therapy on Arms I, II, or III.

[4]Mandatory only for patients consented after Update #06.

7.1 Required pre-treatment core biopsy for biomarker and correlative studies

- Samples collection is mandatory for all patients prior to initiating study therapy
- It is recommended to use the specimen collection/shipping kits provided.
- The needle used to obtain the core biopsies should be 14 G.
- It is strongly suggested that core biopsies be image guided.
- The required biopsies include two core biopsies placed in 10% buffered formalin (a single pre-filled formalin container is provided in the kit to hold both cores) and two core biopsies frozen

immediately at bedside in separate OCT blocks to preserve the proteome and transcriptome of the tumor.

- Tissue may be obtained concurrent with another procedure (clip placement, axillary node FNA) or as a separate procedure. It is permissible to submit samples that were taken prior to study enrollment, such as during the diagnostic biopsy procedure as long as: a) the additional (un-sectioned) frozen biopsies were prepared in cryomolds and OCT at the bedside as described in the kit and held with an accession number in a pathology department at -70C or lower until release to the Alliance WUSTL biorepository and b): the two formalin-fixed biopsies were placed in 10% buffered formalin overnight and processed into paraffin blocks.

7.2 Required 4-week core biopsy for integral Ki67 assay on Arms I, II and III for treatment decision making and correlative studies

- Biopsy at Week 4 is required and need to be collected on day 1 of cycle 2 +/- 3 days
- It is recommended to use the specimen collection/shipping kits provided.
- The needle used to obtain the core biopsies should be 14 G.
- It is strongly suggested that core biopsies be image guided.
- The required biopsies include two core biopsies in 10% buffered formalin and two core biopsies **frozen immediately at bedside** in separate OCT blocks to preserve the proteome and transcriptome of the tumor.

7.3 Required post-treatment core biopsy at time of surgery for all patients after the completion of neoadjuvant endocrine or chemotherapy for correlative studies

- Following completion of neoadjuvant therapy, an intra-operative core biopsy of residual tumor by the surgeon prior to its resection or pre-operative core biopsy (up to 1 week prior to surgery) during a tumor localization procedure (such as radioactive seed placement) is required. If no gross residual tumor can be identified, then a core biopsy of the tumor bed should be done prior to its resection.
- The required biopsies include: Two core biopsies placed in 10% buffered formalin (a single pre-filled formalin container is provided in the kit to hold both cores) and two core biopsies **frozen immediately at bedside** in separate OCT blocks to preserve the proteome and transcriptome of the tumor.
- Alternatively, but not preferred, the site pathologist can remove the tumor samples during the dissection of the surgical specimen (using a 5-mm skin punch biopsy device) as long as the samples are frozen or fixed within 30 minutes of removal from the patient.

7.4 Required fixed tumor tissue slide submission for Ki67 to calculate PEPI and modified PEPI score on Arms I, II and III at time of surgery

- Slide submission for modified PEPI, PEPI, ER and Ki67 testing is required for all patients, in addition to the two core biopsies frozen in separate OCT blocks and two core biopsies in 10% buffered formalin described in Section 7.3.

When the local pathological analysis of tumor samples from the definitive surgical procedure is complete, 10 unstained Superfrost Plus slides cut from the most representative and tumor cell enriched block, and the corresponding pathology report should be submitted to the Alliance WUSTL biorepository. Alternatively, an entire tissue block containing the residual cancer may be submitted and stored at the Alliance WUSTL biorepository. Blocks will be returned to the site within 7 days of a written request, at which time 10 sections and 4 1-mm tissue cores will be taken, prior to return shipment of the block. Further fixed material from the diagnostic biopsy or surgical specimen may be requested by the Alliance WUSTL biorepository at a later date to complete sample analyses, if insufficient tumor is present in the specimens that were previously provided.

The Ki67 value to calculate the modified PEPI score will be provided to the registering physician within 2 weeks of submission of the surgical resection specimen. Patients with a modified PEPI score of 0 will be informed that their outcome is likely to be favorable enough that chemotherapy is unlikely to be of benefit. The use of adjuvant chemotherapy in this patient population will be recorded.

7.5 Optional 12-week core biopsies for Ki67 biomarker assay on Arms I, II and III for treatment decision making and correlative studies

Week 12 sample collection discontinued in Update #07

- The 12-week biopsy is optional and will be performed on day 1 (+/- 3 days) of cycle 4 in patients on Arms I, II and III with a clinical response less optimal (i.e. no tumor shrinkage as judged by treating physician), or in patients whose 4-week biopsy was unsuccessful for Ki67 analysis.
- It is recommended that the specimen collection/shipping kits that are provided be used.
- The needle used to obtain the core biopsies should be 14 G.
- It is strongly suggested that core biopsies be image guided.
- The biopsies include two core biopsies in 10% buffered formalin and two core biopsies frozen immediately at bedside in separate OCT blocks to preserve the proteome and transcriptome of the tumor.

7.6 Optional cycle 1, day 2 core biopsy for patients in the Neoadjuvant Chemotherapy Group on neoadjuvant paclitaxel only for correlative studies

- The biopsies at these two time points are optional, but strongly encouraged for correlative studies of early predictors of paclitaxel efficacy.
- It is recommended to use the specimen collection/shipping kits provided.
- The needle used to obtain the core biopsies should be 14 G.
- It is strongly suggested that core biopsies be image guided.
- The biopsies include two core biopsies placed in 10% buffered formalin (a single pre-filled formalin container is provided in the kit to hold both cores) and two core biopsies **frozen immediately at bedside** in separate OCT blocks to preserve the proteome and transcriptome of the tumor.

7.7 Optional core biopsy at disease progression

Following disease progression, tissue samples are optional and will be acquired in the same way as the on-therapy biopsies from patients who have consented. The optional tumor biopsy at progression will only be collected if the site of progressive disease is to be resected, thereby allowing the tumor tissue to be collected at the time of the resection.

7.8 Neoadjuvant serum, plasma and anti-coagulated whole blood for DNA collection

The anti-coagulated whole blood collection for germline DNA extraction at baseline (prior to study drug administration) is required for all patients consented after Update #06. All other blood samples are optional for patients who have consented.

Week 12 sample collection discontinued in Update #07

Schedule: For patients who have consented, blood for serum, plasma and DNA are collected on the same schedule as the tumor biopsies discussed above including baseline (prior to study drug administration), week 4 (Cycle 2 day 1[+/- 3 days]) and week 12 (Cycle 4 day 1[+/- 3 days]) in Arms I, II and III and Day 2 (Cycle 1 day 1[+ /- 3 days]) in the Neoadjuvant Chemotherapy Group, at the completion of neoadjuvant therapy and prior to off study therapy due to disease progression or toxicity.

Collection: The following peripheral blood samples will be collected by standard venous phlebotomy for patients who have consented (the biopsy kit contains the necessary supplies for the blood sample collection).

- One 10 cc red top Vacutainer tube (or other standard "clot-tube" used for serum chemistry) for serum (provided in the sample collection and shipment kit)
- Two 10 cc Vacutainer tubes containing EDTA (1 for plasma and 1 for DNA) (provided in the sample collection and shipment kit)

Serum and Plasma: After sitting at room temperature for 15 minutes, the clot tube and one of the EDTA tubes should be spun in a standard clinical centrifuge (e.g., ~2500 RPM at 4 °C for 10 minutes). After spinning, serum and plasma should be drawn off from the tube separately into approximately three, 1.5 ml portions and placed into the vials (1.5 ml per vial) provided with the kit. Each vial should be labeled with the study patient ID number. The vials should be placed in a zip-lock bag provided and labeled with the study patient ID number and collection date and time. Vials (serum and plasma) should be frozen and maintained by placing them in an -80 °C freezer until ready for shipment on dry ice. **Do not submerge vials in liquid nitrogen.**

NOTE: Where facilities for centrifugation are not available or logistics prevent sample preparation, the serum and plasma samples can be shipped as whole blood specimens overnight on a wet pack.

Anti-coagulated whole blood for germline DNA extraction: The other EDTA tube should be mixed several times and labeled with the patient's study number, date of birth, and collection date and time. Whole blood specimens are shipped in the specimen kit and must be received by the Alliance WUSTL biorepository **within 48 hours of the time of collection. Do not freeze whole blood.** After being received by the Alliance WUSTL biorepository, anticoagulated blood specimens are spun and the buffy coat isolated. Residual red blood cells are removed by hypotonic lysis. Nucleated cells are washed in phosphate buffered saline, counted, divided into $10x10^6$ cells per aliquot, and spun down. Cell pellets are snap frozen in liquid nitrogen, labeled with the unique specimen ID number, and stored under liquid nitrogen vapor in a locked and alarmed storage inventory unit until they are recalled for DNA extraction.

The end of treatment sample may be collected at the time of surgery to facilitate sample shipping.

7.8.1 Optional follow-up plasma and whole blood in BCT-Streck tubes for ctDNA collection in the adjuvant setting

The blood samples for the collection of ctDNA and other potential circulating biomarkers is optional in the adjuvant setting for all patients following protocol Update #08.

Collection and Shipment: Follow-up plasma and Streck tube blood collection/ship kits are available. At each time point, peripheral blood will be collected in one 10 cc EDTA tube and three 10 cc Streck tubes. Processed frozen plasma and ambient temperature whole blood in Streck tubes will be shipped in the dual chamber shipper in the kit provided.

Plasma processing: After sitting at room temperature for 15 minutes, the EDTA tubes should be spun in a standard clinical centrifuge (e.g., ~2500 RPM at 4 °C for 10 minutes). After spinning, plasma should be drawn off from the tube separately into approximately three, 1.5 ml portions and placed into the vials (1.5 ml per vial) provided with the kit. Each vial should be labeled with the study patient ID number. The vials should be placed in a zip-lock bag provided and labeled with the study patient ID number and collection date and time. Vials with processed plasma should be frozen and maintained by placing them in an -80 °C freezer until ready for shipment on dry ice. **Do not submerge vials in liquid nitrogen.**

Streck tube whole blood processing: Mix by gentle inversion 8 to 10 times immediately after collection, and shipped overnight to Alliance WUSTL biorepository at room temperature in the kit provided. Storing the blood containing Streck tubes at room temperature for a maximum of 3 days following blood collection is allowed if overnight shipment is not possible due to

weekend or holidays. Do not refrigerate or freeze the Streck tube collected blood. Further processing to plasma and ctDNA will be performed at the Alliance WUSTL biorepository.

Optional follow-up research blood (plasma and whole blood in 3 Streck tubes at each time point; kits are available) is collected at the following time points (+/- 6 months acceptable for yrs 1-10 collection):

- All patients: at 5 yrs (+/- 6 months) after surgery and at recurrence (any time prior to the start of new treatment)
- mPEPI ≥ 4 or if switched to neoadjuvant chemotherapy due to Ki67 > 10% at wk 4 or 12: at postop visit (2-8 wks post surgery), yearly for years 1-10 (+/- 6 months) post surgery until recurrence and at recurrence (any time prior to the start of new treatment)

For patients who have already passed the earlier timepoints in their treatment course at the time of Update #08 activation, follow-up plasma and Streck tube blood collection starts at the next immediate timepoint or at the next visit if the missing timepoint was the final timepoint in the schedule.

7.9 Forms submission

The institutional pathology report and any other clinical reports must be submitted using the Medidata Rave® system.

A completed Biomarker Assay Request Form must be submitted with all tumor specimens collected at baseline, 4-week and 12-week time points, for those patients receiving neoadjuvant endocrine therapy. The Biomarker Assay Request Form must also be completed and submitted with the tumor samples collected at the time of surgery (both tumor tissue cores collected at surgery and unstained slides or blocks submitted after surgery) for patients who have completed neoadjuvant endocrine therapy on Arms I, II and III. The form can be accessed when logging specimens in BioMS. Additional instructions for completing this form are provided with the specimen shipping kit. Please print and place the completed Biomarker Assay Request Form for each specimen in the shipping kits with these specimens, when they are shipped to Alliance Biorepository at Washington University (WUSTL). For answers to additional questions on the use or completion of the Biomarker Assay Request Form, please contact the BioMS help desk by phone or email: 1-855-55BIOMS or bioms@alliancenctn.org.

7.10 Specimen registration and tracking

USE OF THE ALLIANCE BIOSPECIMEN MANAGEMENT SYSTEM (BioMS) IS MANDATORY AND ALL SPECIMENS MUST BE LOGGED AND SHIPPED VIA THIS SYSTEM.

BioMS is a web-based system for logging and tracking all biospecimens collected on Alliance trials. Authorized individuals may access BioMS at the following URL: http://bioms.allianceforclinicaltrialsinoncology.org using most standard web browsers (Safari, Firefox, Internet Explorer). For information on using the BioMS system, please refer to the 'Help' links on the BioMS web page to access the on-line user manual, FAQs, and training videos. To report technical problems, such as login issues or application errors, please contact: 1-855-55BIOMS. For assistance in using the application or questions or problems related to specific specimen logging, please contact: 1-855-55BIOMS.

After logging collected specimens in BioMS, the system will create a shipping manifest. This shipping manifest must be printed and placed in the shipment container with the specimens.

7.11 Shipment of samples

- All samples should be labeled with institutional surgical pathology number (for slides and blocks), study patient ID number, patient initials and sample collection date and time and be accompanied by the completed specimen submission shipping manifest as generated by BioMS.
- All samples should be shipped to the Alliance WUSTL biorepository. The same kit used for the sample collection is also used for shipment, with dry ice added in one of the chambers for the frozen samples (OCT-embedded tumor, serum and plasma), which results in a temperature of 15º C in the other chambers for the formalin-fixed samples. **NOTE:** Where facilities for centrifugation are not available or logistics prevent sample preparation, the serum and plasma samples can be shipped as whole blood specimens overnight on a wet pack.
- Specimens may be sent to the Alliance WUSTL biorepository on Monday through Thursday for next day delivery. **The Bank cannot receive specimens on Saturdays, Sundays or holidays. Do not send specimens on Friday or the day before a holiday.**
- Since sample processing for tumor Ki67, ER and other biomarker analysis needs to be performed within 72 hours of sample collection, tumor biopsy or tumor collection at surgery should not be performed on Fridays or the day before a 1 or 2-day holiday or weekend if overnight shipment (also see shipping instruction in section 7.11) is needed for samples to arrive at the Alliance WUSTL biorepository.

  Biopsies or surgery could be performed the day before a 1-day holiday if samples are stored under the conditions specified below and able to be shipped the day after the holiday within 48 hours of tissue collection.

  Tumor tissue storage instruction before shipment:

  * Store frozen cores in OCT at -70C or in dry ice.
  * Store tissue cores in formalin at room temperature.

  Blood samples should be shipped with the tumor samples collected from the same time point of collection. If storage is needed prior to shipment, please following the following instruction:

  * Store processed plasma and serum at -70C or in dry ice.
  * Store Whole blood for DNA in refrigeration.
  * Store Streck tube with whole blood at room temperature.

  Washington University and Alliance WUSTL biorepository holidays:

New Year's Day	January 1st
Martin Luther King Day	The 3rd Monday in January
Memorial Day	The 4th Monday in May
4th of July	July 4th
Labor Day	The first Monday in September
Thanksgiving and Day After	The 4th Thursday and Friday of November
Christmas Eve and Day	December 24 and 25th
New Year’s Eve	December 31

- The institution is expected to pay the cost of shipping specimens and will be reimbursed through capitation fees set for each individual study.
- Arrange for Federal Express pick-up through your usual institutional procedure. Ship specimens to the address below:

Mark A. Watson, M.D., Ph.D.
Alliance Biorepository at Washington University
425 S. Euclid Ave, Room 5120
St. Louis, MO 63110-1005
Phone: (314) 454-7615
Fax: (314) 454-5525
E-mail: tbank@wudosis.wustl.edu

7.12 Funding for research biopsy procedures

Information regarding funding of the study research biopsy procedures can be found on the A011106 funding sheet that is available on the A011106 study page of the CTSU website.

8.0 TREATMENT

Protocol treatment is to begin within 14 days of registration/randomization.

8.1 Neoadjuvant therapy:

Arm I: Anastrozole

Agent	Dose and Route	Day	Schedule
**Anastrozole**	1 mg daily by mouth	Days 1-28	Every 4 weeks x 6 cycles*

Arm II: Fulvestrant

Agent	Dose and Route	Day	Schedule
**Fulvestrant**	500 mg IM	Days 1 and 15 of Cycle 1 only.<br>Day 1 of cycles 2-6.	Every 4 weeks x 6 cycles*

Arm III: Anastrozole + Fulvestrant

Agent	Dose and Route	Day	Schedule
**Anastrozole**	1 mg daily by mouth	Days 1-28	Every 4 weeks x 6 cycles*
**Fulvestrant**	500 mg IM	Days 1 and 15 of Cycle 1 only.<br>Day 1 of cycles 2-6.	Every 4 weeks x 6 cycles*

*For Arms I, II, or III: Each cycle is 28 days [+/- 3 days] (or 4 weeks).
Surgery must be performed between days 7-28 of Cycle 6 in Arms I, II and III.

8.1.1 Neoadjuvant Chemotherapy Group:

Patients who are determined to have Ki67 > 10% at either the 4-week or the 12-week time point will discontinue the endocrine protocol therapy. It is recommended that patients be switched to neoadjuvant paclitaxel (preferred) using the recommended regimen below, or another taxane and/or anthracycline or CMF regimen per NCCN Guidelines. The cycle length of the non-paclitaxel chemotherapy is specified by the NCCN Guidelines. If, at the discretion of the treating physician, the patient would not be a candidate for paclitaxel treatment or the other chemotherapy regimens, the patient may proceed to surgery.

Agent	Dose and Route	Day	Schedule
**Paclitaxel**	80 mg/m2 in 250 mL in D5W or NS IV infusion over 1 hour in a non-PVC container and through a polyethylene lined set.	Days 1, 8, 15 and 22.	Every 4 weeks x 3 cycles

Premedication regimen prior to paclitaxel infusion should be administered per institutional standard of care.

8.2 Neoadjuvant treatment evaluation and decision tree

If a patient who has registered on study has been found not to have fulfilled all of the eligibility requirements, after starting protocol therapy, may continue on study per protocol if she is deriving benefit. Otherwise, all online data submission, up to the point of study treatment discontinuation should be submitted and the patient will continue to be followed per Section 9.0 of the protocol.

If a patient refuses to continue protocol treatment, develops intolerable toxicity, or wishes to receive alternative therapy, the patient will continue to be followed per Section 9.0 of the protocol.

8.2.1 During neoadjuvant endocrine therapy phase (Arms I, II and III)

Schedule of evaluations:

- Tumor (breast and axilla) should be clinically assessed by measuring tape, ruler or caliper prior to the start, every four weeks (+/- 3 days) during, and at the discontinuation of neo-adjuvant endocrine therapy. Bi-dimensional measurements should be obtained using the same technique (measuring tape, ruler or caliper) and if possible, by same person, as at each evaluation.
- Mammogram and ultrasound of the diseased breast at baseline and completion of neoadjuvant therapy.

Treatment/follow-up decision tree during neoadjuvant treatment:

- ***If a patient is found to have a 4 week Ki67 > 10%***, it is recommended that the patient be switched to neoadjuvant chemotherapy.

  - If the patient chooses to receive neoadjuvant chemotherapy, then the patient should be followed as outlined in the test schedule found in Section 5.3.2.

  - If the patient refuses to switch to neoadjuvant chemotherapy, or is not a candidate for neoadjuvant chemotherapy, decides to start neoadjuvant treatment with another regimen, or decides to undergo immediate surgery, submit on-line data indicating whether the patient will go immediately to surgery or begin other anti-neoplastic approaches. These patients will go to the survival and disease status follow-up period where further treatment is at the discretion of her treating physician.

Week 12 sample collection discontinued in Update #07

- ***If a 4 week Ki67 value cannot be obtained due to lack of sufficient tumor in the biopsy specimen,*** the patient may either immediately be re-biopsied or continue study therapy per protocol at the discretion of her treating physician. If the second biopsy does not provide sufficient tumor to determine Ki67, the patient may continue on study therapy at the discretion of her treating physician, or enter the survival and disease status follow-up period where further treatment is at the discretion of her treating physician.

  - Note that tissue collection/shipment for the repeat biopsy should follow the same procedure as that for the 4-week biopsy. The specimen will be registered to BioMS as an independent (or recollected) specimen from the 1st submission at the 4-week time point.

- ***If there is no evidence of clinical progression, during neoadjuvant treatment,*** the patient should continue to follow protocol procedures.
- ***If there is physical evidence for clinical progression with bi-dimensional measurements of the primary tumor by measuring tape, ruler or caliper,*** a mammogram and/or ultrasound of the breast should be done to confirm/rule out progressive disease. If there is physical evidence for clinical progression with clinical assessment of the lymph node mass, an ultrasound of the axilla should be done to confirm/rule out progressive disease.

1. If progression is ***confirmed*** by ultrasound **OR** mammographic imaging, treatment is at the discretion of the treating physician, operate as soon as possible or begin other anti-neoplastic approaches such as chemotherapy or radiation therapy. **NOTE:** Other anti-

neoplastic approaches such as chemotherapy or radiation must not be administered while the patient is taking study drug.

a. If immediate surgery is the decision, study drug should be continued until the day before surgery and the core biopsies (two frozen in OCT and two formalin fixed) from all patients and the optional blood samples from consented patients should be collected at the time of surgery.

b. If other anti-neoplastic approaches such as chemotherapy or radiation therapy are to be administered, all study drugs must be discontinued before alternate therapy begins.

Submit on-line data indicating whether the patient will go to immediate surgery or begin other anti-neoplastic approaches such as chemotherapy or radiation therapy. The patients will go to the survival and disease follow-up period of the trial per Section 9.0 of the protocol.

2. If disease progression ***is not confirmed*** by ultrasound or mammographic imaging, study drug may be continued at the investigator's discretion, and the protocol followed as described in Section 5.2.

    a. If immediate surgery is the decision, study drug should be continued until the day before surgery and the core biopsies (two frozen in OCT and two formalin fixed) from all patients and the optional blood samples from consented patients should be collected at the time of surgery.

    b. If other anti-neoplastic approaches such as chemotherapy or radiation therapy are to be administered, all study drugs must be discontinued before alternate therapy begins.

If the study drug is to be discontinued, submit on-line data indicating whether the patient will immediately go to surgery or begin other anti-neoplastic approaches such a chemotherapy or radiation therapy. These patients will go to survival and disease status follow-up period of the trial per Section 9.0 of the protocol.

- ***If there is suspicion of clinical progression outside the primary site*** (i.e., the development of a new breast mass or the development of clinical suspicion for advanced disease or suspicion of second primary cancer), imaging or biopsy should be done to confirm.

1. If confirmed, study drug will be discontinued and subsequent management is at the investigator's discretion. Submit on-line data indicating whether the patient will go immediately to surgery or begin other anti-neoplastic approaches such as chemotherapy or radiation therapy. These patients will go to survival and disease status follow-up period of the trial per Section 9.0 of the protocol.

2. If not confirmed by imaging or biopsy, study drug may be continued at the investigator's discretion, and the protocol followed as described in Section 5.2.

    a. If immediate surgery is the decision, study drug should be continued until the day before surgery and the core biopsies (two frozen in OCT and two formalin fixed) from all patients and the optional blood samples from consented patients should be collected at the time of surgery.

    b. If other anti-neoplastic approaches such as chemotherapy or radiation therapy are to be administered, all study drugs must be discontinued before alternate therapy begins.

If the study drug is to be discontinued, submit on-line data indicating whether the patient will immediately go to surgery or begin other anti-neoplastic approaches such a chemotherapy or radiation therapy. These patients will go to survival and disease status follow-up period of the trial per Section 9.0 of the protocol.

8.2.2 Schedule of evaluations for patients switching to neoadjuvant chemotherapy (Neoadjuvant Chemotherapy Group):

- Tumor (breast and axilla) should be clinically assessed by measure tape, ruler or caliper prior to the start of neoadjuvant chemotherapy (but after completion of endocrine therapy) and every day 1 of each cycle (+/- 3 days) during and at the discontinuation of neo-adjuvant chemotherapy therapy. Bi-dimensional measurements should be obtained using the same technique (measuring tape, ruler or caliper) and if possible, by same person, as at each evaluation.
- Mammogram and ultrasound of the diseased breast at completion of neoadjuvant therapy.

Treatment/follow-up decision tree:

- If no evidence of clinical progression, continue to follow protocol procedures.
- ***If there is physical evidence for clinical progression*** with bi-dimensional measuring tape, ruler or caliper tumor measurements of the primary tumor the mammogram and/or ultrasound of the breast should be done to confirm/rule out progressive disease. If there is physical evidence for clinical progression with clinical assessment of the lymph node mass, an ultrasound of the axilla should be done to confirm/rule out progressive disease.

1) ***If progression is confirmed by ultrasound OR mammographic imaging***, treatment is at the investigator's discretion, operate as soon as possible or begin other anti-neoplastic approaches such as chemotherapy or radiation therapy. **NOTE**: Other anti-neoplastic approaches such as chemotherapy or radiation must not be administered while the patient is taking study drug.

   i Discontinue neoadjuvant chemotherapy.

   ii Obtain blood and core biopsies of tumor tissue (two frozen in OCT and two formalin-fixed) at the time of surgery, if immediate surgery is the decision.

   iii Obtain optional blood and core biopsies of tumor tissue (two frozen in OCT and two formalin-fixed) before the patient receives non-protocol chemotherapy.

   iv Submit on-line data indicating whether the patient will go to immediate surgery or begin other anti-neoplastic approaches such as chemotherapy or radiation therapy. The patient will continue to be followed per Section 9.0 of the protocol.

2) ***If disease progression is not confirmed by ultrasound or mammographic imaging***, study drug may be continued at the investigator's discretion, and the protocol should be followed as described in Section 9.0. If the study drug is to be discontinued, submit the on-line data indicating whether the patient will immediately go to surgery or begin other anti-neoplastic approaches such as chemotherapy or radiation therapy. The patient will go to the ***Survival and disease status follow-up period*** per Section 9.0 of the protocol.

- ***If suspicion of progression outside the primary site*** (i.e., the development of a new breast mass, development of clinical suspicion for advanced disease or suspicion of second primary cancer) should lead to further imaging evaluation and if confirmed, study drug will be discontinued after correlative samples of the primary tumor have been obtained. Subsequent management is at the investigator's discretion. Submit on-line data indicating whether the patient will immediately go to surgery or begin other anti-neoplastic approaches such as chemotherapy or radiation therapy. The patient will go to the ***Survival and disease status follow-up period, per Section 9.0 of the protocol.***

8.3 Dose modifications, and management of toxicity

8.3.1 Anastrozole

There will be no anastrozole dose reductions. Anastrozole will be held for > grade 3 hepatic function impairment. Recheck liver function tests in one week. Resume anastrozole at the previous dose when hepatic toxicity resolves to < grade 2. If anastrozole is held for > 3 weeks, permanently discontinue anastrozole therapy.

If unanticipated grade 3 or 4 toxicity is encountered, that is considered at least possibly related to anastrozole, the patient will be discontinued from anastrozole therapy.

In patients who cannot swallow, anastrozole tablet can be dispersed in 10 ml of water to give an almost clear dispersion (agitating to aid dispersion) that flushes down an 8Fr NG tube without blockage. The tablets are slow to disperse and take in excess of 5 minutes) (BPNG data on file 2004). Standard precautions apply. Crushing is not recommended.

Missed doses of anastrozole are not made up.

Note: Serious adverse events will be reported through CTEP-AERS using CTCAE v5.0.

8.3.2 Fulvestrant

There are no planned dose modifications for fulvestrant. If unanticipated grade 3 or 4 toxicity is encountered, that is considered at least possibly related to fulvestrant, the patient will be discontinued from fulvestrant therapy.

Patients who need more than a two-week delay from fulvestrant therapy should discontinue study therapy due to toxicity and continue to be followed per Section 9.0 of the protocol.

Note: Serious adverse events will be reported through CTEP-AERS using CTCAE v5.0.

8.3.3 Paclitaxel or other chemotherapy

The management of toxicities and dose modifications is per standard of care of the treating physician.

Note: Serious adverse events will be reported through CTEP-AERS using CTCAE v5.0.

8.4 Surgery

Schedule: Surgery should be scheduled during the last 3 weeks (days 7 to 28) of the 6th cycle of neoadjuvant endocrine therapy (Arms I, II and III) or 3-6 weeks following the last dose of neoadjuvant paclitaxel or other NCCN regimen (Neoadjuvant Chemotherapy Group). Patients who refuse to undergo surgery or are unable to undergo surgery due to co-morbid conditions will go to the survival and disease status follow-up period of the trial per Section 9.0 of the protocol. Further treatment is at the discretion of the patient's physician.

Note: A surgical delay beyond the time frame described above, requires a discussion with the principal investigator or co-investigator. Given the patient population (older with co-morbid illnesses) it may be appropriate for a patient to remain on the endocrine agent in Arms I, II and III and allow a delay in surgery to allow a recovery from inter-current illness for example. These issues will be dealt with on a case-by-case basis.

Breast surgery: The type of breast surgery (either mastectomy or lumpectomy) is determined by the treating surgeon according to institutional standard, with the goal to completely excise the tumor. A negative margin is required unless further excision is not possible.

The actual surgical/therapeutic approaches taken will be recorded as:

- Partial Mastectomy at first attempt (removal of the cancer as well as some of the breast tissue around the tumor and the fascia over the chest muscles below the tumor, if indicated).
- Re-excision after Partial Mastectomy as first attempt
- Total Mastectomy after Partial Mastectomy as first attempt
- Modified Radical Mastectomy (record preservation or removal of pectoralis minor)
- Radical Mastectomy (does not include resection of less than 25% of pectoralis major)
- Remained inoperable and received chemotherapy
- Progressive disease and received chemotherapy
- Other (specify)

Lymph node surgery: The type of lymph node surgery (sentinel lymph node with or without axillary lymph node dissection) will be determined by the treating surgeon according to institutional standard.

It is encouraged but, not required that surgeons have completed the standard training for performing a sentinel lymph node dissection. At the investigator's discretion, SLND and backup Level I and II dissection may be performed in conjunction with a mastectomy to determine the accuracy of the sentinel node procedure after neoadjuvant therapy. SLND may be performed with isosulfan blue dye, a radiopharmaceutical, or combination of both. Accurate axillary staging is important for this study since PEPI score and residual cancer burden is the primary end points of the study and all patients should undergo post-treatment axillary node staging.

Surgical pathology report: Size of the breast tumor present at start of surgery; number of lymph nodes examined and the number of positive by H&E staining; final tumor margins; and extent of surgery should be reported with submission of surgical and pathology reports.

Residual Cancer Burden (RCB) report (Neoadjuvant Chemotherapy Group): The largest two dimension (mms) of the residual tumor bed in the breast; histologic assessment of the percentage of the overall cancer (including invasive and in situ) in the tumor bed; histologic assessment of the percentage of the carcinoma that is in situ; the number of positive lymph nodes; and the diameter of the largest nodal metastasis should be reported, in addition to the calculated Residual Cancer Burden with the submission surgical and pathology reports. The Residual Cancer Burden Calculator and detailed description of reporting can be found at the following web site: http://www3.mdanderson.org/app/medcalc/index.cfm?pagename=jsconvert3.

Required tissue sampling at surgery: Two core biopsies frozen in separate OCT blocks and two formalin-fixed core biopsies should be obtained (preferred). Tumor samples may also be removed by dissection of the surgical specimen (not preferred) as long as the samples are frozen or fixed within 30 minutes of removal from the patient (see Section 7.3). In addition, slides for PEPI score will be collected from fixed surgical pathology tumor tissue blocks (see Section 7.4).

8.5 Postoperative chemotherapy

8.5.1 Arms I, II or III

8.5.1.1 Modified PEPI 0 Group: Adjuvant chemotherapy is not recommended. Participation in other adjuvant trials of pharmaceutical interventions is not allowed.

8.5.1.2 Modified PEPI Non-0 Group: Adjuvant treatment is at the discretion of treating physician and will be documented by online data submission. Patient may participate in other adjuvant clinical trials.

8.5.2 Neoadjuvant Chemotherapy Group

8.5.2.2 Additional chemotherapy such as an anthracycline based regimen is administered at the discretion of treating physician. Patient may participate in other adjuvant clinical trials.

8.6 Postoperative endocrine therapy

In patients who received adjuvant chemotherapy, adjuvant endocrine therapy is to be administered within 2-8 weeks at the completion of adjuvant chemotherapy based on evaluation of chemotherapy related toxicities.

In patients for whom adjuvant chemotherapy is not planned, adjuvant endocrine therapy is to be administered within 2-8 weeks postoperatively.

8.6.1 Arm I:

- **Modified PEPI 0 Group:** Adjuvant anastrozole 1 mg PO daily for 54 cycles (approximately 4.5 years). Standard of care endocrine therapy beyond 5 years of protocol therapy is per physician discretion. Patient may ***not*** participate in other adjuvant trials of pharmaceutical interventions.
- **Modified PEPI Non-0 Group:** Adjuvant hormonal therapy of treating physician's choice. Patient may participate in other adjuvant clinical trials.

8.6.2 Arm II:

- Modified PEPI 0 Group: Adjuvant fulvestrant 500 mg IM day 1 for 18 cycles followed by anastrozole 1 mg PO daily for 36 cycles (approximately 3 years). If the last dose of neoadjuvant fulvestrant was administered more than 4 weeks prior to beginning adjuvant fulvestrant, a reload with fulvestrant (500mg IM on days 1 and 15) during the first cycle is needed. Standard of care endocrine therapy beyond 5 years of protocol therapy is per physician discretion.

  Patient may ***not*** participate in other adjuvant trials of pharmaceutical interventions.
- Modified PEPI Non-0 Group: Adjuvant hormonal therapy of treating physician's choice.

  Patient may participate in other adjuvant clinical trials.

8.6.3 Arm III:

- Modified PEPI 0 Group: Adjuvant anastrozole 1 mg PO daily in combination with fulvestrant 500 mg IM day 1 for 18 cycles, followed by anastrozole 1 mg PO daily for 36 cycles (approximately 3 years). If the last dose of neoadjuvant fulvestrant was administered more than 4 weeks prior to beginning adjuvant fulvestrant, a reload with fulvestrant (500mg IM on days 1 and 15) during the first cycle is needed. Standard of care endocrine therapy beyond 5 years of protocol therapy is per physician discretion.

  Patient may not participate in other adjuvant trials of pharmaceutical interventions.
- Modified PEPI Non-0 Group: Adjuvant hormonal therapy of treating physician's choice.

  Patient may participate in other adjuvant clinical trials.

8.6.4 Neoadjuvant Chemotherapy Group: Standard adjuvant hormonal therapy such as anastrozole is recommended. Patient may participate in other adjuvant clinical trials.

8.7 Adjuvant radiation therapy

- **The administration of adjuvant radiation to the breast and draining lymph nodes is according** to institutional standard.
- Whole breast radiation rather than partial breast or brachytherapy, is recommended for patients where adjuvant radiation is indicated.

8.8 Study drug pharmacology

8.8.1 Anastrozole:

Please refer to the FDA-approved package insert for anastrozole for product information and a comprehensive list of adverse events.

Chemical Name or Amino Acid Sequence: 1,3-Benzenediacetonitrile, a, a, a', a'-tetramethyl-5-(1H-1,2,4-triazol-1-ylmethyl)

Other Names: Arimidex

Classification: Non-steroidal aromatase inhibitor

Molecular Formula: $C_{17}H_{19}N5$ M.W.: 293.4

Approximate Solubility: Anastrozole has moderate aqueous solubility (0.5 mg/mL at 25°C); solubility is independent of pH in the physiological range. Anastrozole is freely soluble in methanol, acetone, ethanol, and tetrahydrofuran, and very soluble in acetonitrile.

Mode of Action: In postmenopausal women, the principal source of circulating estrogen (primarily estradiol) is conversion of adrenally-generated androstenedione to estrone by aromatase in peripheral tissues, such as adipose tissue, with further conversion of estrone to estradiol. Many breast cancers also contain aromatase; the importance of tumor-generated estrogens is uncertain.

Anastrozole is a potent and selective non-steroidal aromatase inhibitor. It significantly lowers serum estradiol concentrations and has no detectable effect on formation of adrenal corticosteroids or aldosterone (AstraZeneca, Package Insert).

Pharmacokinetics

Inhibition of aromatase activity is primarily due to anastrozole, the parent drug. Studies with radiolabeled drug have demonstrated that orally administered anastrozole is well absorbed into the systemic circulation with 83 to 85% of the radiolabel recovered in urine and feces. Food does not affect the extent of absorption. Elimination of anastrozole is primarily via hepatic metabolism (approximately 85%) and to a lesser extent, renal excretion (approximately 11%), and anastrozole has a mean terminal elimination half-life of approximately 50 hours in postmenopausal women. The major circulating metabolite of anastrozole, triazole, lacks pharmacologic activity. The pharmacokinetic parameters are similar in patients and in healthy postmenopausal volunteers. The pharmacokinetics of anastrozole are linear over the dose range of 1 to 20 mg and do not change with repeated dosing. Consistent with the approximately 2-day terminal elimination half-life, plasma concentrations approach steady-state levels at about 7 days of once daily dosing and steady-state levels are approximately three to four-fold higher than levels observed after a single dose of Anastrozole. Anastrozole is 40% bound to plasma proteins in the therapeutic range [AstraZeneca, Package Insert].

Pharmacodynamics

Effect on Estradiol: Mean serum concentrations of estradiol were evaluated in multiple daily dosing trials with 0.5, 1, 3, 5, and 10 mg of Anastrozole in postmenopausal women with advanced breast cancer. Clinically significant suppression of serum estradiol was seen with all doses. Doses of 1 mg and higher resulted in suppression of mean serum concentrations of estradiol to the lower limit of detection (3.7 pmol/L). The recommended daily dose, Anastrozole 1 mg, reduced estradiol by approximately 70% within 24 hours and by approximately 80% after 14 days of daily dosing. Suppression of serum estradiol was maintained for up to 6 days after cessation of daily dosing with Anastrozole 1 mg.

Effect on Corticosteroids: In multiple daily dosing trials with 3, 5, and 10 mg, the selectivity of anastrozole was assessed by examining effects on corticosteroid synthesis. For all doses,

anastrozole did not affect cortisol or aldosterone secretion at baseline or in response to ACTH. No glucocorticoid or mineralocorticoid replacement therapy is necessary with anastrozole.

Other Endocrine Effects: In multiple daily dosing trials with 5 and 10 mg, thyroid stimulating hormone (TSH) was measured; there was no increase in TSH during the administration of Anastrozole. Anastrozole does not possess direct progestogenic, androgenic, or estrogenic activity in animals, but does perturb the circulating levels of progesterone, androgens, and estrogens (AstraZeneca, Package Insert).

Metabolism and Excretion

Studies in postmenopausal women demonstrated that anastrozole is extensively metabolized with about 10% of the dose excreted in the urine as unchanged drug within 72 hours of dosing, and the remainder (about 60% of the dose) is excreted in urine as metabolites. Metabolism of anastrozole occurs by N-dealkylation, hydroxylation and glucuronidation. Three metabolites of anastrozole have been identified in human plasma and urine. The known metabolites are triazole, a glucuronide conjugate of hydroxy-anastrozole, and a glucuronide of anastrozole itself. Several minor (less than 5% of the radioactive dose) metabolites have not been identified. Because renal elimination is not a significant pathway of elimination, total body clearance of anastrozole is unchanged even in severe (creatinine clearance less than 30 mL/min/1.73m2) renal impairment, dosing adjustment in patients with renal dysfunction is not necessary. Dosage adjustment is also unnecessary in patients with stable hepatic cirrhosis (AstraZeneca, Package Insert).

How Supplied: Anastrozole tablets for oral administration contain 1 mg of anastrozole.

Anastrozole is an off-white powder. Each tablet contains as inactive ingredients: lactose, magnesium stearate hydroxypropylmethylcellulose, polyethylene glycol, povidone, sodium starch glycolate, and titanium dioxide [AstraZeneca, Package Insert].

Storage: Store at controlled room temperature at 20 - 25°C.

Route(s) of Administration: Oral

Method of Administration: Take anastrozole with or without food.

Availability: Anastrozole is commercially available.

Toxicity: Hot flashes, asthenia, arthritis, pain, pharyngitis, HTN, depression, N/V, rash, osteoporosis, fractures, headache, bone pain, peripheral edema, dyspnea.

Potential Drug Interactions: Anastrozole is generally safe to administer with other medicines. However, concomitant use of agents and herbal products that alter ER function are specifically not allowed.

8.8.2 Fulvestrant

Please refer to the FDA-approved package insert for fulvestrant for product information, extensive preparation instructions and a comprehensive list of adverse events.

Chemical Name or Amino Acid Sequence: 7-alpha-[9-(4,4,5,5,5-penta fluoropentylsulphinyl) nonyl]estra-1,3,5-(10)-triene-3,17-beta-diol.

Other names: Faslodex

Classification: Estrogen receptor antagonist

Molecular Formula: $C_{32}H_{47}F_5O_3S$ M.W.: 606.77

Approximate Solubility: Each injection contains as inactive ingredients alcohol, USP, benzyl alcohol, NF, and benzyl benzoate, USP as co-solvents and castor oil, USP as a co-solvent and release rate modifier.

Mode of Action: Fulvestrant is a competitive estrogen receptor antagonist. Fulvestrant binds to the ER with an affinity that is comparable to estradiol and downregulate ER expression, resulting in inhibition of the transcription of ER-regulated genes [52]. In addition, the down-regulation of ER was associated with a dose-related decrease in the expression of the progesterone receptor, an estrogen-regulated protein. These effects on ER were also associated with a decrease in cell proliferation (decreased Ki67 labeling index). Fulvestrant has no agonist-type effect. In animal studies, fulvestrant showed no induction of transcription, and when it was administered prior to estradiol or tamoxifen, it completely blocked estrogen or tamoxifen gene induction (e.g., induction of calbindin-D, insulin-like growth factor, vascular endothelial cell growth factor, and c-fos) [52]. In postmenopausal women, fulvestrant administration has no effect on the concentrations of FSH and LH, suggesting no peripheral steroidal effects. In postmenopausal women treated with single doses of fulvestrant 15 - 22 days prior to surgery, there was evidence of increasing down-regulation of ER with increasing dose.

Pharmacokinetics [53, 54]

Following intramuscular injection, fulvestrant 500 mg is absorbed slowly, with a mean maximum plasma concentration (Cmax) reached in approximately 5 days. At steady-state, plasma concentrations of fulvestrant 500 mg are maintained over a 28-day period, with a minimum plasma concentration of 12.2 ng/mL, a Cmax of 28.0 ng/mL and an area under the concentration-time curve (AUC) of 545.8 ng d/mL at month 3. Systemic exposure is approximately dose-proportional over a dose range of 50 – 500mg and steady-state concentrations are reached within approximately 1 month. Following intramuscular injection, fulvestrant has a long terminal elimination half-life of 50 days for the 500 mg regimen.

Fulvestrant distributes with an apparent volume of approximately 3–5 L/kg at steady state. Plasma protein binding of fulvestrant is high (99%) with low-density lipoprotein, very low-density lipoprotein and high-density lipoprotein the main binding components. Fulvestrant is metabolized by cytochrome P450 (CYP) 3A4 and not other CYP isoenzymes, as determined using human liver preparations and recombinant human enzymes in vivo. The relative contribution of CYP and non-CYP pathways of metabolism in vivo is unclear. Fulvestrant is predominantly (approximately 90%) eliminated as metabolites in the feces, with <1% excreted in the urine.

There are no known pharmacokinetic interactions between fulvestrant and other drugs. In particular, co-administration of fulvestrant with ketoconazole (a CYP3A4 inhibitor) or rifampicin (rifampin) [a CYP3A4 inducer] does not affect the pharmacokinetic profile of fulvestrant. Similarly, co-administration of fulvestrant with midazolam, which is metabolized by CYP3A4, does not affect the pharmacokinetic profile of midazolam. Consequently, dosage adjustments of fulvestrant are not required when co-administered with inhibitors or inducers of CYP3A4.

Pharmacodynamics

Fulvestrant inhibits tumor cell proliferation mainly through down regulation of ER expression as demonstrated in models of estrogen-dependent breast cell lines in vitro and xenografts in vivo [55]. The dose dependent anti-proliferative effect on tumor cells has been confirmed in the clinical setting using both the Ki67 labeling index (LI; a measure of cell proliferation) [22, 36] and cell turnover index (a composite measure of proliferation and apoptosis) [37] in ER+ breast cancers in postmenopausal women. In the NEWEST trial, mean Ki67 LI and ER at week 4 (primary endpoint) was reduced from baseline by a significantly greater extent with the fulvestrant 500 mg regimen than with fulvestrant 250 mg monthly (Ki67 LI: 78.8% vs 47.3%; $p < 0.0001$; ER: 22% vs 15%; $p<0.0003$) [36].

Fulvestrant has no estrogenic effect on the endometrium of healthy postmenopausal women in a randomized, double-blind phase I study [55]. No clinically significant changes in serum bone-specific turnover markers were observed in 14 patients with advanced breast cancer following 18 months' fulvestrant 250 mg monthly [56] and no clinically significant changes were observed with either the 250 mg monthly or 500 mg regimen at week 16 of the NEWEST trial [36].

Fulvestrant in the treatment of breast cancer

The clinical effectiveness of fulvestrant as a treatment for advanced breast cancer has previously been demonstrated in several phase III clinical trials [33, 34, 57]. In the EFECT trial, fulvestrant was found to be at least as effective as exemestane patients with tumors that are resistant to non-steroidal AIs [34]. Fulvestrant is therefore indicated as a second line therapy for patients who progressed on an AI in advanced disease setting. Recent studies indicate high-dose fulvestrant at 500 mg monthly is more effective compared to the standard dosing of 250 mg monthly [37], which prompted FDA’s recent approval of high-dose fulvestrant for refractory metastatic breast cancer [58]. Based on the encouraging data obtained in the metastatic setting as discussed in the Background section, result of the ALTERNATE trial will provide biological evidence for evaluation of fulvestrant in the adjuvant setting.

Description: Estrogen receptor antagonist

How Supplied: Fulvestrant injection is provided by AstraZeneca as a sterile single-patient pre-filled syringe containing 250 mg at a concentration of 50 mg/ml. The solution is a clear, colorless to yellow, viscous liquid.

Storage: The syringes of fulvestrant for all cycles of treatment should be stored in the original container and refrigerated at 2°- 8°C (36°- 46°F).

Routes of Administration: IM injection

Method of Administration: Remove glass syringe barrel from tray and check that it is not damaged. Peel open the safety needle (SafetyGlide™) outer packaging. Break the seal of the white plastic cover on the syringe luer connector to remove the cover with the attached rubber tip cap. Twist to lock the needle to the luer connector. Remove needle sheath. Remove excess air from the syringe (a small gas bubble may remain).

For this study, fulvestrant will be administered at a dose of 500 mg (2 X 250 mg injections) IM on Day 1 and Day 15 of Cycle 1 and then on Day 1 of each cycle in each subsequent cycle. Administer intramuscularly slowly in the buttock. (NOTE: 500 mg dose will require one 250 mg injection in each buttock.) Immediately activate needle protection device upon withdrawal from patient by pushing lever arm completely forward until needle tip is fully covered. Visually confirm that the lever arm has fully advanced and the needle tip is covered. If unable to activate, discard immediately into an approved sharps container.

Availability: Fulvestrant will be provided by AstraZeneca and distributed by McKesson. The Fulvestrant Drug Request Form is available on the A011106 study page of the CTSU website.

The FDA has determined that fulvestrant is IND exempt for this study.

Toxicity: The most common toxicities reported in trials of fulvestrant to date include gastrointestinal symptoms, menopausal symptoms and injection site reactions. Nausea and vomiting have been the primary GI symptoms, occurring in approximately 50% of patients in phase III trials. They were generally of mild to moderate severity. Hot flashes were reported in approximately 20% of patients. Injection site reactions include pain, hemorrhage and inflammation. Such reactions may be more frequent when fulvestrant is administered as 2 injections of 2.5 ml (125 mg) each, as compared with 1 injection of 5 ml (250 mg).

Potential Drug Interactions: In vitro studies using human hepatocytes, fulvestrant was metabolized predominantly by conjugation. The metabolites thus formed are thought to possess no estrogenic activity and minimal anti-estrogenic activity. In studies using human liver microsomes, fulvestrant inhibited the activity of CYP1A2, 2C9 and 3A4 minimally. CYP3A4 did metabolize fulvestrant in these studies, but the human hepatocyte studies noted above indicate conjugation is a more important metabolic pathway. In addition, studies in healthy volunteers indicate that fulvestrant metabolism is not significantly affected by inducers or inhibitors of CYP3A4, nor does fulvestrant affect the metabolism of 3A4 substrates. Thus, fulvestrant is not expected to be involved in significant drug interactions mediated by CYP3A4.

8.8.3 Paclitaxel

Please refer to the FDA-approved package insert for paclitaxel for product information, extensive preparation instructions and a comprehensive list of adverse events.

Chemical Name or Amino Acid Sequence: (2α,4α,5β,7β,10β,13α)-4,10-bis(acetyloxy)-13-{[(2R,3S)- 3-(benzoylamino)-2-hydroxy-3-phenylpropanoyl]oxy}- 1,7-dihydroxy-9-oxo-5,20-epoxytax-11-en-2-yl benzoate

Other Names: Taxol

Classification: Novel antimicrotubule agent

Molecular Formula: $C_{47}H_{51}NO_{14}$ M.W.: 853.9

Approximate Solubility: It is highly lipophilic, insoluble in water

Description

Paclitaxel is a semisynthetic antineoplastic agent. It is a diterpenoid taxane derivative from the bark of the Pacific yew tree, Taxus brevifolia. Paclitaxel was first discovered in 1971 as a result of the National Cancer Institute (NCI) screening program for natural cytotoxic products. Paclitaxel injection is very insoluble in water and requires formulation with Cremophor EL and ethanol. In 1991, an agreement between Bristol-Myers Squibb and the NCI led to the development alternative sources of paclitaxel. Paclitaxel has subsequently been isolated from other members of the Taxus genus and is produced by Taxomyces andreannae, a fungal endophyte isolated from the inner bark of the Pacific yew. The production of a semisynthetic form of paclitaxel using the precursor 10-deacetyl-baccatin III, which is found in the needles of the European yew, Taxus baccata, has allowed large supplies to be produced. Due to its unique mechanism of action, paclitaxel has been studied and subsequently FDA approved as a single agent and in combination with other chemotherapy agents in the treatment of many solid tumors including ovarian, breast, lung and head and neck cancers.

Mechanism of Action

Paclitaxel is an antimicrotubule chemotherapy agent. Paclitaxel promotes the assembly of microtubules and stabilizes their formation by inhibiting depolymerization, arresting the cell cycle in mitosis. Paclitaxel also inhibits the transition from G0 to S phase by disrupting tubulin in the cell membrane and/or direct inhibition of the disassembly of the cytoskeleton interrupting intracellular transport and communications. Cells treated with paclitaxel show distinctive morphologic effects. Multiple bundles of microtubules are noted in paclitaxel treated cells. Abnormal spindle asters are formed during mitosis. Paclitaxel also induces the expression of tumor necrosis factor-alpha and inhibits angiogenesis, although the exact roles of these actions in the cytotoxic effects of paclitaxel is not known.

Pharmacokinetics

Paclitaxel is given by IV administration. Paclitaxel undergoes nonlinear pharmacokinetics due to saturable distribution and/or metabolism. Clinical implications of the nonlinear pharmacokinetics include disproportionate increases in AUC, peak plasma concentrations, and toxicity with dose increases, while dose reductions may lead to decreased cytotoxicity. Neutropenia and, to a lesser extent, neurotoxicity have been associated with exposure of cells above a critical plasma concentration (> 0.05 micromolar/L) or increased duration of exposure and do not correlate to dosage. Paclitaxel is extensively protein bound (95—98%) to tissue proteins, especially tubulin [59]. It is widely distributed throughout the body except for the brain and testes. Following a 3-hour infusion the alpha-half-life is 16 minutes, beta-half life is 140 minutes, and final elimination half-life is about 19 hours [60]. Paclitaxel is metabolized via cytochrome P-450 (CYP) isoenzymes 2C8 to 6-alpha-hydroxypaclitaxel and 3A4 to 3'-p-hydroxypaclitaxel and 6-alpha,3'-para-dihydroxypaclitaxel. Alterations of metabolism may occur when drugs affecting the CYP system are given concurrently. Elimination is due to hepatic metabolism, biliary and fecal excretion, and tissue binding. Approximately 70—80% of the dose is eliminated in the feces within 1 week. Only 1—8% of paclitaxel is eliminated unchanged in the urine.

Pharmacodynamics

Paclitaxel promotes accelerated assembly of excessively stable microtubules [59]. As a result, paclitaxel induces mitotic arrest of treated cells [59, 61] and subsequent apoptosis in vitro [62, 63] and in vivo [64, 65]. The kinetics of taxol-induced mitotic arrest and apoptosis in murine mammary carcinoma MCA-4 and ovarian carcinoma OCA-I tumors were determined [64]. Mice were treated with 60 mg/kg taxol given once when tumors reached 8 mm or twice, with the second dose being administered 3 days later. Tumors were histologically analyzed at various time points ranging from 1 to 96 h after treatment to quantify mitotic and apoptotic activity. Mitotic arrest was visible at 1 h, and increased with time to reach peak values of 36% in MCA-4 tumors and 22% in OCA-I tumors at 9 h, followed by a baseline of 1%-3% at 3 days for MCA-4 tumors and 1 day for OCA-I tumors. Apoptosis occurred at later time points following mitotic arrest, beginning at the time of peak mitotic arrest (9 h), increasing to the highest level of about 20% at 18-24 h after treatment and gradually declining to the normal level of 3%-6% after 3-4 days. Kinetic analysis performed after the second dose of taxol showed a considerably lower percentage of cells arrested in mitosis with minimum apoptosis compared to that from the 1st dose.

Paclitaxel in the treatment of early stage breast cancer

Paclitaxel is a standard adjuvant chemotherapy agent for the treatment of early stage breast cancer. The addition of every 3-week paclitaxel for 4 cycles following doxorubicin and cyclophosphamide has been shown to improve the DFS in patients with node-positive early stage breast cancer [66, 67]. A subsequent study by Sparano et al demonstrated that weekly paclitaxel is more effective than every 3-week paclitaxel in improving DFS and OS in the adjuvant setting, establishing the superiority of the weekly regimen for early stage breast cancer.

How Supplied:

Paclitaxel is commercially available in 5 ml (30 mg), 16.7 ml (100 mg) and 50 ml (300mg) multidose vials. Each ml contains 6 mg paclitaxel, 527 mg of purified Cremaphor EL®> and 49.7% dehydrated alcohol, USP. Refer to the package insert for further information.

Storage: Intact vials should be stored at room temperature, between 20-25°C (68-77°F), and protected from light. Further diluted solutions prepared in non-PVC containers with non-PVC tubing are stable for up to 27 hours at room temperature.

Route(s) of Administration: Paclitaxel will be administered as an IV infusion.

Method of Administration: All patients should be premedicated prior to administration in order to prevent severe hypersensitivity reactions. Such premedication may consist of dexamethasone 20 mg PO administered approximately 12 and 6 hours before paclitaxel, diphenhydramine (or its equivalent) 50 mg IV 30 to 60 minutes prior to paclitaxel, and cimetidine (300 mg) or ranitidine (50 mg) IV 30 to 60 minutes before paclitaxel. Administer through an in-line filter, not greater than 0.22 microns.

Availability: Paclitaxel is commercially available.

Preparation

Paclitaxel must be diluted to a final concentration of 0.3 to 1.2 mg/ml in either 0.9% sodium chloride or D5W. It is preferred that solutions be prepared in glass, polypropylene or polyolefin to avoid leaching of the diethylhexylphthalate (DEHP) plasticizer when polyvinyl chloride (PVC) bags are used. Non-PVC tubing and connectors are also preferred.

Toxicities

Hematologic: The most common dose limiting toxicity is myelosuppression, primarily leukopenia.

Allergy: Hypersensitivity reactions are common. They are thought to be due, at least in part, to the Cremaphor EL vehicle. Reactions generally occur early during administration. The most common symptoms observed in severe reactions include dyspnea, flushing, chest pain, and tachycardia. Patients should be pretreated to prevent hypersensitivity reactions.

Cardiac: Cardiovascular events observed with paclitaxel include hypotension and bradycardia; typically, neither discontinuation of paclitaxel nor specific therapy for the event is required.

Neurologic: The frequency and severity of neurologic events are dose-dependent. Peripheral neuropathy is rarely severe, but often interferes with function. Sensory symptoms may improve or resolve within several months of completion of treatment. Serious neurologic events such as grand mal seizures, syncope, ataxia and neuroencephalopathy are rare. Pre-existing neuropathies are not a contraindication to treatment with paclitaxel.

Gastrointestinal: The most common GI toxicities, which include nausea, vomiting, diarrhea and mucositis, are typically mild or moderate in severity. Mucositis occurs more frequently with a 24 hour infusion than with shorter infusion schedules.

Other: Although 60% of all patients experience arthralgia and myalgia, there is no consistent relationship between the dose or schedule of paclitaxel and the frequency of these events. The symptoms, which usually begin 2 or 3 days after paclitaxel treatment, are generally transient. Infusion site reactions are more common with 24 hour infusions. They are typically mild, consisting of erythema, tenderness, skin discoloration or swelling at the infusion site. Paclitaxel is generally considered to be an irritant, but isolated cases of more severe tissue damage following extravasation have been reported. Almost all patients receiving paclitaxel experience alopecia. Nail changes are uncommon. Occasionally, edema is seen, usually of mild severity.

8.9 Prohibited medications

The following agents must be stopped at least one week prior to registration and the harvest of tumor, serum and plasma for correlative science and must not be administered during the study intervention.

- Any agent with estrogenic or putatively estrogenic properties, including herbal preparations.

  NOTE: This includes hormone replacement therapy of any type, megestrol acetate, or raloxifene.
- Over-the-counter products and supplements considered to have an estrogenic effect such as: Ginseng, Gingko Biloboa, Black Cohosh, Dong Quoi and fortified soy supplements/phytoestrogen preparations.
- Any other anti-neoplastic approach such as chemotherapy or radiation must not be administered while the patient is taking study drug.

8.10 Concomitant medications

- Patients should receive full supportive care, including transfusions of blood and blood products, antibiotics, antiemetics, etc., when appropriate. The reason(s) for treatment, dosage, and the dates of treatment should be recorded.
- During weekly paclitaxel, filgrastim or sargramostim may be used on days 2 through 6 at the discretion of the treating physician if neutropenia becomes a problem. Pegfilgrastim may not be used during weekly paclitaxel.
- Anastrozole and fulvestrant are generally safe to administer with other medicines. Concomitant use of agents and herbal products that alter ER function are specifically not allowed.
- Vaginal atrophy refractory to local measures (example Replens, astroglide, etc) may be treated, at the discretion of the investigator, with intermittent vaginal estrogens (example vagifem, estrogen vaginal cream, testosterone and estradiol vaginal gel, Estring). Vaginal creams should be used sparingly and at the lowest dose necessary to control symptoms and still maintain an estrogen stimulated mucosa. Once a patient has had a week of daily dose estrogen, the cream may be used one to two times per week up to a maximum of three times per week. NOTE: Estring emits a constant daily dose of estrogen and its use should be limited to those women who continue to have symptoms despite three times a week vaginal cream use.

8.11 Criteria for early discontinuation of study drug

Study drugs must be discontinued if:

- Patient withdraws consent for the study
- Disease progression is confirmed by ultrasound or mammography studies after clinical assessment
- Second primary cancer (except basal cell and squamous cell skin cancer) is confirmed
- Intolerable toxicity (see Sections 8.3.1, 8.3.2, 8.3.3)

NOTE: If study drugs are discontinued early, refer to the Follow-up section (9.0) for guidelines.

9.0 FOLLOW-UP

The ***Clinical monitoring period*** is the phase of the trial where the patient has completed adjuvant therapy and is still being followed under the test schedule set out in Section 5.3. CRFs are to be completed after each of these protocol specified evaluations indicating all treatments received or a disease event since the last protocol specified evaluation. Patients will undergo research blood collection at recurrence if consented.

The ***Survival and disease status follow-up period*** is the phase of the trial where the patient's follow-up evaluations/treatment is at the discretion of their physician. The site that registered the patient is to submit CRFs yearly indicating whether the patient has had a disease event or has died.

9.1 Patients who have been registered to the study and signed a consent form, but have *withdrawn consent prior to receiving any study therapy* will be considered a cancellation. On-study data, but no biospecimens or images are to be submitted. No further follow-up is required.

9.2 Patients who have been *found not to have fulfilled all of the eligibility requirements* after signing a consent form and starting study therapy may continue on study following pre-protocol (that is, follow the treatment and evaluation schedules laid out in the protocol), if they are deriving benefit.

Otherwise, all online data collection up to the point of study therapy discontinuation are to be submitted and the patient is to go to the survival and disease status follow-up period where information regarding disease and survival status will be collected and reported by on-line data submission yearly from the time of registration until death or a maximum of 10 years post registration.

9.3 If any one of the following scenarios is true, then the patient will enter the survival and disease status follow-up phase of the study where information regarding disease and survival status will be collected and reported by on-line data submission yearly from the time of registration until death or a maximum of 10 years post registration.

Week 12 sample collection Ki67→ discontinued in Update #07

1. Patient discontinues their assigned neo-adjuvant endocrine therapy due to a 4 or 12 week Ki67 > 10% and chooses to go immediately to surgery.
2. Patient ***discontinues their assigned neoadjuvant endocrine therapy*** due to progression, refusal, intolerable toxicity, desire for alternative non-protocol therapy or other trials, or unable or unwilling to undergo surgery.
3. Patients who ***discontinued their neoadjuvant chemotherapy*** due to progression, refusal, intolerable toxicity, desire for alternative non-protocol therapy or other trials, or unable or unwilling to undergo surgery.
4. Patient ***with a modified PEPI 0 score*** discontinues assigned adjuvant treatment or clinical monitoring phase due to disease progression, refusal, intolerable toxicity, desire for alternative non-protocol therapy or other trial participation.

Week 12 sample collection Ki67→ discontinued in Update #07

5. Patient ***with a modified PEPI NON-0 score*** discontinues adjuvant treatment or clinical monitoring phase due to disease progression, refusal, or intolerable toxicity.
6. Patient ***who switched to neo-adjuvant chemotherapy due to*** 4 or 12 week Ki67 > 10% that discontinues adjuvant treatment or clinical monitoring phase due to disease progression, refusal, or intolerable toxicity.
7. A patient who develops a second primary cancer (other than basal cell or squamous cell skin cancer) is to discontinue protocol treatment and enter the survival and disease status follow-up phase of the study where treatment is at the discretion of the patient's medical team.

9.4 Research blood collection during the follow up period

Optional research blood (plasma in 1 EDTA tube and whole blood in 3 Streck tubes at each time point; Kits are available) is collected at the following time points (+/-6 months acceptable for yrs 1-10 collection):

- All patients: at 5 yrs after surgery and at recurrence (any time prior to the start of new treatment)
- mPEPI ≥ 4 or if switched to neoadjuvant chemotherapy due to Ki67 > 10% at wk 4 or 12: at postop visit (2-8 wks post surgery), yearly for years 1-10 (+/- 6 months) post surgery until recurrence and at recurrence (any time prior to the start of new treatment)

Please refer to Section 7.8.1 for sample collection details.

10.0 Evaluation of Outcomes

10.1 Neoadjuvant treatment phase

10.1.1 Clinical responses will be based on the WHO criteria

Prior to each cycle of neoadjuvant treatment and at the completion of neoadjuvant treatment, the longest axis and the perpendicular axis of the measurable lesion should be measured and recorded in metric notation by bi-dimensional tape, ruler or caliper technique.

Complete Response (CR) is defined as the disappearance of all known disease based on a comparison between the pre-treatment measurements and the measurements taken at the completion of neoadjuvant therapy (that is, at the end of cycle 6 neoadjuvant endocrine therapy for Arms I, II and III and the completion of neoadjuvant chemotherapy for the Neoadjuvant Chemotherapy Group. In addition there is no appearance of new lesions.

Partial Response (PR) is defined as a 50% or greater decrease in the product of the bi-dimensional measurements of the lesion (total tumor size) between the pre-treatment measurements and the measurements taken at the completion of neoadjuvant therapy (that is, at the end of cycle 6 neo-adjuvant endocrine therapy for Arms I, II and III and the completion of neoadjuvant chemotherapy for the Neoadjuvant Chemotherapy Group). In addition there can be no appearance of new lesions or progression of any lesion.

No Change (NC) is defined as a 50% decrease in total tumor size cannot be established nor has a 25% increase in the size of the lesion been demonstrated.

Progressive Disease (PD) is defined as a 25% or greater increase in the total tumor size of the lesion from its pretreatment measurements or the appearance of new lesions.

10.1.2 Endocrine resistance: a patient is said to have endocrine resistant disease if any of the following occurs:

Week 12 sample collection → discontinued in Update #07

- Ki67 > 10% after 4 weeks on neoadjuvant endocrine therapy;

- Ki67 > 10% after 12 weeks on neoadjuvant endocrine therapy;

- progressive disease is documented anytime during neoadjuvant endocrine therapy;

- surgical findings at 21-24 weeks post neoadjuvant endocrine therapy are such that: pT stage is 3/4, positive lymph nodes (except N1mi) are present or Ki67 > 2.7% (i.e. modified PEPI score of not being 0); or

- discontinued neoadjuvant endocrine treatment for any reason

10.2 Surgery

10.2.1 A pathologic complete response is defined as no histology evidence of invasive tumor cells in the surgical breast specimen and sentinel or axillary lymph nodes.

10.3 Following surgery, diagnosis of breast cancer recurrence and other cancer events

10.3.1 Local recurrence

Local recurrence is defined as histologic evidence of ductal carcinoma in situ or invasive breast cancer in the ipsilateral breast or chest wall.

10.3.2 Regional recurrence

Regional recurrence is defined as the cytologic or histologic evidence of disease in the ipsilateral internal mammary, ipsilateral supraclavicular, ipsilateral infraclavicular and/or ipsilateral axillary nodes or soft tissue of the ipsilateral axilla.

10.3.3 Distant recurrence

Distant recurrence is defined as the cytologic, histologic, and/or radiographic evidence of disease in the skin, subcutaneous tissue, lymph nodes (other than local or regional metastasis), lung, bone narrow, central nervous system or histologic and/or radiographic evidence of skeletal or liver metastasis.

10.3.4 Second primary breast cancer

Second primary breast cancer is defined histologic evidence of ductal carcinoma in situ or invasive breast cancer in the contralateral breast or chest wall.

10.3.5 Second primary cancer (non-breast)

Any non-breast second primary cancer other than squamous or basal cell carcinoma of the skin, melanoma in situ, or carcinoma in situ of the cervix is to be reported and should be confirmed histologically whenever possible.

10.3.6 Death

Underlying cause of death is to be reported.

11.0 ADVERSE EVENT REPORTING

The prompt reporting of adverse events (AEs) is the responsibility of each investigator engaged in clinical research, as required by Federal Regulations. Toxicities/adverse events must be described and graded using the terminology and grading categories defined in the most current version of the NCI's Common Toxicity Criteria (CTCAE) version 4.0. However, CTCAE v5.0 must be used for serious AE reporting through CTEP-AERS as of April 1, 2018. The CTCAE is available at ctep.cancer.gov/protocolDevelopment/electronic_applications/ctc.htm. Attribution to protocol treatment for each adverse event must be determined by the investigator and reported on the required forms. Please refer to the section on AE reporting procedures. NCI Guidelines: Adverse Event Reporting Requirements for further details.

Attribution of the AE:

- Definite - The AE *is clearly related* to the study treatment.

- Probable - The AE *is likely related* to the study treatment.

- Possible -The AE *may be related* to the study treatment.

- Unlikely – The AE *is doubtfully related* to the study treatment.

- Unrelated – The AE *is clearly NOT* related to the study treatment.

Arms I, II and III

Adverse events are to be graded at each evaluation and pretreatment symptoms/conditions are to be evaluated at baseline per the Common Terminology Criteria for Adverse Events (CTCAE) CTEP Version 4.0 grading unless otherwise stated:

System Organ Class	Adverse Event Symptoms	Baseline	Each Evaluation
Gastrointestinal disorders	Nausea	X	X
General disorders and administration site conditions	Fatigue	X	X

Vascular disorders	Hot flashes	X	X
Psychiatric disorders	Agitation<br>Anxiety<br>Depression<br>Euphoria	X<br>X<br>X<br>X	X<br>X<br>X<br>X
Musculoskeletal and connective tissue disorders	Arthralgia<br>Myalgia	X<br>X	X<br>X

Neoadjuvant Chemotherapy Group

Adverse events are to be reported at the end of neoadjuvant chemotherapy and pretreatment symptoms/conditions to be evaluates at baseline per the Common Terminology Criteria for Adverse Events (CTCAE) CTEP Version 4.0 grading unless otherwise stated (or unless submitting an expedited report):

System Organ Class	Adverse Event Symptoms	Baseline	Each Evaluation
General disorders and administration site conditions	Infusion related reaction		X
Immune system disorders	Allergic reaction/anaphylaxis		X
Musculoskeletal and connective tissue disorders	Arthralgia<br>Myalgia	X<br>X	X<br>X
Nervous system disorders	Peripheral sensory neuropathy	X	X

11.1 Routine adverse event reporting

The reporting of adverse events (AEs) described in the table above is in addition to and does not supplant the reporting of adverse events as part of the report of the results of the clinical trial, e.g., cooperative group data reporting (see Section 13.0).

The FDA has determined that fulvestrant is IND exempt for this study.

Adverse event data collection and reporting, which are required as part of every clinical trial, are done to ensure the safety of patients enrolled in the studies as well as those who will enroll in future studies using similar agents. Adverse events are reported in a routine manner at scheduled times during the trial using Medidata Rave. Additionally, certain adverse events must be reported in an expedited manner for more timely monitoring of patient safety and care. Please see Section 11.2 for instructions related to expedited AE reporting.

11.2 Expedited adverse event reporting

Investigators are required by federal regulations to report serious adverse events as defined below. Alliance investigators are required to notify the Alliance Central Protocol Operations Program Office, the Study Chair, and their Institutional Review Board if a patient has an adverse event requiring expedited reporting. All such events must be reported in an expedited manner using the NCI CTEP Adverse Event Reporting System (CTEP-AERS). The descriptions and grading scales found in the NCI Common Terminology Criteria for Adverse Events (CTCAE) version 5.0 will be utilized for AE reporting beginning April 1, 2018. All appropriate treatment areas should have access to a copy of the CTCAE. A copy of the CTCAE version 5.0 can be downloaded from the CTEP web site https://ctep.cancer.gov/protocolDevelopment/electronic_applications/ctc.htm.

The Alliance requires investigators to route all expedited adverse event reports through the Alliance Central Protocol Operations Program Office for Alliance coordinated studies.

Be sure to read this entire protocol section, as requirements are described in both the table and the bullet points following the table. Note that the additional instructions or exclusions are protocol specific, and in the case of a conflict, the additional instructions or exclusions supersede the table.

A011106: Expedited Reporting Requirements for Adverse Events that Occur within 30 Days of the Last Administration of Treatment[1]

FDA REPORTING REQUIREMENTS FOR SERIOUS ADVERSE EVENTS (21 CFR Part 312)

NOTE: Investigators **MUST** immediately report to the sponsor (NCI) **ANY** Serious Adverse Events, whether or not they are considered related to the investigational agent(s)/intervention (21 CFR 312.64)

An adverse event is considered serious if it results in **ANY** of the following outcomes:

1) Death
2) A life-threatening adverse event
3) An adverse event that results in inpatient hospitalization or prolongation of existing hospitalization for ≥ 24 hours
4) A persistent or significant incapacity or substantial disruption of the ability to conduct normal life functions
5) A congenital anomaly/birth defect.
6) Important Medical Events (IME) that may not result in death, be life threatening, or require hospitalization may be considered serious when, based upon medical judgment, they may jeopardize the patient or subject and may require medical or surgical intervention to prevent one of the outcomes listed in this definition. (FDA, 21 CFR 312.32; ICH E2A and ICH E6).

ALL SERIOUS adverse events that meet the above criteria **MUST** be immediately reported via CTEP-AERS within the timeframes detailed in the table below.

Hospitalization	Grade 1 Timeframes	Grade 2 Timeframes	Grade 3 timeframes	Grade 4 & 5 Timeframes
Resulting in Hospitalization ≥ 24 hrs	10 Calendar Days			24-Hour 5 Calendar Days
Not resulting in Hospitalization ≥ 24 hrs	Not required		10 Calendar Days	

Expedited AE reporting timelines are defined as:

- “24-Hour; 5 Calendar Days” - The AE must initially be reported via CTEP-AERS within 24 hours of learning of the AE, followed by a complete expedited report within 5 calendar days of the initial 24-hour report.
- “10 Calendar Days” - A complete expedited report on the AE must be submitted within 10 calendar days of learning of the AE.

[1]Serious adverse events that occur more than 30 days after the last administration of treatment require reporting as follows:

Expedited 24-hour notification followed by complete report within 5 calendar days for:

- All Grade 4, and Grade 5 AEs that are at least possibly related to treatment

Expedited 10 calendar day reports for:

Grade 2 adverse events resulting in hospitalization or prolongation of hospitalization, and that are at least possibly related to treatment.

- Grade 3 adverse events that are at least possibly related to treatment.

Additional Instructions or Exclusions to CTEP-AERS Reporting Requirements

- Adverse events occurring in patients receiving neoadjuvant chemotherapy (e.g., paclitaxel) without endocrine therapy do not require CTEP-AERS.
- Adverse events occurring in patients receiving adjuvant chemotherapy (e.g., paclitaxel) with endocrine therapy require CTEP-AERS according to the instructions and table above.
- Death due to progressive disease should be reported as Grade 5 "Disease progression" in the system organ class (SOC) "General disorders and administration site conditions." Evidence that the death was a manifestation of underlying disease (e.g., radiological changes suggesting tumor growth or progression: clinical deterioration associated with a disease process) should be submitted.
- For purposes of expedited reporting, expected events are those listed in the product information for anastrozole, fulvestrant or chemotherapy or in Section 11.3. Expected events do not require CTEP-AERS, but should be reported via routine data submission.

- Secondary malignancy: A secondary malignancy is a cancer caused by treatment for a previous malignancy (e.g., treatment with investigational agent/intervention, radiation or chemotherapy). A secondary malignancy is not considered a metastasis of the initial neoplasm.
  - CTEP requires all secondary malignancies that occur following treatment with an agent under an NCI IND/IDE be reported via CTEP-AERS. In CTCAE version 5.0, three options are available to describe the event:
  - -Leukemia secondary to oncology chemotherapy (e.g., acute myelocytic leukemia [AML])
  - -Myelodysplastic syndrome (MDS)
  - -Treatment-related secondary malignancy
  - Any malignancy possibly related to cancer treatment (including AML/MDS) should also be reported via the routine reporting mechanisms outlined in each protocol.

- Second malignancy: A second malignancy is one unrelated to the treatment of a prior malignancy (and is NOT a metastasis from the initial malignancy). Second malignancies require ONLY routine reporting unless otherwise specified.
- All pregnancies and suspected pregnancies occurring in female patients or in the partner of a male patient during or within 30 days after the end of treatment on A011106 must be reported via CTEP-AERS. Using CTCAE version 4.0, pregnancies should be reported as Grade 4 "Pregnancy, puerperium and perinatal conditions-other (fetal exposure)" (see CTCAE Pregnancy, puerperium and perinatal conditions).
- Pregnancy loss is defined in CTCAE as "Death in utero." Any pregnancy loss should be reported expeditiously as Grade 4 "Pregnancy loss" under the Pregnancy, puerperium and perinatal conditions SOC. A pregnancy loss should NOT be reported as a Grade 5 event under the Pregnancy, puerperium and perinatal conditions SOC, as currently CTEP-AERS recognizes this event as a patient death.
- A neonatal death should be reported expeditiously as Grade 4, "Death neonatal" under the General disorders and administration SOC.
- All adverse events reported via CTEP-AERS (i.e., serious adverse events) should also be forwarded to your local IRB, according to local IRB policies.
- The reporting of adverse events described above is in addition to routine data submission, for those events requiring expedited reporting.

11.3 Expected adverse events

11.3.1 Adverse event list(s) for anastrozole

Please also refer to the package insert(s) for the comprehensive list of adverse events.

- Likely **(>15%)**

  Weakness, joint or musculoskeletal pain, back pain, nausea, vomiting, mood disturbance, and Hoh flashes

- Less Likely **(1-15%)**

  Vaginal discharge, vaginal bleeding, cataracts

- Rare **(<1%)**

  Bone fracture, deep venous thrombosis, thomboembolic events, ischemic cerebrovascular event, endometrial cancer, angina, and heart attack/MI

11.3.2 Adverse event list(s) for fulvestrant[68]

Please also refer to the package insert(s) for the comprehensive list of adverse events.

- Likely **(> 15%)**

  Nausea, muscle/joint/bone pain, headache, tiredness, hot flashes, weakness, increased liver enzymes

- Less likely **(1-15%)**

  Injection site pain, vomiting, constipation, shortness of breath, cough, loss of appetite

- Rare (< 1%)
  Vaginal bleeding

11.3.3 Adverse event lists for paclitaxel is listed in Section 8.8.3 and below.

Please also refer to paclitaxel package insert.

Hematologic: The most common dose limiting toxicity is myelosuppression, primarily leukopenia.

Allergy: Hypersensitivity reactions are common. They are thought to be due, at least in part, to the Cremaphor EL vehicle. Reactions generally occur early during administration. The most common symptoms observed in severe reactions include dyspnea, flushing, chest pain, and tachycardia. Patients should be pretreated to prevent hypersensitivity reactions.

Cardiac: Cardiovascular events observed with paclitaxel include hypotension and bradycardia; typically, neither discontinuation of paclitaxel nor specific therapy for the event is required.

Neurologic: The frequency and severity of neurologic events are dose-dependent. Peripheral neuropathy is rarely severe, but often interferes with function. Sensory symptoms may improve or resolve within several months of completion of treatment. Serious neurologic events such as grand mal seizures, syncope, ataxia and neuroencephalopathy are rare. Pre-existing neuropathies are not a contraindication to treatment with paclitaxel.

Gastrointestinal: The most common GI toxicities, which include nausea, vomiting, diarrhea and mucositis, are typically mild or moderate in severity. Mucositis occurs more frequently with a 24 hour infusion than with shorter infusion schedules.

Other: Although 60% of all patients experience arthralgia and myalgia, there is no consistent relationship between the dose or schedule of paclitaxel and the frequency of these events. The symptoms, which usually begin 2 or 3 days after paclitaxel treatment, are generally transient. Infusion site reactions are more common with 24 hour infusions. They are typically mild, consisting of erythema, tenderness, skin discoloration or swelling at the infusion site. Paclitaxel is generally considered to be an irritant, but isolated cases of more severe tissue damage

following extravasation have been reported. Almost all patients receiving paclitaxel experience alopecia. Nail changes are uncommon. Occasionally, edema is seen, usually of mild severity.

11.3.4 Surgery

Pain, edema in breast or arm, numbness at incision site and in arm, scarring and/or indentation in the area of the incision, bleeding/hematoma/seroma, wound infection, symptoms from injury to the brachial plexus, increased susceptibility to infection, decreased range of motion.

12.0 DATA CONSIDERATIONS

Medidata Rave is a clinical data management system being used for data collection for this trial/study. Access to the trial in Rave is controlled through the CTEP-IAM system and role assignments.

Requirements to access Rave via iMedidata:

- A valid CTEP-IAM account; and
- Assigned a Rave role on the LPO or PO roster at the enrolling site of: Rave CRA, Rave Read Only, Rave CRA (LabAdmin), Rave SLA, or Rave Investigator.

Rave role requirements:

- Rave CRA or Rave CRA (Lab Admin) role must have a minimum of an Associate Plus (AP) registration type;
- Rave Investigator role must be registered as an Non-Physician Investigator (NPIVR) or Investigator (IVR); and
- Rave Read Only role must have at a minimum an Associates (A) registration type

Refer to https://ctep.cancer.gov/investigatorResources/default.htm for registration types and documentation required.

Upon initial site registration approval for the study in Regulatory Support System (RSS), all persons with Rave roles assigned on the appropriate roster will be sent a study invitation e-mail from iMedidata. To accept the invitation, site staff must log in to the Select Login (https://login.imedidata.com/selectlogin) using their CTEP-IAM username and password and click on the accept link in the upper right-corner of the iMedidata page. Site staff will not be able to access the study in Rave until all required Medidata and study specific trainings are completed. Trainings will be in the form of electronic learnings (eLearnings) and can be accessed by clicking on the link in the upper right pane of the iMedidata screen. If an eLearning is required and has not yet been taken, the link to the eLearning will appear under the study name in iMedidata instead of the Rave EDC link; once the successful completion of the eLearning has been recorded, access to the study in Rave will be granted, and a Rave EDC link will display under the study name.

Site staff that have not previously activated their iMedidata/Rave account at the time of initial site registration approval for the study in RSS will receive a separate invitation from iMedidata to activate their account. Account activation instructions are located on the CTSU website in the Data Management section under the Rave resource materials (Medidata Account Activation and Study Invitation Acceptance). Additional information on iMedidata/Rave is available on the CTSU members' website in the Data Management > Rave section at www.ctsu.org/RAVE/ or by contacting the CTSU Help Desk at 1-888-823-5923 or by e-mail at ctsucontact@westat.com.

Common Terminology Criteria for Adverse Events: This study will utilize the Common Terminology Criteria for Adverse Events (CTCAE) version 4.0 for routine toxicity and adverse event (AE) reporting. For expedited AE reporting, CTCAE version 5.0 will be used.

12.1 Patient data quality control

All data received will be subjected to Alliance validation and quality-control measures. Issues arising from inaccurate, discrepant or incomplete data will be communicated to participating sites

on a regular basis. Any data submitted via Medidata Rave® is subject to audit against the patient's source documents. Consistent failure to complete and submit data in a timely fashion may subject a participating site to sanction up to and including the suspension of participation in the study.

12.2 Data Quality Portal

The Data Quality Portal (DQP) provides a central location for site staff to manage unanswered queries and form delinquencies, monitor data quality and timeliness, generate reports, and review metrics.

The DQP is located on the CTSU members' website under Data Management. The Rave Home section displays a table providing summary counts of Total Delinquencies and Total Queries. DQP Queries, DQP Delinquent Forms and the DQP Reports modules are available to access details and reports of unanswered queries, delinquent forms, and timeliness reports. Review the DQP modules on a regular basis to manage specified queries and delinquent forms.

The DQP is accessible by site staff that are rostered to a site and have access to the CTSU website. Staff that have Rave study access can access the Rave study data using a direct link on the DQP.

To learn more about DQP use and access, click on the Help icon displayed on the Rave Home, DQP Queries, and DQP Delinquent Forms modules.

Note: Some Rave protocols may not have delinquent form details or reports specified on the DQP. A protocol must have the Calendar functionality implemented in Rave by the Lead Protocol Organization for delinquent form details and reports to be available on the DQP. Site staff should contact the LPO Data Manager for their protocol regarding questions about Rave Calendaring functionality.

13.0 STATISTICAL CONSIDERATIONS

13.1 Study design:

This clinical trial was designed to address two issues: (1) whether the endocrine resistance rate with either of the fulvestrant containing regimens is significantly less than the endocrine resistance rate with anastrozole, and (2) whether the 5 year recurrence-free survival rate (RFS) among women with a modified PEPI score of 0 following treatment with the neoadjuvant anastrozole is at most 90%.

13.2 Endpoints

13.2.1 Primary endpoints

Neoadjuvant Comparison Phase (First Phase):

The primary endpoint of the neoadjuvant portion of this trial is the rate of endocrine resistant disease. A patient is considered to have endocrine resistant disease if any of the following holds true:

Week 12 sample collection → discontinued in Update #07

- Ki67 > 10% after 4 weeks on neoadjuvant endocrine therapy;
- Ki67 > 10% after 12 weeks on neoadjuvant endocrine therapy;
- Progressive disease is documented anytime during neoadjuvant endocrine therapy;
- Surgical findings at 24-26 weeks post neoadjuvant endocrine therapy are such that: pT stage is 3/4, positive lymph nodes (except N1mi) are present or Ki67 > 2.7% (i.e. non-zero modified PEPI score of not being 0); or
- Discontinued study treatment for any reason without completing surgery (to preserve the intent to treat principle).

Note: A patient who does not meet any of the criteria for endocrine resistant disease will be referred to as having endocrine sensitive disease.

Thus, the endocrine resistant disease rate (ERR) for a given treatment arm is the percentage of patients randomized to that treatment arm who fulfill the criteria for endocrine resistant disease (and 100% - ERR is the endocrine sensitive disease rate).

A secondary endpoint is the pathologic complete response rate (pCR rate): The pathologic complete response rate for a given treatment arm is defined as 100% times the proportion of patients with no histologic evidence of invasive tumor cells in the surgical breast specimen and the axillary lymph nodes among all the patients randomized to that treatment arm.

Modified PEPI 0 Validation Phase (Second Phase): Primary endpoint is the 5 year RFS rate among the women whose modified PEPI score was 0.

The primary endpoint is recurrence-free survival (RFS) where recurrence-free survival is defined as the time of surgery to the first of the following events: Invasive ipsilateral breast tumor recurrence, local/regional invasive breast cancer recurrence, distant recurrence or death from breast cancer (STEEP criteria).

13.3 Stratification

During the neoadjuvant comparison phase of the trial, the Pocock - Simon dynamic allocation procedure will be used to allocate an equal number of patients to each of the 3 treatment strategies. This procedure will balance the marginal distributions of the stratification factors between these treatment strategies. The stratification factors are clinical tumor stage, clinical lymph node status, and performance status (Section 6.2).

Effective November 1st, 2018, after the enrollment goal of 1275 patients (425 patients per arm) for the neoadjuvant phase of the trial is met and the analysis of their endocrine resistant data is underway, all newly enrolled patients will be assigned to the anastrozole arm.

13.4 Accrual rate

It is anticipated that 20 patients per month will be enrolled.

13.5 Sample size

The sample size required to complete the neoadjuvant comparison is 1275 patients.

We anticipate that enrollment will be open to the anastrozole arm for an additional 9 months [this is to allow the last of the 1275 patients to complete neoadjuvant treatment (24 weeks), undergo surgery (2-4 weeks), and submit their data forms (4 weeks) and then to complete data analysis (4 weeks)]. With an accrual rate of 20 patients per month, we anticipate enrolling an additional 180 patients after the 1275 patient goal is met, and prior to the release of the neoadjuvant phase results. That is, we anticipate a total of 605 patients enrolled onto the anastrozole arm before the close of study enrollment.

13.5.1 Study design and sample size justification for neoadjuvant comparison (First Phase)

The primary aims of the first phase of this clinical trial are:

1. To determine whether the endocrine sensitive disease rate among those randomized to the fulvestrant alone (Arm 2) is at least 10% higher than the endocrine sensitive disease rate among those randomized to anastrozole alone (Arm 1).
2. To determine whether the endocrine sensitive disease rate among those randomized to the combination of fulvestrant and anastrozole (Arm 3) is at least 10% higher than the endocrine sensitive disease rate among those randomized to anastrozole alone (Arm 1).
3. If both of the fulvestrant containing arms are found to have an endocrine sensitive disease rate at least 10% higher than that of the anastrozole arm, we will assess whether the endocrine sensitive disease rate is greater with the combination of anastrozole and fulvestrant than with fulvestrant alone.

An estimate for the proportion of patients who will have endocrine sensitive disease in the anastrozole alone arm is taken from the ACOSOG Z1031 Cohort B study.

Of the 236 eligible patients, 49 (21%) patients had a 2 week Ki67 > 10%. There were another 22 patients who lacked sufficient tumor tissue to ascertain a 2 week Ki67: Six of these patients choose to continue on AI and not be re-biopsied and the remaining 16 patients were re-biopsied after 4 weeks of treatment with a finding of Ki67 ≤ 10%. One patient who had a 2-4 week Ki67 ≤ 10% refused to continued AI treatment.

Of the 186 patients who continued on AI, 9 patients did not undergo surgery due to refusal, comorbid conditions, or disease progression; 4 patients who had surgery either did not have nodal surgery or did not have a Ki67 value determined from their surgical specimen; and 109 had a non-zero mPEPI score.

Thus, we would expect approximately 70% [1-(236-49-1-9-4-109)/236] of the patients enrolled onto this trial to meet one or more of the criteria for endocrine resistant disease.

Two pairwise comparisons are planned, namely, (1) the endocrine sensitive disease rate in the fulvestrant arm to that of the anastrozole arm, and (2) the endocrine sensitive disease rate in the anastrozole + fulvestrant arm, to that of the anastrozole arm. To maintain an overall alpha level of 0.05, these comparisons will be made with the probability of a Type I error set at 0.025.

With 425 patients per treatment arm, a one-tailed alpha=0.025 chi-square test of two independent proportions will have 84% power to detect an increase of 10% or more in the endocrine sensitive disease rate for a given fulvestrant containing arm relative to that in the anastrozole arm when the endocrine sensitive disease rate is at most 30% in the anastrozole arm.

If both of the fulvestrant containing arms are found to have an endocrine sensitive disease rate significantly greater than anastrozole, then we will test whether the endocrine sensitive disease rate is greater with the combination of anastrozole and fulvestrant, than with fulvestrant alone.

The following table provides the power for a one-tailed alpha=0.05 chi-square test of two independent proportions will have to detect that one of the fulvestrant containing regimens have a higher endocrine sensitive disease rate (ESDR) than the other fulvestrant containing regimen.

ESDR in the fulvestrant containing regimen with poor rater	ESDR in the fulvestrant containing regimen with better rate	Sample size per treatment arm	Power
0.40	0.50	425	88%
0.40	0.45	425	40%

13.5.2 Study design and sample size justification for Modified PEPI 0 validation (Second Phase)

Background: A number of clinical trials are assessing whether there is a subgroup of breast cancers which do not derive significant clinical benefit with adjuvant chemotherapy. Albain et al. reported that the 10 yr. DFS was 48% (5 yr DFS rate of 69%) in the 361 women with pathological stage T1–3, N1–2 ER positive breast cancer randomized to adjuvant tamoxifen alone arm of the Southwest Oncology Group (SWOG)-8814, INT-0100. However, when they examined the subgroup of women with 1-3 positive lymph nodes, ER-positive, HER2 negative breast cancer and an Oncotype DX recurrence score of ≤ 25 who were randomized to tamoxifen alone, the 5 year DFS was found to be 91%.

The second phase of this clinical trial is designed to examine whether 5 year RFS rate is at most 90% among the women randomized to anastrozole who had a modified PEPI score of 0 and continue to receive protocol treatment for an additional 4.5 years after surgery.

The total number of patients that will be available to accomplish the goals of the adjuvant phase of this trial will depend upon will depend ESDR. If the ESDR is between 25 to 40%, we would expect 150 of the 605 (425 + 180) patients enrolled on that arm to be eligible for the second phase of this trial.

The table below provides the power under a variety of scenarios that a one-sided alpha=0.05 nonparametric Brookmeyer-Crowley type one sample survival test [69] will have to reject that the 5 year RFS rate is at most 90% in patients treated with anastrozole who have endocrine sensitive disease when the true 5 year RFS rate in this patient population is at least 95%.

Enrollment period	Minimum follow-up period	ESDR	sample size	power
		0.25	150	80.0%
75 months	48 months	0.30	180	84.7%
		0.35	210	88.4%

13.5.3 Sample size for the neoadjuvant paclitaxel treatments

The number of patients who will switch to neoadjuvant paclitaxel depends upon the percentage of patients with a Ki67 value > 10% after 2-4 weeks of neoadjuvant endocrine therapy who agree to switch to neoadjuvant chemotherapy, as well as the percentage of these patients who will choose paclitaxel over other neoadjuvant chemotherapy regimens. As of August 24, 2011, 45 (21%) of the 211 patients for whom 2-4 week Ki67 testing was completed had a Ki67 value > 10% but 10 chose immediate surgery and 1 remained on AI. As such, we expect

approximately 16% of 1455 (1275 + 180) patients registered onto this study (n=232) to be switched to neoadjuvant chemotherapy.

13.6. Study duration

With an accrual rate of 20 patients per month, it is anticipated that the enrollment goal of 1275 patients for the neoadjuvant phase of this clinical trial will be met in 5.5 years.

An additional 9 months will be needed to gather data on the 24 week treatment course and surgical outcomes of these 1275 patients, and complete the analysis of the first phase of the trial.

The efficacy analysis for the adjuvant phase of this trial should begin approximately 4 to 5 years after the release of the neoadjuvant phase results.

13.7 Analyses plans

13.7.1 First phase

For each of the following objectives:

- To determine whether fulvestrant, administered for 24 weeks as neoadjuvant endocrine treatment, decreased the proportion of endocrine resistant tumors relative to patients treated with 1 mg of anastrozole.
- To determine whether fulvestrant in combination with anastrozole, administered for 24 weeks as neoadjuvant endocrine treatment, decreases the proportion of endocrine resistant tumors relative to patients treated with 1 mg of anastrozole.
- To assess whether the degree of tumor Ki67 suppression at week 4 differs between patients randomized to fulvestrant and those randomized to anastrozole.
- To assess whether the degree of tumor Ki67 suppression at week 4 differs between patients randomized to fulvestrant in combination with anastrozole and those randomized to anastrozole alone.

All women who meet all of the eligibility criteria and began neoadjuvant endocrine therapy will be included in the analyses of these objectives. A 95% binomial confidence interval for the true neoadjuvant endocrine resistance rate will be constructed for each treatment arm.

Logistic regression modeling with likelihood ratio tests will be used to examine the likelihood that endocrine resistance differs with respect to neoadjuvant treatment or pretreatment patient and disease characteristics [70].

Following the approach of Dowsett et al.[26], the percent change in the 4-week Ki67 value from pretreatment levels will be determined on a log scale. For each treatment arm, a 95 % t-confidence interval for the mean percent change in the 4-week Ki67 value from baseline will be constructed if appropriate. For each of the 3 possible pairwise comparisons of percent change in Ki67 value from baseline between neoadjuvant endocrine treatment groups, a two sample t test for means or a Wilcoxon rank sum test will be used to examine whether the percent change in 4-week Ki67 values from baseline differs with respect to treatment.

For each of the following objectives:

- To examine the differences in surgical outcome, clinical and radiological response rates, and the safety profile between the fulvestrant and the anastrozole arm.
- To examine the differences in surgical outcome, clinical and radiological response rates, and the safety profile between patients randomized to fulvestrant in combination with anastrozole and those randomized to anastrozole.

All women who meet all of the eligibility criteria, began neoadjuvant endocrine therapy and had a 4 week Ki67 value < 10% will be included in the analysis of these objectives. For the

outcomes of clinical response rate, radiographic response rate, pathologic complete response rate, and pCR/RCB-1 rate, a 95% binomial confidence interval for the true rate of the outcome will be constructed by treatment arm. Logistic regression modeling with likelihood ration tests will be used to examine whether the likelihood of a given response outcome differs with respect to neoadjuvant treatment; pretreatment patient and disease characteristics; or week 4 biopsy findings (detailed in Section 15.0).

The type, severity and attribution of each adverse event reported will be assessed using the NCI CTCAE definitions. The proportion of patients who develop severe (grade 3+) toxicity considered possibly, probably or definitively related to treatment will be determined for each treatment arm.

For the following objective:

- To examine the differences in surgical outcome, clinical and radiological response rates and safety profile and those who switched to neoadjuvant chemotherapy.

All women who meet all of the eligibility criteria, has a 4-week Ki67 value > 10% after 4 or 12 weeks of neoadjuvant endocrine therapy and switched to neoadjuvant chemotherapy will be included in the analysis of these objectives. For the outcomes of clinical response rate, radiographic response rate, pathologic complete response rate, and pCR/RCB-1 rate, a 95% binomial confidence interval for the true rate of the outcome will be constructed for those who chose neoadjuvant paclitaxel as well as those who choose another neoadjuvant chemotherapy regimen. For the cohort who chose neoadjuvant paclitaxel, the type, severity and attribution of each adverse event reported will be assessed using the NCI CTCAE definitions. The proportion of patients who develop sever (grade 3+) toxicity considered possibly, probably or definitively related to treatment will be determined.

13.7.2 Second phase

All women who had a week-4 Ki67 value $\leq$ 10% and a modified PEPI score of 0 will be included in the analysis of recurrence-free survival.

Recurrence-free survival (RFS) is defined as the time from surgery to the first of the following disease events: Invasive ipsilateral breast tumor recurrence, local/regional invasive recurrence, distant recurrence, or death. If a patient develops DCIS or invasive breast cancer in the contralateral breast, or a second non-breast cancer primary (except basal or squamous cell skin cancer or cervical carcinoma in situ), her recurrence-free survival time will be censored at the time of that diagnosis. Patients alive without any disease events, second non-breast cancer primary events, or contralateral DCIS or invasive breast events and patients who die of unknown or non-breast cancer causes will be censored at the time of their last breast evaluation.

Disease free survival (DFS) is defined as the time from surgery to the first of the following disease events: Invasive ipsilateral breast tumor recurrence, local/regional invasive recurrence, distant recurrence, second primary cancer (except basal or squamous cell skin cancer or cervical carcinoma in situ) or death due to any cause.

Overall survival time is the time from surgery to death due to any cause.

For each neoadjuvant treatment arm, the distribution of recurrence-free survival times, disease free survival , and overall survival will be estimated using the Kaplan-Meier method [71].

A 95% confidence interval for the 5 year RFS rate will be constructed using a point-wise confidence interval for the survival function based on a log-minus-log transformation. Cox modeling with partial likelihood score tests will be used to assess the strength of association between RFS and patient/disease/treatment characteristics [68, 72].

For the women who have a week-4 Ki-67 value > l0% and switched to paclitaxel, the distributions of recurrence-free survival times and overall survival times will be estimated using the Kaplan-Meier method with the start point of study entry.

For the women who have a week-4 Ki67 value ≤ 10%, and a non-zero modified PEPI score, the distributions of recurrence-free survival times and overall survival times will be estimated using the Kaplan-Meier method with the start point of surgery.

14.0 REGULATORY CONSIDERATIONS

14.1 Registering physician

All enrolling investigators must have an NCI investigator number and must maintain an "active" investigator registration status through the annual submission of a complete investigator registration packet to the Pharmaceutical Management Branch.

14.2 Registering institution

Patients must be enrolled at clinical sites that have a valid assurance number from the United States Office for Human Research Protections (OHRP). Most institutions have a Multiple Project Assurance (MPA), Cooperative Project Assurance (CPA) number or Federal wide Assurance (FWA). If the clinical site does not have such an assurance, the clinical site must apply and obtain an assurance before patients can be enrolled to this study.

Unaffiliated Investigator Agreements (UIAs) are needed from investigators who independently accrue patients on ambulatory protocols outside an institution (e.g., in private practice) but who rely on an institution's IRB for review of ACOSOG protocols.

14.3 Submission of IRB approval

Documentation of IRB approval must be submitted to CTSU for entry into the Regulatory Support System (RSS) before patient registration will be allowed. Submission instructions and coversheets are available at http://www.ctsu.org/rss/.

14.4 Inclusion of women and minorities

This study will be available to all eligible female patients, regardless of race or ethnic origin. There is no information currently available regarding differential effects of this protocol in subsets defined by race or ethnicity; and there is no reason to expect such differences to exist. Therefore, although the planned analysis will, as always, look for differences in accuracy based on racial groupings, the sample size is not increased in order to provide additional power for such subset analyses.

Men are excluded from this study because the number of men with breast cancer is insufficient to provide a statistical basis for assessment of effects in this subpopulation of people with breast cancer.

Race and Ethnicity Table

**Accrual Targets**					
**Ethnic Category**	**Sex/Gender**				
	**Females**		**Males**		**Total**
Hispanic or Latino	189	+	0	=	189
Not Hispanic or Latino	1286	+	0	=	1286
**Ethnic Category: Total of all subjects**	1475 (A1)	+	0 (B1)	=	1475 (C1)
**Racial Category**					
American Indian or Alaskan Native	4	+	0	=	4
Asian	48	+	0	=	48
Black or African American	220	+	0	=	220
Native Hawaiian or other Pacific Islander	1	+	0	=	1
White	1202	+	0	=	1202
**Racial Category: Total of all subjects**	1475(A2)	+	0 (B2)	=	1475 (C2)

14.5 Clinical site audits

All clinical sites at which patients are enrolled are subject to an audit in accordance with guidelines provided by and available from the Clinical Trials Monitoring Branch (CTMB) of the NCI. Information on these regulations may be obtained from the CTMB web site at http://ctep.cancer.gov/.

14.6 Clinical monitoring

This study will be monitored by the current version of the Clinical Data Update System (CDUS). Cumulative CDUS data will be submitted quarterly by Alliance to CTEP by electronic means.

14.7 Data safety and monitoring

The Alliance Data and Safety Monitoring Board will review the data available from the trial at each of its biannual meetings. This will include accrual data, adverse events, and results of interim analyses when available. The DSMB will also consider the evidence regarding safety, i.e. adverse events and the feasibility of completing the trial, i.e. the accrual rate.

15.0 CORRELATIVE RESEARCH STUDIES (A011106-ST)

15.1 Real time essential biomarker studies

15.1.1 Hypotheses

1. Patients who achieve a modified PEPI score of 0 following 24 weeks of anastrozole or fulvestrant have a 5-year RFS (recurrence free survival) of at least 95%. **Sample**
2. Ki67 >10% at 4-week, or 12-week or modified PEPI score of non-zero on neoadjuvant endocrine therapy predicts worse RFS compared to those with Ki67 ≤10% or PEPI of 0.
3. The degree of 4-week Ki67 suppression correlates with modified PEPI score and RFS.
4. Four week, or surgery Ki67 suppression compared to baseline differs between patients randomized to fulvestrant or fulvestrant in combination with anastrozole and those randomized to anastrozole.

15.1.2 Integral biomarkers:

Week 12 sample collection discontinued in Update #07 →

1. **Tumor Ki67 4 weeks (all patients) and 12 weeks (Arms I, II and III) on endocrine therapy**

Purpose: Tumor Ki67 value at 4-week and 12-week on therapy will determine whether a patient continues their assigned neoadjuvant endocrine therapy or switches treatment (to paclitaxel, another chemotherapy regimen or immediate surgery (see Section 6.4).

Tissue Acquisition: Tumor biopsies for Ki67 at week 4 is part of the mandatory tissue acquisition required for biomarker and correlative studies at week 4 that included 2 cores fixed in 10% formalin and 2 cores frozen in OCT and shipped to the Alliance WUSTL biorepository. Details on the procedures of tissue collection and shipments can be found in Section 7.0. The sample collection at week 12 is optional but follows the same procure for tissue collection and shipment (see Section 7.0).

Tissue Processing and Ki67 Analysis: Upon receipt of the specimen, the 2 fixed biopsy specimens (A & B) will be further processed for tumor Ki67 analysis at the CLIA certified Anatomic and Molecular Pathology Core Labs at Washington University (CLIA number 26D2013203) using the antibody against Ki67 (clone 30-9) and the Ventana Benchmark platform. Remaining tissue will be returned to the Alliance WUSTL biorepository at the completion of testing.

Ki67 Scoring and Reporting: Ki67 scoring will be performed as recommended by the International Ki67 in Breast Cancer Working Group [73], and the established scoring standard of procedure at Washington University with the result expressed as number of immunoreactive cells over among the total number of invasive cells scored. Ki67 will be reported to the treating physician and the patient real time as a quantitative/continuous variable.

Patients will continue on protocol therapy while awaiting analysis results. The results will be emailed and faxed to the CRA listed on the Biomarker Assay request Form that is sent with the tissue samples that are shipped to the Alliance WUSTL Biorepository.

Note: If the first biopsy at either 4-week or 12-week yields insufficient tumor cells for a Ki-67 determination, the patient may either: (1) continue on the assigned endocrine therapy and undergo second biopsy at week 12 for Ki67 determination; (2) proceed immediately with a second biopsy for Ki67 determination; (3) go off study treatment and enter the extended follow-up period (See Section 8.2.1).

2. Modified PEPI (Preoperative Prognostic Index) score determination (Arms I, II and III):

Purpose: The primary objective of the study is to prospectively validate the modified PEPI 0 score as a surrogate endpoint for success in long-term outcome such as RFS in ER+ breast cancer treated with neoadjuvant anastrozole or fulvestrant +/- anastrozole and to compare the modified PEPI 0 rate between neoadjuvant anastrozole and fulvestrant +/- anastrozole. In addition, modified PEPI 0 patients are recommended not to receive adjuvant chemotherapy and to complete 4.5 years of adjuvant endocrine therapy per her assigned treatment arm. Therefore, the modified PEPI score needs to be determined real time for each patient who completed neoadjuvant endocrine therapy on Arms I, II and III.

Method: Modified PEPI score is determined based on post-neoadjuvant endocrine therapy surgical staging (tumor size, lymph node status) and Ki67. The surgical staging will be obtained from surgical and pathology reports. Ki67 will be tested on surgical specimens centrally at the CLIA certified Anatomic and Molecular Pathology Core Labs at Barnes Jewish Hospital in St. Louis (CLIA number 26D2013203). The registering physician will be responsible for combining the information from the surgical pathology report and calculating the modified PEPI score.

Table 4 Score Determination

Surgical Specimen	PEPI Points	Modified PEPI points
**Tumor size**		
T1/2	0	0
T3/4	3	3
**Node status**		
Negative or N1mi	0	0
Positive	3	3
**Ki67 level**		
0-2.7%	0	0
>2.7-7.3%	1	1
>7.3-19.7%	1	1
19.7-53.1%	2	2
>53.1%	3	3
**ER, Allred score**		
0-2	3	
3-8	0	

Note that patients who have radiographic evidence of progression during neoadjuvant therapy who do not go on to surgery will be considered to have a Non-0 modified PEPI value.

Tissue acquisition of surgical specimen: Submission of representative unstained slides from the surgical resection specimen is required as described in Section 7.4 for this purpose.

Tissue Processing: Submitted tumor tissue slides will be further processed for Ki67 analysis at the AMP lab. In cases of inadequate specimens, the FFPE core independently collected at the time of surgery will be further processed.

Ki67 reporting for calculation of Modified PEPI Score: Ki67 will be reported to the registering physician within two weeks after receipt of the specimen. The registering physician will be responsible for combining Ki67 and pathologic T and N status from the surgical pathology report to derive the modified PEPI score. Patients with a modified PEPI score of 0 will be informed that their outcome is likely to be favorable enough that chemotherapy is unlikely to be of benefit. The use of adjuvant chemotherapy in this patient population will be recorded.

15.1.3 Integrated biomarker analyses

1) Baseline central ER testing (all patients):

There is a direct relationship between the degree of ER expression and tumor sensitivity to endocrine manipulation [74, 75]. Therefore, as in ACOSOG Z1031 [14], an ER Allred score of 6, 7 or 8 is required to be eligible for the study. However, the accuracy of ER testing can be affected by pre-analytical, analytical, and post-analytical factors, which results in an inaccurate test in at least 10% of cases in some reports. In the Z1031 study, 14% (26 of 185) patients had baseline ER Allred score of < 5 by central testing and would not have been eligible for the study [14]. We therefore propose to test ER centrally at the CLIA certified Anatomic and Molecular Pathology Core Labs at Barnes Jewish Hospital at St. Louis (CLIA number 26D2013203) as an integrated assay to correlate with outcome.

Tissue acquisition and shipment are detailed in Section 7.0 as part of the mandatory baseline tumor biopsy for biomarker and correlative studies that included 2 cores in 10% formalin and 2 frozen in OCT. Sample processing and analysis for ER IHC can be performed at the same time as the Ki67 analysis. The results of baseline ER testing will be sent to the Alliance Statistical Data Center only and not reported to the registering physician.

2) Baseline Ki67 and 4-week and surgery Ki67 suppression (all patients):

The objective is to assess whether the degree of tumor Ki67 suppression at week 4 is greater in women randomized to fulvestrant than that in those randomized to anastrozole and to correlate with PEPI 0 rate and recurrence free survival (RFS), so that it could be used as a primary endpoint in future endocrine trials.

Since the 4-week and surgery tumor Ki67 values are being examined in real time as an integral marker for treatment decision making, the baseline Ki67 testing must be integrated into the real time assessment for all patients enrolled on the trial to ensure that the methodology used on the baseline samples is the same as that used for the on-treatment Ki67 assessments.

Tissue acquisition and shipment procedures for baseline Ki67 are detailed in Section 7.0 as part of the mandatory baseline tumor biopsy for biomarker and correlative studies that included 2 cores in 10% formalin and 2 frozen in OCT. Ki67 analysis follows the same procedure as described above for the 4-week Ki67 evaluation. Results of baseline Ki67 testing will be sent to the Statistical Center only and not reported to the registering physician.

3) PEPI score determination (Arm I):

A secondary endpoint is to validate PEPI 0 score as a surrogate endpoint for success in long-term outcome such as RFS in ER+ breast cancer treated with neoadjuvant anastrozole. Therefore PEPI score is determined in patients who completed neoadjuvant anastrozole (Arm I). PEPI score is the sum of the point values of tumor size, nodal status, and Ki67 and ER value for surgical specimen (Table 4). Results of surgical ER testing will be sent to the Alliance Statistical Data Center for PEPI score calculation.

Tissue processing and Ki67 and PEPI score determination: Surgical tumor slides submission is required as described in Section 7.4 for this purpose. Upon receipt of the slides, future evaluation and process will be performed at the AMP lab. In cases of inadequate specimens, the FFPE core collected at the time of surgery will be further processed. ER expression is reported as a semi-quantitative/ordered categorical value (0-8) by using the Allred Scoring system. The Allred Score is calculated as the sum of an intensity score (range, 1 to 3) and a frequency score (range, 0 to 5), which is currently used in clinical practice. Ki67 scoring will be performed as described above, with the result expressed as number of immunoreactive cells among the total number of invasive cells scored. PEPI score is not reported to the treating physician.

4) Circulating tumor DNA

A secondary endpoint is to assess whether circulating tumor DNA (ctDNA) positivity following 4 weeks of NET is associated with tumor Ki67 response, mPEPI 0 and pCR follwing NET in those with week 4 Ki67 ≤ 10%, or RCB categories in response to neoadjuvant chemotherapy in those with week 4 Ki67 >10%. Additional exploratory endpoints including ctDNA positivity before and during neoadjuvant therapy, and during follow-up after surgery in association with short and long-term outcomes. The objectives, background and rationale, specimen collection and assay methods as well as statistical analysis plan are described in detail in Section 15.3 (Analysis of plasma circulating tumor DNA [ctDNA]).

5) NF1 Status

A secondary endpoint is to determine the impact of NF1 gene copy loss and stop/gain mutations on short- and long-term neoadjuvant/adjuvant endocrine therapy outcomes, with an exploratory end point to determine RCB profile in response to neoadjuvant chemotherapy in the setting of NF1-low endocrine resistant tumors triaged to neoadjuvant chemotherapy compared with tumors harboring normal NF1. The objectives, background and significance, assay information, and statistical plan are described in detail in Section 15.4.

15.2 Additional biomarker studies

1) Evaluation of tumor cells Ki67, TUNEL based apoptosis and other markers on day 2 tumor biopsies from patients treated with neoadjuvant weekly paclitaxel to explore for potential predictors of pCR/RCB-1 to paclitaxel

Hypothesis: Tumor cell apoptosis and a decrease in Ki67 on day 2 post paclitaxel therapy predict pCR/RCB-1. On treatment Ki67 levels have proven valuable for predicting long term response to aromatase inhibition but currently there are no proven surrogate endpoint biomarkers for paclitaxel. Since paclitaxel is both an antiproliferative agents as well as a cytotoxic agent, it is logical to assess markers of both cell death and the cell cycle within a time frame where the drug is exerting its maximal pharmacological effect.

Rationale and methodology: Paclitaxel promotes and induces mitotic arrest and subsequent apoptosis in vitro and in vivo [64, 65]. The kinetics of paclitaxel-induced miotic arrest and apoptosis has been determined in murine mammary carcinoma MCA-4 and ovarian carcinoma OCA-1 tumors [64]. Mice were treated with 60 mg/kg of taxol given once when tumors reach 8mm or twice, with the second dose being administered 3 days later. Tumors were histologically analyzed at various time points ranging from 1 to 96 hours after treatment to quantify mitotic and apoptotic activity. Mitotic arrest was visible at 1 hour, and increased with time to reach peak values of 36% in MCA-4 tumors and 22% in OCA-1 tumors at 9 hours, followed by a baseline of 1%-3% at 3 days for MCA-4 tumors and day 1 for OCA-tumors. Apoptosis occurred at later time points following mitotic arrest, beginning at the time of peak mitotic arrest (9 hours), increasing to the highest level of about 20% at 18-24 hours after treatment and gradually declining to the normal level of 3%-6-% after 3-4 days. Kinetic analysis performed after the second dose of taxol showed a considerably lower percentage of cells arrested in mitosis with minimum apoptosis compared to that from 1st dose. Therefore, it is rational to biopsy the tumor for apoptotic induction on day 2-3 post paclitaxel. This is supported by results from a neoadjuvant chemotherapy study by Stearns et al., in which tumor biopsies were performed at baseline and 2-3 days post the first dose of chemotherapy (paclitaxel or doxorubicin) and demonstrated an increase in tumor cell apoptosis on day 2-3 in those who achieved complete clinical response [76].

Week 12 sample collection discontinued in Update #07 →

Patients on paclitaxel in the neoadjuvant chemotherapy group will be consented for optional day 2 tumor biopsies following initiation of paclitaxel. FFPE tissue sectioning will be subjected to IHC analysis of Ki67, TUNEL based apoptotic assay and other markers of paclitaxel

molecular effects. We will follow the same Ki67 testing procedure as that used for samples obtained at baseline, and 4- or 12- week on endocrine therapy. TUNEL assay will be performed using the TumorTACSTM In Situ Apoptosis Detection Kit (Tregvigen). Since paclitaxel inhibits mitosis, other proteins of interest include markers of mitosis, such as pHistone H3, which we are routinely using in preclinical xenograft studies to monitor cell cycle effect of chemotherapy agents [77]. FFPE sections of breast cancer xenograft tumors treated with DNA damaging agents or vehicle [77] will be used as positive and negative control, respectively, at each run to ensure specificity of the assay. We will use the point-counting methodology to score these markers to avoid bias in the counting process.

Statistical approach: For each of these biomarkers, arrow plots of their changes at day 2 from pre-treatment levels versus pCR/RCB-1 (yes, no) will be performed. Each graph will be visually inspected for differences between those with a pCR/RCB-1 and those without. If appropriate (that is, depending upon the number of pCR/RCB-1 seen), Wilcoxon rank sum tests will be used to assess whether changes in the biomarkers at day 2 from pretreatment levels differ between those with a pCR/RCB1 and those without.

2) Genomic analysis of tumors mPEPI 0 vs mPEPI > 0 on neoadjuvant endocrine therapy

Hypothesis: We hypothesize that somatic mutation patterns in luminal-type breast cancer can be translated into etiology-based disease classifiers to guide the use of standard therapies (chemotherapy and endocrine therapy) and to identify disease subsets sensitive to experimental pharmacological interventions.

Specific Aims: Analysis of samples from the Z1031 trial provided an integrated analysis of whole genome and exome-based somatic mutation detection, gene-expression and gene copy profiles that identified molecular explanations for aromatase inhibitor-resistant proliferation [78]. Mutations in TP53 were associated with endocrine therapy resistance and poor prognosis Luminal B features, mutations in the stress kinase MAP3K1 with low proliferation and Luminal A features and mutations in GATA3 with increased responsiveness to aromatase inhibition. The TCGA breast project defined at least 24 additional significantly mutated genes (SMG) in ER+ HER2- breast cancer [79]. It is therefore the fundamental hypothesis for this ALTERNATE trial analysis that each SMG has the potential to have a positive or negative impact on endocrine therapy response and prognosis and that this knowledge can be translated into a clinically useful prognostic/predictive index.

Recently a role for ER translocation, point mutation and amplification in acquired resistance to endocrine therapy in the advanced disease setting [80]. This finding underscores a central problem with “maintenance” cancer treatments - ultimately efficacy is limited by resistance mutations in the pharmacological target. More effective elimination of disseminated cells in which these lethal resistance mutations occur is a logical and a potentially effective long-term strategy to improve outcomes for estrogen receptor positive (ER+) breast cancer. Conventional chemotherapy is used to achieve this therapeutic goal, but it is insufficiently effective for many patients. A critical step in improving outcomes is to define all the somatic mutations driving relapse in order to identify new therapeutic hypotheses that can be tested in clinical trials [81].

Three Specific Aims listed below will be performed with the objective to develop and validate etiology-based disease classifiers to guide the use of standard therapies (chemotherapy and endocrine therapy) and to identify disease subsets sensitive to experimental pharmacological interventions.

Specific Aim 1. To develop a somatic mutation-based classification of ER+ breast cancer that is predictive for endocrine therapy and chemotherapy responsiveness. Preliminary analysis of somatic mutations detected by massively parallel sequencing of baseline samples accrued from neoadjuvant endocrine therapy trials suggests a classification of luminal-type breast cancer that is based on the pattern of significantly mutated genes (SMG) which can have

either a favorable or unfavorable effect on disease prognosis/response [79]. We will therefore conduct further discovery sequencing on all tumors from the ALTERNATE trial. We will use these data to develop a genomic classification that we will interrogate against the neoadjuvant outcomes to uncover patterns that associated with response. The sequencing approach will initially focus on WES, but as the cost of WGS declines we will use deeper and more extensive sequencing techniques. Since WES has entered clinical laboratory use, and only uses 100ng DNA, we will initiate WES sequencing during the course of the ALTERATE study using this technique. WGS will be used in a more targeted way to address the deeper genomic architecture of extreme responders and non-responders.

Specific Aim 2. To utilize RNA seq. analysis to refine somatic mutation-based classification of ER+ breast cancer and to improve druggable genome analysis based on mutant allele expression. RNA seq. is a complementary technology to massively parallel DNA sequencing for somatic mutation detection, with the ability to detect gene fusions arising from translocations or other chromosomal rearrangements. As well as identifying in-frame fusions with novel functionality and possibly druggable properties, inversions and translocations also disrupt genes through out-of-frame fusion events. The detection of loss-of-function fusions will increase the accuracy of classifications based on tumor suppressor inactivation or disruption of DNA repair/damage response genes. Another advantage for RNA seq. is the ability to monitor expression from missense mutations, which are often of uncertain significance. RNA seq. analysis can therefore narrow the search for events that contribute to endocrine therapy resistance classifications and refine searches for druggable mutations [82, 83].

Specific Aim 3. To validate the integrated luminal breast cancer classifier in independent data sets. Genomic classifier exercises require a prospective validation plan because of data over-fitting during discovery. The data set provided by The Cancer Genome Atlas (TCGA) for breast cancer will provide a significant and independent resource. Approaching 1000 cases have already been subjected to a similar WES, gene copy number and RNA seq. analysis pipeline that we outline in our research plan. However, a significant disadvantage of the TCGA data set is that patients were not treated in a controlled fashion. We therefore will conduct a split validation analysis of samples from the ALTERNATE trial.

Specific Aim 4. To study resistance to endocrine therapy through DNA and RNA sequencing of tumor samples acquired during and after neoadjuvant treatment. To uncover resistance clones that have emerged or expanded under the pressure of endocrine therapy treatment, we will study clonality shifts in resistant tumors and compare on-treatment somatic mutation patterns in resistant tumors with that of sensitive tumors. We will also study evidence for treatment emergent mutations in genes that have been linked to resistance in primary tumors, or in sequence studies of advanced breast cancer.

Background and Rationale: The rationale for an integrated analysis of data based on combined DNA and RNA Sequencing is described below.

Classification of luminal-type breast cancer based on somatic mutation patterns. Exome sequencing using a capture reagent followed by massively parallel sequencing is a common technique in genomic investigations of cancer. However new technology has dramatically reduced the cost of whole genome sequencing, which allows an analysis of somatic mutations genome wide, which particularly facilitates the analysis of copy number, clonality and chromosomal rearrangements. Exome sequencing and analysis pipelines have featured in a number of TCGA publications; including breast cancer [80] and a variety of TCGA-developed analytical approaches are available. A standard initial approach has been to identify recurrently mutated genes using a significantly mutated gene (SMG) test that uses an assessment of the background mutation rate and gene size to determine whether somatic mutations are accumulating in any particular genes at a rate higher than would be expected by chance [84]. SMG analysis of TCGA data reveals that ER+ breast cancers harbor at least 26 recurrently mutated genes [80]. In contrast, the SMG list in ER- HER2- breast cancer is very short with only 3 genes reaching significance, PIK3CA, TP53 and RB. A similar very short list was seen in HER2+ disease, only PIK3CA, GATA3, TP53 and PTPN22 [80]. We postulate that the reason why ER+ HER2- disease has a long SMG list, and other breast cancer subtypes have a short SMG list is that these additional SMG reflect the biology of ER-driven tumors that is absent from the ER- and most HER2+ tumors and at least some directly deregulate ER function because our published studies establishes the principle that these ER+ breast cancer specific

genes can have positive or negative influences on endocrine response. MAP3K1 and mutations was associated with prognosis features such as low grade, luminal A status and GATA3 with greater responsive to aromatase inhibition. TP53 provided an example of the opposite effect, with an association with Luminal B status, high Ki67 and high grade[78] As a further potential example, the ER+ breast cancer SMG SF3B1, a gene encoding a protein in the spliceosome, is associated with chronic but not acute lymphocytic leukemia [85] and with good prognosis in uveal melanoma [85]. Thus we can postulate that SF3B1 mutant tumors might be associated with a favorable outcome. Similarly PIK3CA is associated with favorable prognosis, [86] (as long as, we suspect, TP53 is wt). Individually the frequency of these mutations is relatively low, however, and well-powered studies, like ALTERNATE, will be able to link genotype to phenotype even when the incidence of a mutation is relatively uncommon.

Classification approaches associated with mutation burden and mutation mechanisms. A weakness of an SMG-based classification is that there is a significant subset of tumors that do not harbor any of the genes on the SMG list and although many other mutations are usually present, their role in response to endocrine therapy is undetermined. There are, however, other ways to classify tumors using genomic information. Analyses by investigators at the Sanger Institute demonstrated that there are more than twenty different patterns of somatic mutation based on copy number aberrations and nucleotide substitution patterns, with a subset of these observed in breast cancer genomes (APOBEC, BRCA1/2, Age, Signature 8) [87]. One of the more striking findings is that some breast cancers display many more mutations than others [88]. Thus, we propose that even in the absence of a known resistance or sensitivity SMG to classify a luminal tumor, tumors with a high mutation burden are much more likely to have undergone a somatic event that induces endocrine therapy resistance and are therefore present a higher risk of relapse. It is also reasonable to postulate that tumors that are responsive to chemotherapy will also have a higher mutation burden, or display defects in DNA repair diagnosable through the somatic mutation pattern that sensitize tumors to cytotoxic chemotherapy. These phenotypes can be readily derived from WGS data and gene copy analysis.

RNA seq analysis. We have considerable experience of RNAseq for the detection of fusion genes and the assessment of variant allele frequency (VAF) [80, 89, 90]. As an example of the potential yield of this approach in the setting of endocrine therapy resistance, after detecting an ESR1/YAP1 fusion in an endocrine therapy resistant patient derived xenograft (PDX) [80] we examined the TCGA data for further evidence of ESR1 gene fusions and found 5% of LumB tumors harbored good evidence for the presence of translocations and local rearrangements around the ESR1 locus, producing N-terminal ESR1 truncations of varying sizes (depending on which exons of ESR1 were retained 5' of the fusion breakpoint) due to out-of-frame fusions. We also detected in-frame translocations with, for example POLH, PCDH11X and NOP2. We have experimentally evaluated some of these events and found that as long as the AF1 and DNA binding domains were retained before the fusion breakpoint (i.e. >N terminal 365aa), these mutations induce significant estradiol-independent proliferation. Of critical importance, these N terminal fragments do not have a ligand-binding domain and therefore their function cannot be antagonized by ligand-deprivation or anti-estrogen therapy. Thus, RNAseq detects fusion events affecting SMGs, thereby increasing the accuracy of an SMG-based classification. Regarding the detection of mutant allele expression by RNA seq, our PDX studies were again informative [80]. In a setting where the tumor is free of contaminating normal human DNA, mRNA expression could only be detected from 462 (44%) of the 1,056 validated, non-silent SNV identified by DNA sequencing. The expression of a mutation provides critical evidence that a mutation is druggable, illustrated by our published study of HER2 kinase and dimerization mutations that are currently the focus of a clinical trial [91].

Methodology and Statistical Plan: The data generation and analysis plan for each specific aim is described below.

Specific Aim 1. To develop a somatic mutation-based classification of ER+ breast cancer that is predictive for endocrine therapy and chemotherapy responsiveness. The expected total sample size of the trial is approximately 1475, which, will lead to 442 patients with mPEPI 0 and 1032 patients with mPEPI >0 according to an estimated 30% mPEPI0 rate based on the Z1031B trial. Conservatively considering 90% of all samples satisfy specimen and tumor yield quality requirements, we will sequence for the proposed correlative studies all the tumors from about 397 patients with mPEPI 0 and from 928 patients with mPEPI>0. All the tumors will be randomly divided in approximately equal size into a training set and a validation set, each of a total sample size of 429 at minimum and 598 at maximum, each encompassing approximately 141~195 (min~max) mPEPI 0 tumors and 288~398 (min~max) mPEPI >0 tumors. This sample size for both training and validation set (see sample size justification below) provides sufficient samples to develop a genomic signature that predict endocrine therapy responsiveness. As a secondary aim, within the training set, we will also dissect relationships between genomic features and the pathological stage-based components of the PEPI score (N and T stage) and the biological aspect (Ki67 measurements). For chemotherapy response classifiers, the low pCR rate in this population is clearly a statistical challenge (5% in Z1031B), but the protocol also stipulates the assessment of residual cancer burden (RCB) allowing a comparison of RCB-0 and 1 versus RCB-2 and 3, which, by including "near pCR" should generate a chemotherapy-sensitive group of 15%. This will allow us to compare 340 chemo resistant tumors with 60 chemotherapy sensitive tumors in two separate training and test sets of equal size [47]. As the price of genome sequencing continues to fall, we will extend our analysis to all the samples in the trial.

Data Generation and Computational Analysis: Methodology for specimen processing, WGS and WES analysis are described in our recent publications [14]. Standard mutation detection approaches will be conducted as outlined in our recent publication [82]. To estimate the sample purity and clonality to control for sample quality, we will use the WGS reads supporting the variant allele for each mutation from copy number neutral regions to perform clustering on this value in the tumor and normal data pair. We will define clusters using the SciClone (Miller et al., under review) for performing high dimensional clustering. To calculate the tumor/normal ratio for each sample, we take the median of the variant allele frequency of each point in the cluster, and assuming that these represent heterozygous mutations. Multiplication of the resulting number by two generates the estimated purity. As a control we also conduct this calculation for the variant allele frequency in the normal sample data to obtain an estimate of the amount of normal DNA in the sample as well. We will also make improvements to the MUSiC analysis package [92] to include the mutation signatures published by the Sanger group [88]. The long-term objective is to generate a version of MUSiC that provides medically useful output, rather than annotations that are of biological interest only, that can be "locked-down" for the validation step. A combined copy number analysis of the data set from which copy number neutral regions for clonality analysis will be identified and regions of gene gain and loss will be examined for inclusion in the final genomic classifier.

***Statistical Analysis**:* The two binary endpoints of interest are endocrine sensitivity and chemotherapy sensitivity. Two strategies will be adopted to develop somatic mutation and copy number amplification based classification algorithms. In the first tumors will be classified based on mutation burden SMGs, and amplification events. We will develop adjustments of the mutation burden estimate based on sample purity (see above) and coverage depth. The predictive ability of tumor burden will be analyzed by ROC analysis with each endpoint and the associated cut-points derived by maximizing the Youden index (sensitivity+specificity). In a second statistical-modeling oriented strategy, sophisticated statistical models will be

considered to train a binary endpoint with features including mutation burden, SMGs, amplification and other gene mutations (rare mutations/amplifications <5% in a group will be collapsed into meaningful biological units such as kinases, ontology terms, pathways, gene networks or biologically known good- or bad-prognosis gene categories etc). Features will be automatically selected while building predictive models. We will consider classification tree-based methods (random forest and classification and regression tree (CART) and the penalized logistic regression model (both are supervised methods as clinical endpoints are used for model building [93]. Tree-based methods, which take all types of data input (binary/categorical/continuous) and automatically identify optimal cut-points, classify samples similar to the biological strategy, but the goal is achieved by optimizing an objective target function rather than being guided solely by biological knowledge to split tumors into subsets with more or less homogeneous mutation patterns. A five-fold cross-validation procedure will be adopted during the supervised model-building step to reduce over-fitting. In the case where patients of similar clinical endpoint share few mutations, the unsupervised network-based stratification [94] of tumor mutations will be conducted where somatic mutation profiles and amplification will be smoothed over a molecular interaction network (such as STRING [95], HumanNet [96], PathwayCommons [97]), subtype prototypes will be derived by non-negative matrix factorization and patients will be assigned into groups of similar mutation profiles. The predicted response status (from the supervised models) and the assigned groups from the biological-knowledge based method and the network-based stratification will be tabulated with the true endpoint and their association will be examined by Fisher's exact test and logistic regression. Model performance summary statistics including area under ROC, sensitivity, specificity, positive and negative predictive values and Akaike information criterion (AIC), the Bayesian information criterion (BIC) will be calculated (the latter specific for logistic regression model). The models considered above will be taken forward to next aim for model refining with addition of RNA-seq. markers.

Sample size Justification.

The expected total sample size of the trial of 1475 will lead to 442 patients with mPEPI 0 and 1032 patients with mPEPI >0 according to an estimated 30% mPEPI 0 rate based on the Z1031B trial. Conservatively considering 90% of all samples satisfy specimen and tumor yield quality requirements, we will sequence for the proposed correlative studies all the tumors from about 282~390 (min~max) patients with mPEPI 0 and from 576~796 (min~max) patients with mPEPI >0. All the tumors will be randomly divided in approximately equal size into a training set and a validation set, each of a total sample size of 429 at minimum and 598 at maximum, each encompassing approximately 141~195 (min~max) mPEPI 0 tumors and 288~398 (min~max) mPEPI >0 tumors. According to extensive simulation results for a binary endpoint in a logistic regression framework in Polley et al. [98], a study of a total sample size of 400 provides at least 80% power when the misclassification rate is <35% (i.e., an overall accuracy of 65%) with a type-I error rate of 5% or lower while the best power is usually achieved when samples were equally split into a training and validation set. For the minimum sample size of 429 in the training set, the table below provides power calculations at a 5% alpha level when varying P0 the conditional probability that mPEPI is truly 0 given that the genomic signature predicts mPEPI as being >0 (aka, 1-negative predictive value) in the range of 0.1~0.6 and an odds ratio in the range of 1.5~2, while P1 the conditional probability of mPEPI being 0 given that the prediction is 0 (aka, positive predictive value) can be correspondingly calculated. Except for scenario at the 1st row, all the scenarios provide at last 83% power. Since we expect a much higher PPV than considered in the table (minimum PPV of 0.143 and a maximum PPV of 0.516), the minimum sample size of 429 provides enough power on building a logistic regression based classifier.

Power	N	P0	P1	Odds ratio
0.71208	429	0.1	0.143	1.5
0.8316	429	0.1	0.151	1.60.90942
0.90942	429	0.1	0.159	1.7
0.95471	429	0.1	0.167	1.8
0.97874	429	0.1	0.174	1.9
0.99054	429	0.1	0.182	2
0.91914	429	0.2	0.273	1.5
0.97344	429	0.2	0.286	1.6
0.99258	429	0.2	0.298	1.7
0.99819	429	0.2	0.31	1.8
0.99961	429	0.2	0.322	1.9
0.99992	429	0.2	0.333	2
0.97052	429	0.3	0.391	1.5
0.99381	429	0.3	0.407	1.6
0.99895	429	0.3	0.421	1.7
0.99985	429	0.3	0.435	1.8
0.99998	429	0.3	0.449	1.9
1	429	0.3	0.462	2
0.98439	429	0.4	0.5	1.5
0.99752	429	0.4	0.516	1.6
0.99969	429	0.4	0.531	1.7
0.99997	429	0.4	0.545	1.8
1	429	0.4	0.559	1.9
1	429	0.4	0.571	2
0.98743	429	0.5	0.6	1.5
0.99818	429	0.5	0.615	1.6
0.9998	429	0.5	0.63	1.7
0.99998	429	0.5	0.643	1.8
1	429	0.5	0.655	1.9

1	429	0.5	0.667	2
0.98439	429	0.6	0.692	1.5
0.99752	429	0.6	0.706	1.6
0.99969	429	0.6	0.718	1.7
0.99997	429	0.6	0.73	1.8
1	429	0.6	0.74	1.9
1	429	0.6	0.75	2

The sample size for the validation set was justified under the objective to determine the concordance between predictions generated by the genomic signature against the true mPEPI 0 status. The first level of achieve the objective is to estimate a desirable sensitivity and also a desirable specificity of the predictions within a pre-specified precision and the final sample size will be the larger one from the calculations for sensitivity and specificity respectively. Conservatively assuming that the genomic signature leads to a sensitivity and specificity both at least 70% when used to predict the mPEPI 0 status and the mPEPI rate is 30%. A total of 320 and 158 patients will be needed in the training set to estimate a sensitivity of 0.7 and a specificity of 0.7 within a 5% margin of error with a 97.5% confidence (alpha level=2.5%), respectively, while the required total number of patients reduces to 138 and 68, respectively, if both increase to 0.8 [99]. The second level to achieve the objective is to compare the estimated sensitivity (specificity) to random chance. A total of 280 patients (with 93 mPEPI 0) allows 98% power on conducting the one-sample hypothesis testings H0: Sensitivity=0.5 (random chance) vs. H1: Sensitivity=0.7 with a 2.5% type-I error rate and a total of 138 patients allows 98% power for the hypothesis testing H0: Specificity=0.5 vs. H1: Specificity=0.7 based on normal approximation [100].

In summary, the trial should provide enough samples on for signature development and signature validation purposes in the proposed correlative study.

Specific Aim 2. To utilize RNA seq. analysis to refine somatic mutation-based classification and to improve druggable genome analysis based on mutant allele expression.

Data Generation and Computational Analysis: The samples for RNA analysis will be the same as Aim 1 processed for DNA sequencing. The RNA-Seq pipeline first conducts quality control using FastQC (http://www.bioinformatics.bbsrc.ac.uk/projects/fastqc/) and Picard CollectRnaSeqMetrics (http://picard.sourceforge.net/). Next we will leverage existing publicly available tools for mapping (Bowtie), [101] assembly (TopHat) [102], and transcript quantification (Cufflinks) [103]. For gene fusion discovery we will use ChimeraScan [90] (developed in the Maher lab), which we have previously used to identify ESR1 gene fusions [80]. Given our preliminary evidence of ESR1 fusions, we will first utilize the low quality ChimeraScan gene fusion predictions to increase our sensitivity for detecting ESR1 fusions that have even weak evidence supporting a fusion event. However, for *de novo* gene fusion discovery we will focus on the high quality filtered ChimeraScan output. Given the challenge of prioritizing “driver” events from private non-specific fusion events, we will prioritize candidates that are either (i) recurrent across samples, (ii) a gene (and even the same exons) found to be recurrently altered across samples with different partners suggesting functional recurrence (analogous to the ESR1 fusions), (iii) fusion genes involving a SMG, and (iv) fusion events involving a gene predicted to have a drug interaction. In order to determine if a gene

has a drug interaction we intend to leverage a database developed at The Genome Institute, DGIdb, that is the aggregation of curating numerous existing drug-gene interaction resources [83]. Mutations identified by DNA sequencing will be considered 'supported' by the transcriptome data if one or more variant supporting reads are identified. The variant will be considered to 'expressed' if the variant has a read count greater than 5 and the gene is expressed (FPKM >1). A variant is considered to have mutant biased expression if the variant is expressed and the variant allele frequency is greater than 20% higher in the RNA-seq data compared to the WGS and exome sequencing data. A variant is considered to have wild type biased expression if the gene is expressed, the region of the variant is covered at 5X or greater depth, and the VAF is at least 20% lower in the RNA-seq data compared to the WGS and exome sequencing data. Similar to our gene fusion analysis, priority will be given to expressed variants within gene found to have existing drug interactions as found in DGIdb.

Statistical analysis. The RNA seq aim uses the same patient samples as the DNA analysis but will integrate RNA-seq. data to screen for mutated genes which are expressed as potential druggable targets and to elucidate relationships between somatic mutation and mRNA gene expression. The goal of this aim is to refine the classification algorithms developed by somatic mutation detection by incorporating gene fusion and differential gene expression data. Fisher's exact test will be used to examine association between somatic mutations and RNA-seq gene fusions. Differential expression (DE) analysis will be performed, between somatic mutations and gene expression, and between resistant/sensitive tumors and gene expression. DE analysis will be conducted in two manners: (1) the Bioconductor packages including DEseq [104] and edgeR [105] will be directly applied to raw counts of RNA-seq in the unit of genes or pathways. Both packages model raw counts based on the negative binomial (NB) distribution which is recommended for suitable RNA-Seq DE analysis to handle the well-known over-dispersion problem while relevant variables can be adjusted and the multiple testing issue will be handled; (2) the RNA-seq raw counts of genes/pathways will first be normalized and DE analysis will be proceeded using conventional microarray DE methods/softwares. We will implement various normalization methods, including FPKM (**F**ragments **P**er **K**ilobase of exon per **M**illion fragments) and log2-counts per million (logCPM) as adopted by the BioConductor limma package [106] which normalize read counts using the edgeR NB-based model. The significance analysis of microarray (SAM) [107] will be applied to the normalized gene expression data in the context of conventional two-class comparison and false discovery rate adjusted p-values will be returned through permutations. The limma package will be used for linear model fitting and response group contrasting as well as multiple gene set testing (mean-rank gene set enrichment, ROAST and Camera [108-110]

<u>Predictive Models.</u> Predictive models have been described in details in Aim 1 and will be conducted in a similar manner here in Aim 2, except with incorporation of gene fusion and gene expression in the FPKM format or the standardized logged gene expression estimated from edgeR and DEseq to facilitate model validation. The refined models with addition of RNA-seq data will be compared to the previous models based on diagnostic test summary statistics as listed in Aim 1.

<u>Sample size Justification</u>. We assume that adding RNA-seq data will increase predictive power in models developed from Aim 1 and thus power is expected to be greater than Aim 1.

Pitfalls and Problems*:* The pairing of every DNA data set with a RNAseq data set may not be possible in every case due to sample constraints. We estimate, based on our initial experience, no more than 5% of the cases analyzed will not be paired.

Specific Aim 3. To validate the integrated luminal breast cancer classifier in independent data sets. Model Validation will be provided by an independent cohort from the ALTERNATE trial, for both the chemotherapy sensitivity question, as well as the endocrine sensitivity index

based on SMG and mutation burden/type. With such a large trial we will be able to easily generate an equivalent sized cohort as the training set, approximately 141~195 (min~max) mPEPI 0 tumors and 288~398 (min~max) mPEPI>0 tumors.

Computational analysis. We will file a validation plan with the NCI to "lock-down" our algorithms before we embark on the validation set analysis.

Statistical analysis. Aim 3 will use 400 independent patient samples from ALTERNATE trial. We also expect that other data sets will emerge over the next 5 years that we can take advantage of at the validation stage.

Predictive Model Validation. The expression of genes selected into predictive models will be examined on distribution for potential batch effect by graphical displays (principal component analysis and hierarchical clustering) and statistical tests. The predictive models developed previously in Aim 1 and Aim 2 will be applied to data (total mutation burden, somatic mutation, amplification, gene fusion and gene expression) on the independent validation samples to predict response in a single sample predictor manner while accounting for potential batch effect as elaborated below.

Batch effects and single sample predictor development. Specimens from the ALTERNATE trial will be sequenced in a first-come-first-sequence order focused on samples with relevant clinical information (e.g., treatment arm, treatment response, PEPI status, successful on treatment Ki67 analysis). Conventional QC metrics applied by Baylor genome sequencing center will include read depth, sequence alignment rate, exonic and intronic mutation rate, rRNA and duplication rates. The purpose is to apply a quality control (QC) pass/failure filter before analysis. Samples that pass QC will be randomized into a training set and a validation set each of a sample size of approximately 141~195 (min~max) mPEPI 0 tumors and 288~398 (min~max) mPEPI>0 tumors. The randomization ensures that ALTERNATE patient samples in the training and validation are not profiled in completely separate batches but are instead admixed to avoid confounding between bias introduced by sample processing and the biological effect of interest. We recognize the final number of cases in the two groups is subject to sample availability and sample quality and may have to be adjusted, but in principle the two cohorts should be of similar size. The analysis of the training set will commence as soon as data become available. All necessary clinical data mainly endocrine sensitivity and chemotherapy sensitivity, including drug assignment will be provided to analysis for supervised predictive model building while the clinical outcomes associated with validation samples will be blinded to the statisticians who are in charge of predictive model building and prediction. A final predictive model built using the training samples will be locked down to predict each new independent validation sample one case at a time using a single sample predictor (SSP) approach. Although samples are randomized into a training and validation set, potential unmeasured batch effect may still exist and affect the applicability and performance of the predictive model in the validation samples. To tackle batch effect in the training samples, the surrogate variable analysis (SVA) method [111] will be applied using the BioConductor package "sva" [112, 113]. Probability weights of each gene being associated with unmeasured confounders will be iteratively estimated using the expression data of the training samples. The latent factors representing unmeasured batch variables will be estimated from the probability weighted expression data by singular value decomposition (SVD), and the associated coefficients will be estimated from linear modeling using the training samples with incorporation of the biological effect of interest, known batch variables and the estimated unmeasured/latent batch effect variables. The cleaned expression data of the training samples, after adjustment for potential unmeasured batch variables (namely, the residuals from subtracting the unmeasured confounders) will be used for predictive model building as described in details in Aim 1. The predictive model and the estimated probability weights on

genes will be locked down for response prediction on the validation samples. To handle potential batch effect in validation samples when applying the predictive model as a SSP to an independent validation sample, the frozen surrogate variable analysis (fSVA) method [111] will be adopted using the BioConductor package "sva" [112, 113]. Specifically, the normalized gene expression data of each new validation sample will separately be concatenated to the data of the training samples. Using the probability weight matrix estimated in the training stage, the latent factors representing unmeasured batch effects which are associated with a new sample will be estimated via SVD on the concatenated expression data of the training samples and a new validation sample. The cleaned expression data of a new sample will subsequently be obtained as residuals subtracting the effect of the estimated unmeasured batch variables. To the end, the final predictive model will be applied to the cleaned expression data of a new sample for treatment response prediction. To confirm the predictive performance of the SSP, predicted outcomes from the SSPwill be associated with true endocrine-sensitivity outcome for diagnostic test summary measures (sensitivity, specificity, positive and negative predictive value) and associated with survival endpoints by the KM method and log rank test, as described in the training set analysis section. With respect to relapse free survival all patients from the POL trial, Z1031 trial and the ALTERNATE trial will be followed for 10 years. The POL/Z1031 data set will have 5 years of median follow up by 2015.

Computational analysis. We will file a validation plan with the NCI to "lock-down" our algorithms before we embark on the validation set analysis.

Statistical analysis. Aim 3 will use 400 independent patient samples from ALTERNATE trial. We also expect that other data sets will emerge over the next 5 years that we can take advantage of at the validation stage.

<u>Predictive Model Validation</u>. The expression of genes selected into predictive models will be examined on distribution for potential batch effect by graphical displays (principal component analysis and hierarchical clustering) and statistical tests; if batch effect exists, a simple transformation will be performed to adjust the new samples to have the same mean and standard deviation as in training samples for each gene and other more sophisticated statistical methods including ComBat and edgeR [114, 115]. The predictive models developed previously in Aim 1 and Aim 2 will be applied to data (total mutation burden, somatic mutation, amplification, gene fusion and gene expression) on the independent samples to predict outcome. The predicted outcomes from each model will each be associated with true endocrine-sensitivity outcome as described in the training set analysis to validate and the models' predictive ability. With respect to relapse free survival all patients from the POL trial, Z1031 trial and the ALTERNATE trial will be followed for 10 years. The POL/Z1031 data set will have 5 years of median follow up by 2015.

Specific Aim 4. To study resistance to endocrine therapy through an analysis of tumor samples acquired during and after neoadjuvant treatment.

Breast cancer is a multi-clonal disease, with somatic mutations present in minor clones becoming dominant over time because they are driving resistance to treatment. For example, it is now well established that somatic mutations in ESR1, particularly those at positions Y537 and N538, cause ligand-independent activation of the receptor [116, 117]. These mutations are present at very low frequencies at baseline (1%) but can be present in at least 10% of tumors after the development of resistance to several lines of endocrine treatment [116, 117]. Sequencing of DNA and RNA from tumors exposed to neoadjuvant endocrine treatment is therefore likely to uncover resistance mutations, not only in ESR1, but also more widely in other genes that play a role in acquired resistance. Identifying these mutations and developing druggable hypotheses before the onset of overt clinical resistance could eventually improve outcomes for patients otherwise destined to relapse. The use of fulvestrant in ALTERNATE is

a critical consideration because ER-down regulation may circumvent, or prevent, the development of resistance by opposing the action of ESR1 mutations that remain sensitive to SERD treatment [116, 117]. To investigate these questions we will apply WGS, and RNAseq (using the pipeline described above) to DNA and RNA acquired from post treatment samples, drawing equally from resistant and sensitive cases. We will use SciClone [118] and other software approaches to study clonality shifts in (Ki67-defined) resistant tumors and compare on-treatment somatic mutation patterns with sensitive tumors. To validate these findings we will use targeted sequencing approaches with customized capture reagents (that will include ESR1) to generate deep coverage of potential resistance alleles to quantify the variant allele frequencies of treatment emergent somatic mutations uncovered by discovery sequencing (as well as by other groups). The sample size for this analysis will depend on sample availability but we intend to analyze all resistant tumors (Ki67 elevated in post treatment samples) available to the sequencing pipeline and match them to tumors where the Ki67 continues to be suppressed. We will also conduct DNA and RNA analysis on paraffin embedded material obtained from patients triaged to neoadjuvant chemotherapy, to study the response of the ER+ genome to chemotherapy exposure. These will be exploratory studies, but in principle we will be able to contrast the effect of chemotherapy versus endocrine therapy on mutational signatures and variant allele fractions.

3) Additional correlative sciences studies

An amendment or proposal for any additional correlative science studies to be performed on biological samples will be submitted to CTEP, NCI for review and approval according to NCTN guidelines. Amendments to the protocol and/or proposals for use of biological samples will include the appropriate background, experimental plans with assay details, and a detailed statistical section. Samples for testing will not be released for testing until the appropriate NCI approvals have been obtained.

15.3 Analysis of plasma circulating tumor DNA (ctDNA)

15.3.1 Background and Rationale

In recent years, advances in ctDNA technology has allowed the detection of tumor DNA non-invasively with high sensitivity and accuracy[119-121]. In the metastatic setting, assessment of ctDNA has become an attractive approach, to complement tissue based analysis, for the identification of therapeutic target, resistance mechanisms and response monitoring due to the advantage of capturing the molecular heterogeneity of different metastatic sites and the ability of serial sampling to monitor dynamic adaptations[119-121]. Various commercial targeted panels are available for clinical use for patients with metastatic breast cancer[122].

ctDNA levels are usually lower in patients with early stage disease than those with metastatic disease, which poses substantial challenges. However, several studies have demonstrated that personalized ctDNA analysis panel designed based on tumor tissue sequencing allows high sensitivity of ctDNA detection in patients with early stage disease as well as minimum residual disease (MRD) or molecular relapse before clinical evidence of metastasis[123-132].

Signatera test is a personalized ultra-deep sequencing (average >100,000X) ctDNA detection assay designed to target 16 patient-unique somatic variants selected from whole exome of the individual patient's cancer [124, 133-135]. Signatera received FDA "Breakthrough Device" designation for post-surgical relapse detection through the quantification of ctDNA in the blood of patients previously diagnosed with cancer. In a study of 49 patients with early stage breast cancer, including stage I (2%), II (31%) and III (67%), recruited after surgery, who underwent serial plasma collection every 6 months for up to 4 years, signatera test demonstrated high sensitivity (89%) and specificity (100%) for relapse prediction with a lead time of up to 2 years[124]. In the subgroup of 34 patients with a history of HR+/HER2- breast

cancer, the sensitivity for relapse prediction was 82% with a lead time of 301 days [124], demonstrating its ability to detect MRD in this patient population. However, this study was limited by the small sample size and included patients with various breast cancer subtypes.

Early changes in ctDNA by signatera test have recently been shown to predict response to neoadjuvant chemotherapy (NAC) in patients with high-risk early stage breast cancer (30% T3 or T4 tumors; 47% node positive and 61% MammaPrint High 2) enrolled in the I-SPY 2 trial[132]. In this study that included 84 patients (35% HR+/HER2-, 23% HER2+, 43% TNBC), personalized ctDNA assay (signatera test) designed based on whole-exome sequencing of primary breast cancer was used to analyze plasma samples collected at pretreatment (T0), 3 weeks after initiation of treatment (T1), between paclitaxel and anthracycline regimens (T2), and after NAC prior to surgery (T3). At pretreatment (T0), 73% of the patients had detectable ctDNA. ctDNA positive rate was significantly higher among HER2+ (84%) and TNBC (86%) subtypes as compared with the HR+/HER2- (48%) subtype ($P < 0.01$). ctDNA positivity was also associated with larger tumors (T3/T4, 91%, $P = 0.014$) and MammaPrint High 2 compared to MammaPrint High 1 ($P <0.01$). ctDNA positivity decreased during the course of NAC, from 73% before treatment (T0), to 35% at 3 weeks (T1), to 14% at the inter-regimen time point (T2), and down to 9% after NAC (T3). Clearance of ctDNA at T1 was associated with a significantly higher pCR rate post NAC (48% vs 17%, $p = 0.012$). Patients who did not clear ctDNA at T3 had a significantly higher risk of metastatic recurrence (HR 22.4; 95%, CI 2.5-201, $P < 0.001$). These data indicate the potential utility of ctDNA response in monitoring NAC response.

None of the previous studies investigated ctDNA changes during neoadjuvant endocrine therapy (NET) and its association with endocrine therapy response such as Ki67, PEPI score, pathologic stage and long term outcomes. This is important since if confirmed, on-treatment tumor biopsy for Ki67 could potentially be replaced by minimally invasive blood draw for ctDNA and patients with high risk cancer could be identified early in the course of therapy for tailored therapeutic approaches.

In addition, none of the previous studies have tested the utility of ctDNA detection 5 years after surgery in predicting late recurrence. Disease recurrence following 5 years of adjuvant endocrine therapy occurs at a steady rate of 1-3% per year in the subsequent years in patients with HR+ breast cancer [136]. Continuing endocrine therapy beyond 5 years has shown a small benefit but with associated side effects [137-139]. Biomarkers that identify patients at greater risk for late relapse are in great need. Plasma in cancer patients often carries small amounts of fragmented cell-free DNA of 160-180 base pairs, which are originated from the necrosis or apoptotic process of cancer cells. Advances in the next generation sequencing (NGS) technology and digital genomic techniques support the clinical validity of cell-free circulating DNA (ctDNA) sequencing analysis to non-invasively identify actionable genomic alterations, monitor treatment response, and investigate resistance mechanisms [140]. In a prospective cohort of 55 patients with early stage breast cancer of various subtypes receiving neoadjuvant chemotherapy, detection of mutations in ctDNA in plasma after completion of surgery, either at a single postsurgical time point or with serial follow-up samples, not at diagnosis, predicted metastatic relapse with high accuracy [127], with a median lead time of 7.9 months. In addition, serial monitoring of the mutation variant allele frequencies of the ctDNA identified potential lethal clones that drove the recurrence [127]. However, the study was limited by the small sample size and included patients with various breast cancer subtypes.

The ALTERNATE trial, with the ongoing tumor genomic analysis and long term follow-up of all patients enrolled to the study, offers a unique opportunity to examine the role of ctDNA analysis in assessing ET sensitivity, disease monitoring, and predicting metastatic recurrence including late recurrence in individual patients. Assessment of ctDNA mutation profiles at

metastatic recurrence will assist with the identification of driver mechanisms of recurrence and genomic evolution of HR+/HER2- breast cancer on ET. We hypothesized that 1) Pre-treatment ctDNA detection is associated with high risk clinical, pathologic features as well as genomic characteristics; 2) ctDNA dynamics during NET predicts Ki67 responsive and mPEPI score and long term outcome; 3) Detection of ctDNA during follow up and at 5 years following curative surgery forecasts recurrence; 4) ctDNA mutation profile at recurrence captures driver events of metastatic recurrence, by comparing dynamics of tumor tissue mutation profiles during neoadjuvant therapy.

15.3.2 Experimental Approach

15.3.2.1 Specimens and Methods

Tumor tissue collected pre-treatment and/or surgery time point will be subjected to whole exome sequencing. Data from whole exome sequencing will be used for the design of personalized ctDNA detection assay as previously described[124, 132]. The presence of ctDNA will be tested for in the following samples:

- Neoadjuvant period from all consented patients: (plasma from 10 cc EDTA tube):

  Pre-treatment; Week 4; Surgery

- Adjuvant period (plasma from Streck tubes):
  - All consented patients: 5 years (+/- 6 months) post-surgery and at recurrence
  - High-risk patients (mPEPI ≥4 or if switched to chemo due to Ki67 >10% at wk 4 or 12 on NET): At postop visit (2-8 wks post-surgery), yearly (+/- 6 months) for years 1-10 post-surgery or until recurrence, and at recurrence

A table that lists the blood sample collection time points for each of the ctDNA objectives is provided below.

Protocol	Objectives	Blood collection tube/ time point
2.2 #8	To assess whether women with ctDNA present after 4 weeks of NET is less likely to achieve mPEPI 0 or pCR among those with week 4 Ki67 ≤ 10% and continued on NET	EDTA blood/Wk 4
2.2 #9	To examine whether the proportion of women with ctDNA present at week 4 differs between those with week 4 Ki67 >10% on NET and those with week 4 Ki67 ≤ 10%	EDTA blood/Wk 4
2.2 #10	To assess whether RCB class differs with respect to the presence of ctDNA after week 4 NET among those with a week 4 Ki67 levels > 10% who switched to neo-adjuvant chemotherapy.	EDTA blood/Wk 4
2.3 #12 (a)	To examine the association between the presence of pre-NET ctDNA and each of the following patient and disease characteristics: age, race, body mass index, cTstage, cN stage, pre-NET Ki67, tumor grade, histology, breast cancer intrinsic subtype, gene expression or mutation profiles, week 4 Ki67 levels ≤ 10% , week 4 Ki67 ≤ 2.7% (complete cell cycle arrest)	EDTA blood/pre-NET
2.3 #12 (b)	To assess whether the presence of pre-NET ctDNA decreases the likelihood to achieve mPEPI 0 + pCR among patients with week 4 Ki67 levels ≤ 10% who completed NET or subsequently discontinued NET due to disease progression	EDTA blood/pre-NET

2.3 #12 (c)	To estimate the proportion of women who maintain ctDNA positivity or attain ctDNA positivity after completion of 4 weeks, or after 24 weeks of NET	EDTA blood/Wk 4 and surgery
2.3 #12 (d)	To assess whether the presence of ctDNA at completion of NET decreases the duration of breast cancer-free interval among patients with week 4 Ki67 levels ≤ 10% who completed NET	EDTA blood/surgery
2.3 #12 (e)	To assess whether the duration of breast cancer-free interval is decreased in those with ctDNA present at week 4 NET among patients with a week 4 Ki67 levels > 10% who switched to neo-adjuvant chemotherapy	EDTA blood/Wk 4
2.3 #12 (f)	To assess whether RCB class or duration of breast cancer-free interval differs with respect to the presence of ctDNA at the completion of NCT among those with a week 4 Ki67 levels > 10% who switched to NCT	EDTA blood/surgery
2.3 #12 (g)	To determine the sensitivity, specificity, and lead-time interval for ctDNA detection during follow up after surgery for distant disease recurrence among high risk patients	Streck tube blood/follow up after surgery then yearly in the high risk group)
2.3 #12 (h)	To assess the ctDNA positivity rate at 5 years after surgery and its association with late recurrence among women with a week 4 Ki67 levels ≤ 10% who completed NET	Streck tube blood/5 years after surgery
2.3 #12 (i)	To assess the ctDNA positivity rate at 5 years after surgery and its association with late recurrence among women with a week 4 Ki67 levels > 10% who switched to neoadjuvant chemotherapy	Streck tube blood/5 years after surgery
2.3 #12 (j)	To examine changes in ctDNA quantity over time up to surgery during neoadjuvant therapy among patients with week 4 Ki67 >10%, those with week 4 Ki67 ≤ 10%, and mPEPI and RCB categories	EDTA blood/Pre-NET, Wk 4 and surgery
2.3 #12 (k)	To compare mutation profiles of ctDNA at metastatic recurrence with persistent/emerging mutations in tumor tissues at surgery post neoadjuvant therapy to identify driver mechanisms of recurrence	Streck tube blood/at recurrence

15.3.2.2 Description of the Assay and Cutpoint for ctDNA Positivity

The assay platform to be performed is Natera, Inc's Signatera assay. Analytes used in this platform are tumor DNA isolated from frozen tumor tissue, germline DNA isolated from whole blood, and cell-free DNA isolated from plasma. Briefly--tumor DNA and germline DNA will be sequenced; tumor-specific variants are identified and prioritized using a combination of variant callers in collaboration with Natera; a tumor-specific 16-plex PCR assay is designed for each case per Natera's standard operating procedures; cfDNA from patient plasma is interrogated for the presence of tumor DNA by subjecting it to targeted amplification with the 16-plex assay and sequencing at Natera, using their reagents and standard operating procedures [124, 132].

For both the tissue workflow (tumor DNA and matched germline DNA) and the plasma workflow (cfDNA isolated from plasma) NGS sequencing data are acquired. These data are quantitative and are processed to identify sequencing reads that correspond to either the germline reference genome or the tumor-specific variants. Scoring of the sequence reads is performed using Natera's proprietary algorithm. Presence or absence of tumor DNA in the plasma sample is established and mean tumor molecules per milliliter of plasma is calculated and reported.

The Signatera test requires 2 targets to be above the confidence threshold to call a sample positive for ctDNA, because this increases the specificity of the assay. As stated in Coombes et al. 2019 [124], Specificity was estimated to be 99.71% for individual mutation detection. Specificity for a 16-plex assay would thus be only 95.5% if a single measurement above the threshold was used to call the plasma sample ctDNA positive. In order to achieve high specificity of > 99.8% we require 2 mutations to be measured above the confidence threshold as previously described [124].

15.3.2.3 Analytical Performance of the Assay

Natera's Signatera assay has been analytically validated (Research Use Only) and CLIA validated. RUO sensitivity, precision, and ranges have been published [124, 133-135]. For the plasma workflow, negative in-line controls and positive sequencing controls are included in every assay plate and sequencing run. Critical variables include tumor percentage in the frozen tumor sections, DNA yield from tumor DNA and plasma, and sequencing QC metrics such as depth of read and error rate.

15.3.2.4 Statistical Analysis Plan: Secondary Objectives 8-10

-To assess whether women with ctDNA present after 4 weeks of NET is less likely to achieve mPEPI 0 or pCR among those with week 4 Ki67 ≤ 10% who continued NET.

This analysis will exclude those patients for whom it could not be determined whether the patient's mPEPI score was 0 or non-zero due to missing pT stage, pN stage, or Ki67 in residual specimen. Patients who discontinued NET for reasons other than progression will also be excluded.

Among the 926 women who had a Ki67 ≤ 10% and completed NET or discontinued due to disease progression, there were 268 women who had either a mPEPI 0 score or a pCR (ACSO 2020 oral presentation [141]). The table below provides the likelihood under a number of scenarios that a two sided Fisher's exact test at a significance level of 0.05 of detecting that proportion of women with ctDNA present at week 4 among those with PEPI 0 or pCR is less than the proportion of women with ctDNA present at week 4 among those with PEPI 1-9 or who progressed on NET.

Proportion of women with ctDNA present at week 4 among those with PEPI 0 or pCR (n=268)	Proportion of women with ctDNA present at week 4 among those with PEPI 1-9 or who progressed on NET (n=658)	Power
0.20	0.30<br>0.35	87.1%<br>99.6%
0.25	0.35<br>0.40	83.2%<br>99.2%
0.30	0.40<br>0.45	80.2%<br>98.9%

-To assess whether RCB class differs with respect to the presence of ctDNA after week 4 NET differs among between those with a week 4 Ki67 levels > 10% who switched to neo-adjuvant chemotherapy

There were 23 women with a week 4 Ki67 levels > 10% who switched to neo-adjuvant chemotherapy whose surgical pathology findings were a pCR or RCB class I. There were 123 women with a week 4 Ki67 levels > 10% who switched to neo-adjuvant chemotherapy whose surgical pathology findings were RCB class II-III. (SABCS 2020 oral presentation[142]).

The table below provides the likelihood under a number of scenarios that a two sided Fisher's exact test at a significance level of 0.05 of detecting that proportion of women with ctDNA present at week 4 among those with a week 4 Ki67 levels > 10% who switched to neo-adjuvant chemotherapy whose surgical pathology findings were a pCR or RCB class I is less than the proportion of women with ctDNA present at week 4 among those with a week 4 Ki67 levels > 10% who switched to neo-adjuvant chemotherapy whose surgical pathology findings were RCB class II-III.

Proportion of women with ctDNA present at week 4 among those with pCR or RCB I after switching to NCT (n=23)	Proportion of women with ctDNA present at week 4 among those with pCR or RCB I after switching to NCT (n=123)	Power
0.25	0.60<br>0.65	84.6%<br>93.9%
0.35	0.70<br>0.75	84.9%<br>94.2%
0.45	0.80<br>0.85	88.2%<br>96.1%

-To estimate whether the proportion of women with ctDNA present differ at week 4 between those with week 4 Ki67 >10% on NET and those with week 4 Ki67 ≤ 10%

There were 267 women with a week 4 Ki67 levels > 10% and 928 women with a week 4 Ki67 levels ≤ 10%.

The table below provides the likelihood under a number of scenarios that a two-sided Fisher's exact test at a significance level of 0.05 of detecting that proportion of women with ctDNA present at week 4 among those with a week 4 Ki67 levels ≤ 10% is less than the proportion of women with ctDNA present at week 4 among those with a week 4 Ki67 levels > 10%.

Proportion of women with ctDNA present at week 4 among those with week 4 Ki67 ≤ 10% (n=928)	Proportion of women with ctDNA present at week 4 among those with week 4 Ki67 > 10% (n=267)	Power
0.20	0.30<br>0.35	90.7%<br>99.8%
0.25	0.35<br>0.40	87.3%<br>99.5%
0.30	0.40<br>0.45	84.4%<br>99.3%

15.3.2.5 Statistical Analysis Plan: Exploratory Objective 12

Logistic regression modeling will be used to assess whether presence of pre-NET ctDNA differs with respect to age, race, body mass index, cTstage, cN stage, pre-NET Ki67 levels ≤ 15%, tumor grade, histology, breast cancer intrinsic subtype, gene expression or mutation profiles among all eligible patients who began protocol treatment.

For all eligible patients who began protocol treatment who have a week 4 Ki67 result: Fisher's exact test will be used to assess whether the proportion of women with week 4 Ki67 levels ≤ 10% differs between those with ctDNA present prior to treatment and those who do not. Fisher's exact test will be also used to assess whether the proportion of women with week 4 Ki67 levels ≤ 2.7% differs between those with ctDNA present prior to treatment and those who do not.

For all eligible women with a week 4 Ki67 levels ≤ 10% who completed NET or discontinued due to disease progression, a Fisher's exact test will be used to assess whether mPEPI 0 + pCR rate differs with respect to the presence of ctDNA prior to NET. Also, a 95% binomial confidence interval will be constructed for the difference in mPEPI 0 + pCR rate between those with ctDNA present prior to NET and those who do not have ctDNA present prior to NET.

For all eligible patients who started protocol treatment, a 95% binomial confidence interval for the proportion of women who maintain ctDNA positivity or attain ctDNA positivity after completion of 4 weeks NET will be constructed overall and by treatment arm.

For all eligible women with a week 4 Ki67 levels ≤ 10% who completed NET, a 95% binomial confidence interval for the proportion of women who maintain ctDNA positivity or attain ctDNA positivity after completion of 24 weeks NET will be constructed overall and by treatment arm.

For all eligible patients with a week 4 Ki67 levels > 10% who switched to neo-adjuvant chemotherapy and completed surgery, Gray's test will be used to assess whether the cumulative incidence of a breast cancer event post-surgery differs with respect to ctDNA status after 4 weeks of NET or at completion of NCT.

Women in the high risk group (defined by modified PEPI score of 4 or more, or patients who undergo neoadjuvant chemotherapy due to 4-week or 12-week Ki67 over 10%) who do not have ctDNA present in the first post-surgical blood sample drawn will comprise the cohort to examine the sensitivity, specificity, and lead-time interval for ctDNA detection after surgery for distant disease recurrence. Women with ctDNA present at their post-surgical blood draw will not be included in these analyses as we do not know when they converted to ctDNA positive. An estimate of proportion of women with no ctDNA present prior to or at the time metastatic disease is diagnosed will be determined (with its associated 95% confidence interval) and an estimate of proportion of women with ctDNA present prior to or at the time metastatic disease is diagnosed will be determined (with its associated 95% confidence interval). The lag from ctDNA presence first detected to diagnosis of metastatic disease will be determined.

For all eligible women with a week 4 Ki67 levels ≤ 10% who completed NET that have remained event-free for 5 years post surgery and have a ctDNA results 5 years post-surgery, Fine and Gray's competing risk regression modeling will be used to assess whether breast event-free interval 5 years after surgery differs with respect to whether ctDNA is present 5 years post surgery adjusting for PEPI score (0, 1-3, 4-9).

A Wilcoxon rank-sum test will be used to assess whether the percent change in amount of ctDNA at completion of NCT from pre-NCT levels differs between those with RCB class 0-1 and those with RCB class 2-3. A Spearman rank correlation coefficient will be used to assess the association between the percent change in amount of ctDNA after 4 weeks of NET from pre-NET levels and the precent change in Ki67 after weeks of NET from pre-NET levels. A

Kruskal Wallis test will be used to assess whether the percent change in amount of ctDNA at completion of NET from week 4 levels differs with respect to mPEPI risk category (where low risk group included those with a is pCR or mPEPI 0 score; immediate risk group includes those with a mPEPI 1-3 score, and the high risk group includes those with mPEPI 4-9 score).

15.4 Analysis of NF1 Status

15.4.1 Objectives for the analysis of NF1 status

The overall goal of assessing the NF1 status in the ALTERNATE trial is to advance new treatment approaches for early stage ER+ breast cancer [143]. The primary objective is to determine the impact of NF1 gene copy loss and stop/gain mutations on the short and long-term neoadjuvant endocrine therapy outcomes for ER positive and HER2 negative breast cancer. We hypothesize that NF1 loss, detected by whole exome sequencing (WES), is associated with intrinsic endocrine therapy resistance. Consequently, loss of NF1 in the pre-treatment specimen predicts poor neoadjuvant outcomes and increased risk of metastatic disease for patients with ER positive and HER2 negative breast cancer [144, 145]. WES also facilitates the discovery of other causes of intrinsic endocrine therapy resistance driven by somatic mutation. While NF1 gene copy loss and stop gain mutations are present in 17% of ER+ HER2- primary breast cancers, and therefore represent a common driver of poor outcomes, additional explanations for primary endocrine therapy resistance must be defined in order to develop a suite of diagnostic tests that can accurately guide therapy. Since tumor DNA is extracted using protein sparing techniques, the ALTERNATE trial samples also provide a rich resource for other analyses that can assist in this goal. For example, mass spectrometry-based proteomic analyses conducted by the NCI supported Clinical Proteogenomic Tumor Analysis Consortium (CPTAC) are planned. Here, WES is critical for data analysis, since proteomics provides an opportunity to understand the functional consequences of complex somatic alterations such as NF1 loss (proteogenomics) [146]. Indeed, we are currently proposing ALTERNATE samples for the next phase of CPTAC funding, where there is a priority for clinical trial sample analyses in settings where genomic data is already available. WES also facilitates the identification of somatic mutations for the development of sensitive circulating tumor DNA tests for relapse detection. Thus, while this application is focused on detecting the clinical significance of somatic NF1 as the primary objective, the proposed WES analysis will also provide a deep resource to address multiple secondary hypotheses and exploratory analyses.

15.4.2 Background and Rationale

This project centers on the tumor suppressor NF1/neurofibromin which represses Ras signaling by acting as a GTPase activating protein (GAP). We have recently discovered that NF1 also has a GAP-independent function as a transcriptional co-repressor for estrogen receptor α [147]. When NF1 is disrupted two oncogenic pathways are simultaneously activated to promote the signaling cross-talk that is responsible for endocrine therapy resistance and poor outcomes. Critically, NF1 loss creates specific therapeutic vulnerabilities that are under investigation. For example, preclinical studies suggest that ER+ NF1low breast cancers are resistant to tamoxifen and aromatase inhibition but may remain responsive to selective estrogen receptor degraders (SERD), such as fulvestrant. The ALTERNATE trial examined differences in efficacy between anastrozole, fulvestrant or the combination, providing an opportunity to address the endocrine response profile of NF1low tumors in a clinical setting. There are many oral SERDs in development but no clear biomarkers that delineate which tumors are differentially responsive to SERD versus AI. Our proposed analysis would therefore further our knowledge of the SERD responsiveness of early-stage breast cancer. Furthermore, our preclinical findings on ER+ NF1low tumors form the basis for a current NCI ComboMatch trial that will investigate

the combination of the MEK inhibitor, binimetinib, and fulvestrant for the treatment of advanced ER+ HER2- NF1low breast cancer. If this advanced disease trial were successful, we could propose to bring this combination to the early disease setting for ER+ HER2- NF1low tumors. On this basis we propose to investigate the hypothesis that NF1-deficiency, due to genomic deletion or stop-gain mutation, plays a critical role in endocrine therapy resistance. A determination of copy number (CN) loss/stop-gain mutation in METABRIC and in a preliminary WES analysis of subset of ALTERNATE samples indicates that 17% of ER+ HER2- breast cancers have shallow deletions in NF1. In the METABRIC database [148, 149] NF1 shallow deletion and stop-gain mutations (which co-occur) is associated with elevated relapse risk (P=5.2xe-6). Our primary aim is to replicate this finding in the ALTERNATE trial. In a preliminary WES analysis of 301 ALTERNATE trial samples (about one third of the eventual sample size), 31% of cases with Ki67 values above 10% at week 4 (the protocol definition of intrinsic resistance) had an NF1 deletion, versus 12.5% of tumors with a Ki67 value of below 10% (P=0.0005). Thus, we have established that tumors with intrinsic endocrine therapy resistance in ALTERNATE are highly enriched for NF1 genomic loss. However, we need to analyze all available samples to determine if there is a differential SERD versus AI response. Since tumors with week 4 Ki67 values of >10% are triaged to chemotherapy in ALTERNATE, we will also be able to determine the degree to which NF1 deleted tumors are sensitive to standard of care treatments for high risk ER+ breast cancer.

15.4.3 Experimental Approach

15.4.3.1 Specimens and Methods

Analyte	Assay	Specimen source/requirement(s)	Time of specimen collection	Time of specimen analysis
Tumor DNA from samples with tumor content >50%<br>Matched normal DNA	Matched pair tumor/normal whole exome sequencing	Tumor tissue: frozen core biopsies at baseline<br><br>Normal DNA from the peripheral blood	Pretreatment tumor tissue.<br><br>Blood specimens for germline DNA also taken before treatment	Whole exome sequencing (WES) will be completed non-real time after sample collection.

15.4.3.2 Describe the expected distribution of the biomarker in the study population

Endocrine Sensitivity	Resistant	Sensitive	Total
NF1 cnv loss	24	28	52
NF1 cnv intact/gain	54	195	249
Total	78	223	301
301/329 sequenced cases had week 4 Ki67 data available			p=0.0005

Table 1. A contingency table demonstrating a significant interaction between NF1 CN loss and intrinsic endocrine therapy resistance (Ki67 >10% after 4wk of neoadjuvant treatment)

In 301 ALTERNATE samples with WES data described in Table 1, the incidence of NF1 genomic loss was 52/301=17.3%, which is consistent with our METABRIC analysis. The incidence of NF1 CNV loss, in endocrine resistant tumors was 24/78 (31%), while in sensitive tumors NF1 was lost in 28/223 cases, (12.5%). Hence, in this preliminary data, the incidence of tumors with NF1 allele loss in primary ET resistant tumors is 2.5 times that observed in primary endocrine therapy sensitive tumors (p=0.0005). As of June 1, 2021, 301 baseline paired tumor and blood normal WES analyses are now complete and there are 619 baseline WES candidate samples with tumor

content >50% for which 450 have tumor and normal DNA already prepared and 169 are currently in tumor tissue processing. All samples will be available for analysis.

The effect of NF1 deletion on NF1 mRNA expression is shown in Figure 2 below.

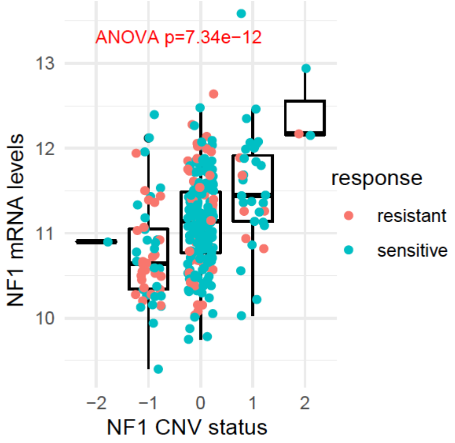

Figure 2. Association between NF1 gene copy status and NF1 mRNA levels. Red dots are endocrine therapy resistance cases and blue dots

Cut-points: The somatic variants such as NF1 shallow deletion will be called using paired tumor and blood normal from BAM files. Somatic CN alterations will be predicted by applying copywriteR algorithm on blood normal (control) and tumor (sample) BAM files. Hg19.UCSC.add_miR.140312.refgene will used to map the CN information to genes. GITSIC2.0 threshold of +/-0.3 will be applied to identify NF1 gain or loss of CN respectively. Hence, CN estimates for NF1 for tumors will be made by comparing against matched normal to provide somatic CN estimations as CN log2 ratio (continuous variable) as well as GISTIC thresholded categories. The integer values range from −2 to 2, where 0 means no amplification or deletion of magnitude greater than the threshold parameters described above. Deletions are represented by negative numbers: −1 means deletion beyond the threshold (shallow heterozygous deletion); −2 means deletions greater than the minimum arm-level CN observed in the sample (homozygous deletion).

Based on estimations obtained from preliminary data from ALTERNATE and METABRIC, **17%** of ER+/HER2- tumors will qualify the criteria of NF1 loss (Shallow deletion; denoted by GISTIC2.0 CNA = - 1). The expected incidence of tumors with NF1 shallow deletion in primary endocrine therapy resistant tumors is 2.5 times that observed in primary endocrine therapy sensitive tumors.

15.4.3.2 Description of the Assay

a. The assay: Tumor DNA will be extracted from fresh-frozen biopsies and matched germline DNA from blood samples will be subjected to WES at Baylor College of Medicine Human Genome Sequencing Center (HGSC) (Appendix 17.4). WES data will be generated for unique DNA samples using the Illumina platform. For this, paired-end libraries will be constructed as described previously [150] with the following modifications. Samples will be barcoded at ligation step using Illumina unique dual barcodes adapters (Cat# 20022370) and will be amplified 6-8 cycles using the Library Amplification Ready-mix containing KAPA HiFi DNA Polymerase (Kapa Biosystems, Inc). For capture enrichment, libraries will be pooled in equimolar ratios in groups of 10 and will be hybridized in solution to the HGSC VCRome 2.1 design [151]. To this design, exome coverage across >3,500 clinically relevant genes that are previously <20X (~2.72Mb) will be supplemented. Enriched libraries will be sequenced on the NovaSeq 6000 instrument using the S4 reagent kit (300 cycles) to generate 2x150bp paired-end reads. For these DNA samples, on average, 11.01 Gb of unique sequence data will be generated with 97.3% of the bases in the exome design coverage to 20x read depth or greater.

b. Describe the specimens and anticipated methods for specimen acquisition, fixation/stabilization and processing. Tumor biopsies using a 14G core needle were taken at baseline (pre-neoadjuvant treatment), 4-week and surgery, which included two cores frozen in separate OCT blocks. These samples are processed using our published protocol which provides DNA, RNA and protein for down-stream analysis [152]. As part of the tissue processing approach, multiple 5um sections are obtained (after every 6th 50um section) to ensure that tumor content exceeds 50%. This is critical for assays that address CN loss, as too much normal DNA reduces sensitivity. Whole blood was collected and stabilized in an EDTA tube. Matched germline DNA is immediately extracted at the Alliance tissue bank when the tube is received.

c. Describe the scoring procedures and type of data to be acquired. Details of CN estimation has been provided in the assay description above. In summary, NF1 CNV loss is a discrete variable determined using a standard CN algorithm (CopywriteR). This software implements somatic CNV calling using sample (tumor) and control (matched blood normal). The CNV estimation is followed by GISTIC2.0 [153] which provides sensitive and confident localization of the targets of focal somatic copy-number alterations. GITSIC2.0 threshold of +/-0.3 will be applied to identify NF1 gain or loss of CN respectively.

15.4.3.3 Statistical plan

a. Identify the clinical endpoints and the biomarker measurements involved in the analysis:

1) Primary analysis: To establish relationships between NF1 CN loss, resistance to neoadjuvant endocrine therapy and relapse-free survival
2) Co-primary analysis: Integrate NF1 CN loss into the PEPI score to determine whether the addition of a genomic element provides independent prognostic information, thereby improving the PEPI model [8, 154].
3) To determine the RCB profile to chemotherapy in the setting of NF1-low endocrine therapy resistant tumors triaged to neoadjuvant chemotherapy compared with tumors with normal NF1 CN. For patients triaged to chemotherapy, residual disease burden rates according to NF1 CN (low versus normal)

b. Specify the case selection method if only a subset of patients will be included in the biomarker evaluation.

There will be no case selection. Patients who have met eligibility criteria for ALTERNATE clinical trial, provided genome consent and have a sample with at least 50% tumor content will be included in these analyses (as lower tumor content obscures the ability to observe single copy loss).

c. Justify the numbers of patients to be studied and biomarker assays/tests to be performed
The sample size justification is provided in Section D with the statistical analysis plan. The biomarker is based on an NF1 mutation/Deletion algorithm based on matched normal/tumor analysis described in the methods.

The sample size justification is provided in Section D with the statistical analysis plan. The biomarker is based on an NF1 mutation/Deletion algorithm based on matched normal/tumor analysis described in the methods.

d. Describe the statistical analysis methodology and underlying assumptions.

The proportion of post-menopausal women with clinical stage II-III ER+ Her2- breast cancer treated neo-adjuvant endocrine therapy who had a week 4 ki67 > 10% was 22%. If NF1 CN results are available for 950 of these women and the proportion of patients with NF1 CN loss is 17%, then we would expect 0.17 x (proportion of patients with NF1 CN loss who had a week 4 ki67 > 10%) + 0.83 x (proportion of patients without NF1 CN loss who had a week 4 ki67 > 10%)=0.22. The table below provides a number of scenarios for which a two sample test of proportions, with significance level of 0.05 would have at least a 85% likelihood of detecting a difference of 12% or more in proportion of patient with a the week 4 ki67 > 10% between those with NF1 CN loss and those without NF1 CN loss.

proportion of patients with NF1 CN loss who had a week 4 ki67 > 10%	proportion of patients with no NF1 CN loss who had a week 4 ki67 > 10%	power
0.50	0.165	99.9%
0.40	0.185	99.9%
0.35	0.196	97.7%
0.325	0.201	89.7%
0.30	0.206	69.7%

The proportion of post-menopausal women with clinical stage II-III ER+ Her2- breast cancer treated neo-adjuvant endocrine therapy who had a PEPI 0 or pCR was 20.7%. Using similar argument as above, a two sample test of proportions, with significance level of 0.05 would have at least a 83% likelihood of detecting a difference of 11% in proportion of patient with a PEPI 0 or pCR between those with NF1 CN loss and those without NF1 CN loss, when the proportion of patients with a PEPI 0 or pCR among those with NF1 CN loss is 30%. Within the METABRIC cohort of post-menopausal women with ER+, HER2 N+, the 5-year RFS was 70% among those with NF1 CN loss and 80% among those without NFI CN loss. The ALTERNATE study [143] was designed to have an enrollment period of 75 months and a follow-up period after the close of enrollment of 48 months (the trial was closed about 18 months ago and so at this point the follow up period is about 30 months away). Assuming the 5-year RFS rate among the patients who completed NAI and surgery (regardless of PEPI score) is 90% and 17% of the patient with have NF1 CN loss, a two sided log rank test achieves 84% power at a 0.050 significance level to detect a hazard ratio of 0.5131, when the 5-year PFS rate among those with NF1 CN loss is 80%. The expected number of events is 122. Cox modelling or Fine-Gray competing risk modelling will be used to assess whether RFS differs with respect to NF1 CN loss after accounting for treatment arm and other known prognostic factors.

For the clinical endpoints of the proportion of patients with a week 4 ki67 > 10% among those with NF1 low tumors and the proportion of patients with endocrine sensitive disease among those with NF1 low tumors, the following analyses will be carried out. Multivariate logistic regression modeling will be used to assess whether the given endpoint differs with respect to treatment arm, after accounting for known prognostic factors. If sample sizes prohibit such an approach, then for each of the three possible pairwise comparisons between treatment arms, a 98% confidence interval for the difference in two independent proportions will be constructed. There were 168 patients with week 4 ki67 >10% who chose to switch to neoadjuvant chemotherapy. Approximately 14% of these patients had a RCB index of 0-1. A Fisher's exact test will be used to assess whether the proportion of patient with a RCB index of 0-1 differs with respect to whether the patient had a NF1 low tumor.

For the exploratory analysis regarding mRNA NF1 levels versus NF1 gene copy loss co-occurrence of NF1 gene copy loss with lower expression level will be investigated. Based on partial cohort sequencing, 40% of NF1 low cases (tumors with lowest quartile of NF1 mRNA levels) also have NF1 CN loss (single copy loss) In contrast, only 13% of NF1 high cases (tumors with NF1 mrna > lowest quartile of NF1 mRNA levels) had CN loss. While CN loss cases are enriched in NF1 low cases (Fisher's exact, p=0.0001), an integrative NF1 loss predictive model using information from genomic and transcriptomic profiling will be investigated as a predictor of endocrine therapy response. Each will be examined separately versus the outcomes described above and then also in combination.

15.5 Clinical Proteomic Tumor Analysis Consortium (CPTAC) analysis of ALTERNATE samples

15.5.1 Preliminary data

The prognostic value of tumor proliferation measurements (either mRNA analysis or Ki67 IHC) prompted an examination of cell cycle regulators in HER2- tumors from the Clinical Proteomic Tumor Analysis Consortium (CPTAC) breast cancer prospective data[146]. While the CPTAC dataset has no outcome data, it does generate support for this protocol amendment to address cell cycle mechanics in ALTERNATE trial samples using tandem mass tag (TMT)-based proteomics. The cell cycle is initiated by activation of the cyclin dependent kinases CDK4 and CDK6, which phosphorylate and inhibit the retinoblastoma-associated transcriptional co-repressor RB[155, 156]. Relieving RB repression allows E2F transcription factors to activate expression from gene products that promote progression from G1 to S,

including cyclin E (CCNE1). In turn, CCNE1 activates CDK2, which further phosphorylates RB, as well as other factors regulating progression through S phase. Unsupervised clustering was used to determine how RB phosphorylation (pRB) levels (G1/S transition), CDK2 substrate phosphorylation levels (late G1/S and S-phase progression), and E2F regulated genes (RB de-repression) relate to PAM50-based intrinsic subtypes, defined through RNA-seq analysis. This analysis generated three clusters: Cluster 1 (CDK2-) is almost exclusively LumA, with lower levels of E2F-driven gene expression, consistent with lower proliferation. Furthermore, CDK2 substrate phosphorylation levels (reflective of CDK2 activity) are also low, suggesting the transition into S phase is suppressed. However, pRB and CDK4 substrate phosphorylation levels vary widely within Cluster 1 and correlate well with E2F target gene expression (Spearman p=5.2e-6), suggesting that despite reduced overall relative proliferation in LumA cases, G1/S transition is accelerated in some cases, potentially decoupling pRB from ER activity, thereby driving endocrine therapy resistance. In contrast, Clusters 2 and 3 have highly active E2F-driven transcription with high CDK2 substrate phosphorylation; however, they differ remarkably by pRB levels. Cluster 2 (pRB-, CDK2+) has suppressed pRB (and RB protein levels, data not shown) and therefore must have a compromised G1/S checkpoint. As such, loss of RB is considered to be a marker for chemotherapy sensitivity, since the DNA damage response is compromised when there is no G1/S checkpoint[157, 158]. Consistent with this hypothesis, most of these pRB low tumors are PAM50 basal-like, a more chemotherapy sensitive subtype. However, it is noteworthy that there was two LumB tumors in Cluster 2 with convincingly low pRB levels and high CDK2 activity. Cluster 3 (pRB+, CDK2+) is comprised of a mix of poor prognosis subtypes where pRB, E2F target transcription, and CDK2 activity are all high. Interestingly, there are 4 LumA cases in cluster 3, and one in Cluster 2, suggesting misclassification, as these tumors clearly have features associated with a high proliferation state, which is not a LumA characteristic. A subsequent examination revealed that immune stimulatory proteins were higher in cluster 2, also suggesting greater chemotherapy sensitivity for this group[159, 160].

15.5.2 Baylor Proteogenomics Methods and Capacity

The opportunity to include proteomics in our analysis of ALTERNATE samples is because we have used protein-sparing DNA extraction protocols. Thus, the proteomics objectives do not consume resources because the analysis is using material that is otherwise destroyed during typical organics-based extraction of DNA. Our genomics, proteomics, phosphoproteomics, affinity, and targeted SureQuant data generation and analysis protocols, as well as the performance and standards for quality control meet CPTAC Standards. We have fully adhered to the current CPTAC guidelines for standardization of our proteogenomics pipeline and have capacity to actively participate in further improvements of assay performance as mandated by the future CPTAC4 scientific collective. For the two objectives described in the clinical arm, we plan 25 TMTPro discovery multiplexes for the first 3 years. These will cover the 200 LumA ALTERNATE samples (100 patients, pre/post neoadjuvant therapy) for the endocrine resistance objective and 120 non-LumA baseline tumors with 30 matching one month on-ET biopsies associated with the ALTERNATE patients who went on to get chemotherapy. The discovery samples will also undergo matching SureQuant targeted assays, originally for testing pRB-based, immunology-based targeted panels to address specific hypothesis described below. Each panel will have the primary analyte (e.g., pRB), as well as several additional pathway proteins and tumor content normalizers. If successful in predicting response, either through single or multi-analyte predictors, these SureQuant panels will also be performed on approximately 400 independent samples drawn for validation cohorts. A balanced subset of resistant and sensitive samples of a similar size will also undergo Kinase inhibitor Pulldown Assays (SureKiP) with the specific goal of characterizing alternative repurposed or new pharmaceutical vulnerabilities in resistant tumors. Our group has capacity to perform and fully

analyze up to 15 TMT multiplexes per year on an Eclipse Tribrid instrument for the clinical arm of PTRC, leaving room for troubleshooting if necessary. Separately, there is a dedicated instrument effort on the EvoSep One-Exploris 480 platform for targeted analyses, which fully covers the proposed targeted assay throughput, as these assays are short single sample runs. Furthermore, we have experienced personnel to develop up to 100 new SureQuant assays per year for new discovery-informed targeted panels, envisioned as iterative improvements of diagnostic signatures that are based on analyses of TMT discovery data.

15.5.3 Biomarkers for endocrine therapy response in PAM50 LumA cases

The ALTERNATE NET trial represents an ideal opportunity for proteogenomics-based discovery science. For LumA tumors, endocrine drugs are the mainstay of treatment, and the pCR to chemotherapy is minimal[161]. For example, there was not a single pCR event in 26 LumA tumors triaged to chemotherapy due to a high on-treatment Ki67 value (>10%). As an alternative to chemotherapy, recent data indicates benefit for adjuvant therapy with a CDK4/6 inhibitor. However, eligibility for the MonarchE adjuvant therapy study included Ki67 levels above 20% (more typical of a LumB tumor)[162]. Thus, the emerging question for LumA breast cancers concerns which tumors have sufficient endocrine therapy resistance to warrant additional treatment besides standard chemotherapy. To address this, we will study the relationship between endocrine therapy response in LumA tumors and the phosphorylation level of the major substrate for CDK4/6, the retinoblastoma protein RB1, following our preliminary findings in the CPTAC prospective breast cancer analysis. We will first split the set of LumA specimens from ALTERNATE into a discovery (100 patients) and confirmatory (200 patients) sets, balanced in terms of treatment assignment, percentage with baseline Ki67 > 15%, and clinical stage. TMT-based proteomics and phosphoproteomics will be used on the training specimens to determine the relationship between baseline pRB levels, mPEPI status and Ki67 inhibition at one month and surgery. It is possible, for example, that a high baseline pRB level is predictive of adaptive resistance, only evident after 6 months of treatment. We will use ROC statistics to determine whether there is a threshold level pRB that identifies a LumA tumor that is exhibiting higher levels endocrine therapy resistance at surgery[163]. If we are able to identify a threshold effect, we will develop a pRB SureQuant assay to several RB peptides and use the confirmatory specimen set and stratified logistic regression modeling to assess their association with endocrine resistance.

15.5.4 Analysis of pre- and one-month post-treatment pairs

A critical advantage of our data set is the relatively large number of pre- and on-treatment (one month) pairs we have accrued to study the mechanisms of differential endocrine therapy response and to develop predictive biomarkers that incorporate treatment effects rather than simply measuring pathway or analyte status at baseline. The TMT-based discovery set will therefore be chosen to have an adequate matched one month on-treatment specimen to compare the effect of endocrine treatment on pRB levels and signaling more broadly. We will test whether pRB at one month or the change from baseline to one month is more predictive of acquired resistance (Ki67 levels) at surgery. We will further analyze pre- and 4 week on-treatment paired data to identify treatment-altered mRNAs, proteins, phosphosites, and pathways. Differential analysis will be performed using Wilcoxon rank-sum tests (for unpaired samples) as well as Wilcoxon signed-rank tests (for pre-post paired samples). Signed log p-values derived from Wilcoxon p-values and fold-change between pre and post treatment samples will be used to identify enriched Hallmark and KEGG pathways. Pathway-centric analysis will be performed using Gene Set Enrichment Analysis (GSEA) with WebGestalt[164] from Dr. Zhang's group and single sample GSEA/post-translational modification set enrichment analysis (ssGSEA2.0/PTM-SEA) from the Broad Institute[165]. To test the feasibility of using samples from clinical trial biopsies for pairwise analysis, we

performed proteomics profiling and analysis using 20 pre-treatment samples from 15 patients and 16 on-treatment samples from 10 patients accrued from a multicenter phase II clinical trial that enrolled post-menopausal women to neoadjuvant letrozole[25]. As expected, we found significantly lower levels (False Discovery Rate <0.05) of proteins associated with the cell cycle and estrogen response in post-treatment samples than in pre-treatment samples (data not shown). In the pre- and post-treatment samples from ALTERNATE trial we will pay particular attention to pathways that are activated uniquely in treatment resistant cases and relate these to other aspects of the proteogenomic profiles, including potential DNA defects reflected in COSMIC signatures, mutation load and expression of DNA repair components, including mismatch repair and nucleotide excision repair, following up on our earlier CPTAC study[146]. These analyses will generate hypotheses on endocrine therapy resistance mechanisms to study in model systems (preclinical arm below).

15.5.5 Biomarkers for neoadjuvant chemotherapy response in non-LumA cases

The use of chemotherapy in high stage and/or high biological risk breast cancer is nearly ubiquitous, and chemotherapy regimens are essentially the same regardless of subtype. However, pCR rates vary dramatically according to PAM50 intrinsic subtype. For basal-like breast cancer, the pCR rate is 40% to 50%. The rate for LumB breast cancer is less than half that. For example, for patients triaged to chemotherapy in the ALTERNATE trial, pCR rate amongst PAM50 LumB cancers was only 3/49 (6%). In the proteogenomic data presented in **Figure 3**, we provide evidence that, analogous to our unpublished TNBC findings, loss of the DNA ligase LIG1 could correlate with lack of response to chemotherapy in LumB as well. We have also developed a FISH assay for LIG1 somatic loss that can be applied to formalin-fixed sections as a validation exercise. We have also previously demonstrated that loss of single-strand DNA (ssDNA) break repair can be causal to endocrine therapy resistance[166] which, unlike homologous recombination repair[167], may not be chemotherapy sensitizing[168]. Most LumB tumors demonstrate high levels of immune checkpoint components, e.g., IDO1 and LAG3[169], but the interaction between these factors and chemotherapy efficacy is incompletely understood. Thus, the causes of chemotherapy sensitivity or resistance across breast cancer involve complex interactions between different biological processes, including defects in DNA repair and host immune responses. We recognize that our ability to study the proteogenomics of chemotherapy response in ALTERNATE trial samples is limited by the relatively small number of cases triaged to chemotherapy and the rarity of pCR events. We have therefore designed a study that also draws samples from our institutional Abeyance Pathology protocol. Here, we collect snap frozen OCT embedded samples during standard of care diagnostic biopsies. This approach has many advantages: samples are accrued in an ongoing basis; we can accrue samples from under-represented minorities where clinical trial accrual rates are low, and we can collaborate with other NCI funded programs to increase sample size or identify relatively uncommon responders - like ER+ breast cancers associated with pCR. Thus, our analysis plan is to construct a TMT-based chemotherapy response proteogenomics discovery set that will be co-analyzed with the ALTERNATE LumA discovery set through the use of a common reference. The neoadjuvant chemotherapy discovery set will comprise 120 HER2- cases, half LumB and half non-Luminal. For the ALTERNATE samples that contribute to this sample set, we will also analyze the matched on-treatment samples, when available, to complete the endocrine therapy perturbation analysis described above. The estimated total size for this study will therefore be approximately 150. The Abeyance Pathology program will continue throughout the course of this proposed PTRC funding period with institutional and Baylor Breast SPORE support to provide a validation set for SureQuant targeted assays.

15.5.6 Level 1-3 Data Analysis

Our overall capabilities and the types of software utilized for proteomic and proteogenomic analysis in CPTAC-defined Levels 1-3 are described elsewhere and are available upon request to Dr. Bing Zhang at Baylor College of Medicine.

15.5.7 ALTERNATE Sample Availability

There is a very large number of samples available from the ALTERNATE trial to study, presenting a sample processing challenge. Fortunately, sample processing has proceeded for the last 6 years, as "omics" analyses are embedded into the protocol. Sample preparations are still ongoing, but baseline protein extractions will be complete by the end of August 2021. One month on-treatment samples for mRNA, DNA and protein will be completed by mid-2022 in time for the analysis to begin at the onset of CPTAC grant funding.

15.5.8 Power for Discovery and Validation of endocrine therapy markers in LumA samples

There are 417 LumA samples identified through application of the PAM50 model to RNAseq data already completed in ALTERNATE. For the TMT-based discovery set we will assess the relationship between baseline pRB levels and endocrine therapy response status in LumA tumors using the receiver-operating characteristic (ROC) analysis. Endocrine therapy response will be determined by mPEPI=0/pCR status and changes in Ki67 baseline to one month as secondary analysis. The overall mPEPI=0 (resistant) rate in the LumA population is 26.5% (**Table 1**). A sample size of 100 will provide 88% power to detect the difference between an AUC of 0.7 and the null hypothesis value (0.5) at the significance level of 0.05[163]. If baseline pRB is confirmed to be predictive of response based on the TMT-based analysis, the discovery set will be used to train pRB SureQuant assays to reproduce the ROC curve, which can be then taken into the confirmatory set for validation. If the primary hypothesis is not supported, we will build alternative models in the discovery data set based on other CDK4 targets, if necessary, as a composite score to predict endocrine therapy responsiveness in LumA tumors and then pursue a similar SureQuant-based approach in the confirmatory set for validation.

15.5.9 Power considerations for discovery and validation of chemotherapy response markers

The Abeyance pathology samples are subjected to RNA-seq analysis on an ongoing basis. Thus, by the time the protocol is initiated, we can select 120 cases, about 50% from ALTERNATE and 50% from Abeyance pathology. Specifically, we will select 60 LumB cases and 60 non-Luminal cases for TMT-based proteomics, enriching the LumB population with pCR cases so that there are least 15 LumB pCRs and 30 non-Luminal pCRs. We hypothesize that baseline pRB levels will be predictive for pCR. These cohorts should provide more than 85% power to detect AUC's of 0.75 and 0.72, compared to a null value of 0.5, for LumB and non-Luminal, respectively[163]. We will have similar power to examine the interactions between pCR and the stimulatory immune modulatory score and LIG1 status at the copy number, mRNA and protein level. Each of these analyses can also be combined to produce a composite pCR predictor. If this TMT-based analysis produces a potentially useful predictive model (AUC>0.7), the discovery set samples will be used to train SureQuant assays to reproduce the ROC curve, which can be then taken to an independent set of samples accrued from the Abeyance Pathology protocol.

15.5.10 Multi-protein signatures for predicting endocrine therapy and chemotherapy response in ER+ breast cancer

In addition to the hypothesis-driven analyses required to power our investigations, we will also perform discovery analyses using the TMT data to identify predictive protein signatures for endocrine and chemotherapy response. In this case, we will apply the proMS algorithm[170] recently published by the Zhang lab to identify proteins that have a high association with response but low association with each other. Specifically, a weighted k-medoids clustering

algorithm will be applied to all univariately informative proteins to identify both co-expressed protein clusters and a representative protein (i.e., medoid) for each cluster. We will develop targeted SureQuant assays for these representative proteins, particularly if our hypothesis-driven analyses fail. Data from targeted assays will then be used to train binary classifiers to distinguish sensitive and resistant tumors. Classifiers will be developed using multiple machine learning algorithms including logistic regression, k-nearest neighbors (KNN), support vector machines (SVM), and random forests. Performance of the classifiers will be evaluated through Monte-Carlo cross-validation and quantified on the basis of ROC analysis. These additional SureQuant assays will also be examined in an independent data set, in parallel to the hypothesis-driven approach.

16.0 REFERENCES

1. Howlader, N., et al., *SEER Cancer Statistics Review, 1975-2008, National Cancer Institute. Bethesda, MD, http://seer.cancer.gov/csr/1975_2008/, based on November 2010 SEER data submission, posted to the SEER web site, 2011.* 2011.
2. DeSantis, C., et al., *Breast cancer statistics, 2011.* CA: A Cancer Journal for Clinicians, 2011. **61**(6): p. 409-18.
3. Anderson, W.F., et al., *Estrogen receptor breast cancer phenotypes in the Surveillance, Epidemiology, and End Results database.* Breast Cancer Res Treat, 2002. **76**(1): p. 27-36.
4. Demicheli, R., et al., *Recurrence and mortality according to estrogen receptor status for breast cancer patients undergoing conservative surgery. Ipsilateral breast tumour recurrence dynamics provides clues for tumour biology within the residual breast.* BMC Cancer, 2010. **10**: p. 656.
5. Demicheli, R., et al., *Recurrence and mortality dynamics for breast cancer patients undergoing mastectomy according to estrogen receptor status: different mortality but similar recurrence.* Cancer Sci, 2010. **101**(3): p. 826-30.
6. Early Breast Cancer Trialists' Collaborative, G., *Relevance of breast cancer hormone receptors and other factors to the efficacy of adjuvant tamoxifen: patient-level meta-analysis of randomised trials.* The Lancet, 2011. **378**(9793): p. 771-784.
7. Cuzick, J., et al., *Effect of anastrozole and tamoxifen as adjuvant treatment for early-stage breast cancer: 10-year analysis of the ATAC trial.* The Lancet Oncology, 2010. **11**(12): p. 1135-1141.
8. Ellis, M.J., et al., *Outcome prediction for estrogen receptor-positive breast cancer based on postneoadjuvant endocrine therapy tumor characteristics.* J Natl Cancer Inst, 2008. **100**(19): p. 1380-8.
9. Chia, Y.H., M.J. Ellis, and C.X. Ma, *Neoadjuvant endocrine therapy in primary breast cancer: indications and use as a research tool.* Br J Cancer, 2010. **103**(6): p. 759-64.
10. Ellis, M.J., *Neoadjuvant endocrine therapy as a drug development strategy.* Clin Cancer Res, 2004. **10**(1 Pt 2): p. 391S-5S.
11. Eiermann, W., et al., *Preoperative treatment of postmenopausal breast cancer patients with letrozole: A randomized double-blind multicenter study.* Ann Oncol, 2001. **12**(11): p. 1527-32.
12. Smith, I.E., et al., *Neoadjuvant treatment of postmenopausal breast cancer with anastrozole, tamoxifen, or both in combination: the Immediate Preoperative Anastrozole, Tamoxifen, or Combined with Tamoxifen (IMPACT) multicenter double-blind randomized trial.* J Clin Oncol, 2005. **23**(22): p. 5108-16.
13. Cataliotti, L., et al., *Comparison of anastrozole versus tamoxifen as preoperative therapy in postmenopausal women with hormone receptor-positive breast cancer: the Pre-Operative "Arimidex" Compared to Tamoxifen (PROACT) trial.* Cancer, 2006. **106**(10): p. 2095-103.
14. Ellis, M.J., et al., *Randomized phase II neoadjuvant comparison between letrozole, anastrozole, and exemestane for postmenopausal women with estrogen receptor-rich stage 2 to 3 breast cancer: clinical and biomarker outcomes and predictive value of the baseline PAM50-based intrinsic subtype--ACOSOG Z1031.* Journal of clinical oncology : official journal of the American Society of Clinical Oncology, 2011. **29**(17): p. 2342-9.

15. Seo JH, e.a., *Meta-analysis of pre-operative aromatase inhibitor versus tamoxifen in postmenopausal women with hormone recepgtor-postive breast cancer.* Cancer Chemother Pharmacol, 2009. **63:261-6, 2009**.
16. Ellis, M.J., et al., *Letrozole inhibits tumor proliferation more effectively than tamoxifen independent of HER1/2 expression status.* Cancer Res, 2003. **63**(19): p. 6523-31.
17. Thurlimann, B., et al., *A comparison of letrozole and tamoxifen in postmenopausal women with early breast cancer.* N Engl J Med, 2005. **353**(26): p. 2747-57.
18. Baum, M., et al., *Anastrozole alone or in combination with tamoxifen versus tamoxifen alone for adjuvant treatment of postmenopausal women with early breast cancer: first results of the ATAC randomised trial.* Lancet, 2002. **359**(9324): p. 2131-9.
19. Goss, P.E., et al., *Final Analysis of NCIC CTG MA.27: A Randomized Phase III Trial of Exemestane Versus Anastrozole in Postmenopausal Women with Hormone Receptor Positive Primary Breast Cancer.*, in *San Antonio Breast Cancer Symposium*. 2010: San Antonio, Texas. p. abstract S1-1.
20. Goncalves, R., et al., *Use of neoadjuvant data to design adjuvant endocrine therapy trials for breast cancer.* Nature reviews. Clinical oncology, 2012. **9**(4): p. 223-9.
21. Smith, I.E., et al., *A phase II placebo-controlled trial of neoadjuvant anastrozole alone or with gefitinib in early breast cancer.* J Clin Oncol, 2007. **25**(25): p. 3816-22.
22. Robertson, J.F., et al., *Comparison of the short-term biological effects of 7alpha-[9-(4,4,5,5,5-pentafluoropentylsulfinyl)-nonyl]estra-1,3,5, (10)-triene-3,17beta-diol (Faslodex) versus tamoxifen in postmenopausal women with primary breast cancer.* Cancer Res, 2001. **61**(18): p. 6739-46.
23. Dowsett, M., et al., *Effect of raloxifene on breast cancer cell Ki67 and apoptosis: a double-blind, placebo-controlled, randomized clinical trial in postmenopausal patients.* Cancer Epidemiol Biomarkers Prev, 2001. **10**(9): p. 961-6.
24. Guix, M., et al., *Short preoperative treatment with erlotinib inhibits tumor cell proliferation in hormone receptor-positive breast cancers.* J Clin Oncol, 2008. **26**(6): p. 897-906.
25. Olson, J.A., Jr., et al., *Improved surgical outcomes for breast cancer patients receiving neoadjuvant aromatase inhibitor therapy: results from a multicenter phase II trial.* J Am Coll Surg, 2009. **208**(5): p. 906-14; discussion 915-6.
26. Dowsett, M., et al., *Short-term changes in Ki-67 during neoadjuvant treatment of primary breast cancer with anastrozole or tamoxifen alone or combined correlate with recurrence-free survival.* Clin Cancer Res, 2005. **11**(2 Pt 2): p. 951s-8s.
27. Dowsett, M., et al., *Prognostic value of Ki67 expression after short-term presurgical endocrine therapy for primary breast cancer.* J Natl Cancer Inst, 2007. **99**(2): p. 167-70.
28. Nielsen, T.O., et al., *A comparison of PAM50 intrinsic subtyping with immunohistochemistry and clinical prognostic factors in tamoxifen-treated estrogen receptor-positive breast cancer.* Clin Cancer Res. **16**(21): p. 5222-32.
29. Wakeling, A.E., M. Dukes, and J. Bowler, *A potent specific pure antiestrogen with clinical potential.* Cancer Res, 1991. **51**(15): p. 3867-73.
30. Howell, A., *Pure oestrogen antagonists for the treatment of advanced breast cancer.* Endocr Relat Cancer, 2006. **13**(3): p. 689-706.
31. Howell, A., et al., *Fulvestrant, Formerly ICI 182,780, Is as Effective as Anastrozole in Postmenopausal Women With Advanced Breast Cancer Progressing After Prior Endocrine Treatment.* Journal of Clinical Oncology, 2002. **20**(16): p. 3396-3403.

32. Osborne, C.K., et al., *Double-blind, randomized trial comparing the efficacy and tolerability of fulvestrant versus anastrozole in postmenopausal women with advanced breast cancer progressing on prior endocrine therapy: results of a North American trial.* J Clin Oncol, 2002. **20**(16): p. 3386-95.
33. Robertson, J.F., et al., *Fulvestrant versus anastrozole for the treatment of advanced breast carcinoma in postmenopausal women: a prospective combined analysis of two multicenter trials.* Cancer, 2003. **98**(2): p. 229-38.
34. Chia, S., et al., *Double-Blind, Randomized Placebo Controlled Trial of Fulvestrant Compared With Exemestane After Prior Nonsteroidal Aromatase Inhibitor Therapy in Postmenopausal Women With Hormone Receptor-Positive, Advanced Breast Cancer: Results From EFECT.* J Clin Oncol, 2008. **26**(10): p. 1664-1670.
35. Howell, A. and J. Bergh, *Insights Into the Place of Fulvestrant for the Treatment of Advanced Endocrine Responsive Breast Cancer.* Journal of Clinical Oncology. **28**(30): p. 4548-4550.
36. Kuter, I., et al., *Fulvestrant 500 mg vs 250 mg: First results from NEWEST, a randomized, phase II neoadjuvant trial in postmenopausal women with locally advanced, estrogen receptor-positive breast cancer.* Breast Cancer Research and Treatment, 2007. **106**: p. abstract 589.
37. Di Leo, A., et al., *Results of the CONFIRM phase III trial comparing fulvestrant 250 mg with fulvestrant 500 mg in postmenopausal women with estrogen receptor-positive advanced breast cancer.* Journal of clinical oncology : official journal of the American Society of Clinical Oncology, 2010. **28**(30): p. 4594-600.
38. Robertson, J.F., et al., *Activity of fulvestrant 500 mg versus anastrozole 1 mg as first-line treatment for advanced breast cancer: results from the FIRST study.* J Clin Oncol, 2009. **27**(27): p. 4530-5.
39. Brodie, A., et al., *Therapeutic observations in MCF-7 aromatase xenografts.* Clin Cancer Res, 2005. **11**(2 Pt 2): p. 884s-8s.
40. Jelovac, D., et al., *Additive antitumor effect of aromatase inhibitor letrozole and antiestrogen fulvestrant in a postmenopausal breast cancer model.* Cancer Res, 2005. **65**(12): p. 5439-44.
41. Macedo, L.F., et al., *Combination of anastrozole with fulvestrant in the intratumoral aromatase xenograft model.* Cancer Res, 2008. **68**(9): p. 3516-22.
42. Osborne, C.K., et al., *Comparison of the effects of a pure steroidal antiestrogen with those of tamoxifen in a model of human breast cancer.* J Natl Cancer Inst, 1995. **87**(10): p. 746-50.
43. Bergh, J., et al., *FACT: An Open-Label Randomized Phase III Study of Fulvestrant and Anastrozole in Combination Compared With Anastrozole Alone as First-Line Therapy for Patients With Receptor-Positive Postmenopausal Breast Cancer.* Journal of Clinical Oncology, 2012.
44. Mehta, R.S., et al., *Combination anastrozole and fulvestrant in metastatic breast cancer.* The New England journal of medicine, 2012. **367**(5): p. 435-44.
45. Rastogi, P., et al., *Preoperative chemotherapy: updates of National Surgical Adjuvant Breast and Bowel Project Protocols B-18 and B-27.* Journal of clinical oncology : official journal of the American Society of Clinical Oncology, 2008. **26**(5): p. 778-85.
46. van der Hage, J.A., et al., *Preoperative chemotherapy in primary operable breast cancer: results from the European Organization for Research and Treatment of Cancer trial 10902.* Journal of clinical oncology : official journal of the American Society of Clinical Oncology, 2001. **19**(22): p. 4224-37.

47. Symmans, W.F., et al., *Measurement of residual breast cancer burden to predict survival after neoadjuvant chemotherapy.* J Clin Oncol, 2007. **25**(28): p. 4414-22.
48. Carey, L.A., et al., *The Triple Negative Paradox: Primary Tumor Chemosensitivity of Breast Cancer Subtypes.* Clinical Cancer Research, 2007. **13**(8): p. 2329-2334.
49. Rouzier, R., et al., *Breast Cancer Molecular Subtypes Respond Differently to Preoperative Chemotherapy.* Clinical Cancer Research, 2005. **11**(16): p. 5678-5685.
50. Berry, D.A., et al., *Estrogen-receptor status and outcomes of modern chemotherapy for patients with node-positive breast cancer.* JAMA, 2006. **295**(14): p. 1658-67.
51. Lips, E.H., et al., *Neoadjuvant chemotherapy in ER+ HER2- breast cancer: response prediction based on immunohistochemical and molecular characteristics.* Breast cancer research and treatment, 2012. **131**(3): p. 827-36.
52. Howell, A., et al., *ICI 182,780 (Faslodex): development of a novel, "pure" antiestrogen.* Cancer, 2000. **89**(4): p. 817-25.
53. AstraZeneca., *Faslodex: fulvestrant injection. Prescribing information [online]. Available from URL:* http://www1.astrazeneca-us.com/pi/faslodex.pdf *[Accessed 2011 Jan 5]* 2011.
54. Croxtall, J.D. and K. McKeage, *Fulvestrant: a review of its use in the management of hormone receptor-positive metastatic breast cancer in postmenopausal women.* Drugs, 2011. **71**(3): p. 363-80.
55. Addo, S., R.A. Yates, and A. Laight, *A phase I trial to assess the pharmacology of the new oestrogen receptor antagonist fulvestrant on the endometrium in healthy postmenopausal volunteers.* Br J Cancer. **87**(12): p. 1354-1359.
56. Agrawal, A., et al., *Bone turnover markers in postmenopausal breast cancer treated with fulvestrant - A pilot study.* The Breast, 2009. **18**(3): p. 204-207.
57. Howell, A., et al., *Fulvestrant versus anastrozole for the treatment of advanced breast carcinoma: a prospectively planned combined survival analysis of two multicenter trials.* Cancer, 2005. **104**(2): p. 236-9.
58. deGraffenried, L.A., et al., *Inhibition of mTOR activity restores tamoxifen response in breast cancer cells with aberrant Akt Activity.* Clin Cancer Res, 2004. **10**(23): p. 8059-67.
59. Schiff, P.B., J. Fant, and S.B. Horwitz, *Promotion of microtubule assembly in vitro by taxol.* Nature, 1979. **277**(5698): p. 665-7.
60. Eisenhauer, E.A. and J.B. Vermorken, *The taxoids. Comparative clinical pharmacology and therapeutic potential.* Drugs, 1998. **55**(1): p. 5-30.
61. Liebmann, J.E., et al., *Cytotoxic studies of paclitaxel (Taxol) in human tumour cell lines.* Br J Cancer, 1993. **68**(6): p. 1104-9.
62. Bhalla, K., et al., *Taxol induces internucleosomal DNA fragmentation associated with programmed cell death in human myeloid leukemia cells.* Leukemia, 1993. **7**(4): p. 563-8.
63. Gangemi, R.M., et al., *Taxol cytotoxicity on human leukemia cell lines is a function of their susceptibility to programmed cell death.* Cancer Chemother Pharmacol, 1995. **36**(5): p. 385-92.
64. Milas, L., et al., *Kinetics of mitotic arrest and apoptosis in murine mammary and ovarian tumors treated with taxol.* Cancer Chemother Pharmacol, 1995. **35**(4): p. 297-303.
65. Milross, C.G., et al., *Relationship of mitotic arrest and apoptosis to antitumor effect of paclitaxel.* J Natl Cancer Inst, 1996. **88**(18): p. 1308-14.
66. Mamounas, E.P., et al., *Paclitaxel after doxorubicin plus cyclophosphamide as adjuvant chemotherapy for node-positive breast cancer: results from NSABP B-28.* J Clin Oncol, 2005. **23**(16): p. 3686-96.

67. Henderson, I.C., et al., *Improved outcomes from adding sequential Paclitaxel but not from escalating Doxorubicin dose in an adjuvant chemotherapy regimen for patients with node-positive primary breast cancer.* J Clin Oncol, 2003. **21**(6): p. 976-83.
68. Cox, D., *Regression models and life-tables (with discussion).* Journal of the Royal Statistical Society, 1972. **Series B**: p. 1100-1120.
69. Brookmeyer, R., Crowley, JJ, *A confidence interval for the median survival time.* Biometrics, 1982. **38**: p. 29-41.
70. Walker, S., Duncan DB, *Estimation of the probability of an event as a function of several independent variables.* Biometrika, 1967. **54**: p. 167-179.
71. Kaplan, E., Meier P., *Non-parametric estimation from incomplete observations.* Journal of the American Statistical Association, 1958. **53**: p. 457-481.
72. Andersen, P.K. and R.D. Gill, *Cox's Regression Model for Counting Processes: A Large Sample Study.* The Annals of Statistics, 1982. **10**(4): p. 1100-1120.
73. Dowsett, M., et al., *Assessment of Ki67 in breast cancer: recommendations from the International Ki67 in Breast Cancer working group.* Journal of the National Cancer Institute, 2011. **103**(22): p. 1656-64.
74. Ma, C.X., C.G. Sanchez, and M.J. Ellis, *Predicting endocrine therapy responsiveness in breast cancer.* Oncology (Williston Park), 2009. **23**(2): p. 133-42.
75. Ellis, M.J., et al., *Letrozole is more effective neoadjuvant endocrine therapy than tamoxifen for ErbB-1- and/or ErbB-2-positive, estrogen receptor-positive primary breast cancer: evidence from a phase III randomized trial.* J Clin Oncol, 2001. **19**(18): p. 3808-16.
76. Stearns, V., et al., *A prospective randomized pilot study to evaluate predictors of response in serial core biopsies to single agent neoadjuvant doxorubicin or paclitaxel for patients with locally advanced breast cancer.* Clin Cancer Res, 2003. **9**(1): p. 124-33.
77. Ma, C.X., et al., *Targeting Chk1 in p53-deficient triple-negative breast cancer is therapeutically beneficial in human-in-mouse tumor models.* The Journal of clinical investigation, 2012. **122**(4): p. 1541-52.
78. Ellis, M.J., et al., *Whole-genome analysis informs breast cancer response to aromatase inhibition.* Nature, 2012. **486**(7403): p. 353-60.
79. Cancer Genome Atlas, N., *Comprehensive molecular portraits of human breast tumours.* Nature, 2012. **490**(7418): p. 61-70.
80. Li, S., et al., *Endocrine-therapy-resistant ESR1 variants revealed by genomic characterization of breast-cancer-derived xenografts.* Cell Rep, 2013. **4**(6): p. 1116-30.
81. Ellis, M.J. and C.M. Perou, *The genomic landscape of breast cancer as a therapeutic roadmap.* Cancer Discov, 2013. **3**(1): p. 27-34.
82. Griffith, M., et al., *DGIdb: mining the druggable genome.* Nat Methods, 2013.
83. Kumar, R.D., et al., *Prioritizing Potentially Druggable Mutations with dGene: An Annotation Tool for Cancer Genome Sequencing Data.* PLoS One, 2013. **8**(6): p. e67980.
84. Wang, L., et al., *SF3B1 and other novel cancer genes in chronic lymphocytic leukemia.* N Engl J Med, 2011. **365**(26): p. 2497-506.
85. Furney, S.J., et al., *SF3B1 Mutations Are Associated with Alternative Splicing in Uveal Melanoma.* Cancer Discov, 2013. **3**(10): p. 1122-9.
86. Cizkova, M., et al., *PIK3CA mutation impact on survival in breast cancer patients and in ERalpha, PR and ERBB2-based subgroups.* Breast Cancer Res, 2012. **14**(1): p. R28.

87. Ellis, M.J., et al., *Phosphatidyl-inositol-3-kinase alpha catalytic subunit mutation and response to neoadjuvant endocrine therapy for estrogen receptor positive breast cancer.* Breast cancer research and treatment, 2010. **119**(2): p. 379-90.
88. Alexandrov, L.B., et al., *Signatures of mutational processes in human cancer.* Nature, 2013. **500**(7463): p. 415-21.
89. Maher, C.A., et al., *Chimeric transcript discovery by paired-end transcriptome sequencing.* Proc Natl Acad Sci U S A, 2009. **106**(30): p. 12353-8.
90. Iyer, M.K., A.M. Chinnaiyan, and C.A. Maher, *ChimeraScan: a tool for identifying chimeric transcription in sequencing data.* Bioinformatics, 2011. **27**(20): p. 2903-4.
91. Bose, R., et al., *Activating HER2 mutations in HER2 gene amplification negative breast cancer.* Cancer Discov, 2013. **3**(2): p. 224-37.
92. Dees, N.D., et al., *MuSiC: identifying mutational significance in cancer genomes.* Genome Res, 2012. **22**(8): p. 1589-98.
93. Friedman, J., T. Hastie, and R. Tibshirani, *Regularization Paths for Generalized Linear Models via Coordinate Descent.* J Stat Softw, 2010. **33**(1): p. 1-22.
94. Hofree, M., et al., *Network-based stratification of tumor mutations.* Nat Methods, 2013. **10**(11): p. 1108-15.
95. Szklarczyk, D., et al., *The STRING database in 2011: functional interaction networks of proteins, globally integrated and scored.* Nucleic Acids Res, 2011. **39**(Database issue): p. D561-8.
96. Lee, I., et al., *Prioritizing candidate disease genes by network-based boosting of genome-wide association data.* Genome Res, 2011. **21**(7): p. 1109-21.
97. Cerami, E.G., et al., *Pathway Commons, a web resource for biological pathway data.* Nucleic Acids Res, 2011. **39**(Database issue): p. D685-90.
98. Polley, M.Y., et al., *Two-stage adaptive cutoff design for building and validating a prognostic biomarker signature.* Stat Med, 2014. **33**(29): p. 5097-110.
99. Jones, S.R., S. Carley, and M. Harrison, *An introduction to power and sample size estimation.* Emerg Med J, 2003. **20**(5): p. 453-8.
100. Li, J. and J. Fine, *On sample size for sensitivity and specificity in prospective diagnostic accuracy studies.* Stat Med, 2004. **23**(16): p. 2537-50.
101. Langmead, B., et al., *Ultrafast and memory-efficient alignment of short DNA sequences to the human genome.* Genome Biol, 2009. **10**(3): p. R25.
102. Trapnell, C., L. Pachter, and S.L. Salzberg, *TopHat: discovering splice junctions with RNA-Seq.* Bioinformatics, 2009. **25**(9): p. 1105-11.
103. Trapnell, C., et al., *Transcript assembly and quantification by RNA-Seq reveals unannotated transcripts and isoform switching during cell differentiation.* Nat Biotechnol, 2010. **28**(5): p. 511-5.
104. Anders, S. and W. Huber, *Differential expression analysis for sequence count data.* Genome Biol, 2010. **11**(10): p. R106.
105. Robinson, M.D., D.J. McCarthy, and G.K. Smyth, *edgeR: a Bioconductor package for differential expression analysis of digital gene expression data.* Bioinformatics, 2010. **26**(1): p. 139-40.
106. Smyth, G.K., *Limma: linear models for microarray data*. Bioinformatics and Computational Biology Solutions Using R and Bioconductor. 2005: Springer
107. Tusher, V.G., R. Tibshirani, and G. Chu, *Significance analysis of microarrays applied to the ionizing radiation response.* Proc Natl Acad Sci U S A, 2001. **98**(9): p. 5116-21.

108. Michaud, J., et al., *Integrative analysis of RUNX1 downstream pathways and target genes.* BMC Genomics, 2008. **9**: p. 363.
109. Wu, D., et al., *ROAST: rotation gene set tests for complex microarray experiments.* Bioinformatics, 2010. **26**(17): p. 2176-82.
110. Wu, D. and G.K. Smyth, *Camera: a competitive gene set test accounting for inter-gene correlation.* Nucleic Acids Res, 2012. **40**(17): p. e133.
111. Parker, H.S., H. Corrada Bravo, and J.T. Leek, *Removing batch effects for prediction problems with frozen surrogate variable analysis.* PeerJ, 2014. **2**: p. e561.
112. Leek, J.T., et al., *sva: Surrogate Variable Analysis. R package version 3.14.0.*
113. Leek, J.T., et al., *The sva package for removing batch effects and other unwanted variation in high-throughput experiments.* Bioinformatics, 2012. **28**(6): p. 882-3.
114. Leek, J.T., et al., *Tackling the widespread and critical impact of batch effects in high-throughput data.* Nat Rev Genet, 2010. **11**(10): p. 733-9.
115. Hansen, K.D., R.A. Irizarry, and Z. Wu, *Removing technical variability in RNA-seq data using conditional quantile normalization.* Biostatistics, 2012. **13**(2): p. 204-16.
116. Robinson, D.R., et al., *Activating ESR1 mutations in hormone-resistant metastatic breast cancer.* Nat Genet, 2013. **45**(12): p. 1446-51.
117. Toy, W., et al., *ESR1 ligand-binding domain mutations in hormone-resistant breast cancer.* Nat Genet, 2013. **45**(12): p. 1439-45.
118. Miller, C.A., et al., *SciClone: inferring clonal architecture and tracking the spatial and temporal patterns of tumor evolution.* PLoS Comput Biol, 2014. **10**(8): p. e1003665.
119. Buono, G., et al., *Circulating tumor DNA analysis in breast cancer: Is it ready for prime-time?* Cancer Treatment Reviews, 2019. **73**: p. 73-83.
120. Wan, J.C., et al., *Liquid biopsies come of age: towards implementation of circulating tumour DNA.* Nature Reviews Cancer, 2017. **17**(4): p. 223.
121. Heitzer, E., et al., *Current and future perspectives of liquid biopsies in genomics-driven oncology.* Nature Reviews Genetics, 2019. **20**(2): p. 71-88.
122. Ignatiadis, M., G.W. Sledge, and S.S. Jeffrey, *Liquid biopsy enters the clinic - implementation issues and future challenges.* Nat Rev Clin Oncol, 2021.
123. McDonald, B.R., et al., *Personalized circulating tumor DNA analysis to detect residual disease after neoadjuvant therapy in breast cancer.* Science Translational Medicine, 2019. **11**(504): p. eaax7392.
124. Coombes, C., et al., *Personalized detection of circulating tumor DNA antedates breast cancer metastatic recurrence.* Clinical Cancer Research, 2019: p. clincanres.3663.2018.
125. Li, J., et al., *Patient specific circulating tumor DNA fingerprints to monitor treatment response across multiple tumors.* J Transl Med, 2020. **18**(1): p. 293.
126. Wan, J.C.M., et al., *ctDNA monitoring using patient-specific sequencing and integration of variant reads.* Sci Transl Med, 2020. **12**(548).
127. Garcia-Murillas, I., et al., *Mutation tracking in circulating tumor DNA predicts relapse in early breast cancer.* Science Translational Medicine, 2015. **7**(302): p. 302ra133-302ra133.
128. Olsson, E., et al., *Serial monitoring of circulating tumor DNA in patients with primary breast cancer for detection of occult metastatic disease.* EMBO Mol Med, 2015. **7**(8): p. 1034-47.
129. Riva, F., et al., *Patient-Specific Circulating Tumor DNA Detection during Neoadjuvant Chemotherapy in Triple-Negative Breast Cancer.* Clin Chem, 2017. **63**(3): p. 691-699.
130. Garcia-Murillas, I., et al., *Assessment of Molecular Relapse Detection in Early-Stage Breast Cancer.* JAMA Oncol, 2019. **5**(10): p. 1473-1478.

131. Cavallone, L., et al., *Prognostic and predictive value of circulating tumor DNA during neoadjuvant chemotherapy for triple negative breast cancer.* Sci Rep, 2020. **10**(1): p. 14704.
132. Magbanua, M.J.M., et al., *Circulating tumor DNA in neoadjuvant-treated breast cancer reflects response and survival.* Ann Oncol, 2021. **32**(2): p. 229-239.
133. Abbosh, C., et al., *Phylogenetic ctDNA analysis depicts early-stage lung cancer evolution.* Nature, 2017. **545**(7655): p. 446-451.
134. Christensen, E., et al., *Early Detection of Metastatic Relapse and Monitoring of Therapeutic Efficacy by Ultra-Deep Sequencing of Plasma Cell-Free DNA in Patients With Urothelial Bladder Carcinoma.* Journal of Clinical Oncology, 2019. **37**(18): p. 1547-1557.
135. Reinert, T., et al., *Analysis of Plasma Cell-Free DNA by Ultradeep Sequencing in Patients With Stages I to III Colorectal Cancer.* JAMA Oncology, 2019. **5**(8): p. 1124-1131.
136. Pan, H., et al., *20-Year Risks of Breast-Cancer Recurrence after Stopping Endocrine Therapy at 5 Years.* N Engl J Med, 2017. **377**(19): p. 1836-1846.
137. Goss, P.E., et al., *Extending Aromatase-Inhibitor Adjuvant Therapy to 10 Years.* N Engl J Med, 2016. **375**(3): p. 209-19.
138. Tjan-Heijnen, V.C.G., et al., *Extended adjuvant aromatase inhibition after sequential endocrine therapy (DATA): a randomised, phase 3 trial.* Lancet Oncol, 2017. **18**(11): p. 1502-1511.
139. Blok, E.J., et al., *Optimal Duration of Extended Adjuvant Endocrine Therapy for Early Breast Cancer; Results of the IDEAL Trial (BOOG 2006-05).* JNCI: Journal of the National Cancer Institute, 2017. **110**(1): p. 40-48.
140. De Mattos-Arruda, L. and C. Caldas, *Cell-free circulating tumour DNA as a liquid biopsy in breast cancer.* Molecular oncology, 2016. **10**(3): p. 464-474.
141. Ma, C.X., et al., *ALTERNATE: Neoadjuvant endocrine treatment (NET) approaches for clinical stage II or III estrogen receptor-positive HER2-negative breast cancer (ER+ HER2- BC) in postmenopausal (PM) women: Alliance A011106.* J Clin Oncol 38 (suppl; abstr 504), 2020.
142. Ma, C.X., et al. *GS4-05. Neoadjuvant chemotherapy (NCT) response in postmenopausal women with clinical stage II or III estrogen receptor positive (ER+) and HER2 negative (HER2-) breast cancer (BC) resistant to endocrine therapy (ET) in the ALTERNATE trial (Alliance A011106)* in *San Antonio Breast Cancer Symposium*. 2020. Virtual Meeting.
143. Suman, V.J., M.J. Ellis, and C.X. Ma, *The ALTERNATE trial: assessing a biomarker driven strategy for the treatment of post-menopausal women with ER+/Her2- invasive breast cancer.* Chin Clin Oncol, 2015. **4**(3): p. 34.
144. Razavi, P., et al., *The Genomic Landscape of Endocrine-Resistant Advanced Breast Cancers.* Cancer Cell, 2018. **34**(3): p. 427-438 e6.
145. Griffith, O.L., et al., *The prognostic effects of somatic mutations in ER-positive breast cancer.* Nat Commun, 2018. **9**(1): p. 3476.
146. Krug, K., et al., *Proteogenomic Landscape of Breast Cancer Tumorigenesis and Targeted Therapy.* Cell, 2020. **183**(5): p. 1436-1456 e31.
147. Zheng, Z.Y., et al., *Neurofibromin Is an Estrogen Receptor-alpha Transcriptional Co-repressor in Breast Cancer.* Cancer Cell, 2020. **37**(3): p. 387-402 e7.
148. Rueda, O.M., et al., *Dynamics of breast-cancer relapse reveal late-recurring ER-positive genomic subgroups.* Nature, 2019. **567**(7748): p. 399-404.
149. Curtis, C., et al., *The genomic and transcriptomic architecture of 2,000 breast tumours reveals novel subgroups.* Nature, 2012. **486**(7403): p. 346-52.

150. Rokita, J.L., et al., *Genomic Profiling of Childhood Tumor Patient-Derived Xenograft Models to Enable Rational Clinical Trial Design.* Cell Rep, 2019. **29**(6): p. 1675-1689 e9.
151. Bainbridge, M.N., et al., *Targeted enrichment beyond the consensus coding DNA sequence exome reveals exons with higher variant densities.* Genome Biol, 2011. **12**(7): p. R68.
152. Satpathy, S., et al., *Microscaled proteogenomic methods for precision oncology.* Nat Commun, 2020. **11**(1): p. 532.
153. Mermel, C.H., et al., *GISTIC2.0 facilitates sensitive and confident localization of the targets of focal somatic copy-number alteration in human cancers.* Genome Biol, 2011. **12**(4): p. R41.
154. Ellis, M.J., et al., *Ki67 Proliferation Index as a Tool for Chemotherapy Decisions During and After Neoadjuvant Aromatase Inhibitor Treatment of Breast Cancer: Results From the American College of Surgeons Oncology Group Z1031 Trial (Alliance).* Journal of Clinical Oncology, 2017. **35**(10): p. 1061-1069.
155. Hamilton, E. and J.R. Infante, *Targeting CDK4/6 in patients with cancer.* Cancer Treat Rev, 2016. **45**: p. 129-38.
156. Goel, S., et al., *CDK4/6 Inhibition in Cancer: Beyond Cell Cycle Arrest.* Trends Cell Biol, 2018. **28**(11): p. 911-925.
157. Derenzini, M., et al., *Loss of retinoblastoma tumor suppressor protein makes human breast cancer cells more sensitive to antimetabolite exposure.* Clin Cancer Res, 2008. **14**(7): p. 2199-209.
158. Bosco, E.E. and E.S. Knudsen, *RB in breast cancer: at the crossroads of tumorigenesis and treatment.* Cell Cycle, 2007. **6**(6): p. 667-71.
159. Terranova-Barberio, M., et al., *Exhausted T cell signature predicts immunotherapy response in ER-positive breast cancer.* Nat Commun, 2020. **11**(1): p. 3584.
160. Vafaizadeh, V. and Z. Barekati, *Immuno-Oncology Biomarkers for Personalized Immunotherapy in Breast Cancer.* Front Cell Dev Biol, 2020. **8**: p. 162.
161. Parker, J.S., et al., *Supervised risk predictor of breast cancer based on intrinsic subtypes.* J Clin Oncol, 2009. **27**(8): p. 1160-7.
162. Johnston, S.R.D., et al., *Abemaciclib Combined With Endocrine Therapy for the Adjuvant Treatment of HR+, HER2-, Node-Positive, High-Risk, Early Breast Cancer (monarchE).* J Clin Oncol, 2020. **38**(34): p. 3987-3998.
163. Robin, X., et al., *pROC: an open-source package for R and S+ to analyze and compare ROC curves.* BMC Bioinformatics, 2011. **12**: p. 77.
164. Liao, Y., et al., *WebGestalt 2019: gene set analysis toolkit with revamped UIs and APIs.* Nucleic Acids Res, 2019. **47**(W1): p. W199-W205.
165. Krug, K., et al., *A Curated Resource for Phosphosite-specific Signature Analysis.* Mol Cell Proteomics, 2019. **18**(3): p. 576-593.
166. Anurag, M., et al., *Comprehensive Profiling of DNA Repair Defects in Breast Cancer Identifies a Novel Class of Endocrine Therapy Resistance Drivers.* Clin Cancer Res, 2018. **24**(19): p. 4887-4899.
167. Telli, M.L., et al., *Homologous recombination deficiency (HRD) status predicts response to standard neoadjuvant chemotherapy in patients with triple-negative or BRCA1/2 mutation-associated breast cancer.* Breast Cancer Res Treat, 2018. **168**(3): p. 625-630.
168. Wensink, E., et al., *A review of the sensitivity of metastatic colorectal cancer patients with deficient mismatch repair to standard-of-care chemotherapy and monoclonal antibodies, with recommendations for future research.* Cancer Treat Rev, 2021. **95**: p. 102174.

169. Anurag, M., et al., *Immune Checkpoint Profiles in Luminal B Breast Cancer (Alliance).* J Natl Cancer Inst, 2020. **112**(7): p. 737-746.
170. Shi, Z., et al., *Feature Selection Methods for Protein Biomarker Discovery from Proteomics or Multiomics Data.* Mol Cell Proteomics, 2021. **20**: p. 100083.
171. Lander, E.S., et al., *Initial sequencing and analysis of the human genome.* Nature, 2001. **409**(6822): p. 860-921.
172. Wheeler, D.A., et al., *The complete genome of an individual by massively parallel DNA sequencing.* Nature, 2008. **452**(7189): p. 872-6.
173. Bainbridge, M.N., et al., *Whole exome capture in solution with 3 Gbp of data.* Genome Biol, 2010. **11**(6): p. R62.
174. Albert, T.J., et al., *Direct selection of human genomic loci by microarray hybridization.* Nat Methods, 2007. **4**(11): p. 903-5.
175. Yang, Y., et al., *Clinical whole-exome sequencing for the diagnosis of mendelian disorders.* N Engl J Med, 2013. **369**(16): p. 1502-11.
176. Splinter, K., et al., *Effect of Genetic Diagnosis on Patients with Previously Undiagnosed Disease.* N Engl J Med, 2018. **379**(22): p. 2131-2139.
177. Meng, L.Y., et al., *Use of Exome Sequencing for Infants in Intensive Care Units Ascertainment of Severe Single-Gene Disorders and Effect on Medical Management.* Jama Pediatrics, 2017. **171**(12).
178. Parsons, D.W., et al., *Diagnostic Yield of Clinical Tumor and Germline Whole-Exome Sequencing for Children With Solid Tumors.* JAMA Oncol, 2016. **2**(5): p. 616-624.
179. Yang, Y., et al., *Molecular findings among patients referred for clinical whole-exome sequencing.* JAMA, 2014. **312**(18): p. 1870-9.
180. Liang-Chu, M.M., et al., *Human biosample authentication using the high-throughput, cost-effective SNPtrace(TM) system.* PLoS One, 2015. **10**(2): p. e0116218.

17.0 APPENDICES

17.1 Staging reference

(Selected elements from the AJCC Cancer Staging Manual, 7th edition, 2009)

Primary Tumor (T)	
**TX**	Primary tumor cannot be assessed
**T0**	No evidence of primary tumor
**Tis**	Carcinoma in situ
**Tis (DCIS)**	Ductal carcinoma in situ
**Tis (LCIS)**	Lobular carcinoma in situ
**Tis (Paget's)**	Paget's disease of the nipple NOT associated with invasive carcinoma and/or carcinoma in situ (DCIS and/or LCIS) in the underlying breast parenchyma. Carcinomas in the breast parenchyma associated with Paget's disease are categorized based on the size and characteristics of the parenchymal disease, although the presence of Paget's disease should still be noted
**T1**	Tumor ≤ 20 mm in greatest dimension
**T1mi**	Tumor ≤ 1 mm in greatest dimension
**T1a**	Tumor > 1 mm but ≤ 5 mm in greatest dimension
**T1b**	Tumor > 5 mm but ≤ 10 mm in greatest dimension
**T1c**	Tumor > 10 mm but ≤ 20 mm in greatest dimension
**T2**	Tumor > 20 mm but ≤ 50 mm in greatest dimension
**T3**	Tumor > 50 mm in greatest dimension
**T4**	Tumor of any size with direct extension to the chest wall and/or to the skin (ulceration or skin nodules) Note: Invasion of the dermis alone does not qualify as T4
**T4a**	Extension to the chest wall, not including only pectoralis muscle adherence/invasion
**T4b**	Ulceration and/or ipsilateral satellite nodules and/or edema (including peau d'orange) of the skin, which do not meet the criteria for inflammatory carcinoma
**T4c**	Both T4a and T4b
**T4d**	Inflammatory carcinoma (see "Rules for Classification")
**Regional Lymph Nodes (N)**	
**NX**	Regional lymph nodes cannot be assessed (for example, previously removed)
**N0**	No regional lymph node metastases
**N1**	Metastases to movable ipsilateral level I, II axillary lymph node(s)
**N2**	Metastases in ipsilateral level I, II axillary lymph nodes that are clinically fixed or matted; or in clinically detected* ipsilateral internal mammary nodes in the absence of clinically evident axillary lymph node metastases
**N2a**	Metastases in ipsilateral level I, II axillary lymph nodes fixed to one another (matted) or to other structures
**N2b**	Metastases only in clinically detected* ipsilateral internal mammary nodes and in the absence of clinically evident level I, II axillary lymph node metastases
**N3**	Metastases in ipsilateral infraclavicular (level III axillary) lymph node(s) with or without level I, II axillary lymph node involvement; or in clinically detected* ipsilateral internal mammary lymph node(s) with clinically evident level I, II axillary lymph node metastases; or metastases in ipsilateral supraclavicular lymph node(s) with or without axillary or internal mammary lymph node involvement
**N3a**	Metastases in ipsilateral infraclavicular lymph node(s)
**N3b**	Metastases in ipsilateral internal mammary lymph node(s) and axillary lymph node(s) N3c Metastases in ipsilateral supraclavicular lymph node(s)
**Distant Metastases (M)**	
**M0**	No clinical or radiographic evidence of distant metastases
**cM0(i+)**	No clinical or radiographic evidence of distant metastases, but deposits of molecularly or microscopically detected tumor cells in circulating blood, bone marrow, or other nonregional nodal tissue that are no larger than 0.2 mm in a patient without symptoms or signs of metastases
**M1**	Distant detectable metastases as determined by classic clinical and radiographic means and/or histologically proven larger than 0.2 mm

17.2 ALLRED score

**Allred Score for ER status (0-8)***			
**% Staining Score**	**Proportion of positive staining cells**	**Intensity Score**	**Average intensity of positively stained cells**
0	none	0	none
1	< 1/100	1	weak
2	1/100 to 1/10	2	intermediate
3	1/10 to 1/3	3	strong
4	1/3 to 2/3		
5	>2/3		
*Allred Score = % Staining Score + Intensity Score<br>[Allred 1998]			

17.3 ECOG/Zubrod performance status scale

0 - Asymptomatic and fully active.

1 - Symptomatic; fully ambulatory; restricted in physical strenuous activity.

2 - Symptomatic; ambulatory; capable of self-care; more than 50% of waking hours are spent out of bed.

3 - Symptomatic; limited self-care; spends more than 50% of time in bed, but not bedridden.

4 - Completely disabled; no self-care; 100% bedridden.

17.4 SOP for Whole Exome Sequencing

Background and Experience

The Baylor College of Medicine Human Genome Sequencing Center (HGSC) is an internationally recognized Academic Unit, engaged in a variety of genome projects and the development of high-throughput methods for high-quality DNA sequencing and genome analysis. The HGSC has been operational for more than twenty years, and during this time, it has developed a complete infrastructure to support large-scale sequencing and genomics projects, including a sophisticated informatics core and pipeline, and a state-of-the-art technology development core. Prior accomplishments include a role in the Human Genome Project[171] and in 2006, the HGSC published the first personalized whole genome sequence (WGS) of James Watson[172], paving the way for the field of personal genomics. In 2007, the HGSC developed new "DNA Capture methods" that enabled whole exome sequencing[173, 174] (WES) and transformed both clinical genetic diagnostics and gene discovery in Mendelian disease[175]. The group pioneered the first WES clinical laboratory in 2011 in collaboration with the BCM Department of Human and Molecular Genetics[176-179]. Finally, the HGSC Clinical Laboratory (HGSC-CL) was launched in 2016 and is CAP/CLIA certified (CLIA# 45D2027450) and approved by multiple state authorities, including New York, for the delivery of genetic test data, enabling return of results to individuals via their clinical caregivers. Two such clinical projects are the NIH sponsored Electronic Medical Records and Genomics program (eMERGE) and the Right Drug, Right Dose, Right Time (RIGHT10K) project with the Mayo Clinic, and more recently, the NIH All of Us Research Program which was recently granted an Investigational Device Exemption (IDE) from the FDA to use WGS data to report clinically relevant information back to All of Us participants. Participation in key recent NIH research programs such as TOPMed Phases 2, 3 and 5 and the NHGRI's Centers for Common Disease Genomics as well as other projects sequenced at the HGSC since 2011 are listed in Table 1. Between the HGSC research efforts and the HGSC-CL activities, the group has sequenced hundreds of thousands of genomes and exomes, and targeted gene panels, making the laboratory well positioned to execute the work detailed in this solicitation.

Table 1: BCM HGSC Production on Illumina Instruments by Application since 2011

Project Category	Whole Genome	Whole Exome	Regional Capture	RNAseq
TOPMed	46,348	0	0	0
GMKF	3,289	0	0	0
Cancer	3,900	10,079	2,649	4,030
Population Genetics	538	786	0	5
Microbial or Metagenomic	32,023	0	0	710
Mendelian	449	12,116	523	62
Comparative	1,987	1,700	446	965
Complex Disease	56,727	29,879	19,327	2,251
Clinical Sequencing (CAP/CLIA)	14,146	2,057	28,199	168
**Grand Total**	**159,407**	**56,617**	**51,144**	**8,191**

Whole Exome Sequencing

The WES at the HGSC will follow well-established production procedures with robust tracking, QA and QC. Samples will be procured for sample accession, library construction, sequencing, mapping and variant calling and data dissemination. DNA and blood samples are routinely received for WES processing. The HGSC/HGSC-CL operates a nucleic acid extraction lab where both blood and saliva extractions are validated in the event these sample types are received for clinical processing. Each process is tracked in HGSC-LIMS, with sample tracking and a chain-of-custody achieved by extensive barcoding. The HGSC uses a Fluidigm finger printing assay[180] (96 sites) to confirm gender and to provide a digital ID at intake that can verify concordance with the final sequence to ensure sample identity.

Steps to be conducted for WES are detailed below.

Library Construction: Genomic DNA concentration is first determined via a PicoGreen plate-reader assay. The DNA sample (0.5 ug – 1ug) is fragmented by sonication on the Covaris E-220 instrument in a 96-well format. Next, paired-end pre-capture library preparation is performed with full automation, on the Beckman Biomek FX^p Dual arm robots. This process includes DNA end repair, 3'-adenylation of fragments, ligation to Illumina-specified 'multiplexing PE adapters', adaptor ligation and precapture ligation-mediated PCR (LM-PCR) utilizing Kapa HiFI DNA polymerase. All processes are automated and include multiple SPRI-bead purification steps after each enzyme reaction. For exome sequencing, a set of ~96 dual barcoded adaptors will be used to multiplex samples for capture hybridization and sequencing.

Whole Exome Sequencing (VCRome): The methods for WES have evolved since our first report in 2007, leading to the current NimbleGen liquid capture protocol. VCRome is a commercially available, HGSC-designed, DNA-capture hybridization reagent from Roche/Nimblegen[173]. The reagent targets the coding and near intronic regions of the Vega, CCDS, and RefSeq gene models as well as >1200 miRNA genes, targeting approximately 34 Mbp of genomic DNA including all the coding exons of currently known disease genes (OMIM, HDMG, GeneTest). This reagent provides very high depth of coverage (>140x average coverage, >97% of targeted region at ≥20x coverage) with low sequence input (12 Gbp) and has been used extensively by both the HGSC (>50,000 research exomes) and in clinical service (>10,000 clinical exomes). For this proposal, target enrichment will employ the VCRome capture reagent as well as the 'Panel Killer' probe set of a spike-in probe set (PKv2, 2.5Mb). PKv2 was designed to enhance representation of targets of clinically relevant disease genes that were either missed or below coverage (20x) in the previous WES studies due to factors such as high GC content or low probe density. Methods to enhance clinically relevant target regions have focused on 3,643 genes from GeneTests, and OMIM and ~700 cancer genes. A second spike in design (PKv1, 250 Kb) will also be used to specifically enhance coverage of the *TERT* gene promoter region. We now consistently find 3,200 genes at complete coverage (every base of the transcript ≥20x) representing a 34% increase in complete gene coverage of the targeted clinical genes. Using these same methods, spike in probes from IDT can be designed and validated for coverage of additional reportable sites in UTR or intronic regions as required for this offering. Libraries will be pooled at 10 samples per pool for capture hybridization. The resulting pools will be pooled 7 per Illumina NovaSeq lane (70 total samples per NovaSeq lane).

Illumina NovaSeq Sequencing: Illumina NovaSeq instrumentation will be employed to generate WES 150 bp paired-end sequence reads for all samples in a format of multiplexed pools to target an average coverage of approximately 100x to achieve at least 90% of bases covered at 20X. (~11 Gbp per sample).

Sequence Performance Metrics and Quality Assurance: HGSC-LIMS tracks sequence run set-up, status, and the battery of performance metrics. Real-time analysis (RTA) software provides an initial assessment of quality as soon as the run begins. A PhiX-DNA sequencing control is included on each flow cell. The NovaSeq instrument software (Control Software v1.7) monitors run performance, assessing cluster pass filter, read 2 Q30 score and PhiX error rate. For evaluation of potential batch effects and reproducibility of pipeline performance, a human DNA control (NA12878) and a blind duplicate sample are each included in every tenth plate of processed libraries. A capture analysis pipeline has been integrated with the Illumina analysis pipeline to provide sequence-based metrics for QA/QC. This pipeline reports the proportion of the aligned reads that map to the reference as well as to the targeted region, which is a measure of the effective capture enrichment. Performance is also evaluated based upon the distribution of coverage across the targeted bases; specifically, the proportion of targeted bases

covered at 10X, 20X coverage are carefully examined. In addition, to monitor the complexity of the capture library, read alignment data is used to identify and quantitate sequence reads that are likely to have arisen from PCR duplicates. Finally, a fully automated pipeline has been developed for determining concordance and contamination using genotype array data (ERIS) for WES samples. For each successfully sequenced sample, ERIS compares sequence data to genotypes from available GWA SNP arrays. Using an "e-GenoTyping" approach, ERIS screens all sequence reads for exact matches to probe sequences defined by the variant and position of interest. As stated above, Fluidigm SNPtrace array will be used in conjunction with the ERIS analysis for this project to provide gender relationship with other samples and assessment of identity and contamination. Additionally, using a batch analysis of 50 WES clinical validation samples and the above criteria we have determined that 98% of HGMD (pro 2019.2) variants and 99% of ClinVar hg37 (20190603) variants are represented at ≥20x coverage for clinical utility.

WES data processing and Somatic CAN calling: Reads from whole exome sequencing for a given sample will be curated for quality and adapters will be trimmed. The reads will be aligned against hg19 using bwa algorithm to generate one bam file per sample. The somatic variants, such as NF1 shallow deletions, will be called using paired tumor and blood normal from BAM files. Somatic copy number alterations will be predicted by applying copywriteR algorithm on blood normal (control) and tumor (sample) BAM files. Hg19.UCSC.add_miR.140312.refgene will used to map the copy number information to genes. GITSIC2.0 threshold of +/-0.3 will be applied to identify NF1 gain or loss of copy number respectively. Hence, copy number estimates for NF1 for tumors will be made by comparing against matched normal to provide somatic copy number estimations as CN log2 ratio (continuous variable) as well as GISTIC thresholded categories. These are integer values ranging from −2 to 2. Amplifications are represented by positive numbers: 1 means amplification above the amplification threshold (gain); 2 means amplification larger than the arm level amplifications observed in the sample (amplification). Deletions are represented by negative numbers: −1 means deletion beyond the threshold (shallow heterozygous deletion); −2 means deletions greater than the minimum arm-level copy number observed in the sample (homozygous deletion). GISTIC2.0 has been extensively tested for NF1 in the original article as well as in our earlier genomic profiling experiments. It has been reported that NF1 was robustly identified using gene-based scoring across all parameter combinations[153].

Software used:

**Program used in WES data QC and alignement**	**Version**	**URL**
Human Reference Genome	GRCh37	ftp://ftp.1000genomes.ebi.ac.uk/vol1/ftp/technical/reference/phase2_reference_assembly_sequence/hs37d5.fa.gz
BWA	0.7.15	https://github.com/lh3/bwa

GATK	3.4	https://software.broadinstitute.org/gatk/
Java	1.8	https://www.java.com/
Samtools/ HTSlib	1.6/1.9	https://www.htslib.org/
Sambamba	0.6.7	https://lomereiter.github.io/sambamba/
CopyWriteR	2.0.6	https://github.com/PeeperLab/CopywriteR
GISTIC2.0	2.0.22	ftp://ftp.broadinstitute.org/pub/GISTIC2.0/GISTIC_2_0_23.tar.gz
Gene Annotation	hg19.UCSC.add_miR.140312.refgene	https://github.com/bzhanglab/GISTIC2_example/blob/master/hg19.UCSC.add_miR.140312.refgene.mat
