# Supplementary material for: Endocrine-Sensitive Disease Rate in Postmenopausal Patients With Estrogen Receptor–Rich/ERBB2-Negative Breast Cancer Receiving Neoadjuvant Anastrozole, Fulvestrant, or Their Combination: A Phase 3 Randomized Clinical Trial
Source: JAMA Oncol. 2024 Jan 18;10(3):362–71. doi: 10.1001/jamaoncol.2023.6038 (PMC10797521; doi:10.1001/jamaoncol.2023.6038)
Supplement: Supplement 2. — eAppendix. eTable 1. Common Adverse Events possibly, probably, or definitely related to NET eTable 2. Characteristics of Patients with Ki67wk4||wk12>10% eTable 3. Characteristics of Patients with PAM50 Determination vs. Patients without PAM50 determination eTable 4. Patient and Tumor Characteristics for the PAM50 Subtype Analysis Cohort by Treatment Arm eTable 5. NET Outcomes by Treatment Arm in All Patients and by LumA and LumB Subtype eFigure 1. The ALTERNATE Trial Study Schema eFigure 2. Scatterplot of Paired Ki67pre and Ki67wk4 Levels eFigure 3. REMARK Diagram [file jamaoncol-e236038-s002.pdf]

Supplemental Online Content

Ma CX, Suman VJ, Sanati S, et al. Endocrine-sensitive disease rate in postmenopausal patients with estrogen receptor–rich/ERBB2-negative breast cancer receiving neoadjuvant anastrozole, fulvestrant, or their combination: a phase 3 randomized clinical trial. *JAMA Oncol.* Published online January 18, 2024. doi:10.1001/jamaoncol.2023.6038

eAppendix.

eTable 1. Common Adverse Events possibly, probably, or definitely related to NET

eTable 2. Characteristics of Patients with $Ki67_{wk4||wk12}$>10%

eTable 3. Characteristics of Patients with PAM50 Determination vs. Patients without PAM50 determination

eTable 4. Patient and Tumor Characteristics for the PAM50 Subtype Analysis Cohort by Treatment Arm

eTable 5. NET Outcomes by Treatment Arm in All Patients and by LumA and LumB Subtype

eFigure 1. The ALTERNATE Trial Study Schema

eFigure 2. Scatterplot of Paired $Ki67_{pre}$ and $Ki67_{wk4}$ Levels

eFigure 3. REMARK Diagram

This supplemental material has been provided by the authors to give readers additional information about their work.

© 2024 Ma CX et al. *JAMA Oncology.*

eAppendix.

Central Ki67 and ER assessment:
The CAP/CLIA certified Washington University Anatomic and Molecular Pathology Core (CLIA number 26D2013203) performed central Ki67 IHC on pre-treatment, Wk4, Wk12, and surgical FFPE sections and central ER IHC on pre-treatment and surgical FFPE sections using the Ventana Medical System platform. Monoclonal antibody clone 30-9 was utilized for Ki67 and clone SP1 for ER. To quantify the Ki67 index (% tumor cells positive for Ki67 staining), whole-slide sections were scanned on the VENTANA iScan Coreo Au slide scanner, followed by pathologist-guided imaging analysis using the VIRTUOSO software as described previously.[1,2] At least 200 tumor cells were counted. Cases with fewer than 200 tumor cells on the slide were considered non-informative for Ki67. ER staining and scoring were performed per American Society of Clinical Oncology/College of American Pathologists guidelines.[3] mPEPI was calculated on the resected tumor specimens from patients who completed their assigned NET.

mPEPI score determination:
Modified PEPI score was determined based on post-neoadjuvant endocrine therapy surgical staging (tumor size, lymph node status) obtained from surgical and pathology reports and Ki67 of residual tumor centrally tested as discussed above on surgical FFPE sections. The modified PEPI score was calculated according to the Table below.

PEPI and mPEPI Score Determination

Surgical Specimen	PEPI Points	Modified PEPI points
**Tumor size**		
T1/2	0	0
T3/4	3	3
**Node status**		
Negative or N1mic	0	0
Positive	3	3
**Ki67 level**		
0-2.7%	0	0
>2.7-7.3%	1	1
>7.3-19.7%	1	1
19.7-53.1%	2	2
>53.1%	3	3
**ER, Allred score**		
0-2	3	N/A
3-8	0	

Note that patients who have radiographic evidence of progression during neoadjuvant endocrine therapy who do not go on to surgery were considered to have a Non-0 mPEPI value.

Pathologic response following neoadjuvant chemotherapy:
Pathologic complete response is defined as no invasive breast cancer in the breast and axillary lymph nodes. Residual Cancer Burden (RCB) is determined based on surgery findings post neoadjuvant chemotherapy for patients who switched to chemotherapy due to on-treatment Ki67 over 10%, according to the MD Anderson Residual Cancer Burden Calculator (http://www3.mdanderson.org/app/medcalc/index.cfm?pagename=jsconvert3).

Tumor RNA-sequencing and PAM50 subtype determination:
Pre-treatment frozen biopsy cores with tumor cellularity of ≥50% were subjected to RNA extraction, and RNA-sequencing (RNA-Seq) data generation and analysis as previously described.[4] PAM50 subtype was determined from RNA-Seq data using the publically available PAM50 predictor after applying a subgroup-specific normalization method.[5,6]

© 2024 Ma CX et al. *JAMA Oncology.*

Data and Safety Monitoring and Data Quality:
The study enrolled patients from 179 institutions in the United States National Cancer Institute (NCI)'s National Clinical Trials Network (NCTN). The Alliance Data and Safety Monitoring Board (DSMB) reviewed the available trial data at each of its biannual meetings. Data collection and statistical analyses were conducted by the Alliance Statistics and Data Management Center (SDMC). Data quality was ensured by Alliance SDMC and study chairperson review following Alliance policies. An independent statistician verified the results of the primary endpoint analysis.

References:

1. Goncalves R, DeSchryver K, Ma C, et al: Development of a Ki-67-based clinical trial assay for neoadjuvant endocrine therapy response monitoring in breast cancer. Breast Cancer Res Treat 165:355-364, 2017
2. Ellis MJ, Suman VJ, Hoog J, et al: Ki67 Proliferation Index as a Tool for Chemotherapy Decisions During and After Neoadjuvant Aromatase Inhibitor Treatment of Breast Cancer: Results From the American College of Surgeons Oncology Group Z1031 Trial (Alliance). J Clin Oncol 35:1061-1069, 2017
3. Allison KH, Hammond MEH, Dowsett M, et al: Estrogen and Progesterone Receptor Testing in Breast Cancer: American Society of Clinical Oncology/College of American Pathologists Guideline Update. Arch Pathol Lab Med 144:545-563, 2020
4. Satpathy S, Jaehnig EJ, Krug K, et al: Microscaled proteogenomic methods for precision oncology. Nature communications 11:532-532, 2020
5. Parker JS, Mullins M, Cheang MC, et al: Supervised risk predictor of breast cancer based on intrinsic subtypes. J Clin Oncol 27:1160-7, 2009
6. Zhao X, Rødland EA, Tibshirani R, et al: Molecular subtyping for clinically defined breast cancer subgroups. Breast Cancer Res 17:29, 2015

© 2024 Ma CX et al. *JAMA Oncology.*

eTable 1. Common Adverse Events possibly, probably, or definitely related to NET*

Adverse Events*	Anastrozole (n=434)				Fulvestrant (n=430)				Anastrozole + Fulvestrant (n=434)			
	Grade				Grade				Grade			
	2	3	4	Total	2	3	4	Total	2	3	4	Total
Arthralgia	49 (11.3%)	4 (0.9%)	0	53 (12.2%)	34 (7.9%)	0	0	34 (7.9%)	45 (10.4%)	4 (0.9%)	0	53 (11.3%)
Hot flashes	41 (9.4%)	2 (0.5%)	0	43 (9.9%)	41 (9.5%)	1 (0.2%)	0	42 (9.8%)	38 (8.8%)	3 (0.7%)	0	41 (9.4%)
Fatigue	41 (9.4%)	1 (0.2%)	0	42 (9.7%)	36 (8.4%)	1 (0.2%)	0	37 (8.6%)	46 (10.6%)	1 (0.2%)	0	47 (10.8%)
Myalgia	29 (6.7%)	3 (0.7%)	0	32 (17.4%)	16 (3.7%)	1 (0.2%)	0	17 (4.0%)	17 (3.9%)	3 (0.7%)	0	20 (4.6%)
Anxiety	17 (3.9%)	0	0	17 (3.9%)	19 (4.4%)	1 (0.2%)	0	20 (4.7%)	14 (3.2%)	1 (0.2%)	0	15 (3.5%)

* Number of grade 2+ adverse events reported to be at least possibly related to NET in over 2% of the patients on a given treatment arm.

© 2024 Ma CX et al. *JAMA Oncology.*

eTable 2. Characteristics of Patients with $Ki67_{wk4||wk12}$>10%

	**Patients who switched to NCT** (n=167)	**Patients who chose to go to immediate surgery or off study** (n=114)
**Randomization arm**		
Anastrozole	65 (38.9%)	44 (38.6%)
Fulvestrant	58 (34.7%)	46 (40.4%)
Anastrozole + Fulvestrant	44 (26.3%)	24 (21.1%)
**Age at registration, years**		
45-59	56 (33.5%)	34 (29.8%)
60-69	90 (53.9%)	36 (31.6%)
70-95	21 (12.6%)	44 (38.6%)
**Race**		
White	133 (79.6%)	100 (87.7%)
Black/African American	18 (10.8%)	8 (7.0%)
Asian	7 (4.2%)	2 (1.8%)
American Indian/Alaska Native	0	1 (0.9%)
Not reported	9 (5.4%)	3 (2.6%)
**Hispanic or Latino**	26 (15.6%)	6 (5.3%)
**ECOG PS**		
0	138 (82.6%)	86 (75.4%)
1-2	29 (17.4%)	28 (24.6%)
**Body mass index (BMI)**		
Under/Normal Weight	39 (23.4%)	17 (14.9%)
Overweight	39 (23.4%)	26 (22.8%)
Obese	88 (52.7%)	71 (62.3%)
Not reported	1 (0.6%)	0
**Clinical T stage**		
T2	113 (67.7%)	87 (76.3%)
T3-4c	54 (32.3%)	27 (23.7%)
**Clinical N stage**		
N0	82 (49.1%)	58 (50.9%)
N1-3	85 (50.9%)	56 (49.1%)
**ER/PgR status**		
Pos/Pos	139 (83.2%)	100 (87.7%)
Pos/Neg	28 (16.8%)	14 (12.3%)
**Histologic grade**		
G1	10 (6.0%)	13 (11.4%)
G2	102 (61.1%)	64 (56.1%)
G3	53 (31.7%)	37 (32.5%)
Not reported	2 (1.2%)	0
**wk4/wk12 Ki67 leading to discontinuation of NET**		
10.1%-20%	86 (51.5%)	61 (53.5%)
20.1%-30%	28 (16.8%)	22 (19.3%)
30.1-100%	53 (31.7%)	31 (27.2%)

© 2024 Ma CX et al. *JAMA Oncology.*

eTable 3. Characteristics of Patients with PAM50 Determination vs. Patients without PAM50 determination

	PAM50 subtype obtained (n=753)	PAM50 subtype not obtained (n=545)	Fisher's exact test p-value
**Age at registration, years**			
45-59	225 (29.9%)	167 (30.6%)	0.28
60-69	336 (44.6%)	221 (40.6%)	
70-95	192 (25.5%)	157 (28.8%)	
**ECOG PS**			
0	601 (78.8%)	436 (80.0%)	0.94
1-2	152 (20.2%)	109 (20.0%)	
**Body mass index (BMI)**			
Under/Normal Weight	157 (20.8%)	128 (23.5%)	0.49
Overweight	217 (28.8%)	147 (27.0%)	
Obese	379 (50.3%)	269 (49.4%)	
Not reported	(0)	(1)	
**Clinical T stage**			
T2	557 (74.0%)	386 (70.8%)	0.23
T3-4c	196 (26.0%)	159 (29.2%)	
**Clinical N stage**			
N0	415 (55.1%)	338 (62.0%)	0.01
N1-3	338 (44.9%)	207 (38.0%)	
**Histologic grade**			
G1-2	581 (77.9%)	479 (88.9%)	< 0.001
G3	165 (22.1%)	60 (11.1%)	
Not reported	(7)	(6)	
**pre-Ki67**			
0-20%	247 (33.4%)	224 (45.8%)	< 0.001
20.1%-100%	493 (66.6%)	265 (54.2%)	
Not obtained	(13)	(56)	

© 2024 Ma CX et al. *JAMA Oncology.*

eTable 4. Patient and Tumor Characteristics for the PAM50 Subtype Analysis Cohort by Treatment Arm

Characteristics	Anastrozole N=265	Fulvestrant N=234	Anastrozole + Fulvestrant N=254
**Age at registration, years**			
45-59	84 (31.7%)	62 (26.5%)	79 (31.1%)
60-69	121 (45.7%)	107 (45.7%)	108 (42.5%)
70-95	60 (22.6%)	65 (27.8%)	67 (26.4%)
**Race**			
White	222 (83.8%)	186 (79.5%)	212 (83.5%)
Black/African American	21 (7.9%)	28 (12.0%)	15 (5.9%)
Asian	4 (1.5%)	5 (2.1%)	11 (4.3%)
American Indian/Alaskan Other	5 (1.9%)	0	1 (0.4%)
Not reported	2 (0.7%)	2 (0.9%)	1 (0.4%)
	11 (4.2%)	13 (5.6%)	14 (5.5%)
**Hispanic or Latino**	27 (10.2%)	22 (9.4%)	32 (12.6%)
**ECOG PS**			
0	207 (78.1%)	190 (81.2%)	204 (80.3%)
1-2	58 (21.9%)	44 (18.8%)	50 (19.7%)
**BMI**			
Under/Normal Weight	52 (19.6%)	43 (18.4%)	62 (24.4%)
Overweight	78 (29.4%)	65 (27.8%)	74 (29.1%)
Obese	135 (50.9%)	126 (53.9%)	118 (46.5%)
**Clinical T stage**			
T2	192 (72.5%)	172 (73.5%)	193 (76.0%)
T3-4c	73 (27.6%)	62 (26.5%)	61 (24.0%)
**Clinical N stage**			
N0	139 (52.5%)	133 (56.8%)	143 (56.3%)
N1-3	126 (47.6%)	101 (43.2%)	111 (43.7%)
**ER/PgR status**			
Pos/Pos	237 (89.4%)	214 (91.5%)	229 (90.2%)
Pos/Neg	28 (10.6%)	20 (8.5%)	25 (9.8%)
**Histologic grade**			
G1	44 (16.6%)	33 (14.1%)	51 (20.1%)
G2	156 (58.9%)	149 (63.7%)	148 (58.3%)
G3	62 (23.4%)	50 (21.4%)	53 (20.9%)
Not reported	3 (1.1%)	2 (0.9%)	2 (0.8%)
**Baseline Ki67**			
0%-2.7%	6 (2.3%)	5 (2.1%)	2 (0.8%)
2.8%-10%	13 (4.9%)	20 (8.6%)	25 (9.8%)
10.1%-20%	68 (25.7%)	49 (20.9%)	59 (23.2%)
20.1%+	173 (65.3%)	157 (67.1%)	163 (64.2%)
Not available	5 (1.9%)	3 (1.3%)	5 (2.0%)
**Intrinsic Subtype**			
Luminal A	139 (52.5%)	128 (54.7%)	127 (50.0%)
Luminal B	111 (41.9%)	89 (38.0%)	104 (40.9%)
HER2-enriched	14 (5.3%)	14 (6.0%)	17 (6.7%)
Basal-like	1 (0.4%)	3 (1.3%)	6 (2.4%)

© 2024 Ma CX et al. *JAMA Oncology.*

eTable 5. NET Outcomes by Treatment Arm in All Patients and by LumA and LumB Subtype

	N	mPEPI 0	mPEPI 1-3	mPEPI >3	$Ki67_{wk4\|\|wk12}$ >10% or PD	mPEPI indeterminate[1]	Other[2]
**All patients**							
Anastrozole	434	81 (18.7%)	104 (24.0%)	92 (21.2%)	116 (26.7%)	12 (2.8%)	29 (6.7%)
Fulvestrant	430	98 (22.8%)	109 (25.3%)	92 (21.4%)	110 (25.6%)	7 (1.6%)	14 (3.3%)
Anastrozole + Fulvestrant	433	89 (20.6%)	156 (36.0%)	66 (15.2%)	76 (17.5%)	27 (6.2%)	20 (4.6%)
**Luminal A**							
Anastrozole	139	36 (25.9%)	43 (30.9%)	33 (23.7%)	17 (12.2%)	2 (1.4%)	8 (5.8%)
Fulvestrant	128	38 (29.7%)	37 (28.9%)	22 (17.2%)	25 (19.5%)	0	6 (4.7%)
Anastrozole + Fulvestrant	127	31 (24.4%)	58 (45.7%)	21 (16.5%)	11 (8.7%)	4 (3.1%)	2 (1.6%)
**Luminal B**							
Anastrozole	111	10 (9.0%)	23 (20.7%)	24 (21.6%)	49 (44.1%)	1 (0.9%)	4 (3.6%)
Fulvestrant	89	16 (18.0%)	24 (27.0%)	18 (20.3%)	28 (31.5%)	2 (2.2%)	1 (1.1%)
Anastrozole + Fulvestrant	104	18 (17.3%)	43 (41.3%)	12 (11.5%)	22 (21.2%)	6 (5.8%)	3 (2.9%)

1: One or more of the elements to determine mPEPI was missing
2: Discontinue neoadjuvant treatment for reasons other than progressive disease or failed to undergo surgery while on study.

© 2024 Ma CX et al. *JAMA Oncology.*

eFigure 1. The ALTERNATE Trial Study Schema

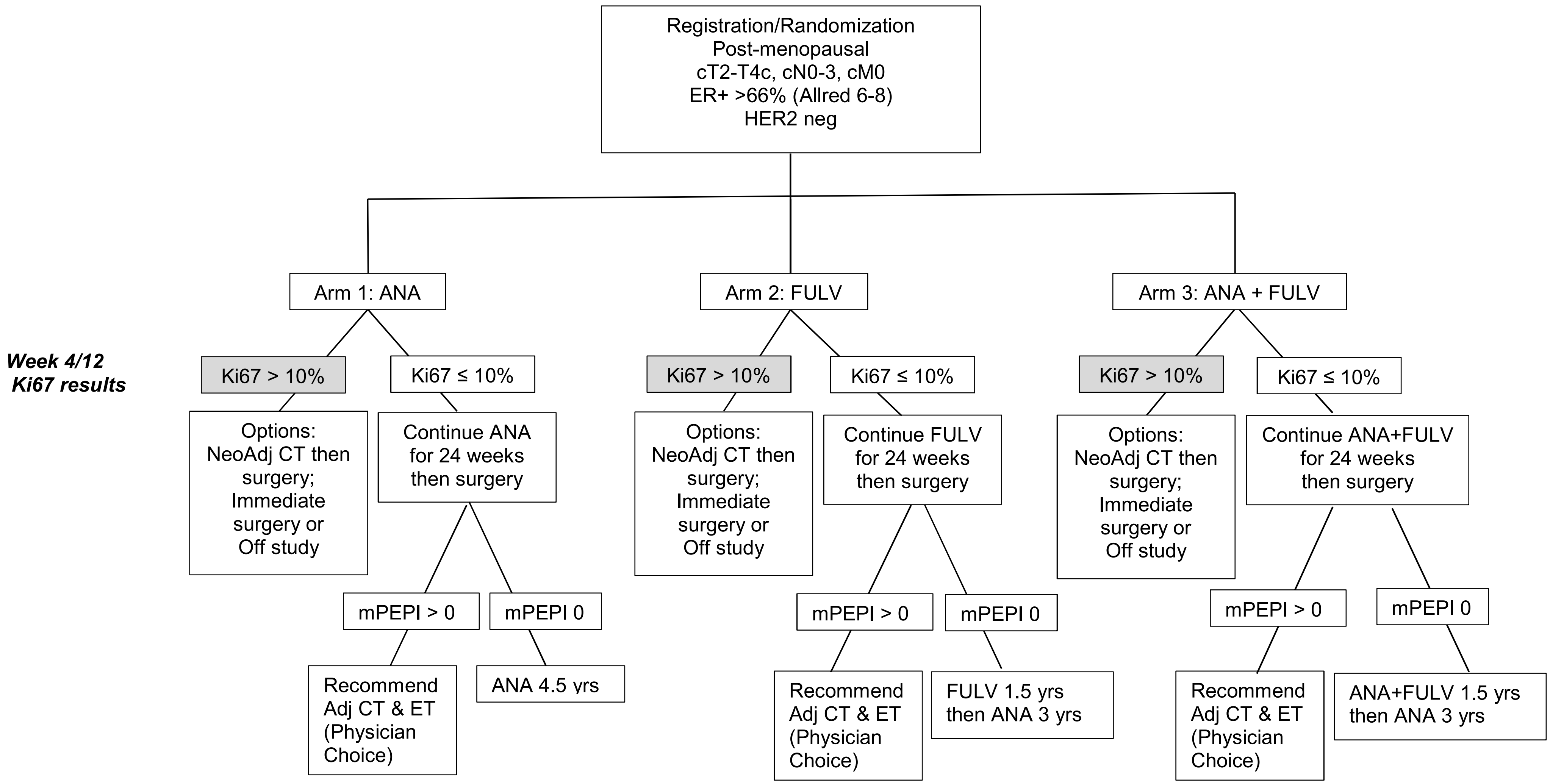

ANA, anastrozole; FULV, fulvestrant; CT, chemotherapy; ET, endocrine therapy; NeoAdj, neoadjuvant; Adj, adjuvant

© 2024 Ma CX et al. *JAMA Oncology.*

eFigure 2A. Scatterplot of Paired Ki67$_{pre}$ and Ki67$_{wk4}$ Levels by Treatment Arm Overall

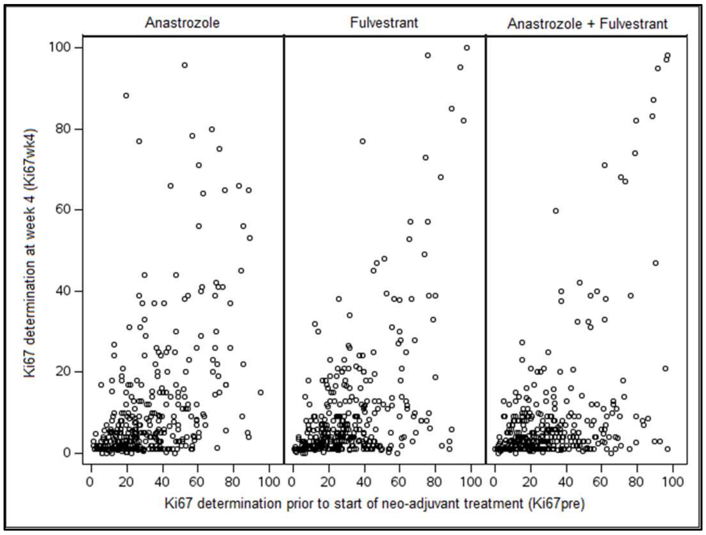

eFigure 2B. Scatterplot of Paired Ki67$_{pre}$ and Ki67$_{wk4}$ Levels by Treatment Arm: Luminal A and B

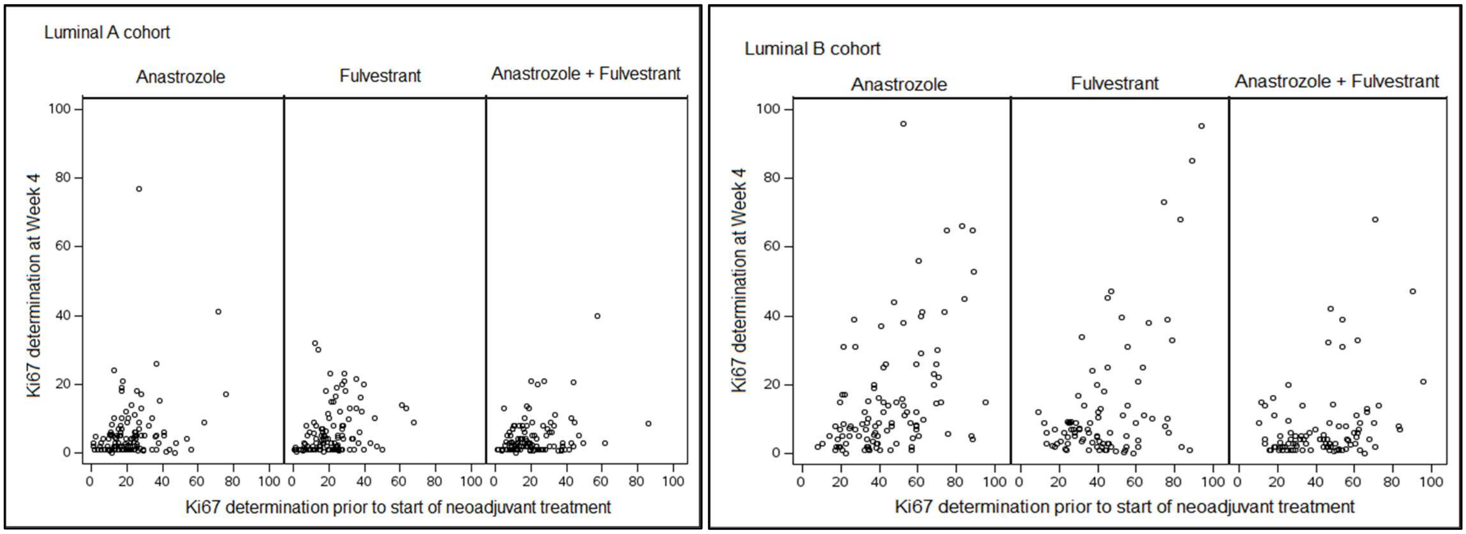

© 2024 Ma CX et al. *JAMA Oncology.*

eFigure 3. REMARK Diagram

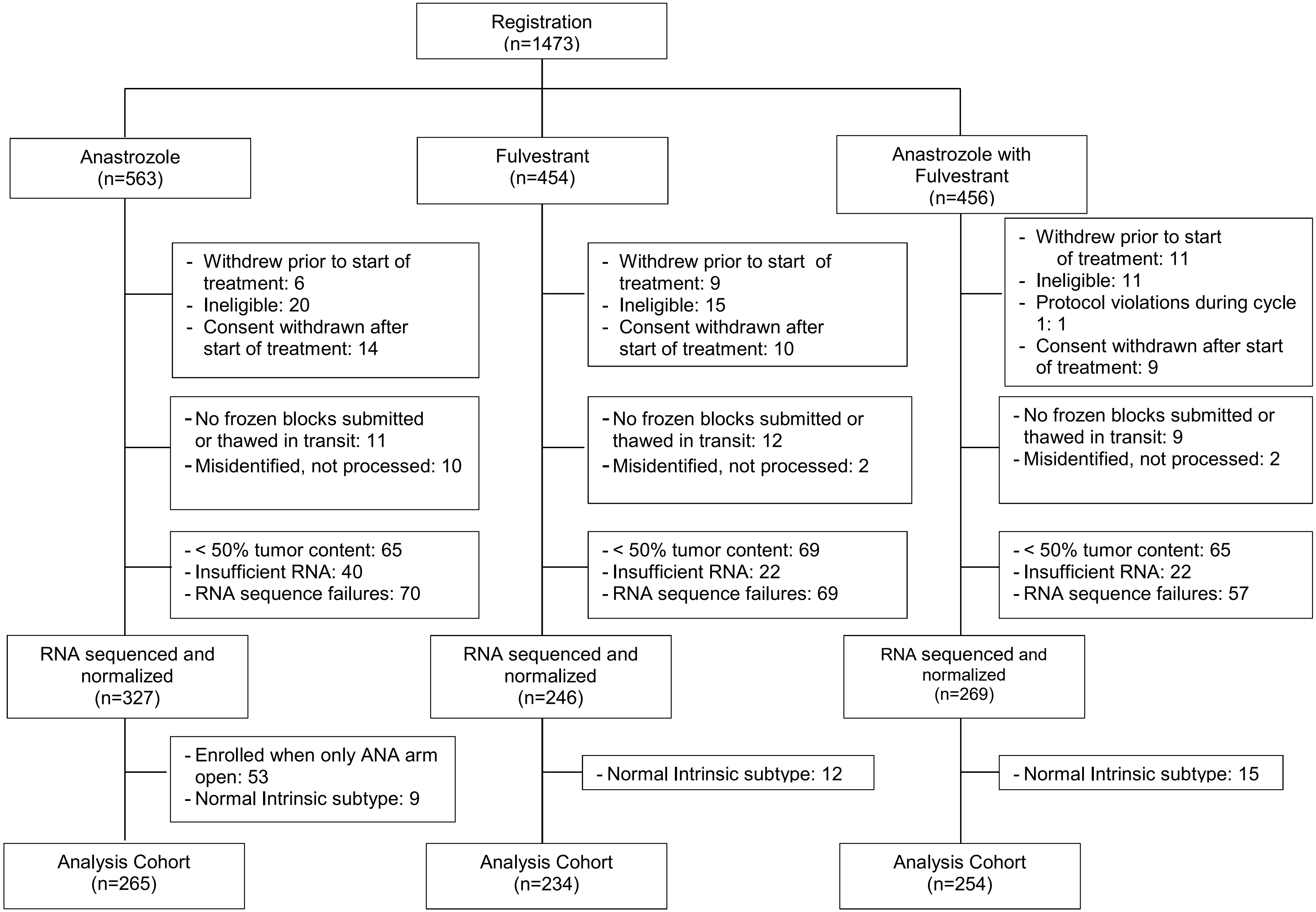

REMARK Diagram for RNA-Seq based PAM50 Subtype Determination

1,473 patients comprised the entire trial population (n=1,362 in the randomized phase and an additional n=111 in the A arm). For normalization purpose, pre-treatment RNA-Seq data from all eligible and consented patients were used. However, only those enrolled in the randomized phase were included in this analysis.

© 2024 Ma CX et al. *JAMA Oncology.*
