# Supplementary material for: Endocrine-Sensitive Disease Rate in Postmenopausal Patients With Estrogen Receptor–Rich/ERBB2-Negative Breast Cancer Receiving Neoadjuvant Anastrozole, Fulvestrant, or Their Combination: A Phase 3 Randomized Clinical Trial
Source: JAMA Oncol. 2024 Jan 18;10(3):362–71. doi: 10.1001/jamaoncol.2023.6038 (PMC10797521; doi:10.1001/jamaoncol.2023.6038)
Supplement: Supplement 3. — Data Sharing STATEMENT [file jamaoncol-e236038-s003.pdf]

Data Sharing Statement

Ma. Endocrine-Sensitive Disease Rate in Postmenopausal Patients With Estrogen Receptor–Rich/ERBB2-Negative Breast Cancer Receiving Neoadjuvant Anastrozole, Fulvestrant, or Their Combination. *JAMA Oncol*. Published January 18, 2024.
doi:10.1001/jamaoncol.2023.6038

Data
Data available: Yes
Data types: Deidentified participant data
How to access data: NCTN/NCORP Data Archive (https://nctn-data-archive.nci.nih.gov/)
When available: beginning date: 01-01-2024

Supporting Documents
Document types: None

Additional Information
Who can access the data: Anyone requesting data will go through a formal review process including verifying the availability of data, conducting a review of any existing agreements that may have implications for the project, and ensuring that any transfer is in compliance with the IRB.
Types of analyses: Upon formal review of request data will be made available for any purpose.
Mechanisms of data availability: Data will be made available with a signed data access agreement.
